# Oxygen Vacancies in β-MoO_3_ Mediate Imine Synthesis via Reductive Coupling of Nitro Compounds and Alcohols

**DOI:** 10.34133/research.0993

**Published:** 2025-12-09

**Authors:** Ziliang Yuan, Yijing Gao, Qingjie Tang, Jianguo Wang, Xun Li, Zehui Zhang

**Affiliations:** ^1^ Key Laboratory of Catalysis and Materials Sciences of the Ministry of Education, South-Central Minzu University, Wuhan 430074, P. R. China.; ^2^ Institute of Industrial Catalysis, State Key Laboratory Breeding Base of Green-Chemical Synthesis Technology, College of Chemical Engineering, Zhejiang University of Technology, Hangzhou 310032, P. R. China.; ^3^ Zhejiang Engineering Laboratory for Green Syntheses and Applications of Fluorine-Containing Specialty Chemicals, Institute of Advanced Fluorine-Containing Materials, Zhejiang Normal University, Jinhua 321004, P. R. China.

## Abstract

Organonitrogen chemicals with C=N bonds are one of the most important groups of chemicals with broad applications, but their synthesis via reductive coupling remains a great challenge, because of the favorable hydrogenation of C=N bonds into C–N bonds. In this study, a nitrogen-doped carbon-supported β-MoO_3_ catalyst with abundant oxygen vacancies (O_v_) was discovered to be robust in the reductive coupling of nitro compounds with biomass-derived alcohols toward the synthesis of organonitrogen chemicals, including imines and *N*-heterocycles with C=N bonds. The O_v_ in β-MoO_3_ serves a crucial role in the adsorption and activation of substrates via the electronic interaction between the negatively charged oxygen atoms in these substrates and the O_v_ sites in β-MoO_3_. The presence of O_v_ greatly lowers the energy barriers of the reductive coupling reaction, and the electron transfer from alcohols to nitro compounds is mediated by the Mo^5+^/Mo^6+^ redox cycle. Our method demonstrates excellent selectivity to C=N bonds and is effective for a wide substrate scope including the highly inert methanol and ethanol. This study highlights the use of non-noble metal oxides as alternatives to traditional metal nanoparticles for various challenging organic transformations.

## Introduction

Organonitrogen chemicals containing C=N bonds, including imines and nitrogen-containing heterocycles, serve as key intermediates in the synthesis of fine chemicals, pharmaceuticals, and molecular machines [1–4]. Traditionally, these compounds are produced via the acid-catalytic condensation of aldehydes or ketones [4–7], suffering from drawbacks in handling acidic waste and a narrow range of suitable aldehyde or ketone substrates [6,7]. Therefore, there has been a growing interest in developing novel methods for synthesizing organonitrogen chemicals with C=N bonds in recent years. Various alternative strategies have been developed for the synthesis of imines, including the oxidation or dehydrogenation of primary or secondary amines [4,8,9], the direct coupling of alcohols with amines [8,10,11], the reductive coupling of nitro compounds and aldehydes [12,13], and the hydroamination of alkynes using amines [14]. Notably, the direct transformation of alcohols and amines has emerged as a particularly attractive and efficient route for imine formation [10,15]. On the one hand, alcohols are easily accessible from both fossil-derived sources and renewable biomass, and the reaction typically produces only hydrogen (under inert conditions) or water (under an oxygen atmosphere) as by-products, aligning with environmentally friendly objectives. Conversely, this method facilitates the efficient synthesis of unsymmetrical imines with remarkable atom economy. Nevertheless, its widespread application is frequently constrained by the requirement of potent basic additives, such as KOH or potassium *tert*-butoxide, to attain satisfactory catalytic performance, which presents challenges to the principles of green and sustainable chemistry [11,16].

**Fig. 1.**
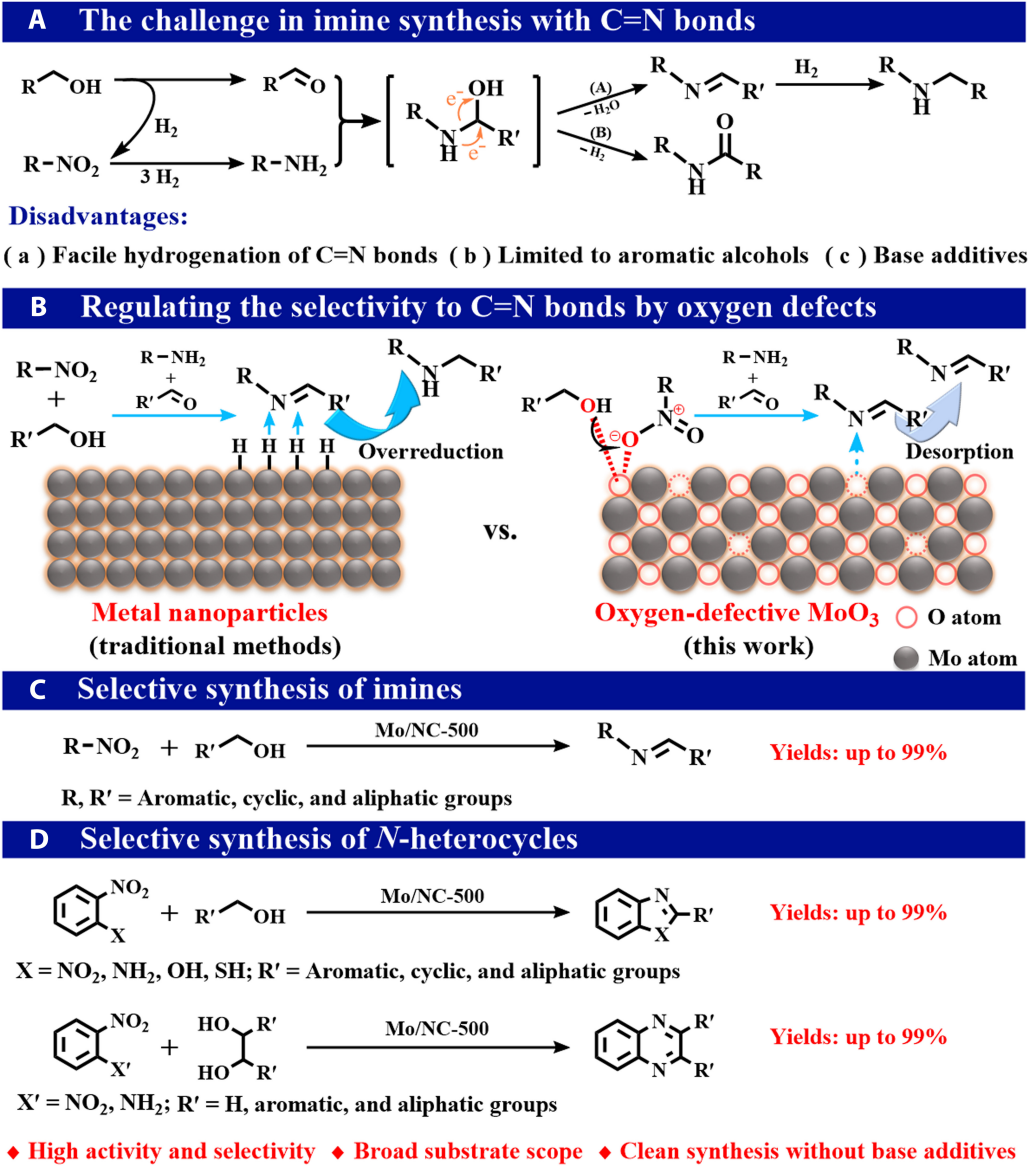
Reductive coupling involving nitro compounds and alcohol substrates. The challenge in imine synthesis with C=N bonds (A). Regulating the selectivity to C=N bonds by oxygen defects (B). Selective synthesis of imines (C). Selective synthesis of *N*-heterocycles (D).

Recently, tandem reactions without the purification of intermediates have attracted broad interest in sustainable chemistry [17–19]. The adoption of the borrowing hydrogen strategy for the direct formation of imines through the reductive coupling of nitro compounds with alcohols presents advantages, as it eliminates the necessity for supplementary reducing agents. [18–24]. The strategy proceeds through alcohol dehydrogenation to form carbonyl intermediates, followed by the in situ reduction of nitro compounds to primary amines and a final condensation step between the resulting amines and carbonyl species, yielding imines (Fig. 1A) [18–21]. Besides imines, other by-products such as amides and secondary amines can also be produced (Fig. 1A) [21–24]. Challengingly, the C=N bonds in imines can be easily hydrogenated into C–N bonds to generate secondary amines over the commonly supported metallic nanoparticles (Fig. 1B, left). In addition, the reported catalytic methods are often limited to active aromatic alcohols [21,22]. Utilizing unreactive aliphatic alcohols as feedstocks for producing organonitrogen chemicals is appealing, particularly in combination with heterogeneous catalysts based on non-noble metals. To realize the selective synthesis of imines from the reduction of nitro compounds with a broad range of aliphatic alcohols, as well as aromatic alcohols, advanced heterogeneous catalysts should be designed with a high activity toward the inert aliphatic alcohols, and they also should have weak or no interaction with imines, resulting in their fast desorption from the catalyst surface once formed.

Metal oxides such as CeO_2_ and MoO*_x_* have recently received great interest in the field of catalysis, due to their acid–base and redox properties, which constitute the largest family of heterogeneous catalysts [25–27]. Among different kinds of metal oxides, MoO_3_ is one of the most interesting metal oxides, serving as either the support or the catalyst for both oxidation and reduction reactions [28–30]. The key characteristic of MoO_3_ is its flexible valence state between Mo^6+^ and Mo^5+^ through the creation and elimination of oxygen vacancies (denoted as O_v_) [28,29]. For example, Prasomsri et al. [28] reported that the Mo^5+^/Mo^6+^ redox cycle mediated the hydrodeoxygenation of acetone into propylene, and the regeneration of oxygen in MoO_3_ was also observed. In recent years, the O_v_ in metal oxides has been discovered to enhance catalytic activity and product selectivity through the selective adsorption and activation of specific functional groups [28,29,31]. As both alcohols and nitro compounds can form stable structures with negatively charged oxygen atoms, it is believed that MoO_3_ with O_v_ would demonstrate a strong ability to adsorb and activate substrates, promoting the reductive transfer coupling of nitro compounds with alcohols to synthesize imines (Fig. 1B, right). The interaction between imines and negatively charged oxygen atoms near O_v_ sites is expected to be weak due to the bulky substituent on C=N moieties and their low polarity [31], resulting in their rapid desorption from the catalyst surface and inhibiting further hydrogenation of C=N bonds.

Targeting highly oxygen-deficient and dispersed molybdenum oxide, carbon was chosen as the support to stabilize small molybdenum oxide. The as-prepared catalysts with abundant oxygen vacancies (O_v_) were discovered to be effective for the reductive coupling of biomass-derived alcohols and nitro compounds toward the synthesis of imines (Fig. 1C). Furthermore, this catalytic system is also applicable to the construction of diverse value-added *N*-heterocycles, such as benzimidazoles, benzoxazoles, benzothiazoles, and quinoxalines (Fig. 1D). To the best of our knowledge, there are no reports of noble-metal-free heterogeneous catalysts that exhibit both broad efficacy and high selectivity for the synthesis of imines and *N*-heterocycles from a wide range of biomass-derived alcohols, particularly less reactive aliphatic alcohols such as methanol and ethanol.

## Results and Discussion

### Catalyst preparation and characterization

**Fig. 2.**
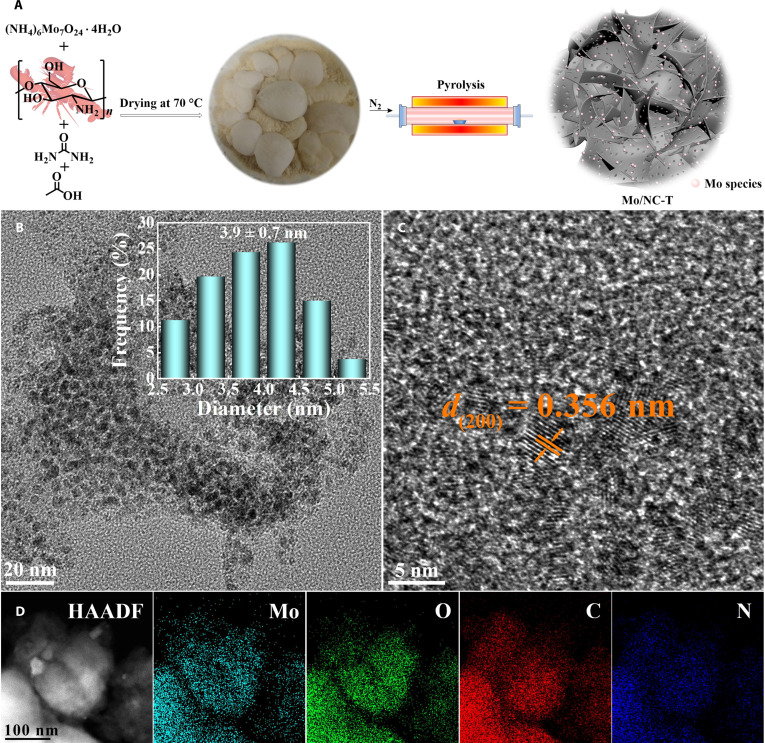
Procedure of the preparation of Mo/NC-T catalysts (A). The transmission electron microscopy (TEM) images (B), high-resolution TEM (HR-TEM) image (C), and high-angle annular dark-field scanning transmission electron microscopy (HAADF-STEM) and energy-dispersive x-ray spectroscopy (EDS) elemental mapping images (D) of Mo/NC-500.

Nitrogen-doped carbon-supported molybdenum oxides were synthesized through a scalable pyrolytic route. Briefly, a homogeneous mixture of chitosan, urea, and a certain amount of ammonium molybdate was first prepared in the presence of acetic acid to get a semitransparent paste (Fig. 2A). This precursor was subsequently subjected to thermal treatment under a N_2_ atmosphere at various temperatures to obtain the final catalysts, denoted as Mo/NC-T, where T refers to the pyrolysis temperature (450 to 800 °C). As shown in Table S1, the Mo content in the Mo/NC-T catalysts increased with rising pyrolysis temperature, which was attributed to the enhanced release of gases from precursor decomposition at elevated temperatures and the evolution of Mo species (MoO_3_ → MoO_2_ → Mo_3_C_2_), as discussed in a later section. For comparison, nitrogen-free analogs (Mo/C-T) were synthesized via the same protocol but without the inclusion of urea. N_2_ adsorption–desorption isotherms revealed type IV isotherms for all Mo/NC-T samples, indicative of mesoporous characteristics in accordance with IUPAC classification (Fig. S1 and Table S1). Moreover, the corresponding pore size distribution profiles confirmed the formation of a hierarchical porous network composed of interconnected micro- and mesopores.

As evidenced by the powder x-ray diffraction (XRD) patterns of Mo/NC-T (Fig. S2a), the molybdenum species underwent a phase evolution from MoO_3_ to MoO_2_ and ultimately to Mo_3_C_2_ upon pyrolysis at 800 °C. In detail, diffraction peaks at 2*θ* = 23.0°, 25.0°, 26.5°, 34.6°, 35.2°, 42.2°, and 53.4° were observed in the XRD pattern of Mo/NC-450, assigned to the (011), (200), (111), (211), (102), (220), and (400) crystalline planes of β-MoO_3_ (JCPDS PDF no. 47-1081) [32]. The XRD pattern of Mo/NC-500 was almost the same as that of Mo/NC-450, except that a new weak diffraction peak appeared at 26.0°, assigned to MoO_2_ generated from the reduction of MoO_3_ by reductive CO released from carbon support at a high pyrolysis temperature (MoO_3_ (s) + CO (g) → MoO_2_ + CO_2_ (g)) [33]. The newly formed MoO_2_ phase emerged as the predominant species in Mo/NC-600. Upon further increasing the pyrolysis temperature to 700 and 800 °C, Mo_3_C_2_ (JCPDS PDF no. 42-0890) was generated, attributed to the reductive transformation of MoO_2_ in the carbon-rich environment, following the reaction pathway 3MoO_2_ (s) + 10CO (g) → Mo_3_C_2_ (s) + 8CO_2_) [33,34]. The particle sizes of Mo/NC-T catalysts were also evaluated to be 4.3, 4.5, 5.1, 2.2, and 3.1 nm, respectively, according to the Debye–Scherrer equation [35]. Distinct from that of the Mo/NC-T catalyst, the XRD patterns of catalysts without N (Mo/C-T; see the materials and methods part for the preparation details; XRD shown in Fig. S2b) did not exhibit any Bragg peaks for Mo species when the pyrolysis temperature was below 700 °C, which may be attributed to their low crystallinity and thick carbon layer (Fig. S2b) [36]. Weak diffraction peaks for Mo_3_C_2_ were observed in the XRD pattern of Mo/C-700, and both Mo_3_C_2_ and Mo_2_C were observed in Mo/C-800 (Fig. S2b) [37]. The different diffraction peaks of Mo/C-800 and Mo/NC-800 (Fig. S2a vs. Fig. S2b) might be attributed to the impact of the nitrogen source, which was also reported by Jia et al. [34].

Scanning electron microscopy analysis of the Mo/NC-500 catalyst revealed a loosely packed, porous, and layered morphology (Fig. S3). Transmission electron microscopy (TEM) further showed the presence of uniformly dispersed nanoparticles with an average diameter of 3.9 ± 0.7 nm (Fig. 2B). The high-resolution TEM (HR-TEM) image (Fig. 2C) displayed clear lattice fringes with a spacing of 0.356 nm, attributed to the (200) crystallographic plane of β-MoO_3_ [32], consistent with XRD results. High-angle annular dark-field scanning transmission electron microscopy revealed that stabilized molybdenum oxides were homogeneously dispersed on the surface of the nitrogen-doped carbon layer (Fig. 2D). Energy-dispersive x-ray spectroscopy elemental mapping (Fig. 2D) confirmed the homogeneous dispersion of N, O, and Mo species throughout the carbon matrix in Mo/NC-500. In the TEM images of Mo/NC-T, nanoparticles were generally invisible, whereas well-defined particles with an average diameter of 4.3 ± 0.8 nm were observed in the Mo/NC-600 sample. HR-TEM analysis of Mo/NC-600 (Fig. S4d) revealed lattice fringes with a spacing of 0.241 nm, corresponding to the (200) plane of MoO_2_ [32]. TEM images of Mo/NC-700 and Mo/NC-800 (Fig. S4e to h) showed the presence of small nanoparticles with sizes in the range of approximately 2 to 4 nm. The HR-TEM images of both samples (Fig. S4f and h) exhibited lattice spacings of 0.245 nm, which can be assigned to the (102) crystallographic plane of Mo_3_C_2_ [34]. In contrast, the nanoparticle was observed only in TEM images of the Mo/C-T catalyst with pyrolysis temperatures above 700 °C (Figs. S5 and S6a vs. Fig. S6). As mentioned earlier, this may be attributed to their low crystallinity and thick carbon layer [36,38].

Raman spectroscopy was utilized to further investigate the structural features of carbon and the state of Mo species in Mo/NC-T (Fig. S7). All samples displayed 2 characteristic bands, located at approximately 1,332 to 1,355 cm^−1^ (D band) and 1,540 to 1,594 cm^−1^ (G band), corresponding to disordered carbon and graphitic carbon domains, respectively. These features collectively reflect the coexistence of amorphous and crystalline carbon structures within the catalyst matrix [39]. It was interesting to note that the D band had a blueshift, while the G band showed a redshift over the Mo/NC-T catalysts with an increase in pyrolysis temperature. The reason should be the gradual change of sp^2^ carbon in the graphitic structure into sp^3^ carbon in the disordered structure [39,40]. The area ratio of *A*_D_/*A*_G_ was calculated to be 1.39 for Mo/NC-450, which was lower than those for Mo/NC-500, Mo/NC-600, and Mo/NC-800 (1.64 to 1.72). The Raman peaks in the extended region from 100 to 1,000 cm^−1^ are attributed to the bending and stretching vibration modes of molybdenum oxides in the Mo/NC-T catalysts. Two weak peaks at 284 and 993 cm^−1^ were attributed to the M=O vibration, while the band at 820 cm^−1^ corresponded to Mo–O–Mo vibrations [41,42]. It was noted that the intensity of the peak at 820 cm^−1^ in the Raman spectrum of Mo/NC-500 was significantly lower than that in Mo/NC-450 and Mo/NC-600, indicating that Mo/NC-500 possessed the lowest abundance of Mo–O–Mo bonds among the 3 catalysts (Fig. S7). More importantly, 3 weak peaks with Raman shifts around 252, 717, and 950 cm^−1^ were the vibration peaks of MoO*_x_* bearing O_v_ according to Alsaif et al. [41] and Kuwahara et al. [42]. Regarding the Mo/NC-800 catalyst, the vibration of the Mo–C bond at 994 cm^−1^ was clearly observed [43].

**Fig. 3.**
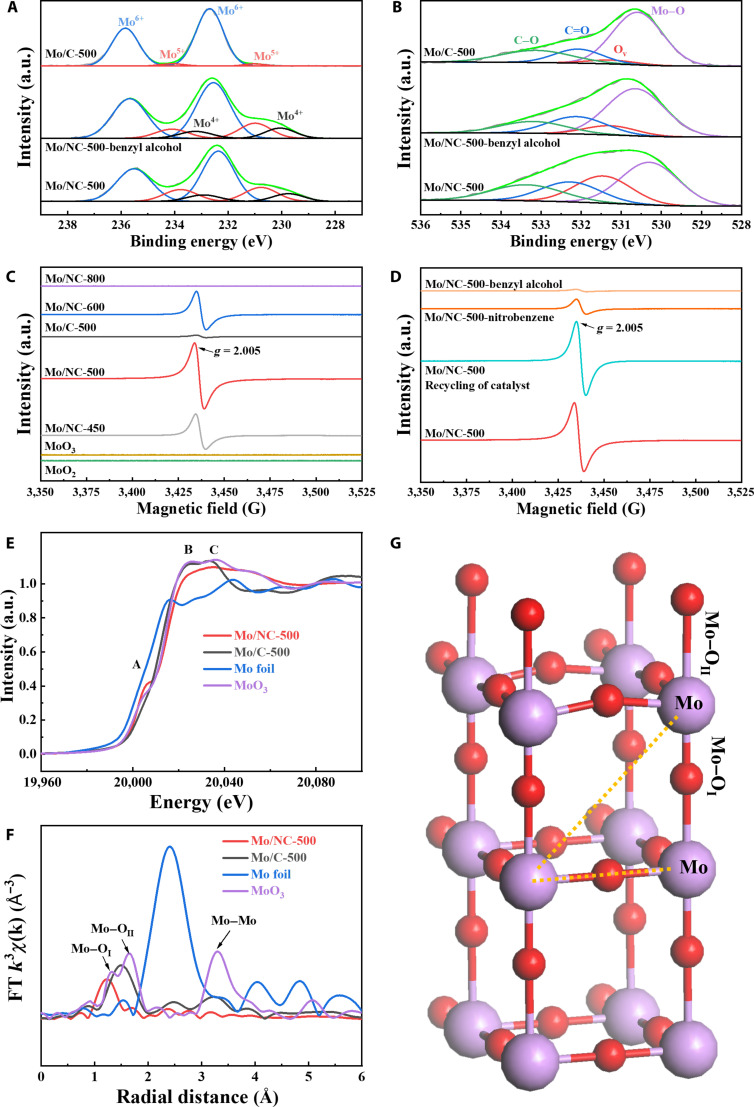
X-ray photoelectron spectroscopy (XPS) spectra of Mo 3d (A) and O 1s (B) in the Mo/NC-500, Mo/NC-500 (T), and Mo/C-500 samples. The electron paramagnetic resonance (EPR) spectra of different Mo samples (C) and the treatment of Mo/NC-500 catalysts (D). Mo/NC-500-benzyl alcohol and Mo/NC-500-nitrobenzene represent the Mo/NC-500 catalyst treated with benzyl alcohol and nitrobenzene, respectively. X-ray absorption near-edge structure (XANES) curves of the Mo foil, MoO_3_, Mo/NC-500, and Mo/C-500 at Mo K edge (E), the Fourier-transformed (FT) *k*^3^-weighted extended x-ray absorption fine structure (EXAFS) curves of the Mo foil, MoO_3_, Mo/NC-500, and Mo/C-500 at Mo K edge (F) and the structural model of β-MoO_3_ (G) (legend: red, O atoms; purple, Mo atoms).

X-ray photoelectron spectroscopy (XPS) was employed to investigate the surface elemental composition and valence states of the as-synthesized catalysts. The survey spectrum of the representative Mo/NC-500 sample revealed the presence of the Mo, C, N, and O elements, as evidenced by their characteristic binding energy peaks (Fig. S8). The Mo 3d spectra were fitted into 4 curves, corresponding to 4 valence states of Mo as follows: Mo^6+^ (232.3 to 232.6 and 235.5 to 235.7 eV), Mo^5+^ (230.6 to 230.8 and 233.8 to 234.0 eV), Mo^4+^ (229.6 to 229.7 and 232.8 to 232.9 eV), and Mo^2+^ (228.6 and 231.8 to 231.9 eV) (Fig. 3A, Fig. S9, and Table S2) [32,42]. According to the XPS results, the Mo species was mainly present as MoO_3_ (Mo^6+^) in Mo/NC-450, Mo/NC-500, and Mo/C-500, while MoO_2_ (Mo^4+^) was mainly present in Mo/NC-600. The average valence states of Mo were calculated to be 5.39, 5.96, 5.53, and 4.25 for the Mo/NC-450, Mo/C-500, Mo/NC-500, and Mo/NC-600 catalysts based on the peak areas of the Mo species with different valence states (Table S3), respectively. These XPS results were consistent with the results obtained from XRD. The obvious lower average valence state of Mo in Mo/NC-500 (5.53) compared with that in Mo/C-500 (5.96) clearly indicated that reduction of Mo^6+^ was easier in the presence of urea in the precursor, which could be due to the reduction of MoO_3_ at a high temperature (e.g., 450 to 600 °C) with CO and H_2_ from the decomposition of urea [33,44,45]. Thus, urea is important to create much more O_v_ in Mo/NC-T catalysts. The presence of Mo^6+^ and Mo^4+^ in Mo/NC-800 should be caused by the surface oxidation of Mo_3_C_2_ nanoparticles by oxygen during storage in the air [46].

The N 1s XPS spectra of the Mo/NC-T catalysts were fitted into 4 types of nitrogen as follows (Fig. S10 and Table S4): pyridinic N (398.2 to 398.6 eV), pyrrolic N (399.4 to 399.8 eV), graphitic N (400.7 to 401.3 eV), and oxidized N (401.8 to 403.0 eV) [47–49]. The total atomic percentage of nitrogen in the Mo/NC-T catalysts decreased from 30.8 to 18.1 at.% when increasing the pyrolysis temperature from 450 to 800 °C (Table S4), suggesting that many more nitrogen atoms were released at higher pyrolysis temperatures. Compared with Mo/C-500, using urea in the precursor greatly increased the nitrogen content in Mo/NC-500 (7.6 at.% vs. 27.4 at.%), which enhanced Mo oxide dispersion and promoted electron transfer during catalysis. The C 1s XPS spectra of the Mo/NC-T catalysts were deconvoluted into several distinct components (Fig. S11 and Table S5), corresponding to C–C/C=C (284.6 to 284.7 eV), C–O (285.6 eV), C=O (287.9 to 288.1 eV), and O–C=O species (289.0 to 289.1 eV) [48,49]. With increasing pyrolysis temperature, a progressive decline in the relative intensities of the oxygen-containing functional groups (C–O, C=O, and O–C=O) was observed, accompanied by a corresponding enrichment in C–C/C=C bonding, indicating enhanced carbonization (Table S5). Notably, a Mo–C bond peak centered at 283.4 eV appeared exclusively in the C 1s spectrum of Mo/NC-800, in agreement with XRD and TEM evidence for Mo_3_C_2_ formation. Similarly, the O 1s spectra were fitted with 4 oxygen species (Fig. 3B, Fig. S12, and Table S6): lattice oxygen in Mo–O (530.3 to 530.8 eV), oxygen vacancies (O_v_, 531.2 to 531.3 eV), C=O (532.1 to 532.2 eV), and C–O (533.1 to 533.2 eV) [41,50]. Mo/NC-500 had the highest peak area percentage of O_v_ at 24.1%, while the peak area percentages of O_v_ in Mo/NC-450 and Mo/NC-600 were calculated to be 10.7% and 18.3%, respectively (Table S6). No peak assigned to O_v_ could be fitted in the O 1s XPS spectrum of Mo/NC-800, as the Mo species in Mo/NC-800 was Mo_3_C_2_. In addition, the content of O_v_ in Mo/C-500 was found to be 4.2%, which was significantly lower than that of Mo/NC-500 (24.1%), in agreement with the average valence state as determined by XPS. Furthermore, electron paramagnetic resonance (EPR) spectra were collected to detect the content of O_v_ in these samples directly. As shown in Fig. 3C, an EPR signal with a *g* value of 2.005 is characteristic of O_v_ [40]. The relative abundance of O_v_, as quantified by EPR, aligns well with the trends observed in the O 1s XPS spectra. The EPR spectra show that Mo/NC-500 has the most abundant O_v_ with the strongest peak intensity, while Mo/C-500 has a very weak peak of O_v_. Interestingly, the commercial MoO_2_ and MoO_3_ gave no signal of O_v_.

X-ray absorption spectroscopy was employed to probe the local electronic and coordination environment of 2 representative catalysts. As depicted in Fig. 3E, the x-ray absorption near-edge structure (XANES) spectra of Mo/NC-500 and Mo/C-500 closely resemble that of the MoO_3_ reference while showing clear deviations from the spectrum of metallic Mo foil, indicating the predominant oxidation state of Mo in these samples. However, some obvious differences still exist between Mo/NC-500, Mo/C-500, and MoO_3_. The reference MoO_3_ and Mo/C-500 showed 2 main peaks at 20,025.7 and 20,037.2 eV (peaks B and C). Peaks B and C were broadened to one wide peak in Mo/NC-500, suggesting the dominant presence of disordered MoO_3_ species due to the abundant presence of O_v_ [51,52]. A distinct pre-edge feature at 20,005.6 eV (denoted as peak A) was identified in the XANES spectrum of Mo/NC-500, which originates from quadrupole-allowed 1s → 4d transitions and dipole-allowed transitions involving hybridized 1s-(5p, 4d) states [53]. This feature indicates the higher redox activity of Mo/NC-500 compared to those of MoO_3_ and Mo/C-500, consistent with its higher average Mo valence state as determined from spectral analysis.

The Fourier-transformed (FT) *k*^3^-weighted extended x-ray absorption fine structure (EXAFS) spectrum of MoO_3_ displays 3 main peaks at 1.3, 1.6, and 3.3 Å (Fig. 3F and Table S7). The former 2 peaks should be assigned to the Mo–O_I_ and Mo–O_II_ bonds, respectively [52]. The bond length of Mo–O_I_ is close to that of Mo=O, namely, terminal oxygen atoms (–Mo=O, Fig. 3G), while the bond length of Mo–O_II_ is close to that of the Mo–O–Mo bond, called bridge oxygen atoms (Mo–O–Mo, Fig. 3G). The peak at 3.3 Å was assigned to the Mo–Mo bond in MoO_3_ (Fig. 3F). Mo/NC-500 exhibited one main peak at ~1.3 Å, which was close to the Mo–O_I_ bond. Clearly, the peak at ~1.6 Å (Mo–O_II_ bond) was much weaker in the FT *k*^3^-weighted EXAFS spectrum of Mo/NC-500, suggesting that the O_v_ in Mo/NC-500 should be caused by the loss of the oxygen atoms in the bridge oxygen atoms in Mo–O–Mo bonds. However, Mo/C-500 has a large peak at a position that overlaps the Mo–O_I_ and Mo–O_II_ bonds observed in the standard MoO_3_. The results obtained from the FT *k*^3^-weighted EXAFS spectra are in good agreement with the average Mo valence states determined by XPS (Table S3). The very weak peak of the Mo–O_II_ bond in Mo/NC-500 clearly indicated that the oxygen atoms in Mo–O–Mo bonds were removed by the in situ formed reductive gases under high pyrolysis temperature. Compared with that in the Mo–O–Mo bond, the oxygen atom in the –Mo=O bond should be much more difficult to remove, as the double bond (Mo=O) had a much higher bonding energy than the single Mo–O–Mo bond. The EXAFS fitting parameters at the Mo K edge in MoO_3_ gave 2 kinds of oxygen-coordinated Mo: terminal oxygen atoms (Mo–O_I_, –Mo=O) with a coordination number (CN) of 1.1 ± 0.3 and bridge Mo–O–Mo oxygen atoms (Mo–O_II_, Mo–O–Mo) with a CN of 2.4 ± 0.9. Terminal oxygen atoms (Mo–O_I_, –Mo=O) with a CN of 1.4 ± 0.5 were fitted for Mo/NC-500. Still, no CN of Mo–O_II_ (Mo–O–Mo) was fitted, which also suggested that O_v_ should be predominantly formed at bridge oxygen sites (Mo–O_II_, Mo–O–Mo).

### Catalytic activity

The catalytic performance of the synthesized materials was evaluated using the reductive coupling of ethanol and nitrobenzene at 180 °C as a model reaction. Ethanol functions as both the solvent and the hydrogen source. The activity of the Mo/NC-T catalysts was found to be strongly influenced by the pyrolysis temperature, exhibiting a volcano-type trend (Table S8, entries 1 to 5). Mo/NC-500 demonstrated optimal performance, achieving a nitrobenzene conversion of 35.5% and a selectivity of 74.9% toward *N*-phenylethanimine, leaving aniline as the intermediate product and no other by-products detected (Table S8, entry 2). In contrast, the Mo-free nitrogen-doped carbon (NC-500) exhibited no catalytic activity (Table S8, entry 6), confirming that the Mo species are the essential active sites for the reductive coupling process. Certainly, nitrogen-doped carbon supports the dispersion and stability of β-MoO_3_, playing a positive synergistic role rather than acting as an independent active phase.

As revealed by the structural characterizations, increasing the pyrolysis temperature from 450 to 800 °C induced a progressive phase transformation of Mo species from MoO_3_ to MoO_2_ and ultimately to M_3_C_2_. Therefore, the dehydrogenative coupling of ethanol and nitrobenzene was also performed over the commercially available MoO_3_, MoO_2_, and Mo_2_C, respectively (Table S8, entries 7 to 9). MoO_3_ and Mo_2_C showed a very low activity in this reductive coupling reaction with low nitrobenzene conversions at 3.5% and 1.1% (Table S8, entries 7 and 9), respectively, while MoO_2_ was totally inactive (Table S8, entry 8). As far as Mo_2_C, it was reported that Mo_2_C had a catalytic activity in the dehydrogenation of alcohols [47]. Thus, the catalytic activity of Mo/NC-700 and Mo/NC-800 should originate from the small-sized carbide nanoparticles. It was unexpected to observe the huge difference in the catalytic performance between the commercial MoO_3_ and MoO_2_. After the reaction, we noted that MoO_3_ completely dissolved in ethanol, while MoO_2_ was stable. As shown in Fig. S13, the hot-filtration experiment confirmed that the activity of the commercial MoO_3_ was caused by the in situ formed homogeneous Mo complexes, which were indeed reported to be active for transfer hydrogenation reactions [23,54]. Compared to commercial MoO_3_, the prepared Mo/NC-500 catalyst demonstrated superior stability, as evidenced by hot-filtration tests (Fig. S14) and recycling experiments (Fig. S15). The XRD, TEM, XPS, and EPR characterization results of the recycled Mo/NC-500 catalyst indicate that its structure remains essentially consistent with that of the fresh catalyst, fully demonstrating the structural stability of the Mo/NC-500 catalyst (Fig. 3D, Figs. S16 and S17, and Tables S2 to S6). This enhanced durability is attributed to the stabilization effect imparted by the electron-rich nitrogen dopants within the catalyst matrix. Catalysts for hydrogenation usually suffer from deactivation during storage in the air due to the unavoidable oxidation of active sites, while in our case, the catalyst maintained robust catalytic performance even after 3 years of ambient air storage, confirming its long-term stability (Table S8, entry 10).

Mo/C-500 gave a low nitrobenzene conversion of 3.5% after 5 h at 180 °C, while that was high at 35.5% for Mo/NC-500 (Table S8, entry 11 vs. entry 2). The O 1s XPS spectra (Table S6) and EPR results (Fig. 3C) revealed that the content of O_v_ in Mo/C-500 was much lower than that in Mo/NC-500. At the same time, Mo/C-500 is also catalytic when the temperature is increased and the reaction time is prolonged (Table S8, entry 12). These findings highlight the pivotal role of oxygen vacancies (O_v_) in molybdenum oxides for facilitating the reductive coupling of nitrobenzene with ethanol over Mo/NC-T. The essential contribution of O_v_ was further confirmed by the observed inactivity of MoO_2_ in the absence of O_v_. Bearing the association of the catalytic activity with O_v_ in mind, it is easy to understand the difference in the catalytic activity of the Mo/NC-T catalysts. As Mo/NC-450 and Mo/NC-500 had a similar surface area with MoO_3_ as the major Mo species, the superior catalytic activity of Mo/NC-500 compared to that of Mo/NC-450 should be caused by the fact that Mo/NC-500 has much more O_v_. Meanwhile, Mo/NC-600 presented with MoO_2_ as the major Mo species, and the inferior activity of Mo/NC-600 compared to that of Mo/NC-500 should also be due to its lower O_v_ content. Although Mo/NC-600 has more O_v_ than Mo/NC-450 (Table S6), the catalytic activities of Mo/NC-600 and Mo/NC-450 were close to each other (Table S8, entry 1 vs. entry 3). This phenomenon can be attributed to the relatively lower specific surface area of Mo/NC-600 compared to that of Mo/NC-450, as a higher surface area generally facilitates more efficient mass transfer of reactants and products. Consequently, both surface area and the concentration of oxygen vacancies (O_v_) are critical factors influencing the catalytic performance of Mo/NC-T catalysts, with O_v_ serving as the dominant determinant.

Furthermore, the catalytic efficiency of Mo/NC-500 was benchmarked against those of commercially available noble metal catalysts such as Pt/C, Pd/C, and Ru/C, demonstrating its competitive performance (Table S8, entries 16 to 18). Although Ru/C delivered conversion comparable to that of Mo/NC-500, it suffered from serious side reactions (Table S8, entry 18). This is due to the different reaction mechanisms over 2 types of catalysts as depicted in Fig. 1B. The active H species on the surface of Ru nanoparticles over Ru/C can also be used for the hydrogenation of C=N bonds in imines (Fig. 1B, left). However, the mechanism of the reductive coupling reaction over Mo/NC-500 was based on the direct transfer of the H atom from ethanol to the nitro group in nitrobenzene after the selective adsorption and activation of ethanol by O_v_ (Fig. 1B, right). The targeted product of *N*-phenylethanimine with a C=N bond cannot generate a stable structure with oxygen anions to contact with O_v_; therefore, it can fast desorb off from the surface of Mo/NC-500 to get high selectivity of *N*-phenylethanimine (Fig. 1B).

As shown in Fig. S18, the conversion of nitrobenzene increased markedly with the elevation of the reaction temperature from 170 to 210 °C. Notably, Mo/NC-500 exhibited excellent thermal stability toward the C=N bond in *N*-phenylethanimine, maintaining product integrity even under high-temperature conditions. The evolution of product distribution over time was monitored at 200 °C (Fig. S19), revealing that nitrobenzene was progressively consumed and reached complete conversion within 10 h, accompanied by a high yield (up to 97%) of *N*-phenylethanimine. Aniline was detected as the sole intermediate, appearing in low molar proportions throughout the reaction, indicating that its condensation with acetaldehyde was a rapid step. Although the formation of imines via aldehyde–amine condensation is known to occur spontaneously under mild conditions, control experiments demonstrated that Mo/NC-500 effectively promoted this step as well, likely due to the intrinsic Lewis acidity of molybdenum oxide species within the catalyst (Fig. S20) [54,55].

### Substrate scope of the synthesis of imines

**Fig. 4.**
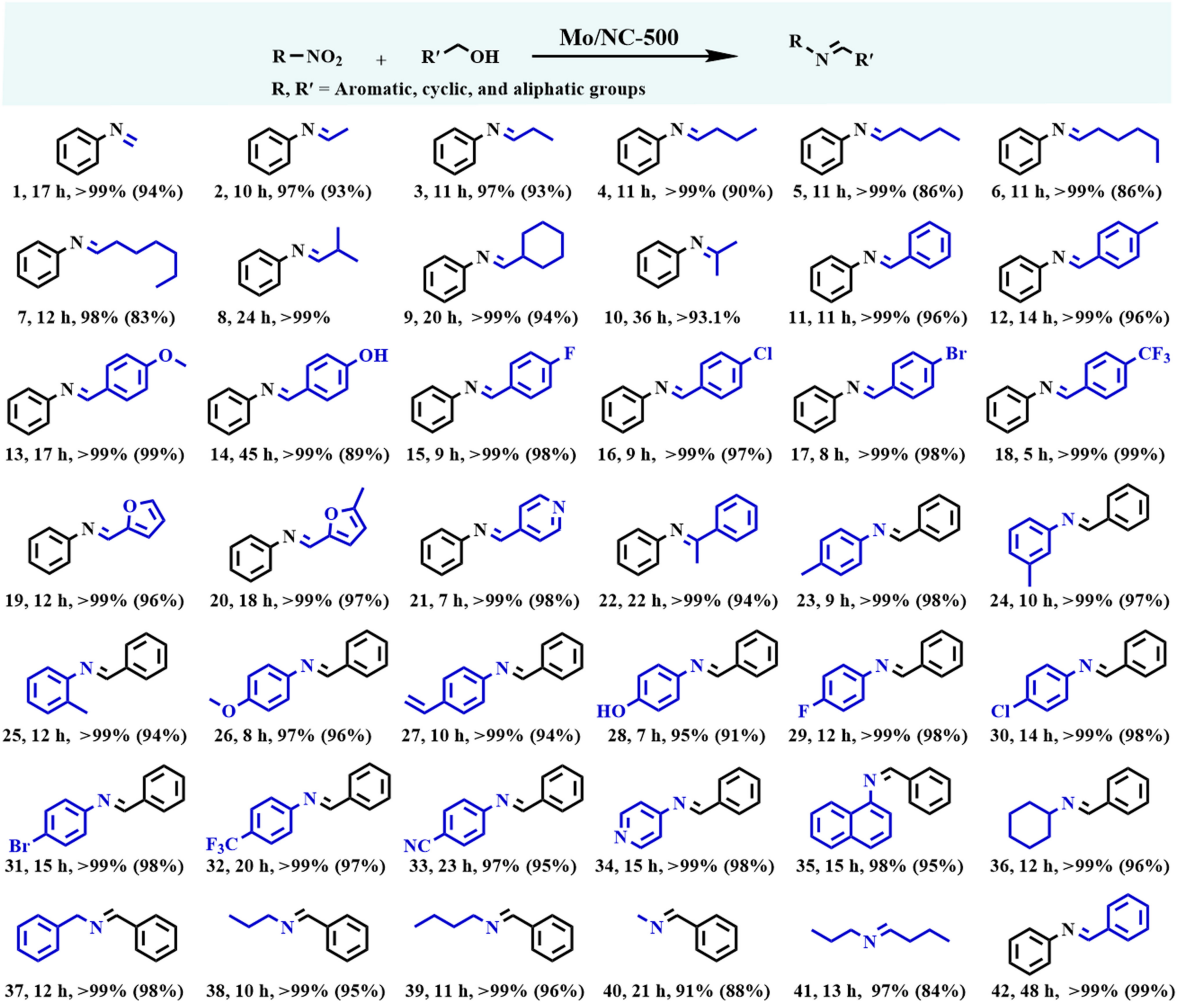
Preparation of imines via the reductive coupling between nitro compounds and alcohols. Reaction conditions: nitro compounds (1.0 mmol), Mo/NC-500 (2.1 mol.% Mo), aromatic alcohols (5.0 mmol), acetonitrile (10 mL), and 200 °C; For aliphatic alcohols, these were also used as the solvents; in 14, THF was the solvent; in 42, Aniline (1.0 mmol) and benzyl alcohol (1.0 mmol) were used. Yields in brackets are the isolated yields.

The substrate scope of the developed catalytic system was systematically evaluated for imine synthesis using Mo/NC-500 (Fig. 4). Firstly, the reductive coupling of nitrobenzene with various biomass-derived alcohols was examined. The reactions involving aliphatic alcohols proceeded efficiently, delivering the target imines in high yields ranging from 93.1% to 99% (Pro. 1 to 10). Methanol, the simplest alcohol with the highest dehydrogenation enthalpy among the nonbranched alcohols [56], demonstrated the weakest activity, requiring the longest time to attain a quantitative gas chromatography (GC) yield (Pro. 1 vs. Pro. 2 to 7); the isolated yield was 94% after 17 h. Furthermore, alcohols with greater steric hindrance exhibit lower reactivity (Pro. 8 and 9). The steric hindrance was much more obvious when *iso*-propanol as the secondary alcohol was used to couple with nitrobenzene (Pro. 10). To our knowledge, catalytic systems capable of efficiently synthesizing imines from inert and plentiful biomass-derived aliphatic alcohols—particularly methanol—remain unreported, especially those based on non-noble metal catalysts.

Certainly, Mo/NC-500 effectively catalyzed the reductive coupling of both aromatic and heteroaromatic alcohols, including primary alcohols (Pro. 11 to 21) and a secondary alcohol (Pro. 22) with nitrobenzene, yielding the corresponding imines in near-quantitative yields as determined by GC analysis when using the optimized acetonitrile as the solvent (Table S9). Notably, aromatic alcohols bearing electron-withdrawing substituents exhibited significantly higher reactivity compared to those with electron-donating groups (Pro. 15 to 18 vs. Pro. 12 to 14). Mo/NC-500 was also effective for the reductive coupling of nitrobenzene with heteroaromatic alcohols, including the biomass-derived furfuryl alcohols and pyridine-2-methanol (Pro. 19 to 21). Generally, it is a great challenge to activate heteroaromatic alcohols for transfer hydrogenation reactions, because of the strong affinity of heteroatoms to common metallic nanoparticle catalysts, inhibiting the adsorption of the –OH group [57]. However, it was not the case for Mo/NC-500, which only selectively adsorbed the –OH groups in heteroaromatic alcohols on O_v_ sites.

Then, the scope of nitro compounds was explored in the reductive coupling with benzyl alcohol (Pro. 23 to 40). The reactions involving aromatic, cyclic, and aliphatic nitro substrates proceeded efficiently in acetonitrile, yielding the corresponding imines in high GC yields ranging from 91% to 99% (Pro. 23 to 40). Steric hindrance was also observed for the reductive coupling of *o*/*p*/*m*-nitrotoluenes as well as 1-nitronaphthalene with benzyl alcohol (Pro. 23 and 24 vs. Pro. 25 and 35). Contrary to the trend observed with aromatic alcohols, substituted nitroarenes containing electron-donating groups exhibited higher reactivity in the dehydrogenative coupling with benzyl alcohol compared to those bearing electron-withdrawing substituents (Pro. 23 and 26 to 28 vs. Pro. 29 to 33). Besides the active nitroarenes, cyclic and aliphatic nitro compounds also smoothly participated in the reductive coupling with benzyl alcohol, affording the corresponding imines efficiently (Pro. 36 to 40). To our pleasure, the developed protocol proved effective for the reductive coupling between inert aliphatic alcohols and aliphatic nitro compounds (Pro. 41). Interestingly, the dehydrogenative coupling of benzyl alcohol with aniline afforded the corresponding imine in quantitative yield (Pro. 42), albeit requiring an extended reaction time of 48 h, compared to only 11 h needed for the reductive coupling of nitrobenzene with benzyl alcohol. These observations support the hypothesis that the reductive coupling of nitro compounds with alcohols proceeds via a transfer hydrogenation mechanism. A significant advantage of the present catalytic system is its high tolerance toward reducible functional groups such as halogens, nitriles, hydroxyls, and vinyl groups (Pro. 17, 18, and 27 to 33), which can be attributed to the selective adsorption of substrates through the interaction of negatively charged oxygen atoms with oxygen vacancy sites (O_v_) on Mo/NC-500.

### Synthesis of *N*-heterocycles

**Fig. 5.**
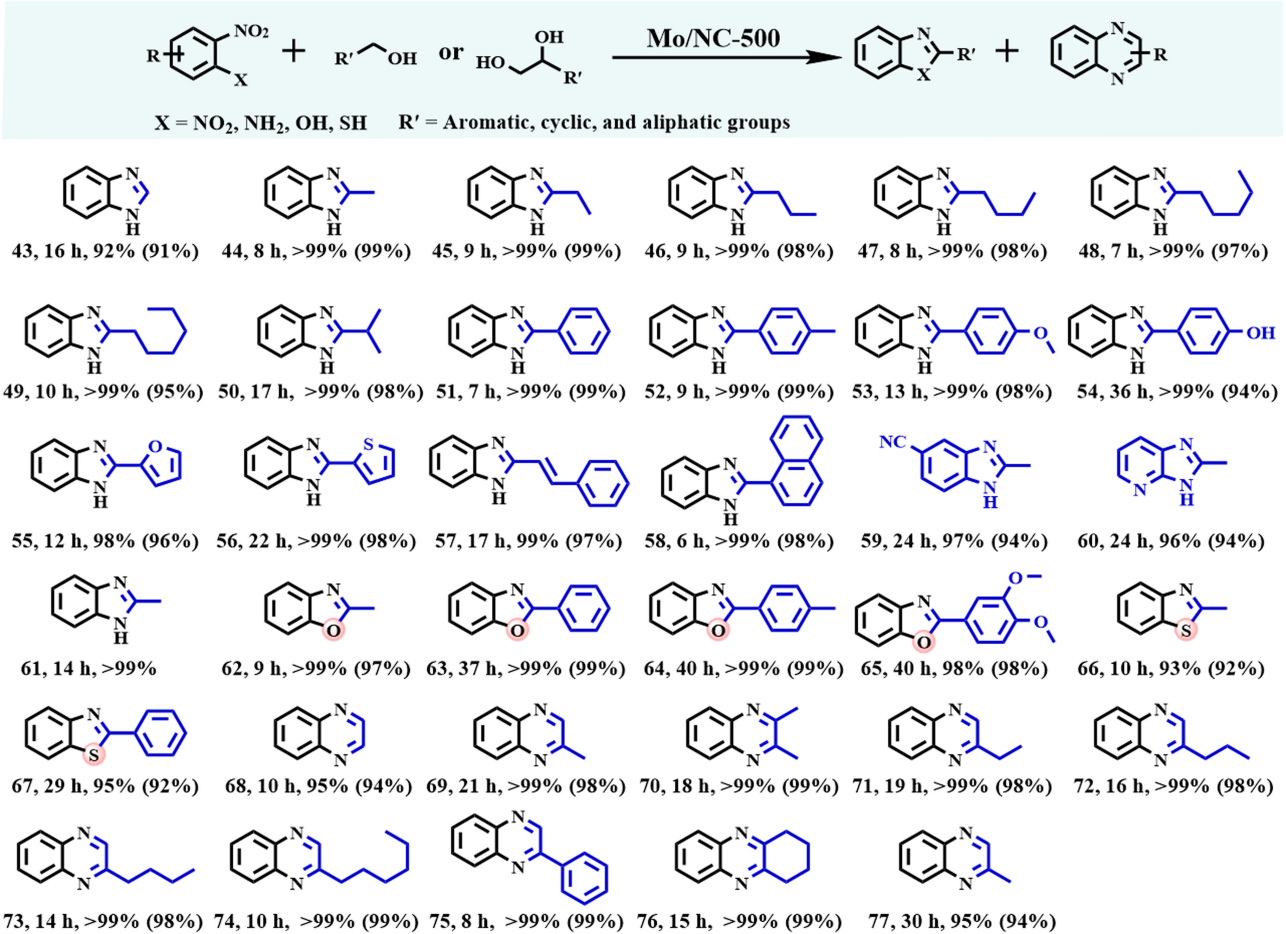
Synthesis of *N*-heterocycles from nitro compounds and alcohols. Reaction conditions: nitro compounds (1.0 mmol), aromatic alcohols (5.0 mmol), acetonitrile (10 mL), Mo/NC-500 (2.1 mol.% Mo), and 200 °C; the aliphatic alcohols will be used as solvents, and the 1, 2-diols are used in 10 mmol; in 54, tetrahydrofuran was the solvent; in 61 1, 2-dinitrobenzene was the substrate; in 77, hydroxyacetone (10.0 mmol) was used. Yields in brackets are the isolated yields.

Encouraged by the outstanding catalytic performance of Mo/NC-500 in imine synthesis, we further explored its applicability in the construction of nitrogen-containing heterocycles, such as benzazoles and quinoxalines (Fig. 5), which yielded results superior to those of O_v_-rich metallic oxide catalysts (Table S10) [15,21,31]. Specifically, the synthesis of benzazoles was investigated through the dehydrogenative coupling of alcohols with nitrobenzene derivatives bearing 2-NH_2_/SH/OH/NO_2_ substituents (Pro. 43 to 67). To our pleasure, the reductive coupling of biomass-derived alcohols including aliphatic alcohols as well as aromatic alcohols with 2-nitroaniline proceeded smoothly, affording the corresponding 2-substituted benzimidazoles with excellent yields (Pro. 43 to 61). In addition, benzimidazoles were efficiently synthesized via the reductive coupling of 1,2-dinitrobenzene with aliphatic alcohols (Pro. 61). Likewise, the coupling of 2-nitrophenol or 2-nitrothiophenol with both aromatic and aliphatic alcohols proceeded smoothly, yielding the corresponding benzoxazoles and benzothiazoles in excellent yields (Pro. 62 to 67).

Similar to benzazoles, quinoxalines (characterized by a heterocyclic framework containing 2 nitrogen atoms) serve as valuable scaffolds in the synthesis of fine chemicals, agrochemicals, and pharmaceuticals [58–60]. A direct and sustainable approach to synthesizing quinoxalines from the reductive coupling of 2-nitroaniline with biomass-derived 1,2-diols is highly desirable, yet remains challenging. Existing methods typically rely on homogeneous noble metal catalysts in combination with stoichiometric amounts of air-sensitive bases [59,61]. Remarkably, the Mo/NC-500 catalyst exhibited high activity for this transformation, enabling the synthesis of quinoxalines (Pro. 68 to 77) under additive-free conditions.

### Practical potential of the developed method

The potential for practical utility of our developed method was further investigated. As previously demonstrated, Mo/NC-500 exhibited catalytic activity in both the reductive coupling of nitroarenes with benzyl alcohol and the dehydrogenative coupling of benzyl alcohol with anilines. This dual functionality suggests a promising application in selectively producing C=N-containing compounds from mixtures of nitroarenes and anilines, an approach of considerable relevance to the chemical and pharmaceutical industries. For example, the reaction of nitro compounds, anilines, and alcohols at a molar ratio of 1:2:3 produced *N*-benzylideneaniline or 2-phenylbenzimidazole with nearly quantitative yields without the use of excessive alcohols (Fig. S21a). Secondly, gram-scale experiments were performed. Some representative products including 2, 11, 44, 51, and 65 were gram-scale-produced with high isolated yields (92% to 98%), demonstrating good potential in the large-scale production of imines and *N*-heterocycles in the chemical industry (Fig. S21b).

Furthermore, the developed method was applied for the gram-scale synthesis of some representative bioactive molecules. In the synthesis of benzazole-based bioactive molecules, 2-(3,4-dimethoxyphenyl)-5-fluorobenzoxazole (Pro. 85, antitumor activity), 5-chloro-2-(4-methylphenyl) benzoxazole (Pro. 86, DNA topoisomerase II inhibitor), and 4-(1*H*-benzimidazol-2-yl)-2-methoxyphenol (Pro. 87, spasmolytic activity) bioactive molecules were successfully produced at 1.2 to 7.0 g via the reductive coupling strategy over Mo/NC-500 (Fig. S21c) [62,63]. Moreover, the complex 1-(4-methoxybenzyl)-2-(4-methoxyphenyl)-1*H*-benzo[*d*]imidazole pharmaceutical (Pro. 89, nonnucleoside inhibitors of HIV-1 reverse transcriptase) [63] was also successfully prepared by a 2-step method, including the first reductive coupling process to generate Pro. 88 over Mo/NC-500, followed by the N-alkylation of Pro. 88 with *p*-anisaldehyde using formic acid as the hydrogen donor over our previously reported acid-resistant Co@CN-800 catalyst (Fig. S21d) [64].

### Kinetics and mechanism studies

As listed in Table S11, although Mo/NC-500 had the ability to promote the dehydrogenation of alcohols, the reduction of nitro compounds with alcohols should mainly proceed via the transfer hydrogenation mechanism. Furthermore, when nitrosobenzene and *N*-phenylhydroxylamine were deliberately introduced as substrates, we observed their complete transformation into the corresponding imine products under the same reaction conditions (Table S8, entries 19 and 20). These results clearly indicate that nitrobenzene, *N*-phenylhydroxylamine, aniline, and benzaldehyde act as key intermediates in the reaction pathway. Kinetic studies were conducted for both the reductive coupling of nitrobenzene with benzyl alcohol and the dehydrogenative coupling of benzyl alcohol with aniline. For the reductive coupling pathway, the reaction rate constants were calculated to be 0.0427, 0.0818, and 0.1349 h^−1^ at temperatures of 160, 170, and 180 °C, respectively (Fig. S22a). The apparent activation energy of the reductive coupling of nitrobenzene with benzyl alcohol was calculated to be 93.9 kJ/mol. The apparent activation energy of the dehydrogenative coupling of benzyl alcohol and aniline was obtained to be 95.8 kJ/mol by a similar method (Fig. S22b). The similar apparent activation energies of the 2 reactions revealed that the cleavage of α-C_sp3_–H or –O–H bonds in alcohols should be the rate-determining step in the synthesis of imines as well as *N*-heterocycles from the reductive coupling of alcohols and nitro compounds via the transfer hydrogenation mechanism. Furthermore, isotope-labeling experiments were performed to compare the activity of α-C_sp3_–H and –O–H in alcohols during the transfer hydrogenation process.

As shown in Fig. S22c, the kinetic isotope effect (KIE) value of CD_3_OD/CH_3_OH (3.04) was much larger than the KIE value of CH_3_OD/CH_3_OH (1.57). By deducting the contribution of –OD in CD_3_OD, the KIE value of CD_3_OH/CH_3_OH should be approximately 2.47, which revealed that the cleavage of α-C_sp3_–H from alcohols was the rate-determining step, requiring overcoming a much higher energy barrier as stated in the following density functional theory (DFT) calculation. It is known that the H^+^ in the –OH group was the active hydrogen, which can move easily either to the basic sites in the catalyst or directly to the nitro group [11,16]. As the 2 types of coupling reactions have similar apparent activation energies, the higher reaction rate of the reductive coupling of benzyl alcohol with nitrobenzene than that with aniline (0.1349 h^−1^ vs. 0.0417 h^−1^ at 180 °C) was also due to a different mechanism of the release of the 2 H atoms from alcohols. For the reductive coupling of benzyl alcohol and nitrobenzene, nitrobenzene served as the hydrogen acceptor to directly capture the H atoms from benzyl alcohol. However, the dehydrogenative coupling of benzyl alcohol and aniline to generate a H_2_ molecule requires the cleavage of α-C_sp3_–H and –O–H bonds to generate H species on the surface of MoO*_x_* and then the combination of the in situ generated hydrogen species (H^*δ*+^ and H^*δ*−^) to generate a H_2_ molecule, followed by the desorption step from the catalyst surface.

To further confirm the importance of O_v_ in the reductive coupling of nitro compounds with alcohols, several controlled experiments were carried out. First, the Mo/NC-500 catalyst was treated with nitrobenzene or benzyl alcohol under the reaction conditions and then was analyzed by EPR technology. As shown in Fig. 3D, the peak intensity of O_v_ greatly decreased in the catalysts treated with nitrobenzene or almost disappeared after being treated with benzyl alcohol. The XPS spectrum of the Mo/NC-500 catalyst treated with benzyl alcohol was also collected. As shown in Fig. S12 and Table S6, the peak area percentage of O_v_ greatly decreased from 24.1% for Mo/NC-500 to 9.5% for the treated catalyst. Meanwhile, the peak area percentage of Mo–O–Mo species and the valence states of Mo in the treated Mo/NC-500 catalyst were both higher than those in the fresh Mo/NC-500 catalyst (Fig. S9 and Table S3). The Raman spectra of Mo/NC-500 treated with benzyl alcohol were also collected and compared with that of the fresh catalyst (Figs. S22d and S7). It was noted that the intensity of the Raman shift at 820 cm^−1^ assigned to the Mo–O–Mo vibration peak was much stronger in the Mo/NC-500 catalyst treated with benzyl alcohol [41], while the peaks for –Mo=O did not change significantly. The above findings suggest that the reductive coupling between alcohols and nitro compounds is initiated by the coulomb interaction between oxygen atoms from substrates and the bridging oxygen vacancy sites on the Mo/NC-500 surface. This mechanistic insight is consistent with the structural information obtained from XAFS results.

**Fig. 6.**
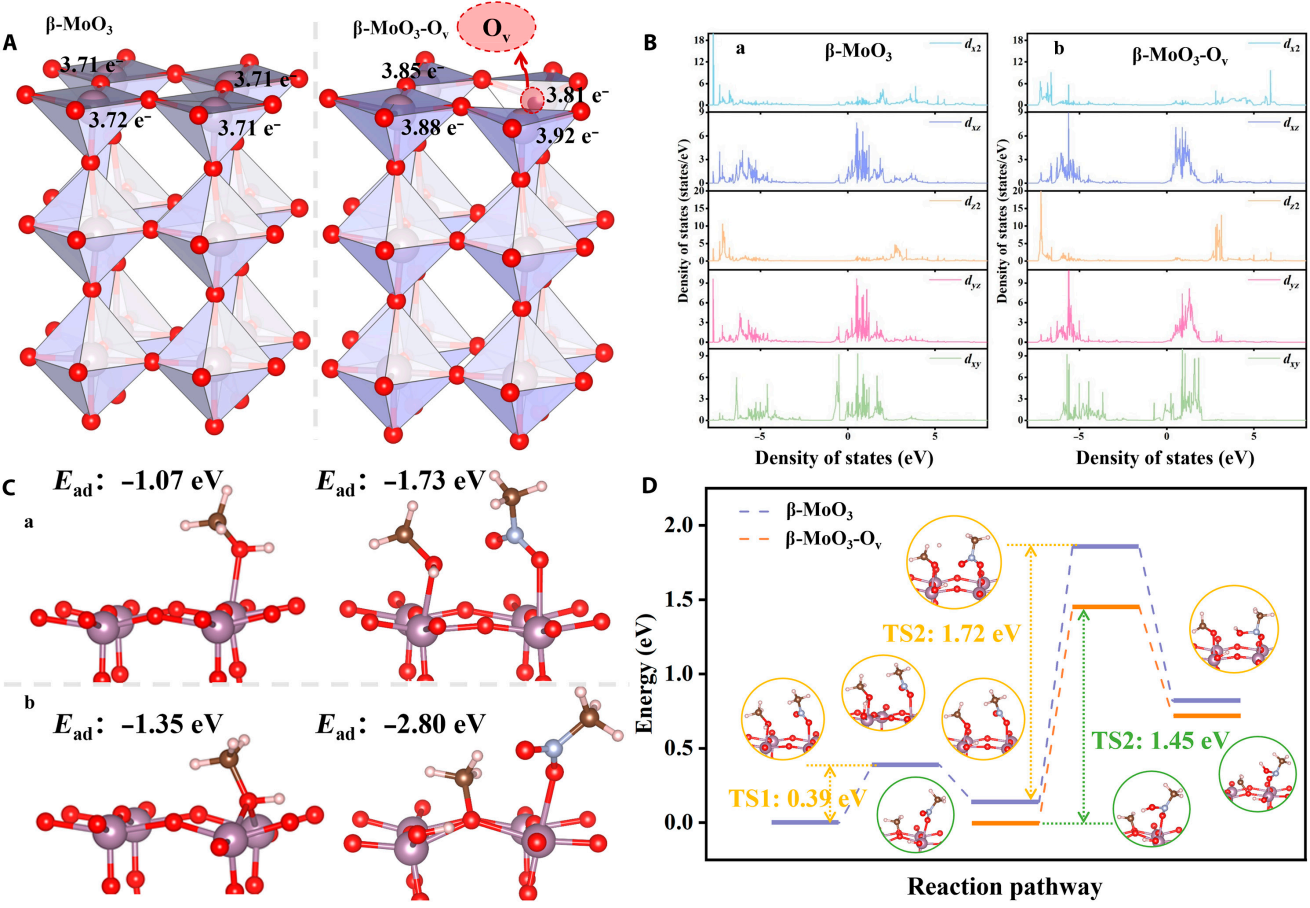
Density functional theory (DFT) calculation of the perfect β-MoO_3_ (200) (a, β-MoO_3_) and oxygen-defective β-MoO_3_ (200) (b, β-MoO_3_-O_v_). The optimal structures of oxygen-defective β-MoO_3_ (200) (A). The density of states calculations of a and b (B). The adsorption structures and the corresponding adsorption energies of CH_3_OH on the surfaces of a and b (C). Potential energy profiles of the transfer hydrogenation process of methanol on the surfaces of a and b (D) (legend: red, O atoms; purple, Mo atoms; pink, H atoms; brown, C atoms).

DFT calculation was then used to give more insights into the mechanism of the O_v_-promoted reductive coupling reactions over Mo/NC-500. According to the XRD and HR-TEM results of Mo/NC-500 (Fig. 2C and Fig. S2), the exposed (200) crystalline planes of β-MoO_3_ were considered the appropriate surface model for DFT calculation (Fig. S23), which has 2 different exposed oxygen atoms (–Mo=O and Mo–O–Mo) as shown in Fig. 3E and F and Fig. S20. The Bader charge analysis reveals that the surface Mo atoms near the O_v_ sites are rich in more electrons (Fig. 6A), which resemble Mo^5+^ atoms. The density of states calculations shows that the antibonding orbital peaks of surface Mo atoms near the O_v_ sites are stronger than those in perfect β-MoO_3_ (Fig. 6B), which revealed that the presence of O_v_ in defective MoO_3_ facilitated the adsorption and activation of these substrate molecules to the surface Mo^5+^ atoms near the O_v_ sites. Next, the reductive coupling of nitro compounds and alcohols was investigated. Since both methanol and nitromethane can be used as substrates for the reductive coupling reaction, they were chosen as the model substrates in the DFT study.

As shown in Fig. 6C, the β-MoO_3_-O_v_ (200) crystal plane with O_v_ (hereinafter referred to as β-MoO_3_-O_v_) showed a much higher ability to adsorb methanol than the β-MoO_3_ (200) crystal plane (hereinafter referred to as β-MoO_3_), where the former has a much larger adsorption energy (−1.35 eV vs. −1.07 eV). As our experiments confirmed that Mo/NC-500 was able to catalyze the direct dehydrogenation of alcohols, DFT calculation was then used to study the dehydrogenation of methanol. As shown in Fig. S24, the dissociation of H atoms from the –O–H and α-C_sp3_–H on the surface of β-MoO_3_-O_v_ was both much easier than those on the surface of β-MoO_3_ (0.18 eV vs. 0.53 eV for –O–H bonds and 1.67 eV vs. 2.64 eV for α-C_sp3_–H). The Bader charges of Mo^5+^ atoms near the O_v_ sites before and after methanol adsorption on the surface of β-MoO_3_-O_v_ were calculated to be 3.92 and 3.84 e^−^ (Fig. 6A vs. Fig. S25), which revealed that adsorption of methanol resulted in the transfer of ~0.08 e^−^ from Mo^5+^ atoms near the O_v_ sites to the CH_3_O^−^ group, serving a role in the activation of the CH_3_O^−^ group. However, that was not the case for the adsorption of methanol on the β-MoO_3_ plane (Fig. 6A vs. Fig. S25). DFT calculation confirmed that the presence of O_v_ in β-MoO_3_ greatly lowered the energy barriers of the dissociated H atoms from alcohols, and the cleavage of the α-C_sp3_–H bond was much more difficult than that of the –O–H bond, in good consistency with our experimental results.

Interestingly, the adsorption energies of methanol were both increased on the surfaces of β-MoO_3_ (1.07 eV vs. 1.73 eV) and β-MoO_3_-O_v_ (1.35 eV vs. 2.80 eV) in the presence of nitromethane (O=N^+^–O^−^), indicating that the presence of O=N^+^–O^−^ group benefited methanol adsorption (Fig. 6C). In particular, the positive H atom from –OH in methanol can spontaneously dissociate to the surface of β-MoO_3_-O_v_ due to the presence of O=N^+^–O^−^, where H^+^ combines with the oxygen atoms in MoO_3_ to form –O–H^*δ*+^ and CH_3_O^−^ adsorbs on the O_v_ sites (Mo^5+^ atoms). Then, the transfer of the negative H atom from the α-C_sp3_–H in CH_3_O^−^ to the oxygen atom in the O=N^+^–O^−^ group was considered, and the energy barriers on the β-MoO_3_-O_v_ and β-MoO_3_ surfaces were calculated to be 1.45 and 1.72 eV, respectively (Fig. 6D). After the adsorption of the methanol molecule on the β-MoO_3_-O_v_ surface, the nitromethane molecule was then adsorbed. It was noted that the Bader charges of Mo^5+^ atoms near the O_v_ sites further decreased (Fig. S26). Meanwhile, the Bader charges of the “O” atom in O=N^+^–O^−^ were calculated to be 6.46 and 6.51 e^−^ in the free nitromethane molecule and the activated nitromethane molecule, respectively. After the transfer of the H atom from α-C_sp3_–H in CH_3_O^−^ to the “O” atom in O=N^+^–O^−^, the Bader charge of the “O” atom in O=N^+^–O^−^ continued to increase to 6.65 e^−^ (Fig. S26), and that of Mo^5+^ atoms fell back to 3.91 e^−^, suggesting that the interaction between O=N^+^–O^−^ and O_v_ resulted in electron transfer from β-MoO_3_ to O=N^+^–O^−^ via Mo^5+^ atoms. In addition, we noted that the density of the Bader charges in the adsorbed CH_3_O^−^ group decreased after the adsorption of the nitromethane molecule (Fig. S25B), which means that the electron should transfer from methanol to the nitro group via Mo^5+^ atoms.

According to these results, the essence of the transfer hydrogenation can be described as a proton-coupled electron transfer process: the nitro group receives electrons from Mo^5+^ and H from α-C_sp3_–H in the adsorbed CH_3_O^−^ group. During this process, the transfer of electrons from Mo^5+^ to the nitro group results in the valence change of Mo^5+^ to Mo^6+^. After the release of the H atom from α-C_sp3_–H in the CH_3_O^−^ group, the electron would transfer to Mo^6+^ to regenerate Mo^5+^ active sites with the formation of one formaldehyde molecule. In short, the Mo^5+^/Mo^6+^ redox cycle over the β-MoO_3_-O_v_ surface accompanied the transfer hydrogenation process: “CH_3_O^−^ → CH_2_O + H^+^ + e^−^” and “O=N^+^–O^−^ + H^+^ + e^−^ → HO–N=O”. The facile electron transfer via the Mo^5+^ atoms near O_v_ should be the reason for the lower energy barrier of these transfer reductive coupling reactions over the defective MoO_3_ species with O_v_.

**Fig. 7.**
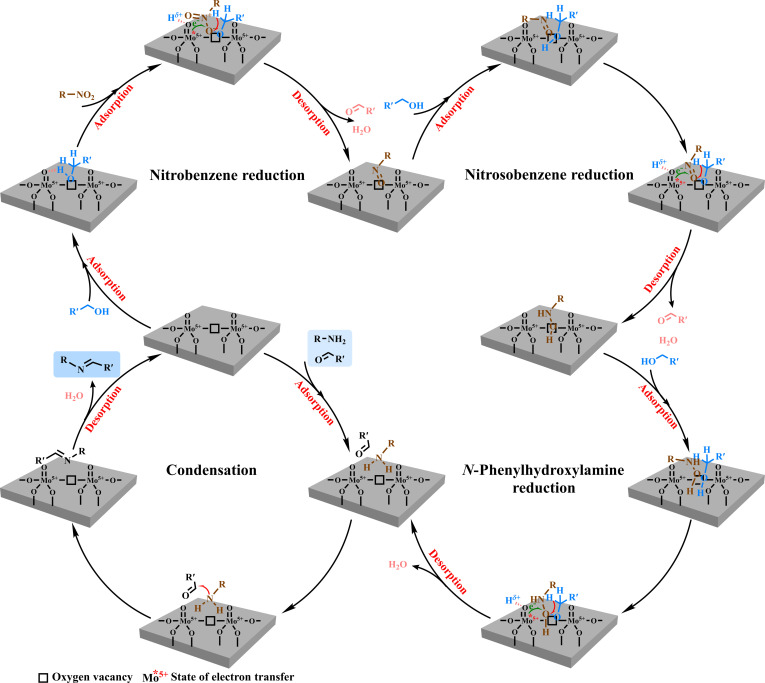
Proposed reaction mechanism for the MoO_3_-catalyzed reductive coupling of nitro compounds with alcohols via oxygen-vacancy-mediated pathways.

On the basis of the theoretical and experimental results together with the previous reports [11,16,20,65,66], a plausible mechanism was proposed for the dehydrogenative coupling of alcohols and nitro compounds (Fig. 7). Briefly, alcohols and nitro compounds initially adsorb on oxygen-defective sites in MoO_3_ (also combining with the Mo^5+^ atoms). Then, the positive H atom from –O–H can spontaneously transfer to the oxygen atom (base sites) in MoO_3_. After the formation of an active alkoxyl group on the oxygen-defective sites, the negative H atom from α-C_sp3_–H and the positive H^+^ from the base sites transfer to the positive “N” and negative “O” atoms of O=N^+^–O^−^ groups in nitro compounds, then followed by the release of one water molecule to generate an intermediate with –N=O groups. Meanwhile, one aldehyde molecule is formed and desorbs from the catalyst surface. Then, a similar transfer hydrogenation process proceeds to reduce the –NO groups into –NHOH groups by alcohols. Finally, 2 hydrogen atoms are transferred from the alcohol to the –NHOH intermediate, accompanied by the elimination of a water molecule, thereby generating the corresponding –NH_2_ group. Moreover, in situ condensation of the primary amine with the aldehyde affords the imine product bearing a C=N– bond. Notably, further transfer hydrogenation of the C=N– moiety is effectively suppressed, likely due to the inability of the resulting imine to form stable anionic adsorption complexes at the oxygen vacancy sites on the catalyst surface.

## Conclusion

In summary, nitrogen-doped carbon-supported molybdenum oxides enriched with oxygen vacancies (O_v_) were successfully synthesized through the pyrolytic treatment of a composite comprising molybdenum salts and a nitrogen-containing carbon precursor. The as-prepared Mo/NC-500 catalyst exhibited outstanding catalytic activity and selectivity for the synthesis of imines and nitrogen-containing heterocycles via the reductive coupling of biomass-derived alcohols with nitro compounds. This system showed excellent functional group tolerance, including tolerance to fragile groups. Notably, the method was scalable to gram-scale synthesis (up to 7.0 g), highlighting its potential for practical applications in fine chemical manufacturing. Experimental results and DFT calculations revealed that the O_v_ in molybdenum oxides selectively adsorbs and activates nitro compounds and alcohols while leaving C=N bonds inert, playing a decisive role in these reductive coupling reactions toward the synthesis of organonitrogen chemicals with C=N bonds. DFT calculations also revealed that the introduction of O_v_ in β-MoO_3_ significantly lowers the energy barriers associated with hydrogen atom transfer from alcohols to nitro groups. The Mo^5+^/Mo^6+^ redox cycle is proposed to facilitate electron transfer, thereby mediating the overall transformation. This study provides a good case for the design of transition metal oxides with adjustable properties for some challenging organic transformations.

## Materials and Methods

### Materials

Urea, chitosan, MoO_3_, MoO_2_, Mo_2_C, Ru/C (5 wt.%), Pd/C (5 wt.%), and Pt/C (5 wt.%) were purchased from Aladdin Chemicals Co. Ltd. (Shanghai, China). All nitro compounds and alcohols were obtained from commercial sources and verified for purity prior to use. (NH_4_)_6_Mo_7_O_24_·4H_2_O, acetic acid, and all solvents were purchased from Sinopharm Chemical Reagent Co., Ltd. (Beijing, China). Unless specified, all reagents were of analytical grade and used without further purification.

### Catalyst preparation

Typically, urea (12.00 g) and (NH_4_)_6_Mo_7_O_24_·4H_2_O (0.25 g) were dissolved in 10 ml of distilled water under stirring. Then, chitosan (1.00 g) was gradually introduced into the solution under vigorous agitation. Once fully dispersed, acetic acid (1.0 ml) was rapidly added, and the mixture was stirred for an additional 30 min to yield a uniform, semitransparent paste. This mixture was then maintained at 70 °C until a white solid Mo-based precursor was obtained. The dried precursor was transferred to an alumina boat and subjected to pyrolysis in a quartz tube furnace under a nitrogen atmosphere. The temperature was ramped from ambient to the target temperature (500 to 800 °C) at a rate of 2 °C/min and held for 2 h, resulting in a black Mo-based catalyst powder. The obtained material was ground into a fine powder and designated as Mo/NC-T, where T indicates the pyrolysis temperature (°C). For comparison, a series of reference catalysts, including Mo/C-500, Mo/C-600, Mo/C-700, Mo/C-800, and NC-500, were prepared using the same protocol, excluding the addition of urea and/or (NH_4_)_6_Mo_7_O_24_·4H_2_O, respectively.

### Catalyst characterization

TEM was performed using a Talos F200X microscope operating at 200 kV. XRD analysis was carried out on a Bruker D8 Advance diffractometer employing Cu Kα radiation, with diffraction patterns collected in the 2*θ* range of 10° to 80° at a scan rate of 0.016°/s. The surface area and pore size distribution were determined by nitrogen adsorption–desorption isotherms at −196.15 °C using a V-Sorb 2800P analyzer, following degassing at 150 °C for 12 h. XPS was conducted on a Thermo VG Scientific ESCA MultiLab-2000 system equipped with a monochromatic Al Kα source (1,486.6 eV), with binding energies calibrated to the C 1s peak at 284.6 eV. The Mo content was quantified via inductively coupled plasma atomic emission spectroscopy on a Thermo IRIS Intrepid II XSP instrument. Raman spectroscopy was performed on a Thermo Fisher DXR confocal micro-Raman system with a 532-nm diode laser. EPR measurements were conducted using a Bruker EMXnano spectrometer. X-ray absorption spectra were collected on beamline BL07A1 in the National Synchrotron Radiation Research Center. A Si (111) double-crystal monochromator was used for energy selection. Data processing for XANES and EXAFS was carried out using the Demeter software suite: Athena (v0.9.25) was employed for background subtraction and normalization, and Artemis (v0.9.25) was used for Fourier-transform fitting [67]. The fitting was performed with *k*^3^ weighting over the *R* range of 1 to 2.2 Å, based on standard models of bulk Mo, MoO_2_, and MoO_3_. CN, bond length (*R*), Debye–Waller factor (*σ*^2^), and energy shift (Δ*E*_0_) were treated as independent fitting parameters without constraints or correlations.

### Catalyst evaluation

In a typical reaction, nitrobenzene (1.0 mmol), Mo/NC-500 (20 mg), and ethanol (10 ml) were introduced into a 50-ml stainless-steel autoclave equipped with magnetic stirring, temperature control, and pressure regulation. The reactor was purged with nitrogen 5 times to eliminate residual air and subsequently pressurized with N_2_ to 1 MPa. The mixture was then heated to 180 °C and stirred for 5 h. Upon completion, the autoclave was cooled to room temperature, and the catalyst was separated by centrifugation. The resulting supernatant was analyzed by GC and gas chromatography–mass spectrometry (GC–MS). For reuse studies, the Mo/NC-500 catalyst was recovered by centrifugation, thoroughly washed with ethanol, and directly reused in subsequent runs without further treatment to minimize catalyst loss. The synthesis of benzazoles (including benzimidazoles, benzoxazoles, and benzothiazoles) and quinoxalines was performed following the same protocol as for imine formation, with appropriate nitro and alcohol substrates substituted accordingly.

For product purification, the crude reaction mixture was concentrated under reduced pressure and subjected to silica gel column chromatography using suitable eluents. The imine products were purified by flash column chromatography on a silica gel using hexane dichloromethane and triethyl amine (*v*_hexane_/*v*_dichloromethane_/*v*_triethyl amine_ = 100/10/5) as the eluent. The silica gel was washed with triethylamine before the purification to avoid imine hydrolysis during the purification process. Ethyl acetate and hexane (*v*_ethyl acetate_/*v*_hexane_ = 70/30) were used as eluent for the purification of *N*-hetero compounds, including benzimidazoles, benzoxazoles, benzothioazoles and quinoxalines. The molecular structures of the obtained products were confirmed by GC–MS and further characterized by ^1^H and ^13^C nuclear magnetic resonance spectroscopy.

### Analytical methods

Product analysis was carried out using an Agilent 7890A gas chromatograph equipped with a flame ionization detector and a cross-linked HP-5 capillary column (30 m × 0.32 mm × 0.4 μm). Nitrogen served as the carrier gas at a flow rate of 40 ml/min. The injection and detector temperatures were both maintained at 300 °C. The oven temperature program was set as follows: initial temperature of 50 °C (held for 1.5 min), ramped to 300 °C at 15 °C/min and held for 3 min. The molecular mass and structural information of the products were confirmed using GC–MS on a Thermo Scientific ISQ 7000 Single Quadrupole system. Quantitative analysis was performed using the internal standard method. Structural elucidation of the compounds was further supported by nuclear magnetic resonance spectroscopy, with spectra recorded on a Bruker TCI III 600-MHz spectrometer.

### Computational details

Structural and charge density calculations were performed using the Vienna Ab initio Simulation Package [68,69], within the framework of spin-polarized DFT. The interactions between ions and electrons were described using the generalized gradient approximation with the Perdew–Burke–Ernzerhof exchange-correlation functional [70]. A plane-wave cutoff energy of 500 eV was employed, and Brillouin zone sampling was conducted using a 3 × 3 × 1 Monkhorst–Pack *k*-point mesh. The convergence criteria were set to 1 × 10^−4^ eV for total energy and 0.01 eV/Å for atomic forces. The catalyst model was constructed based on the β-MoO_3_ phase, with lattice parameters *a* = *b* = *c* = 3.93 Å. Informed by XRD and TEM results (Fig. 2C and Fig. S2), a 2 × 2 supercell of the MoO_3_ (100) surface was selected as the computational slab. The slab consisted of 6 atomic layers, with the bottom 2 layers fixed to simulate bulk constraints. A vacuum layer exceeding 15 Å was introduced along the *z* axis to eliminate spurious interactions between periodic images. Charge density difference analyses were conducted using the VESTA visualization package [71].

The adsorption energies (*E*_ad_) of CH_3_OH followed the equation *E*_ad_ = *E*_total_ − (*E*_catal_ + *E*_mol_). *E*_total_, *E*_catal_, and *E*_mol_ represent the total energy of CH_3_OH adsorption on the catalyst, pristine catalyst, and CH_3_OH, respectively.

## Data Availability

All data required to evaluate the conclusion of the paper are presented in this paper and the Supplementary Materials. Additional data related to this article can be reasonably obtained from the authors.

## Supplementary Materials

Supplementary 1 Figs. S1 to S26 Tables S1 to S11 Supplementary Data Supplementary References
