# Supplementary material for: Oxygen Vacancies in β-MoO3 Mediate Imine Synthesis via Reductive Coupling of Nitro Compounds and Alcohols
Source: Research (Wash D C). 2025 Dec 9;8:0993. doi: 10.34133/research.0993 (PMC12686347; doi:10.34133/research.0993)
Supplement: Supplementary 1 — Figs. S1 to S26 Tables S1 to S11 Supplementary Data Supplementary References [file research.0993.f1.docx]

**Oxygen vacancies in *β*-MoO_3_ mediate imine synthesis *via* reductive coupling of nitro compounds and alcohols**

*Ziliang Yuan**^1,3#^, Yijing Gao^2,4#^, Qingjie Tang^1^, Jianguo Wang^2*^, Xun Li^1^, and Zehui Zhang^1^**

^1^ Key Laboratory of Catalysis and Materials Sciences of the Ministry of Education, South-Central Minzu University, Wuhan, 430074, P. R. China.

^2^ Institute of Industrial Catalysis, State Key Laboratory Breeding Base of Green-Chemical Synthesis Technology, College of Chemical Engineering, Zhejiang University of Technology, Hangzhou 310032, P. R. China.

^3^ Zhejiang Engineering Laboratory for Green Syntheses and Applications of Fluorine-Containing Specialty Chemicals, Institute of Advanced Fluorine-Containing Materials, Zhejiang Normal University, Jinhua 321004, P. R. China.

*^#^* The authors are equally contributed to this work.

**Corresponding authors:** Tel.: +86-27-67842572. Fax: +86-27-67842572. E-mail: jgw@zjut.edu.cn; zehuizh@mail.ustc.edu.cn

**Table of Contents**

[Supplementary Figures S2](#_Toc199093266)

[Supplementary Tables S20](#_Toc199093267)

[Supplementary Data S29](#_Toc199093268)

[Supplementary References S134](#_Toc199093269)

# Supplementary Figures

**Fig. S1.** (a) Nitrogen adsorption-desorption isotherms, and (b) the pore size distribution of the Mo/NC-T catalysts.

**Fig. S2.** XRD patterns of the Mo/NC-T (a) and Mo/C-T (b) catalysts.


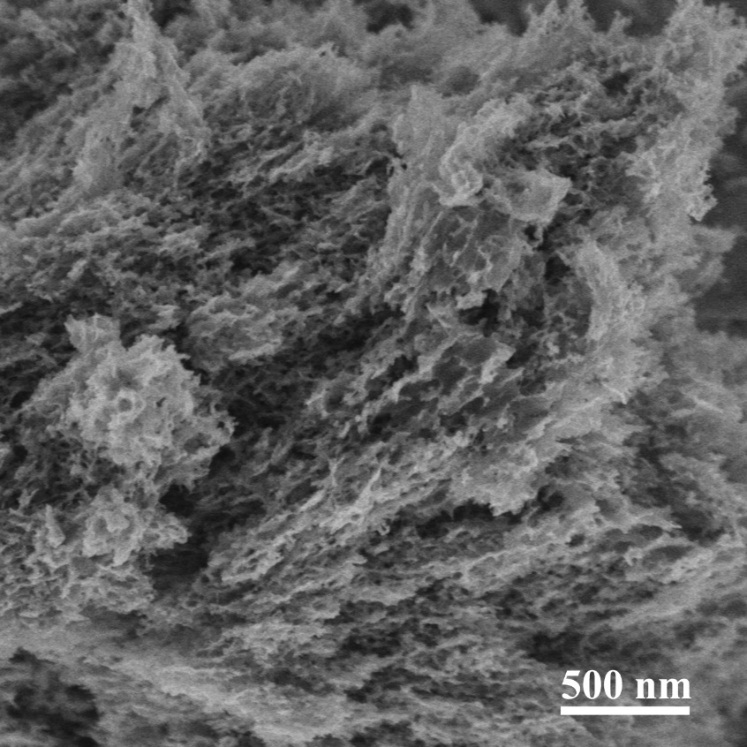


**Fig. S3.** SEM image of the as-prepared Mo/NC-500 catalyst.


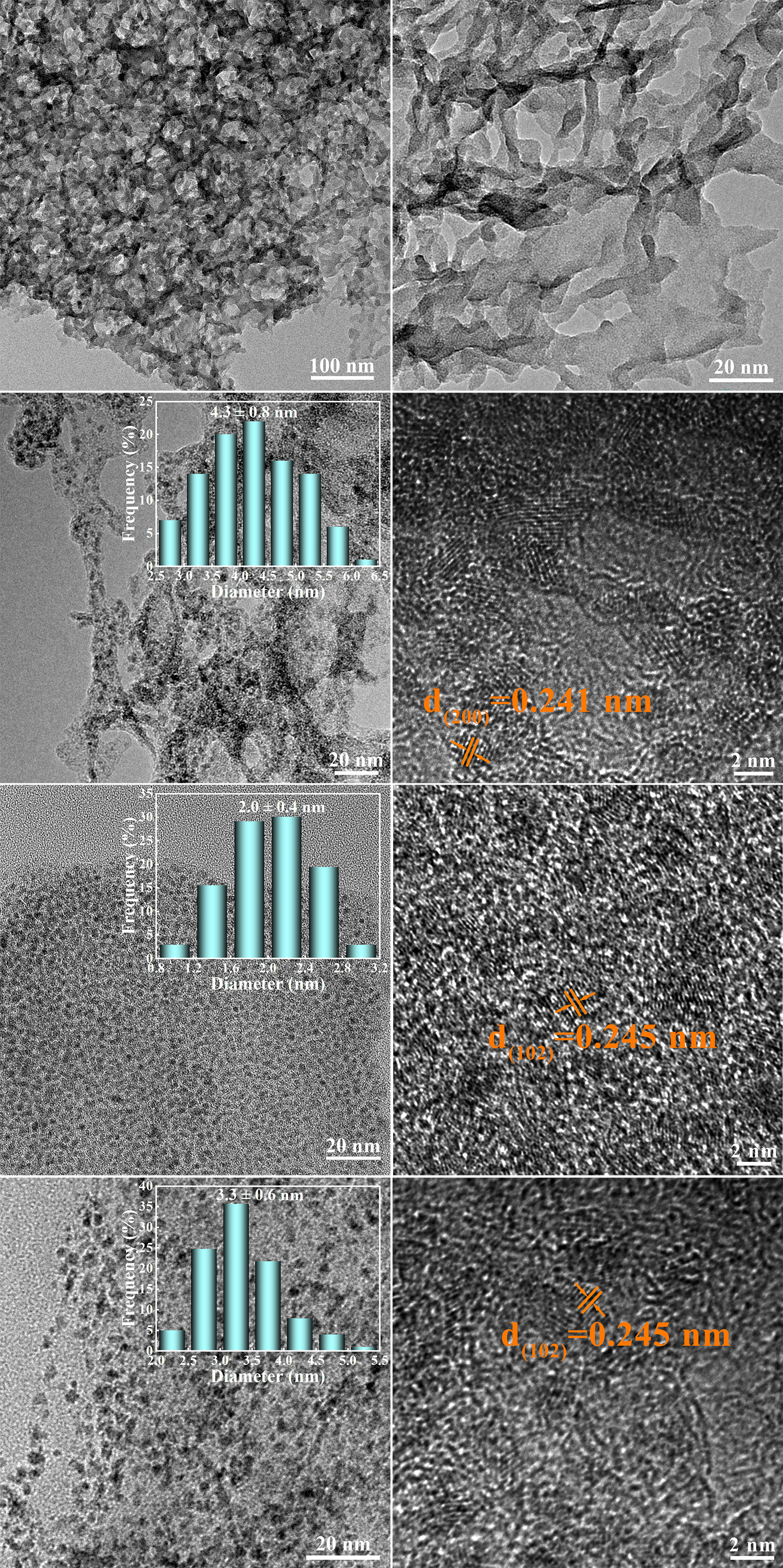


**( g ) ( h )**

**( e ) ( f )**

**( c ) ( d )**

**( a ) ( b )**

**Fig. S4.** TEM and HR-TEM images of the as-prepared Mo/NC-450 (a, b), Mo/NC-600 (c, d), Mo/NC-700 (e, f), and Mo/NC-800 (g, h) catalysts.


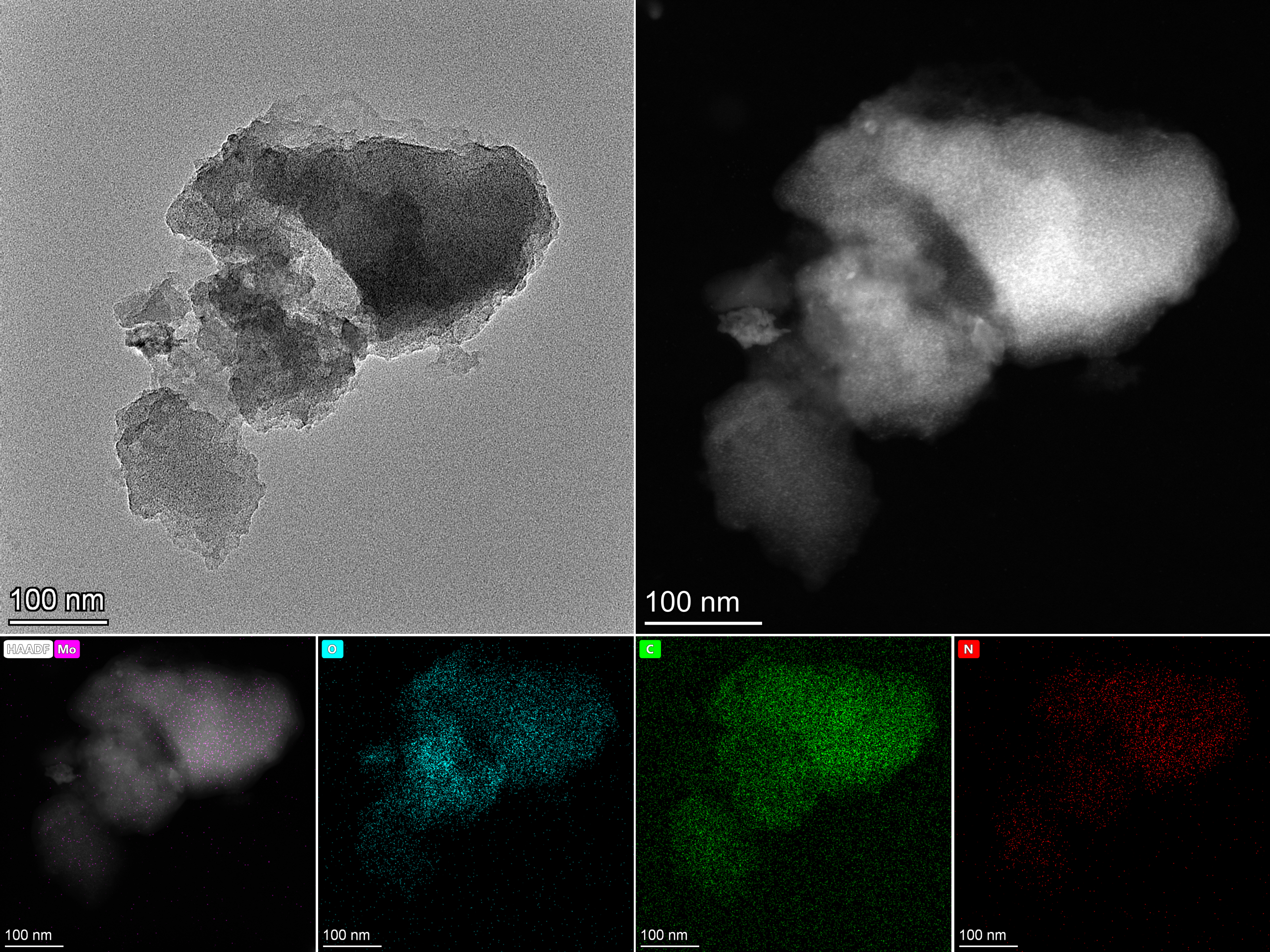


**( c )**

**( a ) ( b )**

**Fig. S5.** The TEM images (a), HAADF-STEM (b), and EDS elemental mapping images (c) of Mo/NC-500.


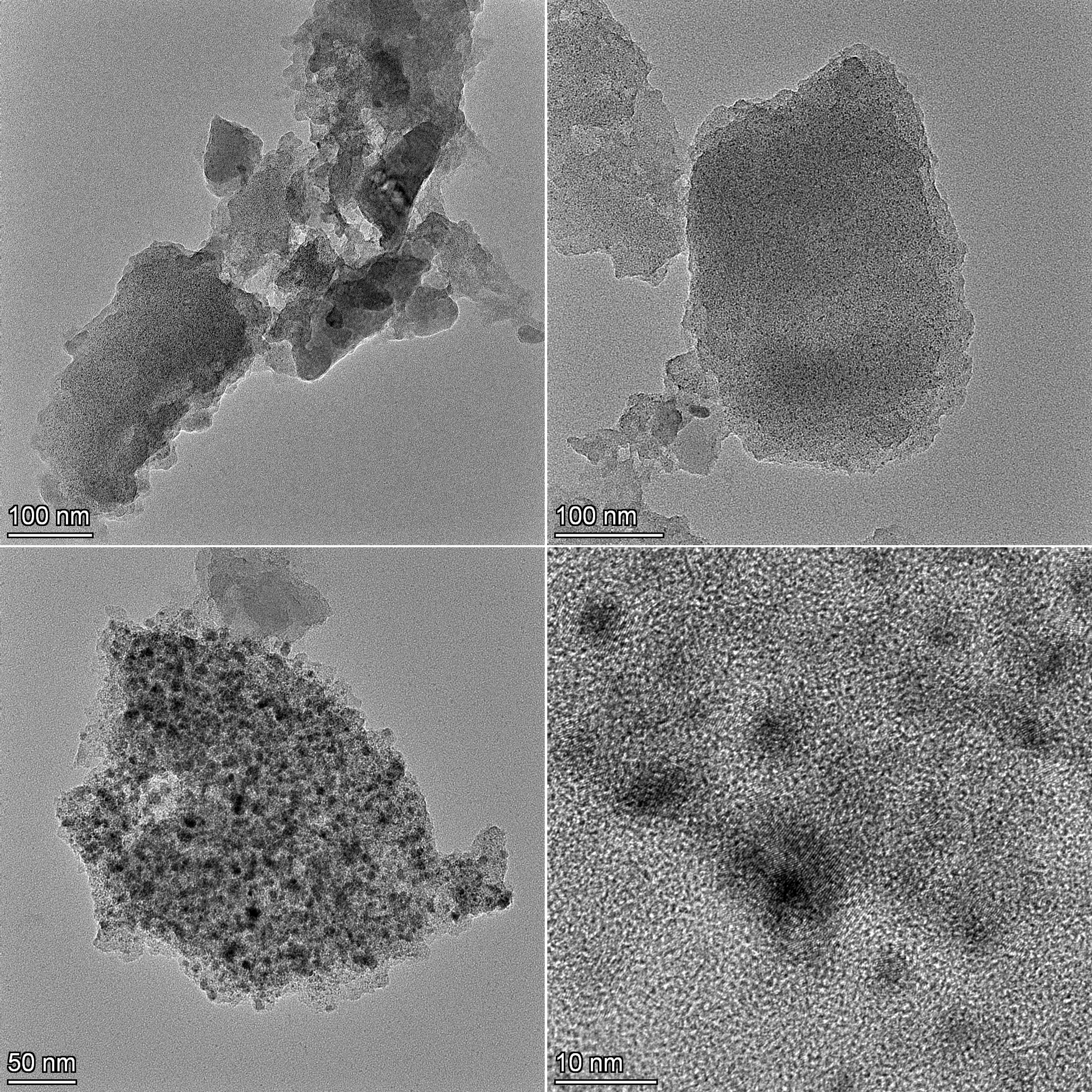


**( c ) ( d )**

**( a ) ( b )**

**Fig. S6.** TEM images of the as-prepared Mo/C-600 (a), Mo/C-700 (b) catalysts and the TEM images (c), HR-TEM image (d) of the Mo/C-800 catalyst.

**Fig. S7.** Raman spectra of the Mo/NC-T catalysts and the zoom in range of 200-1000 cm^-1^.

**Fig. S8.** Full XPS survey spectrum of the representative Mo/NC-500.

**Fig. S9.** Mo 3d XPS spectra of the Mo/NC-T and Mo/C-500 catalysts.

**Fig. S10.** N 1s XPS spectra of the Mo/NC-T and Mo/C-500 catalysts.

**Fig. S11.** C 1s XPS spectra of the Mo/NC-T and Mo/C-500 catalysts.

**Fig. S12.** O 1s XPS spectra of the Mo/NC-T and Mo/C-500 catalysts.

**Fig. S13.** The hot filtration experiment in the dehydrogenative coupling of nitrobenzene with ethanol over the MoO_3_. Reaction conditions: nitrobenzene (1.0 mmol), MoO_3_ (5.0 mg), C_2_H_5_OH (10 mL), 200 °C, 10 bar N_2_, and the catalyst was filtered out from the reaction mixture after 2 h and 4 h at 200 °C with the remaining filtrate was allowed to react up to the 8 h, respectively.

|  |
| --- |
|  |

**Fig. S14.** Hot filtration experiment in the dehydrogenative coupling of nitrobenzene with ethanol over the Mo/NC-500. Reaction conditions: nitrobenzene (1.0 mmol), Mo/NC-500 (20.0 mg), C_2_H_5_OH (10 mL), 200 °C, 10 bar N_2_, and the Mo/NC-500 catalyst was filtered out from the reaction mixture after 4 h at 200 °C and the remaining filtrate was allowed to react up to the 10 h.

|  |
| --- |
|  |

**Fig. S15.** Reusability of the Mo/NC-500 catalyst towards the synthesis of *N*-phenylethanimine. Reaction conditions: nitrobenzene (1.0 mmol), the Mo/NC-500 catalyst (20 mg), ethanol (10 mL), 200 °C, N_2_ (10 bar) and 4 h.

**Fig. S16.** XRD patterns of the Mo/NC-500 and recycling of Mo/NC-500 catalysts.


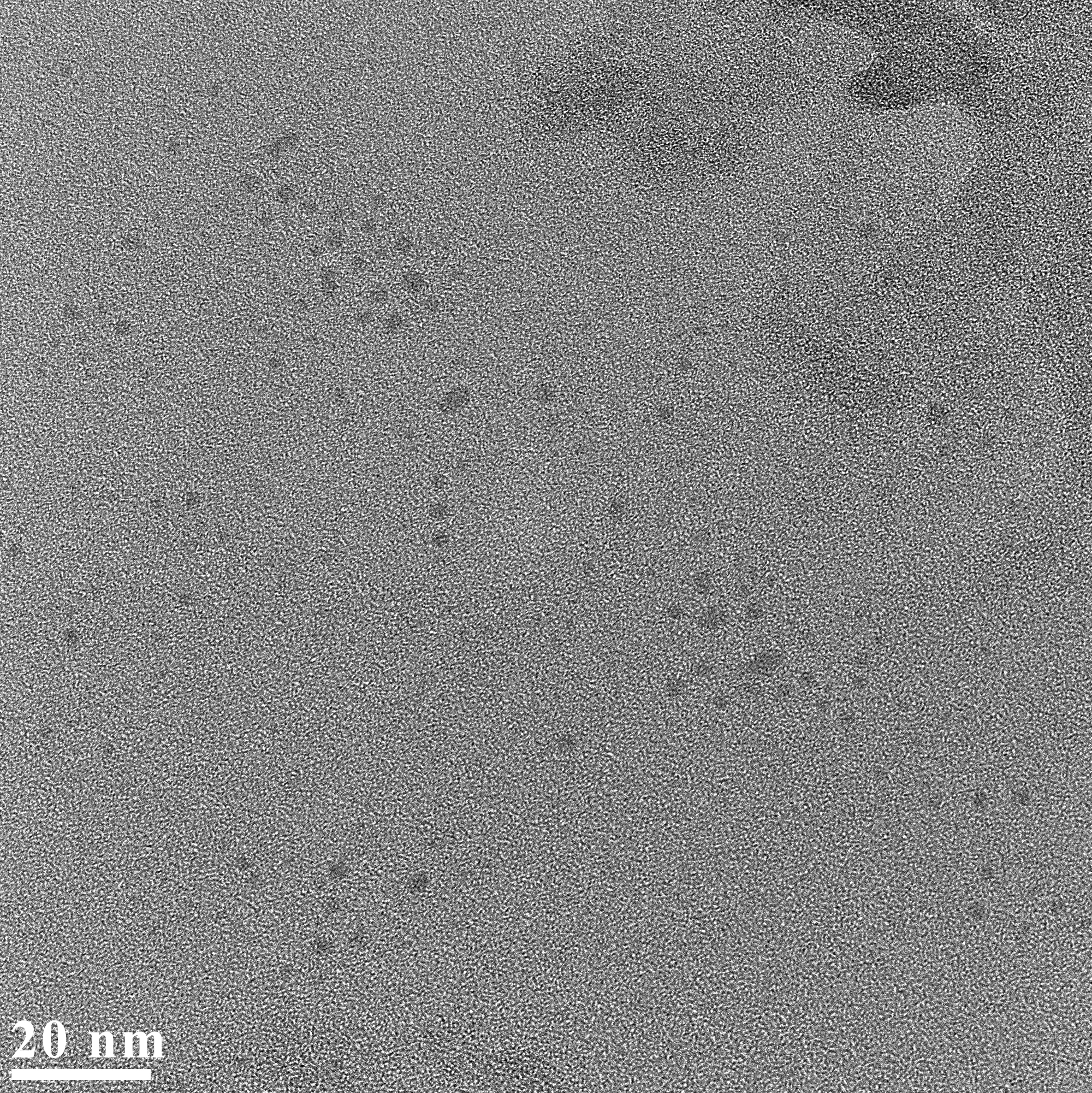

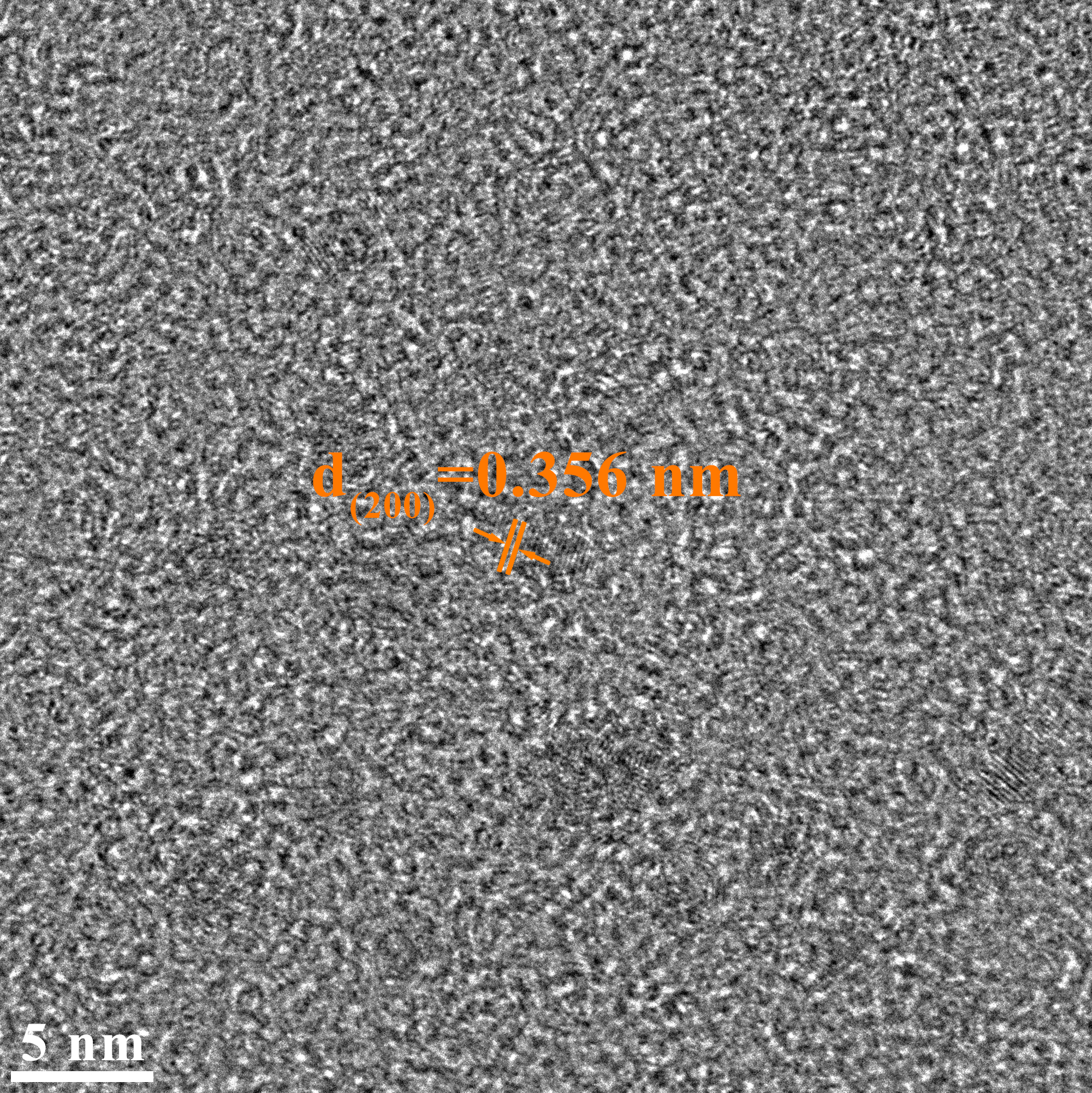


**( a ) ( b )**

**( e ) ( f )**

**( c ) ( d )**

**Fig. S17.** TEM (a), HR-TEM (b) images, XPS spectra (c-f) of the recovered Mo/NC-500 catalysts.

|  |
| --- |
|  |

**Fig. S18.** The effect of the reaction temperature on the synthesis of *N*-phenylethanimine. Reaction conditions: nitrobenzene (1.0 mmol), the Mo/NC-500 catalyst (20 mg), ethanol (10 mL), N_2_ (10 bar) and 5 h.

|  |
| --- |
|  |

**Fig. S19.** Time course of the products distribution of the dehydrogenative coupling of nitrobenzene with ethanol at 200 °C. Reaction conditions: nitrobenzene (1.0 mmol), the Mo/NC-500 catalyst (20 mg), ethanol (10 mL), 10 bar N_2_.

**Fig. S20.** Direct condensation reaction test of aniline and benzaldehyde. Reaction conditions: aniline (1.0 mmol), benzaldehyde (1.0 mmol), Mo/CN-500 (20.0 mg), acetonitrile (10 mL), 30 °C and 10 bar N_2_.

**Fig. S21.** Gram-scale reactions and the synthesis of bioactive molecules. ^a^ Reaction conditions: nitro compounds (1.0 mmol), amines (2.0 mmol), alcohols (3.0 equiv.), acetonitrile (10 mL), Mo/NC-500 (20 mg) and 200 °C; ^b^ Nitro compounds (10.0 mmol), Mo/NC-500 (100 mg), ethanol (40 mL), and 200 °C; ^c^ Nitro compounds (10.0 mmol): amines: alcohols = 1: 2 : 3, acetonitrile (40 mL), Mo/NC-500 (100 mg) and 200 °C; ^d^ Same as “c”, but using nitro compounds (5.0 mmol), and alcohols (5.0 equiv.); ^e^ Step 1: same as “c”; Step 2: 2-(4-methoxyphenyl) benzimidazole (3.0 mmol), *p*-anisaldehyde (5.0 mmol), Co@CN-800 (100 mg), THF (40 mL), formic acid (5 mmol) and 170 °C.


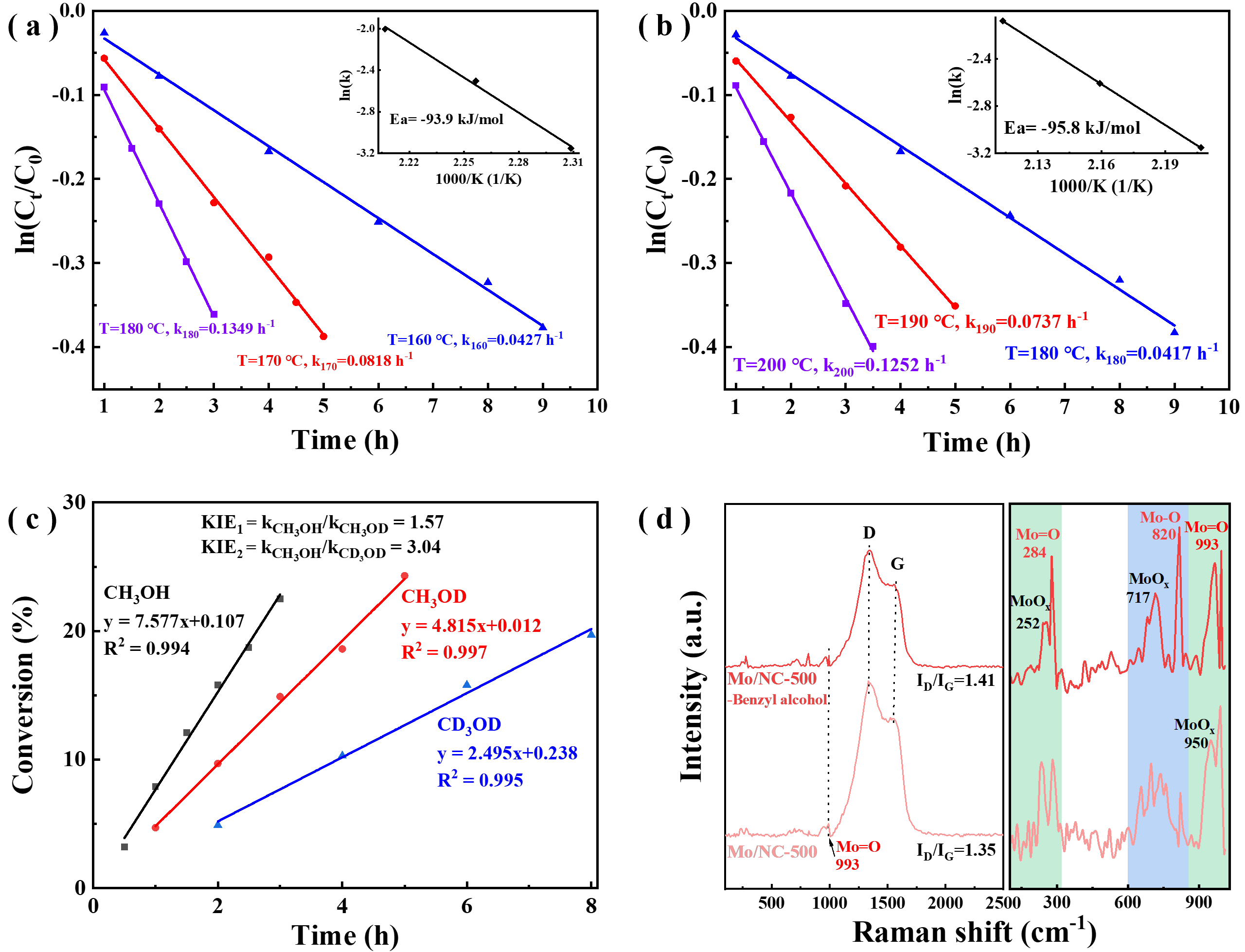


**Fig. S22.** Kinetic studies of the coupling of benzyl alcohol with nitrobenzene (a) or aniline (b); Reaction conditions: nitrobenzene or aniline (10.0 mmol), benzyl alcohol (1.0 mmol), Mo/NC-500 (20.0 mg), and acetonitrile (10 mL). Reaction conditions for the isotopic labeling experiments (c): nitrobenzene (1 mmol), Mo/NC-500 (20.0 mg), solvent (CH_3_OH or CH_3_OD or CD_3_OD, 10 mL), and 200 °C. Raman spectra of the fresh Mo/NC-500 catalyst and the treated catalyst with benzyl alcohol (d).


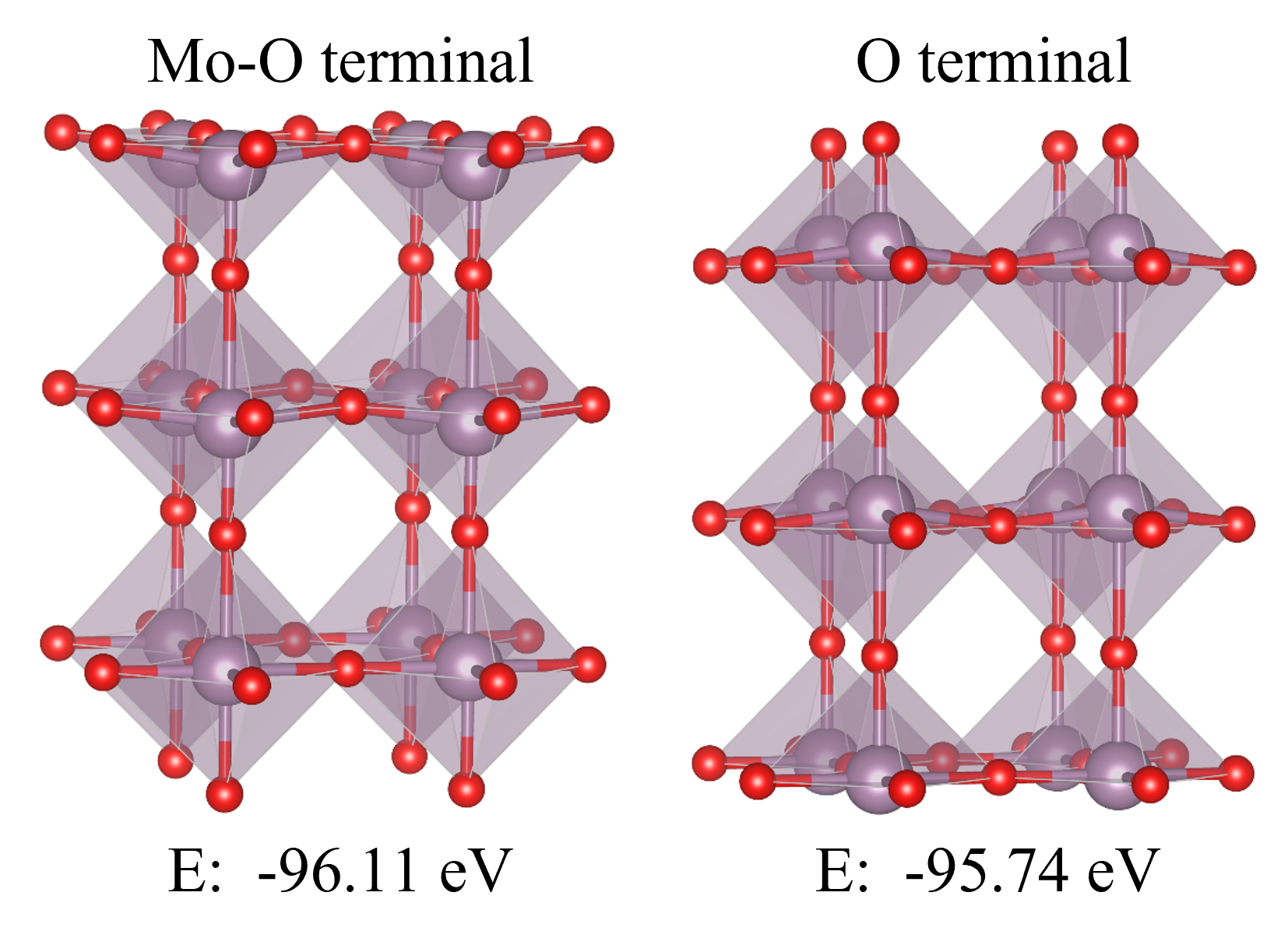


**Fig. S23.** Optimal structures and energies of *β*-MoO_3_ (200) surface with Mo-O terminal and O terminal.


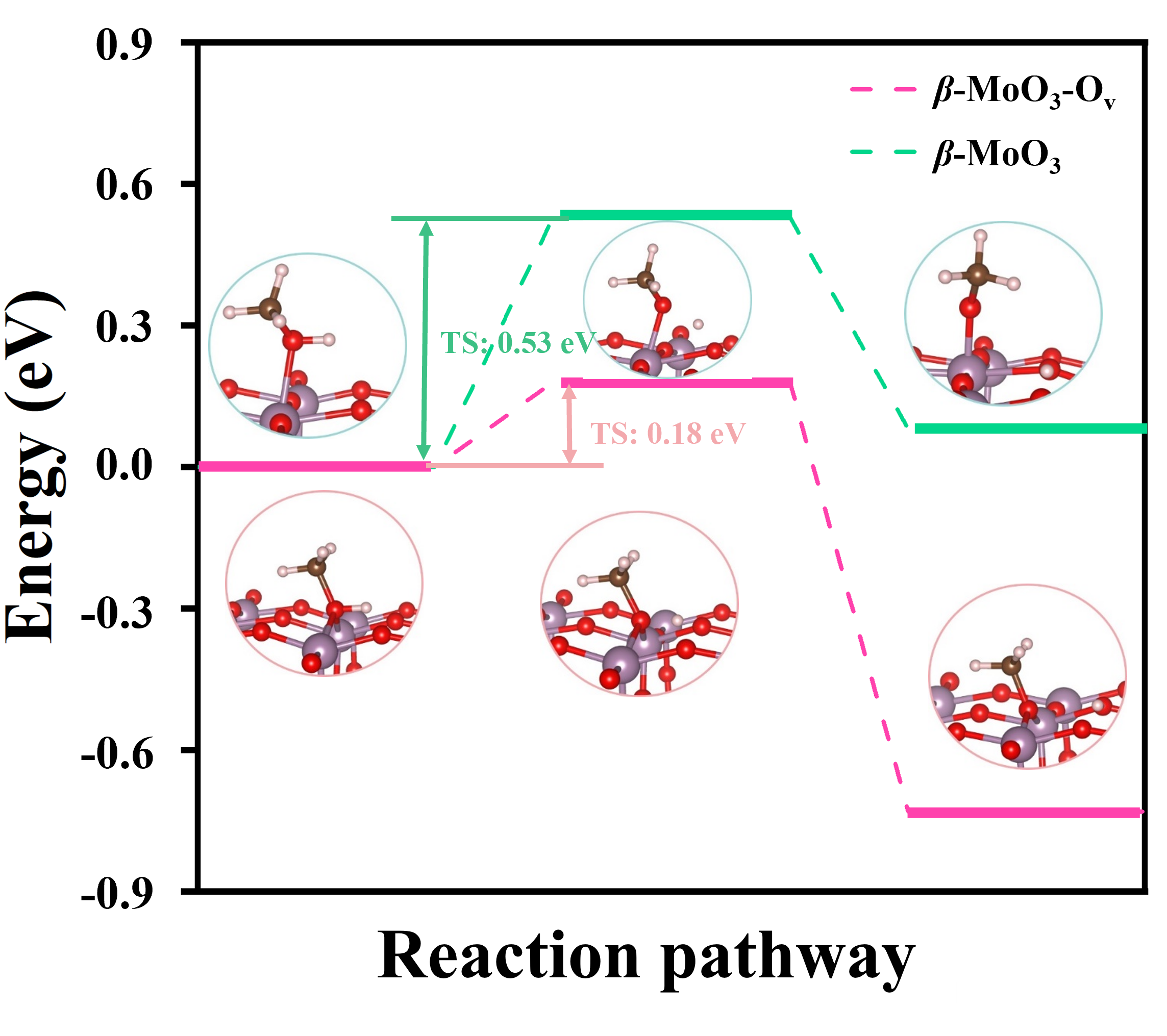

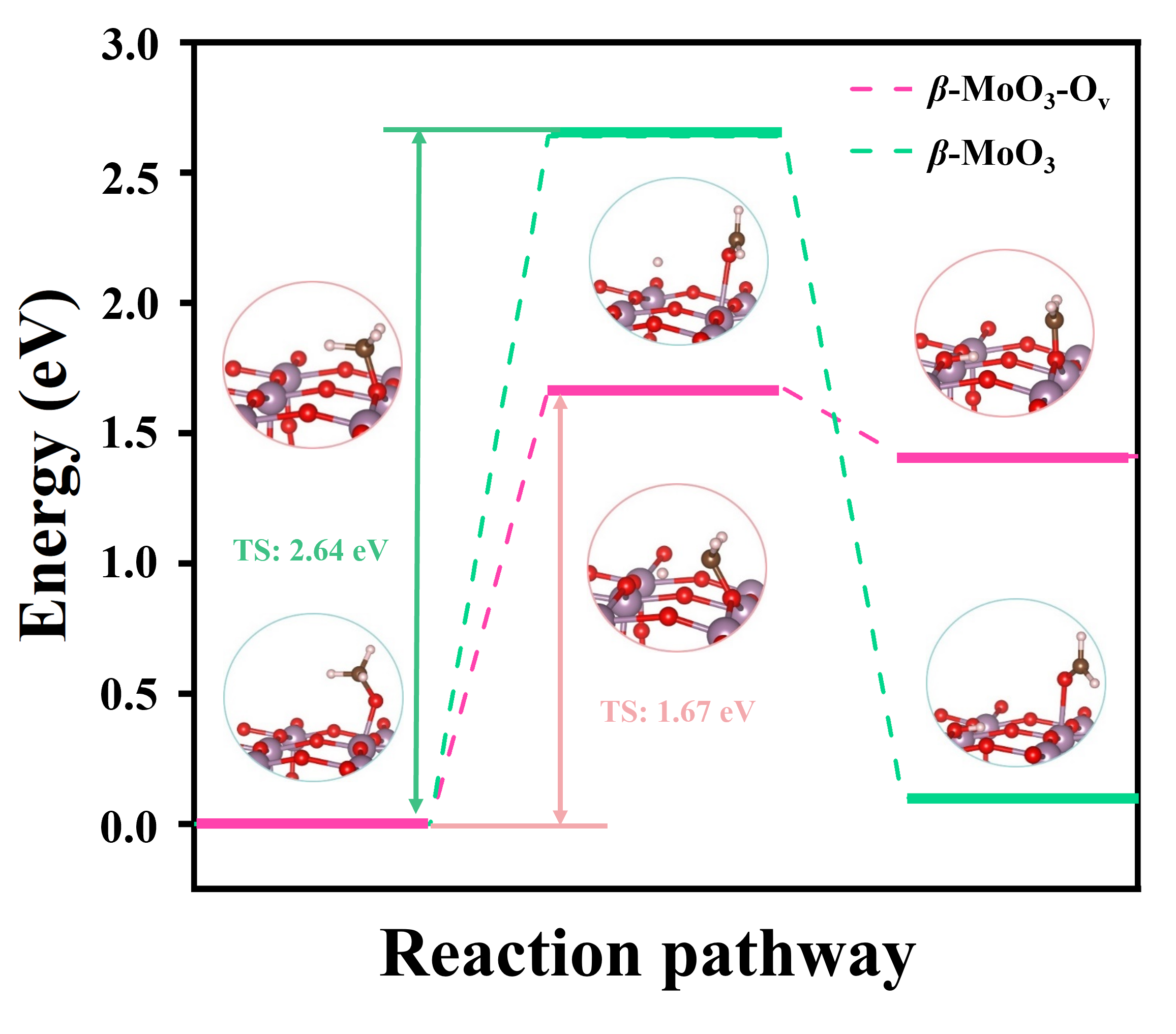


**( a ) ( b )**

**Fig. S24.** Potential energy profiles of dissociation of -O-H (a) and α-C_sp3_-H (b) from methanol on the surfaces of *β*-MoO_3_ and *β*-MoO_3_-O_v_. (Legend: red, O atoms; purple, Mo atoms; pink, H atoms; and brown, C atoms, respectively.)


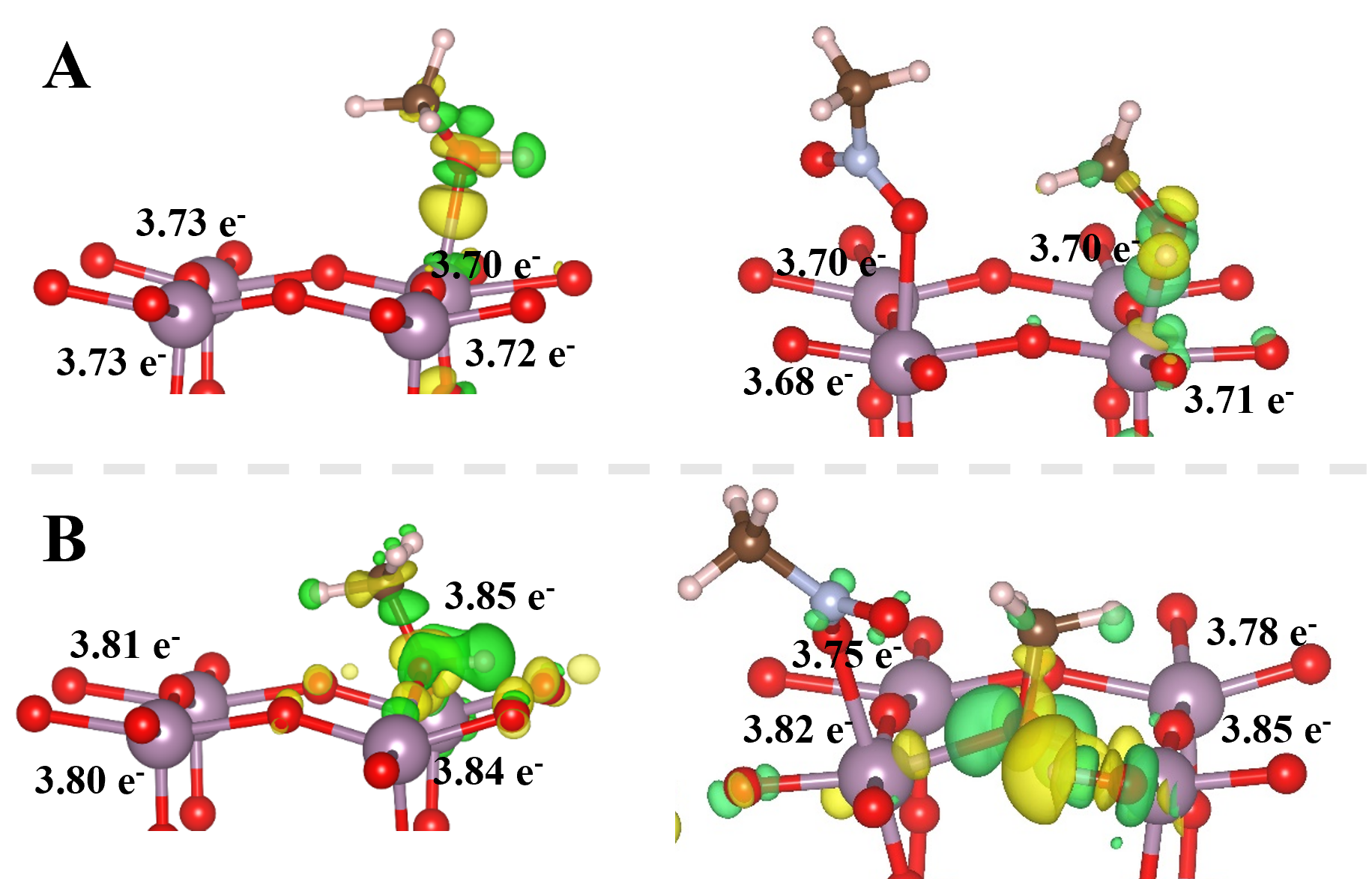


**Fig. S25.** The Bader charge distribution of methanol and nitromethane after adsorption on *β*-MoO_3_ (A) and *β*-MoO_3_-O_v_ (B).


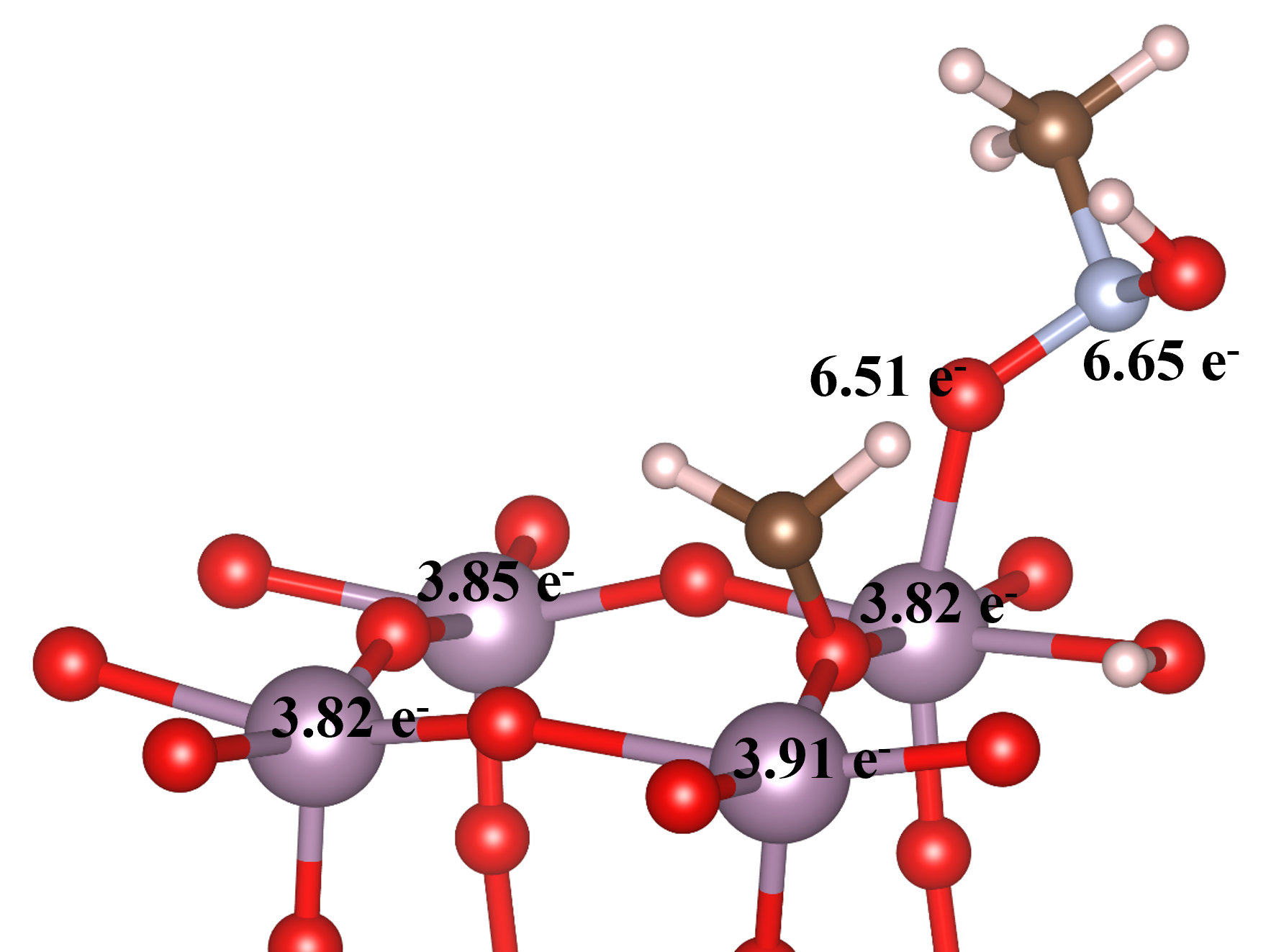


**Fig. S26.** The Bader charge distribution of the "O" atom in O=N^+^-O^-^ after the transfer of the H atom from α-C_sp3_-H in CH_3_O^-^ to the “O” atom in O=N^+^-O^-^ on *β*-MoO_3_-O_v_ surface.

# Supplementary Tables

**Table S1.** Mo content and porosity of the Mo/NC-T catalysts.

| **Catalyst** | **Mo** ^a^  **(wt.%)** | **Surface area**  **(m^2^/g)** | | **Pore size**  **(nm)** | | **Pore volume**  **(cm^3^/g)** | |
| --- | --- | --- | --- | --- | --- | --- | --- |
|  |  | **Meso** | **Micro** | **Meso** | **Micro** | **Meso** | **Micro** |
| Mo/NC-450 | 8.6 | 61.5 | 37.1 | 27.7 | 0.91 | 0.27 | 0.014 |
| Mo/NC-500 | 10.3 | 55.7 | 37.0 | 27.3 | 0.94 | 0.25 | 0.014 |
| Mo/NC-600 | 16.4 | 42.2 | 27.3 | 24.0 | 1.24 | 0.18 | 0.010 |
| Mo/NC-800 | 19.6 | 39.3 | 24.6 | 23.2 | 1.27 | 0.16 | 0.008 |

^a^ Determined by ICP-AES

**Table S2.** The binding energies of the different valence states of Mo in the catalysts.

| **Catalyst** | **Mo^6+^ (eV)** | | **Mo^5+^ (eV)** | | **Mo^4+^ (eV)** | | **Mo^2+^ (eV)** | |
| --- | --- | --- | --- | --- | --- | --- | --- | --- |
| Mo/NC-450 | 235.5 | 232.3 | 233.8 | 230.6 | 232.9 | 229.7 | - | - |
| Mo/C-500 | 235.8 | 232.7 | 234.1 | 231.0 | - | - | - | - |
| Mo/NC-500 | 235.5 | 232.4 | 233.8 | 230.7 | 232.9 | 229.7 | - | - |
| Mo/NC-500-Benzyl alcohol ^a^ | 235.7 | 232.6 | 234.1 | 231.0 | 233.1 | 229.8 | - | - |
| Mo/NC-600 | 235.6 | 232.4 | 234.0 | 230.8 | 232.9 | 229.7 | 231.8 | 228.6 |
| Mo/NC-800 | 235.7 | 232.6 | 234.0 | 230.8 | 232.8 | 229.6 | 231.9 | 228.6 |
| Mo/NC-500  Recycling of catalyst | 235.5 | 232.4 | 233.8 | 230.7 | 232.9 | 229.7 |  |  |

^a^ Mo/NC-500 treated with benzyl alcohol.

**Table S3.** The relative peak area of different valence states of Mo in the catalysts and the calculated average valence state of Mo in the catalysts.

| **Catalyst** | **Mo atomic percentage ^a^ (at.%)** | **Peak area percentage of Mo^6+^ (%)** | **Peak area percentage of Mo^5+^ (%)** | **Peak area percentage of Mo^4+^ (%)** | **Peak area percentage of Mo^2+^ (%)** | **Average valence of Mo** |
| --- | --- | --- | --- | --- | --- | --- |
| Mo/NC-450 | 1.8 | 53.7 | 29.9 | 16.4 | - | 5.39 |
| Mo/C-500 | 3.2 | 96.2 | 3.8 | - | - | 5.96 |
| Mo/NC-500 | 2.0 | 65.8 | 21.1 | 13.1 | - | 5.53 |
| Mo/NC-500- Benzyl alcohol ^b^ | 2.0 | 73.7 | 15.1 | 11.2 | - | 5.63 |
| Mo/NC-600 | 3.6 | 11.2 | 19.9 | 60.2 | 8.7 | 4.25 |
| Mo/NC-800 | 4.6 | 33.4 | 8.3 | 17.9 | 40.4 | 3.94 |
| Mo/NC-500  Recycling of catalyst | 2.0 | 65.6 | 21.0 | 13.4 |  | 5.52 |

^a^ The Mo atomic percentage in the catalysts were determined by XPS; ^b^ Mo/NC-500 treated with benzyl alcohol.

**Table S4.** The binding energies and the percentage of the peak area of different types of nitrogen in the Mo/NC-T and Mo/C-500 catalysts.

|  | **N atomic percentage ^a^**  **(at. %)** | **Pyridinic N** | | **Pyrrolic N** | | **Graphitic N** | | **Oxidized N** | | **Mo 3p_3/2_** |
| --- | --- | --- | --- | --- | --- | --- | --- | --- | --- | --- |
|  |  | **Binding energy (eV)** | **Percentage of peak area (%)** | **Binding energy (eV)** | **Percentage of peak area (%)** | **Binding energy (eV)** | **Percentage of peak area (%)** | **Binding energy (eV)** | **Percentage of peak area (%)** | **Binding energy (eV)** |
| Mo/NC-450 | 30.8 | 398.2 | 48.6 | 399.6 | 41.1 | 400.8 | 8.1 | 402.3 | 2.2 | 396.7 |
| Mo/C-500 | 7.6 | 398.2 | 29.1 | 399.4 | 43.9 | 400.7 | 12.5 | 401.8 | 14.5 | 396.5 |
| Mo/NC-500 | 27.4 | 398.3 | 49.6 | 399.7 | 36.5 | 400.9 | 11.3 | 402.5 | 2.6 | 396.4 |
| Mo/NC-500- Benzyl alcohol ^b^ | 22.0 | 398.4 | 42.1 | 399.7 | 39.2 | 400.8 | 16.1 | 402.3 | 2.6 | 396.4 |
| Mo/NC-600 | 21.4 | 398.4 | 55.5 | 399.8 | 23.9 | 401.0 | 17.3 | 402.8 | 3.3 | 396.5 |
| Mo/NC-800 | 18.1 | 398.6 | 58.3 | 399.8 | 17.1 | 401.3 | 21.1 | 403.0 | 3.5 | 396.6 |
| Mo/NC-500  Recycling of catalyst | 27.5 | 398.3 | 50.9 | 399.7 | 34.9 | 400.9 | 11.6 | 402.5 | 2.6 | 396.4 |

^a^ The N atomic percentage in the catalysts were determined by XPS; ^b^ Mo/NC-500 treated with benzyl alcohol.

**Table S5.** Percentage of the peak area of different types of carbon in the Mo/NC-T and Mo/C-500 catalysts.

| **Catalyst** | **C atomic percentage ^a^**  **(at. %)** | **C-C/C=C**  **(%)** | **C-N**  **(%)** | **C-O**  **(%)** | **C=O**  **(%)** | **O-C=O**  **(%)** | **C-Mo**  **(%)** | **π-π***  **(%)** |
| --- | --- | --- | --- | --- | --- | --- | --- | --- |
| Mo/NC-450 | 59.3 | 56.3 | 17.9 | 14.9 | 8.2 | 2.7 | - | - |
| Mo/C-500 | 68.1 | 69.0 | 14.0 | 12.5 | 2,7 | 1.8 | - | - |
| Mo/NC-500 | 59.4 | 58.6 | 20.3 | 12.9 | 5.9 | 2.3 | - | - |
| Mo/NC-500-Benzyl alcohol ^b^ | 63.1 | 61.4 | 18.1 | 11.4 | 4.3 | 2.3 | - | 2.5 |
| Mo/CN-600 | 59.6 | 62.3 | 22.8 | 8.8 | 4.0 | 2.1 | - | - |
| Mo/CN-800 | 59.8 | 64.5 | 19.0 | 9.4 | 2.3 | 2.1 | 2.7 | - |
| Mo/NC-500  Recycling of catalyst | 59.0 | 58.1 | 20.2 | 12.7 | 5.9 | 2.3 | - | 0.8 |

^a^ The C atomic percentage in the catalysts were determined by XPS; ^b^ Mo/NC-500 treated with benzyl alcohol.

**Table S6.** Percentage of the peak area of different types of oxygen in the Mo/NC-T and Mo/C-500 catalysts.

| **Catalyst** | **O atomic percentage**  **(at.%) ^a^** | **Mo-O (%)** | **O_v_ (%)** | **C=O (%)** | **C-O (%)** |
| --- | --- | --- | --- | --- | --- |
| Mo/NC-450 | 20.1 | 53.3 | 10.7 | 18.6 | 17.4 |
| Mo/C-500 | 9.1 | 60.6 | 4.2 | 15.4 | 19.8 |
| Mo/NC-500 | 11.2 | 40.0 | 24.1 | 18.3 | 17.6 |
| Mo/NC-500-Benzyl alcohol ^b^ | 12.9 | 55.7 | 9.5 | 19.2 | 15.6 |
| Mo/NC-600 | 15.4 | 54.7 | 18.3 | 13.9 | 13.1 |
| Mo/NC-800 | 17.5 | 72.7 | 0 | 14.1 | 13.2 |
| Mo/NC-500  Recycling of catalyst | 11.5 | 43.1 | 20.6 | 18.5 | 17.8 |

^a^ The O atomic percentage in the catalysts were determined by XPS.

**Table S7.** EXAFS fitting parameters at the Mo K-edge of various samples (*Ѕ*_0_^2^=0.88).

| **Sample** | **Path** | **C.N.** | **R (Å)** | **σ^2^×10^-3^ (Å^2^)** | **ΔE (eV)** | **R factor** |
| --- | --- | --- | --- | --- | --- | --- |
| Mo foil | Mo-Mo | 8* | 2.72±0.01 | 4.2±0.7 | -6.1±1.0 | 0.002 |
|  | Mo-Mo | 6* | 3.14±0.01 | 4.0±1.0 | -3.4±2.0 |  |
| MoO_3_ | Mo-O_I_ | 1.1±0.3 | 1.76±0.01 | 2.4±1.0 | 5.0±0.8 | 0.010 |
|  | Mo-O_II_ | 2.4±0.9 | 2.18±0.03 | 14.6±3.1 | -16.8±8.2 |  |
| Mo/NC-500 | Mo-O_I_ | 1.4±0.5 | 1.69±0.02 | 8.2±1.9 | -12.5±7.2 | 0.010 |
| Mo/C-500 | Mo-O | 3.2±0.8 | 2.00±0.02 | 7.7±2.9 | 9.9±2.6 | 0.016 |

*^a^N*: coordination numbers; *^b^R*: bond distance; *^c^σ*^2^: Debye-Waller factors; *^d^* Δ*E*_0_: the inner potential correction. *R* factor: goodness of fit. * The experimental EXAFS fit of metal foil by fixing CN as the known crystallographic value.

**Table S8.** The results of the dehydrogenative coupling of nitrobenzene with ethanol. ^a^

|  | | | | |
| --- | --- | --- | --- | --- |
| **Entry** | **Catalyst** | **Con. (%)** | **Selectivity (%)** | |
|  |  |  | **1** | **2** |
| 1 | Mo/NC-450 | 20.2 | 35.6 | 64.4 |
| 2 | Mo/NC-500 | 35.5 | 25.1 | 74.9 |
| 3 | Mo/NC-600 | 21.3 | 34.7 | 65.3 |
| 4 | Mo/NC-700 | 12.4 | 43.6 | 56.4 |
| 5 | Mo/NC-800 | 11.5 | 48.2 | 51.8 |
| 6^b^ | NC-500 | 0 | - | - |
| 7 | MoO_3_ | 3.5 | 36.4 | 15.2 |
| 8 | MoO_2_ | - | - | - |
| 9 | Mo_2_C | 1.1 | 72.5 | 27.5 |
| 10 ^c^ | Mo/NC-500 | 36.2 | 25.5 | 74.5 |
| 11 | Mo/C-500 | 3.5 | 68.9 | 31.1 |
| 12 ^d^ | Mo/C-500 | 13.8 | 48.6 | 51.4 |
| 13 | Mo/C-600 | <1 | - | - |
| 14 | Mo/C-700 | 2.1 | 69.7 | 30.3 |
| 15 | Mo/C-800 | 7.4 | 52.3 | 47.7 |
| 16 | Pd/C | 3.1 | 64.5 | 35.5 |
| 17 | Pt/C | <1 | - | - |
| 18 | Ru/C | 31.4 | 29.0 | 42.4 |
| 19^e^ | Mo/NC-500 | >99 | 4.3 | 95.7 |
| 20^f^ | Mo/NC-500 | >99 | 3.8 | 96.2 |

^a^ Reaction conditions: nitrobenzene (1.0 mmol), Mo or other metal loading (mole percentage of metal to nitrobenzene = 2.1 mol%), 180 °C, ethanol (10 mL), and 5 h; ^b^ The loading of NC-500 was 20 mg; ^c^ The Mo/NC-500 catalyst was placed in a glass bottle, sealed with sealing film, and stored directly in a desiccator for three years before being used for catalytic testing. ^d^ 200 °C and 20 h. ^e^ Same as “a”, using nitrosobenzene (1.0 mmol). ^f^ Same as “a”, using *N*-phenylhydroxylamine (1.0 mmol).

**Table S9.** The results of the dehydrogenative coupling of nitrobenzene with benzyl alcohol in different solvents. ^a^

| **** | | | | |
| --- | --- | --- | --- | --- |
| **Entry** | **Solvent** | **C (nitrobenzene)** | **Y (aniline)** | **Y (*N*-Benzylideneaniline)** |
| 1 | THF | 51.8 | 20.8 | 31.0 |
| 2 | Toluene | 61.1 | 16.3 | 44.8 |
| 3 | H_2_O | 24.1 | 8.2 | 15.9 |
| 4 | Acetonitrile | 84.9 | 12.0 | 72.9 |
| 5 | Hexane | 78.8 | 2.6 | 76.2 |
| 6^b^ | Acetonitrile | 25.9 | 12.8 | 13.1 |
| 7^c^ | Acetonitrile | 100 | 0.5 | >99 |
| 8^d^ | Acetonitrile | 100 | 0.4 | 95.9 |

^a^ Reaction conditions: nitrobenzene (1.0 mmol), benzyl alcohol (5.0 equiv.), a certain amount of the catalyst with Mo loading of 2.1 mol%, 200 °C, solvents (10 mL), 10 bar N_2_, and 8 h. C for Conversion, Y for Yield. ^b^ Same as “a”, but the reaction temperature is 180 °C. ^c^ Same as “a”, but the reaction time is 11 h. ^d^ Same as “a”, but the benzyl alcohol (3.0 equiv.), and the reaction time is 18 h.

**Table S10**. Comparison of Mo/NC-500 with state-of-the-art catalysts in the hydrogen transfer coupling of alcohols and nitro compounds.

| **Entry** | **Catalyst** | **additives (mol%)** | **Reductant or Oxidant** | **Conditions** | **Product** | **C/Y (%)** | ***Ref*** |
| --- | --- | --- | --- | --- | --- | --- | --- |
| 1 | Mo/NC-500 | - | Alcohols | 200 °C, N_2_, 17 h  1.0 mmol nitro compounds |  | >99/94 | This work |
| 2 | Mo/NC-500 | - | Alcohols | 200 °C, N_2_, 10 h  1.0 mmol nitro compounds |  | >99/97 | This work |
| 3 | Mo/NC-500 | - | Alcohols | 200 °C, N_2_, 8 h  1.0 mmol nitro compounds |  | >99/99 | This work |
| 4 | Mo/NC-500 |  |  | 200 °C, N_2_, 9 h  1.0 mmol nitro compounds |  | >99/99 | This work |
| 5 | Mo/NC-500 |  | Vicinal diols | 200 °C, N_2_, 10 h  1.0 mmol nitro compounds |  | >99/99 | This work |
| 6 | MgO/NC-500 | - | Alcohols | 200 °C, N_2_, 18 h  0.5 mmol nitro compounds |  | >99/94 | 15 |
| 7 | MgO/NC-500 | - | Vicinal diols | 200 °C, N_2_, 36 h  0.5 mmol nitro compounds |  | 98/98 | 15 |
| 8 | CeO_2_ | - | Ethanol | 140 °C, N_2_, 28 h  0.5 mmol nitro compounds |  | 99/97 | 31 |
| 9 | Co-ZnO/NC | - | Aromatic alcohols | 160 °C, N_2_, 4 h  1.0 mmol nitroarene |  | 94/92 | 21 |

The “C” stands for conversion and “Y” stands for yield.

**Table S11.** The control experiments.

| **Entry** | **Substrate** | **Conversion (%)** | | | **Yield (%)** | | |
| --- | --- | --- | --- | --- | --- | --- | --- |
|  |  | Nitrobenzene | Aniline | Benzyl alcohol | Benzaldehyde | Aniline | Imine |
| 1^a^ | Nitrobenzene | - | 6.5 | - | - | 6.5 | - |
| 2^b^ | Benzyl alcohol | - | - | 8.3 | 8.3 | - | - |
| 3^c^ | Nitrobenzene + Benzyl alcohol | 14.8 | - | 49.3 | 41.8 | 7.3 | 7.5 |
| 4^c^ | Aniline +  Benzyl alcohol | - | 8.7 | 11.6 | 2.9 | 93.1 | 8.7 |

^a^ Reaction conditions: substrate (1.0 mmol), the Mo/NC-500 catalyst (20 mg), acetonitrile (10 mL), 200 °C, H_2_ (1 bar) and 4 h. ^b^ Same as “a”, but using N_2_ (10 bar). ^c^ Same as “a”, but using nitrobenzene (aniline), benzyl alcohol = 1:1, and N_2_ (10 bar).

# Supplementary Data

**^1^H NMR and ^13^C NMR for products**

The imines products were purified by flash column chromatography on silica gel using hexane, dichloromethane and trienthyl amine (hexane/ dichloromethane/trienthyl amine = 100/10/5, v/v/v) as eluent. To avoid the hydrolysis of the produced imine during the purification, the silica gel was washed with triethyl amine before the purification. The imidazole, quinoxaline, oxazole and thiazolyl products were purified using ethyl acetate and hexane (ethyl acetate/ hexane = 70/30, v/v) as eluent. NMR experiment was conducted on Bruker TCI IIITM 600 MHz. GC-MS experiment was conducted on Thermo Scientific™ ISQ™ 7000 Single Quadrupole GC-MS (Thermo Scientific, USA).

**1. *N*-methyleneaniline, CAS:** **100-62-9**

^1^H NMR (600 MHz, CDCl_3_) δ 7.23 (t, *J* = 8.0 Hz, 2H), 7.03 (d, *J* = 7.8 Hz, 2H), 6.89 (t, *J* = 7.3 Hz, 1H), 4.91 (s, 2H). ^13^C NMR (151 MHz, CDCl_3_) δ 148.55, 129.12, 120.88, 117.63, 68.56. MS (EI, m/z): 105 (M^+^).

This compound was known [1].

**2. *N*-ethylideneaniline, CAS: 6052-11-5**

^1^H NMR (600 MHz, CD_3_OD) δ 7.16 – 7.11 (m, 2H), 6.74 (dd, J = 8.6, 1.0 Hz, 2H), 6.68 (tt, *J* = 7.4, 1.0 Hz, 1H), 1.40 (d, *J* = 5.8 Hz, 3H). ^13^C NMR (151 MHz, CD_3_OD) δ 166.07, 147.95, 130.33, 130.10, 121.81, 118.97, 114.76, 19.90. MS (EI, m/z): 119 (M^+^).

This compound was known [2].

**3. *N*-propylideneaniline, CAS: 7138-58-1**

^1^H NMR (600 MHz, CD_3_OD) δ 7.12 – 7.07 (m, 2H), 6.75 – 6.70 (m, 2H), 6.66 – 6.62 (m, 1H), 1.83 – 1.60 (m, 2H), 0.96 (t, *J* = 7.5 Hz, 3H). ^13^C NMR (151 MHz, CD_3_OD) δ 169.15, 147.00, 128.66, 120.40, 117.37, 113.26, 86.53, 26.17, 8.75. MS (EI, m/z): 133 (M^+^).

This compound was known [3,4].

**4. *N*-butylideneaniline, CAS: 4275-07-4**

^1^H NMR (600 MHz, CD_3_OD) δ 7.10 – 7.05 (m, 2H), 6.71 (dd, *J* = 8.6, 1.0 Hz, 2H), 6.64 – 6.60 (m, 1H), 1.78 – 1.58 (m, 2H), 1.47 – 1.36 (m, 2H), 0.94 (t, *J* = 7.4 Hz, 3H). ^13^C NMR (151 MHz, CD_3_OD) δ 168.45, 146.97, 128.62, 120.35, 117.32, 113.19, 85.05, 35.33, 18.33, 12.85. MS (EI, m/z): 147 (M^+^).

This compound was known [5].

**5. *N*-pentylideneaniline, CAS: 118793-03-6**

^1^H NMR (600 MHz, CD_3_OD) δ 7.16 – 7.10 (m, 2H), 6.78 – 6.73 (m, 2H), 6.67 (td, *J* = 7.4, 1.0 Hz, 1H), 1.83 – 1.58 (m, 2H), 1.45 – 1.33 (m, 4H), 0.94 (t, *J* = 7.1 Hz, 3H). ^13^C NMR (151 MHz, CD_3_OD) δ 168.42, 146.97, 128.73, 120.43, 117.42, 113.30, 85.18, 33.05, 27.40, 22.28, 13.23. MS (EI, m/z): 161 (M^+^).

This compound has been previously prepared in the literature [4], but ^1^H NMR and ^13^C NMR data were not reported.

**6. *N*-hexylidenebenzenamine, CAS: 117555-73-4**

^1^H NMR (600 MHz, CD_3_OD) δ 7.14 – 7.10 (m, 2H), 6.78 – 6.74 (m, 2H), 6.69 – 6.64 (m, 1H), 1.82 – 1.60 (m, 2H), 1.45 – 1.30 (m, 6H), 0.92 (t, *J* = 7.0 Hz, 3H). ^13^C NMR (151 MHz, CD_3_OD) δ 168.41, 146.96, 128.73, 120.44, 117.43, 113.32, 85.21, 33.33, 31.50, 24.91, 22.41, 13.22. MS (EI, m/z): 175 (M^+^).

This compound was known [6].

**7. *N*-heptylidenebenzenamine, CAS: 4275-05-2**

^1^H NMR (600 MHz, CD_3_OD) δ 7.14 – 7.08 (m, 2H), 6.78 – 6.74 (m, 2H), 6.69 – 6.64 (m, 1H), 1.83 – 1.60 (m, 2H), 1.45 – 1.27 (m, 8H), 0.91 (t, *J* = 7.0 Hz, 3H). ^13^C NMR (151 MHz, CD_3_OD) δ 168.41, 146.96, 128.71, 120.42, 117.41, 113.30, 85.21, 33.35, 31.67, 28.95, 25.18, 22.37, 13.23. MS (EI, m/z): 189 (M^+^).

This compound was known [7].

**8. *N*-(2-Methylpropylidene) benzenamine, CAS: 7020-77-1**

Extremely unstable compound. Due to the instability of this compound, purification was not performed. The excess of aldehyde was removed under vacuum (see crude GC-MS spectra below). MS (EI, m/z): 147 (M^+^).

This compound hasn’t been previously prepared in the literature.

**9. *N*-(cyclohexylmethylene) benzenamine, CAS: 62582-99-4**

^1^H NMR (600 MHz, CD_3_OD) δ 7.77 (d, *J* = 5.3 Hz, 1H), 7.35 (t, *J* = 7.8 Hz, 2H), 7.21 (t, *J* = 7.4 Hz, 1H), 7.05 (dd, *J* = 8.3, 1.0 Hz, 2H), 2.38 (d, *J* = 4.0 Hz, 1H), 1.44 – 1.16 (m, 10H). ^13^C NMR (151 MHz, CD_3_OD) δ 171.69, 128.83, 128.65, 125.56, 120.35, 43.89, 29.05, 28.89, 28.27, 26.17, 25.11. MS (EI, m/z): 187 (M^+^)

This compound was known. Due to the instability of this compound, the excess of aldehyde was removed under vacuum (see crude spectra below). The spectral data of the product are reported in the literature [8].

**10. *N*-(1-methylethylidene) benzenamine, CAS: 1124-52-3**

This compound has been previously prepared in the literature [9]. Extremely unstable compound, Due to the instability of this compound, purification was not performed. The excess of aldehyde was removed under vacuum (see crude GC-MS spectra below). MS (EI, m/z): 133 (M^+^).

**11. *N*-benzylideneaniline, CAS: 538-51-2**

^1^H NMR (600 MHz, CDCl_3_) δ 8.50 (s, 1H), 7.95 (dd, *J* = 7.0, 2.3 Hz, 2H), 7.52 (dd, *J* = 7.6, 2.4 Hz, 3H), 7.44 (t, *J* = 7.8 Hz, 2H), 7.31 – 7.25 (m, 3H). ^13^C NMR (151 MHz, CDCl_3_) δ 160.50, 152.10, 136.22, 131.46, 129.21, 128.84, 126.01, 120.93. MS (EI, m/z): 181 (M^+^).

This compound was known [10].

**12. *N*-(4-methylbenzylidene) aniline****, CAS: 2362-77-8**

^1^H NMR (600 MHz, CDCl_3_) δ 8.43 (s, 1H), 7.82 (d, *J* = 8.0 Hz, 2H), 7.41 (t, *J* = 7.8 Hz, 2H), 7.29 (d, *J* = 7.9 Hz, 2H), 7.24 (dd, *J* = 5.6, 2.8 Hz, 3H), 2.43 (s, 3H). ^13^C NMR (151 MHz, CDCl_3_) δ 159.27, 151.13, 140.76, 132.55, 128.43, 128.04, 127.73, 124.68, 119.82, 20.55. MS (EI, m/z): 195 (M^+^).

This compound was known [10].

**13. *N*-(4-methoxybenzylidene) aniline, CAS: 836-41-9 211.3**

^1^H NMR (600 MHz, CDCl_3_) δ 8.39 (s, 1H), 7.86 (d, *J* = 8.6 Hz, 2H), 7.40 (t, *J* = 7.7 Hz, 2H), 7.22 (t, *J* = 9.5 Hz, 3H), 6.99 (d, *J* = 8.6 Hz, 2H), 3.86 (s, 3H). ^13^C NMR (151 MHz, CDCl_3_) δ 161.16, 158.69, 151.26, 129.46, 128.15, 128.06, 124.52, 119.83, 113.11, 54.35. MS (EI, m/z): 211 (M^+^).

This compound was known [11].

**14. *N*-(1-phenylethylidene) aniline, CAS: 1749-19-5**

^1^H NMR (600 MHz, CDCl_3_) δ 7.99 (dd, *J* = 7.8, 1.7 Hz, 2H), 7.50 – 7.43 (m, 3H), 7.36 (t, *J* = 7.8 Hz, 2H), 7.10 (t, *J* = 7.4 Hz, 1H), 6.83 – 6.79 (m, 2H), 2.24 (s, 3H). ^13^C NMR (151 MHz, CDCl_3_) δ 164.47, 150.64, 138.42, 129.45, 127.92, 127.34, 126.13, 122.18, 118.34, 16.37. MS (EI, m/z): 195 (M^+^)

This compound was known [10].

**15. *N*-(4-hydroxybenzilidene) aniline, CAS:1689-73-2**

^1^H NMR (600 MHz, DMSO-*d*_6_) δ 9.69 (s, 1H), 8.41 (s, 1H), 7.76 – 7.72 (m, 2H), 7.38 – 7.33 (m, 2H), 7.19 – 7.15 (m, 3H), 6.88 – 6.85 (m, 2H). ^13^C NMR (151 MHz, DMSO-*d*_6_) δ 161.65, 160.56, 152.45, 131.17, 129.61, 127.59, 125.77, 121.33, 116.23. MS (EI, m/z): 197 (M^+^)

This compound was known [12].

**16. *N*-(4-fluorobenzylidene) aniline, CAS: 5676-81-3**

^1^H NMR (600 MHz, CDCl_3_) δ 8.38 (s, 1H), 7.89 (dd, *J* = 8.6, 5.6 Hz, 2H), 7.40 (t, *J* = 7.8 Hz, 2H), 7.22 (dd, *J* = 5.4, 3.0 Hz, 3H), 7.14 (t, *J* = 8.6 Hz, 2H). ^13^C NMR (151 MHz, CDCl_3_) δ 164.38, 162.70, 157.64, 150.69, 131.46, 131.44, 129.70, 129.65, 128.09, 124.94, 119.77, 114.86, 114.71. MS (EI, m/z): 199 (M^+^)

This compound was known [10].

**17. *N*-(4-chlorobenzylidene) aniline, CAS: 1613-95-2**

^1^H NMR (600 MHz, CDCl_3_) δ 8.40 (s, 1H), 7.83 (d, *J* = 8.3 Hz, 2H), 7.46 – 7.37 (m, 4H), 7.23 (dd, *J* = 18.4, 7.7 Hz, 3H). ^13^C NMR (151 MHz, CDCl_3_) δ 157.77, 150.57, 136.28, 133.60, 128.89, 128.15, 128.00, 125.15, 119.81. MS (EI, m/z): 215.7 (M^+^)

This compound was known [10].

**18. *N*-(4-bromobenzylidene) aniline, CAS: 1613-97-4**

^1^H NMR (600 MHz, CDCl_3_) δ 8.39 (s, 1H), 7.78 – 7.74 (m, 2H), 7.62 – 7.58 (m, 2H), 7.42 – 7.37 (m, 2H), 7.26 – 7.20 (m, 3H). ^13^C NMR (151 MHz, CDCl_3_) δ 157.87, 150.54, 134.01, 130.96, 129.09, 128.16, 125.19, 124.82, 119.80. MS (EI, m/z): 260 (M^+^)

This compound was known [13].

**19. *N*-[[4-(Trifluoromethyl) phenyl] methylene] benzenamine, CAS: 79128-83-9**

^1^H NMR (600 MHz, CDCl_3_) δ 8.53 (s, 1H), 8.04 (d, *J* = 8.0 Hz, 2H), 7.75 (d, *J* = 8.1 Hz, 2H), 7.44 (t, *J* = 7.8 Hz, 2H), 7.33 – 7.26 (m, 3H). ^13^C NMR (151 MHz, CDCl_3_) δ 157.53, 150.30, 138.19, 131.78, 131.56, 128.23, 127.92, 125.56, 124.70, 124.67, 123.74, 121.94, 119.86. MS (EI, m/z): 249 (M^+^)

This compound was known [13].

**20. *N*-(2-furanylmethylene) benzenamine, CAS: 3237-23-8**

^1^H NMR (600 MHz, CDCl_3_) δ 8.27 (s, 1H), 7.60 (s, 1H), 7.40 – 7.35 (m, 2H), 7.23 (dd, *J* = 12.8, 8.3 Hz, 3H), 6.96 – 6.91 (m, 1H), 6.56 – 6.52 (m, 1H). ^13^C NMR (151 MHz, CDCl_3_) δ 150.98, 150.28, 146.69, 144.63, 128.14, 125.21, 119.98, 115.39, 111.14. MS (EI, m/z): 171 (M^+^)

This compound was known [14].

**21. *N*-[(5-methyl-2-furanyl) methylene] benzenamine, CAS: 61973-96-4**

^1^H NMR (600 MHz, CDCl_3_) δ 8.16 (s, 1H), 7.36 (t, *J* = 7.8 Hz, 2H), 7.23 – 7.18 (m, 3H), 6.82 (d, *J* = 3.2 Hz, 1H), 6.19 – 6.10 (m, 1H), 2.41 (s, 3H). ^13^C NMR (151 MHz, CDCl_3_) δ 155.80, 150.70, 149.63, 146.55, 128.07, 124.83, 120.00, 117.89, 107.78, 13.06. MS (EI, m/z): 185 (M^+^)

This compound was known [11].

**22. *N*-(4-pyridinylmethylene) aniline, CAS: 27768-46-3**

^1^H NMR (600 MHz, CDCl_3_) δ 8.71 (d, *J* = 4.5 Hz, 2H), 8.41 (s, 1H), 7.71 (d, *J* = 4.5 Hz, 2H), 7.42 – 7.34 (m, 2H), 7.22 (dd, *J* = 23.0, 7.8 Hz, 3H). ^13^C NMR (151 MHz, CDCl_3_) δ 156.93, 149.86, 149.52, 141.73, 128.27, 126.00, 121.24, 119.89. MS (EI, m/z): 182 (M^+^)

This compound was known [15].

**23. *N*-benzylidene-4-toluidine, CAS: 2272-45-9**

^1^H NMR (600 MHz, CDCl_3_) δ 8.47 (s, 1H), 7.90 (dd, *J* = 6.4, 3.0 Hz, 2H), 7.49 – 7.46 (m, 3H), 7.21 (d, *J* = 8.0 Hz, 2H), 7.15 (d, *J* = 8.2 Hz, 2H), 2.38 (s, 3H). ^13^C NMR (151 MHz, CDCl_3_) δ 158.63, 148.38, 135.26, 134.79, 130.19, 128.73, 127.72, 127.67, 119.78, 19.99. MS (EI, m/z): 195 (M^+^)

This compound was known [16].

**24. *N*-benzylidene-3-toluidine, CAS: 5877-58-7**

^1^H NMR (600 MHz, CDCl_3_) δ 8.46 (s, 1H), 7.90 (d, *J* = 7.3 Hz, 2H), 7.50 – 7.45 (m, 3H), 7.29 (t, *J* = 7.7 Hz, 1H), 7.04 (dt, *J* = 13.0, 8.1 Hz, 3H), 2.40 (s, 3H). ^13^C NMR (151 MHz, CDCl_3_) δ 159.20, 151.02, 137.95, 135.19, 130.29, 127.94, 127.73, 125.68, 120.59, 116.80, 20.39. MS (EI, m/z): 195 (M^+^)

This compound was known [16].

**25. *N*-benzylidene-2-toluidine, CAS: 5877-55-4**

^1^H NMR (600 MHz, CDCl_3_) δ 8.38 (s, 1H), 7.94 (dd, *J* = 6.5, 3.0 Hz, 2H), 7.49 (dd, *J* = 7.4, 2.9 Hz, 3H), 7.23 (dd, *J* = 11.9, 7.6 Hz, 2H), 7.17 – 7.13 (m, 1H), 6.94 (d, *J* = 7.6 Hz, 1H), 2.38 (s, 3H). ^13^C NMR (151 MHz, CDCl_3_) δ 158.44, 150.07, 135.39, 130.82, 130.20, 129.20, 127.70, 125.68, 124.60, 116.62, 16.83. MS (EI, m/z): 195 (M^+^)

This compound was known [17].

**26. *N*-benzylidene-4-anisidine, CAS: 783-08-4**

^1^H NMR (600 MHz, CDCl_3_) δ 8.49 (s, 1H), 7.90 (dd, *J* = 6.4, 2.9 Hz, 2H), 7.49 – 7.45 (m, 3H), 7.25 (d, *J* = 8.9 Hz, 2H), 6.94 (d, *J* = 8.8 Hz, 2H), 3.84 (s, 3H). ^13^C NMR (151 MHz, CDCl_3_) δ 157.46, 157.22, 143.84, 135.36, 130.03, 127.71, 127.55, 121.17, 113.33, 54.47. MS (EI, m/z): 211 (M^+^)

This compound was known [18].

**27. *N*-benzylidene-4-vinyl-aniline, CAS: 19406-84-9**

^1^H NMR (600 MHz, CDCl_3_) δ 8.46 (s, 1H), 7.89 (dd, *J* = 4.9, 2.8 Hz, 2H), 7.50 – 7.41 (m, 5H), 7.18 (d, *J* = 8.2 Hz, 2H), 6.72 (dd, *J* = 17.6, 10.9 Hz, 1H), 5.73 (d, *J* = 17.6 Hz, 1H), 5.22 (d, *J* = 10.9 Hz, 1H). ^13^C NMR (151 MHz, CDCl_3_) δ 159.07, 150.41, 135.23, 135.11, 134.46, 130.41, 127.77, 126.03, 120.13, 112.36. MS (EI, m/z): 207 (M^+^).

This compound hasn’t been previously prepared in the literature.

**28. 4-hydroxy-*N*-benzylideneaniline, CAS: 588-53-4**

^1^H NMR (600 MHz, DMSO-*d*_6_) δ 9.62(s, 1H), 8.61 (s, 1H), 7.90 (dq, *J* = 6.9, 2.2, 1.8 Hz, 2H), 7.52 – 7.46 (m, 3H), 7.23 – 7.19 (m, 2H), 6.85 – 6.78 (m, 2H). ^13^C NMR (151 MHz, DMSO-*d*_6_) δ 157.61, 156.82, 142.99, 136.90, 131.32, 129.22, 128.73, 122.99, 116.18. MS (EI, m/z): 197 (M^+^)

This compound was known [19].

**29. *N*-benzylidene-4-fluoroaniline, CAS: 331-98-6**

^1^H NMR (600 MHz, CDCl_3_) δ 8.45 (s, 1H), 7.90 (dd, *J* = 7.4, 2.0 Hz, 2H), 7.51 – 7.46 (m, 3H), 7.23 – 7.19 (m, 2H), 7.11 – 7.06 (m, 2H). ^13^C NMR (151 MHz, CDCl_3_) δ 161.00, 159.38, 159.17, 146.97, 134.99, 130.43, 127.77, 127.73, 121.30, 121.24, 114.91, 114.76. MS (EI, m/z): 199 (M^+^)

This compound was known [20].

**30. *N*-benzylidene-4-chloroaniline, CAS: 780-21-2**

^1^H NMR (600 MHz, CDCl_3_) δ 8.44 (s, 1H), 7.91 – 7.88 (m, 2H), 7.49 (t, *J* = 7.1 Hz, 3H), 7.36 (d, *J* = 8.6 Hz, 2H), 7.17 – 7.14 (m, 2H). ^13^C NMR (151 MHz, CDCl_3_) δ 159.76, 149.45, 134.87, 130.62, 130.43, 128.21, 128.17, 127.84, 127.81, 127.45, 126.77, 121.18. MS (EI, m/z): 215 (M^+^)

This compound was known [10].

**31. *N*-benzylidene-4-bromoaniline, CAS: 780-20-1**

^1^H NMR (600 MHz, CDCl_3_) δ 8.42 (s, 1H), 7.92 – 7.87 (m, 2H), 7.53 – 7.46 (m, 5H), 7.12 – 7.07 (m, 2H). ^13^C NMR (151 MHz, CDCl_3_) δ 159.68, 149.85, 134.80, 131.10, 130.58, 127.81, 127.75, 121.54, 118.24. MS (EI, m/z): 260 (M^+^).

This compound was known [10].

**32. *N*-[4-(trifluoromethyl) phenyl] benzaldimine, CAS: 351-98-4**

1H NMR (600 MHz, CDCl_3_) δ 8.43 (s, 1H), 7.94 – 7.89 (m, 2H), 7.65 (d, J = 8.3 Hz, 2H), 7.54 – 7.48 (m, 3H), 7.25 (d, J = 5.2 Hz, 2H). ^13^C NMR (151 MHz, CDCl_3_) δ 160.99, 154.16, 134.63, 130.95, 128.03, 127.86, 125.37, 125.34, 125.32, 125.29, 119.94. MS (EI, m/z): 249 (M^+^).

This compound was known [21].

**33. *N*-benzylidene-4-cyanoaniline, CAS: 17224-21-4**

^1^H NMR (600 MHz, CDCl_3_) δ 8.39 (s, 1H), 7.91 (d, *J* = 7.1 Hz, 2H), 7.65 (d, *J* = 8.4 Hz, 2H), 7.54 – 7.48 (m, 3H), 7.22 (d, *J* = 8.4 Hz, 2H). ^13^C NMR (151 MHz, CDCl_3_) δ 161.41, 154.95, 134.38, 132.26, 131.22, 128.15, 127.89, 120.52, 118.00, 107.88. MS (EI, m/z): 206 (M^+^).

This compound was known [22].

**34. *N*-phenylmethylene-4-pyridinamine, CAS: 19174-13-1**

^1^H NMR (600 MHz, CDCl_3_) δ 8.54 (d, *J* = 6.1 Hz, 2H), 8.32 (s, 1H), 7.88 – 7.85 (m, 2H), 7.50 – 7.42 (m, 3H), 7.00 – 6.96 (m, 2H). ^13^C NMR (151 MHz, CDCl_3_) δ 161.81, 157.94, 149.65, 134.29, 131.24, 128.15, 127.85, 114.64. MS (EI, m/z): 188 (M^+^).

This compound was known [23].

**35. *N*-benzylidene-1-naphthalenamine, CAS: 890-51-7**

^1^H NMR (600 MHz, CDCl_3_) δ 8.56 (s, 1H), 8.39 – 8.34 (m, 1H), 8.04 (dd, *J* = 6.7, 2.9 Hz, 2H), 7.87 (dd, *J* = 7.0, 2.3 Hz, 1H), 7.74 (d, *J* = 8.3 Hz, 1H), 7.56 – 7.51 (m, 5H), 7.48 (dd, *J* = 8.1, 7.3 Hz, 1H), 7.07 (dd, *J* = 7.2, 0.8 Hz, 1H). ^13^C NMR (151 MHz, CDCl_3_) δ 159.38, 148.27, 135.34, 132.85, 130.44, 127.94, 127.79, 127.72, 126.59, 125.38, 125.01, 124.76, 124.69, 122.92, 111.68. MS (EI, m/z): 231 (M^+^).

This compound was known [24].

**36. *N*-benzylidene-cyclohexylamine, CAS: 2211-66-7**

^1^H NMR (600 MHz, CDCl_3_) δ 8.27 (s, 1H), 7.73 (dd, *J* = 6.5, 3.0 Hz, 2H), 7.43 – 7.38 (m, 3H), 3.64 – 3.58 (m, 1H), 1.70 (p, *J* = 7.1 Hz, 2H), 1.38 – 1.26 (m, 5H), 0.89 (t, *J* = 6.8 Hz, 3H). ^13^C NMR (151 MHz, CDCl_3_) δ 160.76, 136.37, 130.46, 128.59, 128.03, 61.87, 31.70, 30.92, 27.07, 22.66, 14.12. MS (EI, m/z): 187 (M^+^).

This compound was known [25].

**37. *N*-benzylidenebenzylamine, CAS: 780-25-6**

^1^H NMR (600 MHz, CDCl_3_) δ 8.42 (s, 1H), 7.85 – 7.79 (m, 2H), 7.45 (qd, *J* = 4.7, 1.7 Hz, 3H), 7.38 (d, *J* = 4.6 Hz, 4H), 7.30 (dt, *J* = 8.9, 4.3 Hz, 1H), 4.86 (d, *J* = 1.2 Hz, 2H). ^13^C NMR (151 MHz, CDCl_3_) δ 162.00, 139.22, 136.07, 130.75, 128.58, 128.47, 128.24, 127.95, 126.96, 65.02. MS (EI, m/z): 195 (M^+^).

The compound was known [26].

**38. *N*-propylidenebenzenamine, CAS: 7138-58-1**

^1^H NMR (600 MHz, CDCl_3_) δ 8.26 (s, 1H), 7.76 – 7.71 (m, 2H), 7.40 (q, *J* = 3.5 Hz, 3H), 3.58 (td, *J* = 6.9, 1.3 Hz, 2H), 1.74 (h, *J* = 7.3 Hz, 2H), 0.97 (t, *J* = 7.4 Hz, 3H). ^13^C NMR (151 MHz, CDCl_3_) δ 159.72, 135.26, 129.37, 127.49, 126.96, 62.46, 23.00, 10.81. MS (EI, m/z): 133 (M^+^).

This compound hasn’t been previously prepared in the literature.

**39. *N*-butylidenebenzenamine, CAS: 4275-07-4**

^1^H NMR (600 MHz, CDCl_3_) δ 8.27 (s, 1H), 7.73 (dd, *J* = 6.6, 3.0 Hz, 2H), 7.41 (q, *J* = 3.6 Hz, 3H), 3.65 – 3.60 (m, 2H), 1.69 (p, *J* = 7.2 Hz, 2H), 1.39 (h, *J* = 7.4 Hz, 2H), 0.95 (t, *J* = 7.4 Hz, 3H). ^13^C NMR (151 MHz, CDCl_3_) δ 160.84, 136.33, 130.49, 128.61, 128.05, 61.52, 33.02, 20.49, 13.96. MS (EI, m/z): 147 (M^+^).

This compound was known [27].

**40. *N*-benzylidenemethylamine, CAS: 622-29-7**

^1^H NMR (600 MHz, CDCl_3_) δ 8.28 (q, J = 1.7 Hz, 1H), 7.71 (dd, J = 6.8, 3.0 Hz, 2H), 7.49 – 7.33 (m, 3H), 3.52 (d, J = 1.7 Hz, 3H). ^13^C NMR (151 MHz, CDCl_3_) δ 162.51, 136.27, 130.53, 128.62, 127.89, 48.25. MS (EI, m/z): 119 (M^+^).

This compound was known [28].

**41. *N*-butylidenepropylamine, CAS: 7707-71-3**

^1^H NMR (600 MHz, CD_3_OD) δ 7.73 – 7.69 (m, 1H), 2.27 (td, *J* = 7.4, 5.3 Hz, 2H), 1.69 – 1.47 (m, 6H), 1.03 – 0.87 (m, 6H). ^13^C NMR (151 MHz, CD_3_OD) δ 166.94, 62.13, 36.95, 23.40, 19.15, 12.93, 10.79. MS (EI, m/z): 113 (M^+^).

This compound was known [29].

**42. Consistent with product 11**

**43. benzimidazole, CAS: 51-17-2**

^1^H NMR (600 MHz, DMSO-*d*_6_) δ 12.73(s, 1H), 8.39 (s, 1H), 7.70 (dq, *J* = 6.5, 3.2 Hz, 2H), 7.23 (dt, *J* = 6.0, 3.4 Hz, 2H). ^13^C NMR (151 MHz, DMSO-*d*_6_) δ 142.55, 138.65, 122.33, 115.86. MS (EI, m/z): 118 (M^+^).

This compound was known [30].

**44. 2-methylbenzimidazole, CAS: 615-15-6**

^1^H NMR (600 MHz, CDCl_3_) δ 7.55 (dd, *J* = 5.9, 3.2 Hz, 2H), 7.22 (dd, *J* = 6.0, 3.1 Hz, 2H), 2.64 (s, 3H). ^13^C NMR (151 MHz, CDCl_3_) δ 150.21, 137.47, 121.20, 113.44, 13.89. MS (EI, m/z): 132 (M^+^).

This compound was known [30].

**45. 2-ethylbenzimidazole, CAS: 1848-84-6**

^1^H NMR (600 MHz, CDCl_3_) δ 7.56 (dd, *J* = 5.9, 3.2 Hz, 2H), 7.22 (dd, *J* = 6.0, 3.1 Hz, 2H), 3.01 (q, *J* = 7.6 Hz, 2H), 1.45 (t, *J* = 7.6 Hz, 3H). ^13^C NMR (151 MHz, CDCl_3_) δ 155.56, 137.47, 121.07, 113.54, 21.58, 11.43. MS (EI, m/z): 146 (M^+^).

This compound was known [30].

**46. 2-propylbenzimidazole, CAS: 5465-29-2**

^1^H NMR (600 MHz, CDCl_3_) δ 7.56 (dd, *J* = 5.9, 3.2 Hz, 2H), 7.22 (dq, *J* = 6.6, 3.5 Hz, 2H), 2.95 (t, *J* = 7.6 Hz, 2H), 1.90 (h, *J* = 7.4 Hz, 2H), 0.98 (t, *J* = 7.4 Hz, 3H). ^13^C NMR (151 MHz, CDCl_3_) δ 154.52, 137.53, 121.01, 113.54, 30.18, 20.71, 12.82. MS (EI, m/z): 160 (M^+^).

This compound was known [31].

**47. 2-butylbenzimidazole, CAS: 5851-44-5**

^1^H NMR (600 MHz, CDCl_3_) δ 7.55 (dd, *J* = 5.8, 3.1 Hz, 2H), 7.23 – 7.19 (m, 2H), 2.98 – 2.93 (m, 2H), 1.88 – 1.81 (m, 2H), 1.39 (h, *J* = 7.4 Hz, 2H), 0.88 (t, *J* = 7.4 Hz, 3H). ^13^C NMR (151 MHz, CDCl_3_) δ 154.52, 137.51, 121.03, 113.48, 29.36, 28.02, 21.38, 12.69. MS (EI, m/z): 174 (M^+^).

This compound was known [32].

**48. 2-amylbenzimidazole, CAS: 5851-46-7**

^1^H NMR (600 MHz, CDCl_3_) δ 7.55 (s, 2H), 7.21 (dq, *J* = 6.8, 3.8 Hz, 2H), 2.94 (t, *J* = 7.7 Hz, 2H), 1.86 (p, *J* = 7.7 Hz, 2H), 1.31 (ddd, *J* = 29.4, 15.0, 7.6 Hz, 4H), 0.83 (t, *J* = 7.1 Hz, 3H). ^13^C NMR (151 MHz, CDCl_3_) δ 154.48, 137.29, 121.04, 113.62, 30.45, 28.31, 27.00, 21.33, 12.87. MS (EI, m/z): 188 (M^+^).

This compound was known [33].

**49. 2-hexylbenzimidazole, CAS: 5851-48-9**

^1^H NMR (600 MHz, CDCl_3_) δ 7.57 (dd, *J* = 5.8, 3.0 Hz, 2H), 7.24 – 7.19 (m, 2H), 2.98 (t, *J* = 7.6 Hz, 2H), 1.87 (p, *J* = 7.8 Hz, 2H), 1.35 (p, *J* = 7.3 Hz, 2H), 1.22 (dt, *J* = 7.2, 3.7 Hz, 4H), 0.84 – 0.78 (m, 3H). ^13^C NMR (151 MHz, CDCl_3_) δ 154.74, 137.53, 120.97, 113.52, 30.44, 28.32, 28.00, 21.43, 12.95. MS (EI, m/z): 202 (M^+^).

This compound was known [34].

**50. 2-isopropylbenzimidazole, CAS: 5851-43-4**

^1^H NMR (600 MHz, CDCl_3_) δ 7.55 (dd, *J* = 5.6, 3.1 Hz, 2H), 7.20 (dt, *J* = 7.1, 3.6 Hz, 2H), 3.29 (ddd, *J* = 13.9, 9.1, 4.8 Hz, 1H), 1.46 (dd, *J* = 6.9, 4.1 Hz, 6H). ^13^C NMR (151 MHz, CDCl_3_) δ 159.10, 137.30, 121.08, 113.64, 28.04, 20.59. MS (EI, m/z): 160 (M^+^).

This compound was known [33].

**51. 2-phenylbenzimidazole, CAS: 716-79-0**

^1^H NMR (600 MHz, DMSO-*d*_6_) δ 12.96 (s, 1H), 8.22 (d, *J* = 7.4 Hz, 2H), 7.54 (dt, *J* = 38.2, 7.4 Hz, 5H), 7.23 (s, 2H). ^13^C NMR (151 MHz, DMSO-*d*_6_) δ 150.63, 143.22, 134.41, 129.58, 129.26, 128.37, 125.84, 121.95, 121.09, 118.30, 110.74. MS (EI, m/z): 194 (M^+^).

This compound was known [33].

**52. 2-(4-methylphenyl) benzimidazole, CAS: 120-03-6**

^1^H NMR (600 MHz, DMSO-*d*_6_) δ 12.87 (s, 1H), 8.09 (d, *J* = 7.9 Hz, 2H), 7.59 (s, 2H), 7.36 (d, *J* = 7.8 Hz, 2H), 7.20 (dd, *J* = 5.9, 3.0 Hz, 2H), 2.37 (s, 3H). ^13^C NMR (151 MHz, DMSO-*d*_6_) δ 151.86, 140.07, 130.00, 127.88, 126.86, 122.46, 21.44. MS (EI, m/z): 208 (M^+^).

This compound was known [35].

**53. 2-(4-methoxyphenyl) benzimidazole, CAS: 2620-81-7**

^1^H NMR (600 MHz, DMSO-*d*_6_) δ 12.85 (b, 1H), 8.09 (d, *J* = 5.3 Hz, 2H), 7.59 (s, 2H), 7.23 – 7.11 (m, 4H), 3.83 (s, 3H). ^13^C NMR (151 MHz, DMSO-*d*_6_) δ 160.72, 151.88, 126.87, 122.47, 121.30, 114.24, 55.08. MS (EI, m/z): 224 (M^+^).

This compound was known [35].

**54. 2-(4-hydroxyphenyl) benzimidazole, CAS: 6504-13-8**

This compound was known. Stable compound, Due to the 4-hydroxybenzyl alcohol and 4-hydroxy benzaldehyde are similar in polarity and solubility to the 2-(4-Hydroxyphenyl) benzimidazole, purification was not performed.

This compound was known. The spectral data of the product are reported in the literature [36].

**55. 2-(2-furanyl) benzimidazole CAS: 3878-19-1**

^1^H NMR (600 MHz, DMSO-*d*_6_) δ 12.96(s, 1H), 7.95 (s, 1H), 7.58 (dd, *J* = 49.4, 14.8 Hz, 2H), 7.21 (d, *J* = 3.3 Hz, 3H), 6.73 (dd, *J* = 3.2, 1.6 Hz, 1H). ^13^C NMR (151 MHz, Chloroform-*d*) δ 150.75, 149.89, 149.83, 148.85, 139.44, 127.81, 127.12, 123.92, 117.52, 116.58, 115.75. MS (EI, m/z): 184 (M^+^).

This compound was known [33].

**56. 2-(2-thienyl) benzimidazole, CAS:** **3878-18-0**

^1^H NMR (600 MHz, CD_3_OD) δ 7.72 (dd, J = 3.7, 1.2 Hz, 1H), 7.58 (dd, J = 5.0, 1.2 Hz, 3H), 7.20 (dt, J = 6.1, 3.6 Hz, 2H), 7.16 (dd, J = 5.0, 3.7 Hz, 1H). ^13^C NMR (151 MHz, CD_3_OD) δ 147.41, 132.58, 128.21, 127.77, 126.83. MS (EI, m/z): 200 (M^+^).

This compound was known [37,38].

**57. 2-styrylbenzimidazole, CAS:** **1456-19-5**

^1^H NMR (600 MHz, CD_3_OD) δ 7.54 (dd, J = 8.0, 6.4 Hz, 3H), 7.48 (dd, J = 6.0, 3.2 Hz, 2H), 7.34 – 7.30 (m, 2H), 7.28 – 7.24 (m, 1H), 7.19 – 7.15 (m, 2H), 7.08 (d, J = 16.6 Hz, 1H). ^13^C NMR (151 MHz, CD_3_OD) δ 151.17, 135.74, 135.46, 128.80, 128.58, 126.75, 122.59, 115.91. MS (EI, m/z): 220 (M^+^).

This compound was known [37].

**58. 2-(1-naphthalenyl)-1H-benzimidazole, CAS: 2562-81-4**

^1^H NMR (600 MHz, CD_3_OD) δ 12.80 (s, 1H), 9.03 – 8.86 (m, 1H), 8.00 – 7.85 (m, 3H), 7.65 (d, J = 7.8 Hz, 1H), 7.60 – 7.40 (m, 4H), 7.19 – 7.06 (m, 2H). ^13^C NMR (151 MHz, CD_3_OD) δ 151.83, 144.36, 134.92, 134.09, 130.98, 130.62, 128.86, 128.34, 128.00, 127.54, 126.82, 125.75, 123.13, 122.09, 119.55, 111.84. MS (EI, m/z): 244 (M^+^).

This compound was known [39].

**59. 2-methyl-1H-benzimidazole-6-carbonitrile, CAS: 92443-13-5**

^1^H NMR (600 MHz, CD_3_OD) δ 7.85 – 7.72 (m, 1H), 7.54 – 7.49 (m, 1H), 7.42 (dd, J = 8.3, 1.5 Hz, 1H), 2.55 (s, 3H). 13C NMR (151 MHz, CD_3_OD) δ 155.41, 125.35, 119.37, 104.47, 13.09.MS (EI, m/z): 157 (M^+^).

This compound was known [40].

**60. 2-methyl-3H-imidazo[4,5-b] pyridine, CAS: 68175-07-5**

^1^H NMR (600 MHz, CD_3_OD) δ 8.27 – 8.12 (m, 1H), 7.82 (dq, J = 8.0, 1.5 Hz, 1H), 7.22 – 7.07 (m, 1H), 2.55 (s, 3H). ^13^C NMR (151 MHz, CD_3_OD) δ 154.68, 142.70, 117.71, 13.42. MS (EI, m/z): 133 (M^+^).

This compound was known [41].

**61.** **Consistent with product 44**

**62. 2-methylbenzoxazole, CAS: 95-21-6**

^1^H NMR (600 MHz, CDCl_3_) δ 7.67 – 7.62 (m, 1H), 7.48 – 7.44 (m, 1H), 7.30 – 7.27 (m, 2H), 2.63 (d, *J* = 1.3 Hz, 3H). ^13^C NMR (151 MHz, CDCl_3_) δ 162.80, 149.92, 140.44, 123.41, 123.05, 118.37, 109.16, 13.51. MS (EI, m/z): 133 (M^+^).

This compound was known [42].

**63. 2-phenylbenzoxazole, CAS: 833-50-1**

^1^H NMR (600 MHz, DMSO-*d*_6_) δ 8.26 – 8.21 (m, 2H), 7.83 (ddd, *J* = 18.9, 7.1, 1.9 Hz, 2H), 7.68 – 7.63 (m, 3H), 7.45 (pd, *J* = 7.3, 1.4 Hz, 2H). ^13^C NMR (151 MHz, DMSO-*d*_6_) δ 162.69, 150.67, 141.95, 132.41, 129.77, 127.72, 126.87, 125.98, 125.33, 120.30, 111.39. MS (EI, m/z): 195 (M^+^).

This compound was known [43].

**64. 2-(4-methylphenyl) benzoxazole, CAS: 835-71-2**

^1^H NMR (600 MHz, CDCl_3_) δ 8.15 (d, J = 4.5 Hz, 2H), 7.80 – 7.73 (m, 1H), 7.60 – 7.53 (m, 1H), 7.38 – 7.30 (m, 4H), 2.44 (s, 3H). ^13^C NMR (151 MHz, CDCl_3_) δ 163.32, 150.70, 142.19, 142.08, 129.67, 127.61, 124.88, 124.50, 124.41, 119.86, 110.51, 21.66. MS (EI, m/z): 209 (M^+^).

This compound was known [43].

**65. 2-(3, 4-dimethoxyphenyl) benzoxazole, CAS: 92552-99-3**

1H NMR (600 MHz, CD_3_OD) δ 7.76 (dd, J = 8.4, 2.0 Hz, 1H), 7.70 (d, J = 2.0 Hz, 1H), 7.68 – 7.60 (m, 1H), 7.60 – 7.55 (m, 1H), 7.38 – 7.29 (m, 2H), 7.06 (d, J = 8.4 Hz, 1H), 3.89 (d, J = 19.0 Hz, 6H). 13C NMR (151 MHz, CD_3_OD) δ 163.38, 152.58, 150.49, 149.43, 141.40, 124.86, 124.53, 121.09, 118.89, 118.70, 111.32, 110.20, 109.99, 55.10. MS (EI, m/z): 255 (M^+^).

This compound was known [44].

**66. 2-methylbenzothiazole, CAS: 120-75-2**

^1^H NMR (600 MHz, CDCl_3_) δ 7.95 (d, *J* = 8.1 Hz, 1H), 7.84 – 7.81 (m, 1H), 7.45 (ddd, *J* = 8.3, 7.3, 1.2 Hz, 1H), 7.37 – 7.32 (m, 1H), 2.84 (s, 3H). ^13^C NMR (151 MHz, CDCl_3_) δ 165.98, 152.31, 134.59, 124.90, 123.67, 121.34, 120.37, 19.11. MS (EI, m/z): 149 (M^+^).

This compound was known [45].

**67. 2-phenylbenzothiazole, CAS: 883-93-2**

^1^H NMR (600 MHz, CD_3_OD) δ 8.15 – 8.10 (m, 2H), 8.04 (ddd, J = 12.4, 8.1, 1.1 Hz, 2H), 7.62 – 7.53 (m, 4H), 7.47 (ddd, J = 8.3, 7.2, 1.1 Hz, 1H).^13^C NMR (151 MHz, CD_3_OD) δ 168.58, 153.67, 134.69, 133.18, 131.04, 128.92, 127.09, 126.33, 125.30, 122.38, 121.58. MS (EI, m/z): 211 (M^+^).

This compound was known [45].

**68. Quinoxaline, CAS: 91-19-0**

^1^H NMR (600 MHz, CDCl_3_) δ 8.64 (s, 2H), 7.91 (dq, *J* = 6.3, 2.8 Hz, 2H), 7.55 (dt, *J* = 6.4, 3.1 Hz, 2H). ^13^C NMR (151 MHz, CDCl_3_) δ 144.85, 142.83, 129.86, 129.36. MS (EI, m/z): 130 (M^+^).

This compound was known [46].

**69. 2-methylquinoxaline, CAS: 7251-61-8**

^1^H NMR (600 MHz, CDCl_3_) δ 8.74 (s, 1H), 8.04 (dd, *J* = 32.8, 8.2 Hz, 2H), 7.76 – 7.68 (m, 2H), 2.78 (s, 3H). ^13^C NMR (151 MHz, CDCl_3_) δ 152.78, 145.01, 141.03, 139.92, 129.03, 128.14, 127.94, 127.62, 21.59. MS (EI, m/z): 144 (M^+^).

This compound was known [47].

**70. 2, 3-dimethylquinoxaline, CAS: 2379-55-7**

^1^H NMR (600 MHz, CDCl_3_) δ 7.90 (dt, *J* = 6.5, 3.2 Hz, 2H), 7.58 (dq, *J* = 6.2, 2.9 Hz, 2H), 2.64 (s, 6H). ^13^C NMR (151 MHz, CDCl_3_) δ 152.38, 139.93, 127.76, 127.19, 22.10. MS (EI, m/z): 158 (M^+^).

This compound was known [47].

**71. 2-ethylquinoxaline, CAS: 29750-44-5**

^1^H NMR (600 MHz, CDCl_3_) δ 8.76 (s, 1H), 8.05 (dd, *J* = 20.1, 8.2 Hz, 2H), 7.72 (dt, *J* = 23.4, 6.9 Hz, 2H), 3.05 (q, *J* = 7.6 Hz, 2H), 1.44 (t, *J* = 7.6 Hz, 3H). ^13^C NMR (151 MHz, CDCl_3_) δ 157.46, 144.55, 141.11, 140.17, 128.92, 128.12, 127.91, 127.81, 28.62, 12.44. MS (EI, m/z): 158 (M^+^).

This compound was known [47].

**72. 2-propylquinoxaline, CAS: 81516-60-1**

^1^H NMR (600 MHz, CDCl_3_) δ 8.74 (s, 1H), 8.05 (dd, *J* = 20.7, 8.2 Hz, 2H), 7.72 (dt, *J* = 23.0, 7.0 Hz, 2H), 3.02 – 2.96 (m, 2H), 1.88 (q, *J* = 7.5 Hz, 2H), 1.04 (t, *J* = 7.3 Hz, 3H). ^13^C NMR (151 MHz, CDCl_3_) δ 156.47, 144.83, 141.16, 140.17, 128.91, 128.13, 127.91, 127.82, 37.41, 21.87, 12.92. MS (EI, m/z): 172 (M^+^).

This compound was known [47].

**73. 2-butylquinoxaline, CAS: 65755-33-1**

^1^H NMR (600 MHz, CDCl_3_) δ 8.73 (s, 1H), 8.04 (dd, *J* = 20.0, 8.2 Hz, 2H), 7.70 (dt, *J* = 23.5, 7.0 Hz, 2H), 3.03 – 2.98 (m, 2H), 1.81 (p, *J* = 7.7 Hz, 2H), 1.45 (dt, *J* = 14.9, 7.4 Hz, 2H), 0.96 (t, *J* = 7.4 Hz, 3H). ^13^C NMR (151 MHz, CDCl_3_) δ 156.67, 144.81, 141.14, 140.13, 128.90, 128.11, 127.88, 127.79, 35.23, 30.64, 21.55, 12.88. MS (EI, m/z): 186 (M^+^).

This compound was known [47].

**74. 2-hexylquinoxaline, CAS: 85061-29-6**

^1^H NMR (600 MHz, CDCl_3_) δ 8.74 (s, 1H), 8.16 – 7.98 (m, 2H), 7.85 – 7.64 (m, 2H), 3.07 – 2.95 (m, 2H), 1.91 – 1.78 (m, 2H), 1.47 – 1.40 (m, 2H), 1.37 – 1.30 (m, 4H), 0.89 (t, J = 7.1 Hz, 3H). 13C NMR (151 MHz, CDCl_3_) δ 157.75, 145.86, 142.22, 141.21, 129.92, 129.18, 128.91, 128.87, 36.57, 31.64, 29.55, 29.14, 22.55, 14.06. MS (EI, m/z): 214 (M^+^).

This compound was known [48].

**75. 2-phenylquinoxaline, CAS: 5021-43-2**

^1^H NMR (600 MHz, CDCl_3_) δ 9.33 (s, 1H), 8.24 – 8.09 (m, 4H), 7.83 – 7.72 (m, 2H), 7.62 – 7.50 (m, 3H). ^13^C NMR (151 MHz, CDCl_3_) δ 151.90, 143.41, 142.33, 141.62, 136.82, 130.32, 130.22, 129.66, 129.58, 129.19, 129.16, 127.59. MS (EI, m/z): 206 (M^+^).

This compound was known [47].

**76. 1,2,3,4-tetrahydrophenazine, CAS: 4829-73-6**

^1^H NMR (600 MHz, CD_3_OD) δ 7.89 (dt, J = 6.6, 3.3 Hz, 2H), 7.69 (dd, J = 6.4, 3.4 Hz, 2H), 3.10 (ddd, J = 6.9, 4.8, 2.4 Hz, 4H), 2.03 – 1.98 (m, 4H). ^13^C NMR (151 MHz, CD_3_OD) δ 154.50, 140.63, 129.17, 127.40, 22.18. MS (EI, m/z): 184 (M^+^).

This compound was known [47,48].

**77. Consistent with product 71**

**78. Consistent with product 11**

**79. Consistent with product 51**

**80. Consistent with product 2**

**81. Consistent with product 11**

**82. Consistent with product 44**

**83. Consistent with product 51**

**84. Consistent with product 67**

**85. 2-(3,4-dimethoxyphenyl)-5-fluorobenzoxazole, CAS: 1049032-80-5**

^1^H NMR (600 MHz, CD_3_OD) δ 7.84 (dd, J = 8.4, 2.1 Hz, 1H), 7.76 (d, J = 2.1 Hz, 1H), 7.64 (dd, J = 8.8, 4.2 Hz, 1H), 7.41 (dd, J = 8.5, 2.6 Hz, 1H), 7.15 (td, J = 8.8, 8.4, 2.0 Hz, 2H), 3.94 (d, J = 12.6 Hz, 6H). ^13^C NMR (151 MHz, CD_3_OD) δ 161.12, 159.53, 152.84, 149.49, 146.98, 142.55, 142.47, 121.29, 118.68, 112.19, 112.01, 111.36, 110.87, 110.80, 110.04, 105.13, 104.95, 55.13. MS (EI, m/z): 273 (M^+^).

This compound was known [49].

**86. 5-chloro-2-(4-methylphenyl) benzoxazole, CAS: 16715-75-6**

^1^H NMR (600 MHz, DMSO-d6) δ 8.07 (d, J = 8.2 Hz, 2H), 7.87 (s, 1H), 7.79 (d, J = 8.6 Hz, 1H), 7.48 – 7.38 (m, 3H), 2.40 (s, 3H). ^13^C NMR (151 MHz, DMSO-d6) δ 164.43, 149.43, 143.34, 143.16, 130.45, 129.46, 127.92, 125.78, 123.70, 119.82, 112.69, 21.66. MS (EI, m/z): 243 (M^+^).

This compound was known [50].

**87. 4-(1H-benzimidazol-2-yl)-2-methoxyphenol, CAS: 7404-01-5**

^1^H NMR (600 MHz, CD_3_OD) δ 7.66 (b, 1H), 7.51 (td, J = 2.8, 1.5 Hz, 3H), 7.16 (dd, J = 3.0, 1.6 Hz, 2H), 6.89 – 6.87 (m, 1H), 3.91(m, 3H). ^13^C NMR (151 MHz, CD_3_OD) δ 152.68, 149.70, 148.36, 148.05, 122.10, 120.54, 119.88, 115.50, 109.83, 55.03. MS (EI, m/z): 240 (M^+^).

This compound was known [51].

**88. Consistent with Product 53**

**89. 2-(4-methoxyphenyl)-1-[(4-methoxyphenyl) methyl]-1H-benzimidazole, CAS: 2620-83-9**

^1^H NMR (600 MHz, CD_3_OD) δ 7.66 (d, J = 7.9 Hz, 1H), 7.57 (dt, J = 8.8, 2.9 Hz, 2H), 7.32 (d, J = 7.4 Hz, 1H), 7.23 (dt, J = 19.7, 7.3 Hz, 2H), 7.02 (dd, J = 8.6, 4.6 Hz, 2H), 6.92 (t, J = 6.7 Hz, 2H), 6.80 (p, J = 4.3, 3.7 Hz, 2H), 5.40 (d, J = 12.2 Hz, 2H), 3.87 – 3.77 (m, 3H), 3.70 (t, J = 3.5 Hz, 3H). ^13^C NMR (151 MHz, CD_3_OD) δ 161.43, 159.33, 154.06, 141.98, 135.56, 130.50, 128.43, 127.13, 122.77, 122.50, 121.62, 118.18, 113.97, 113.93, 110.73, 54.53, 54.30. MS (EI, m/z): 344 (M^+^).

This compound was known [52].


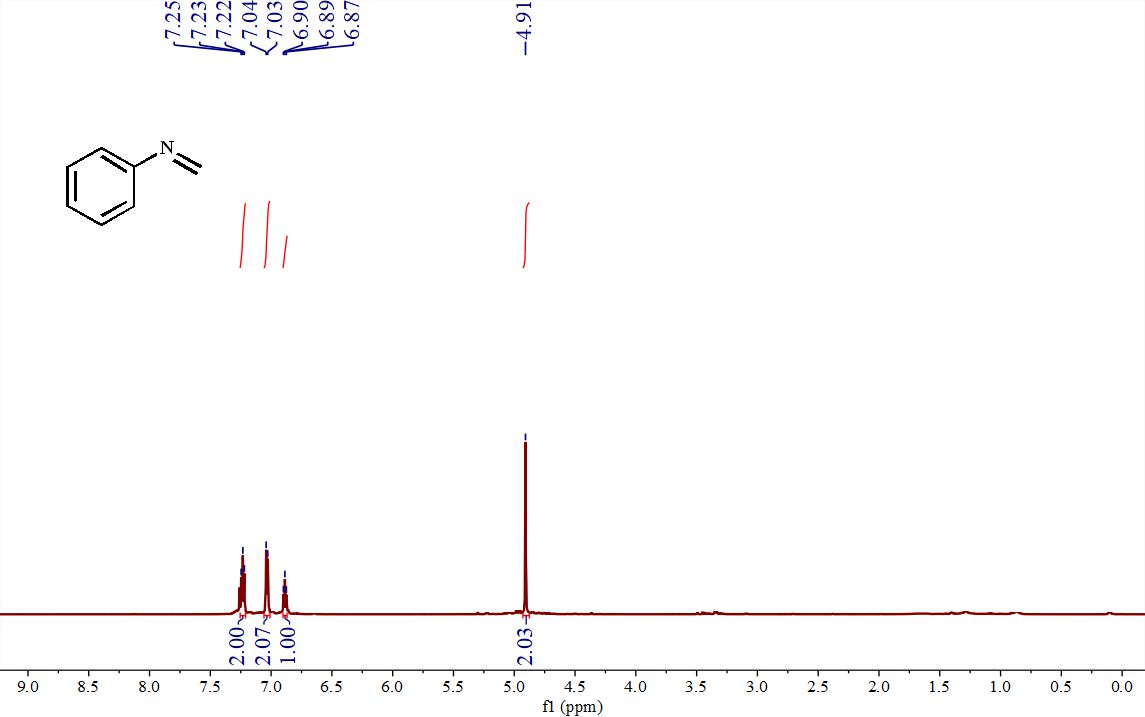


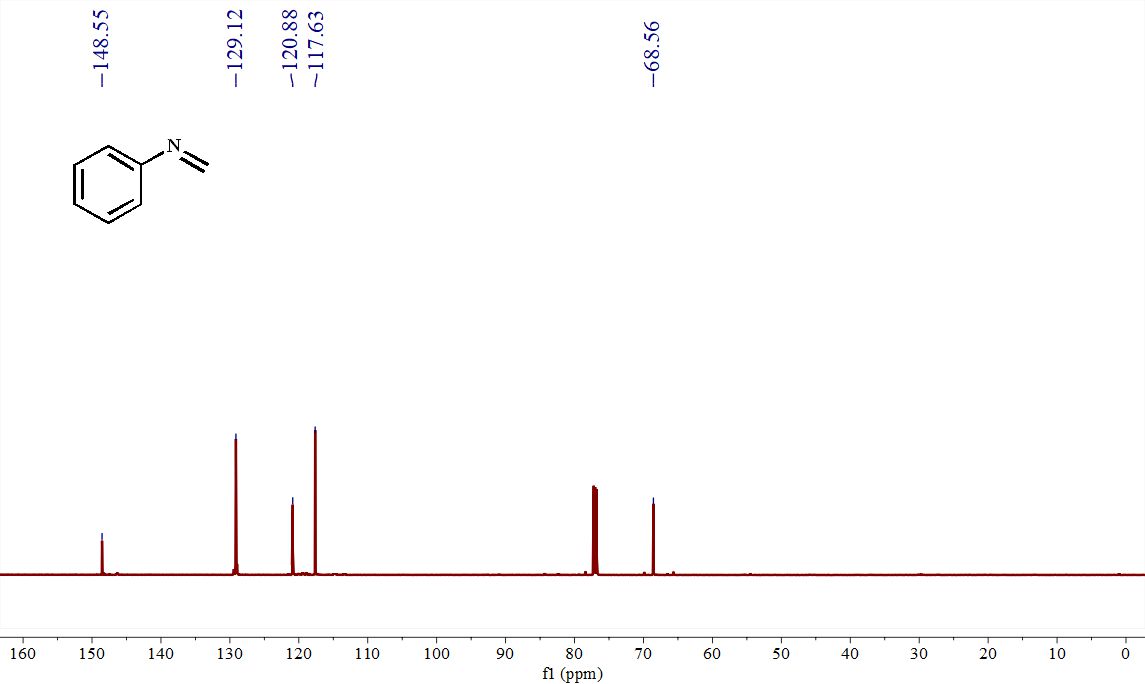


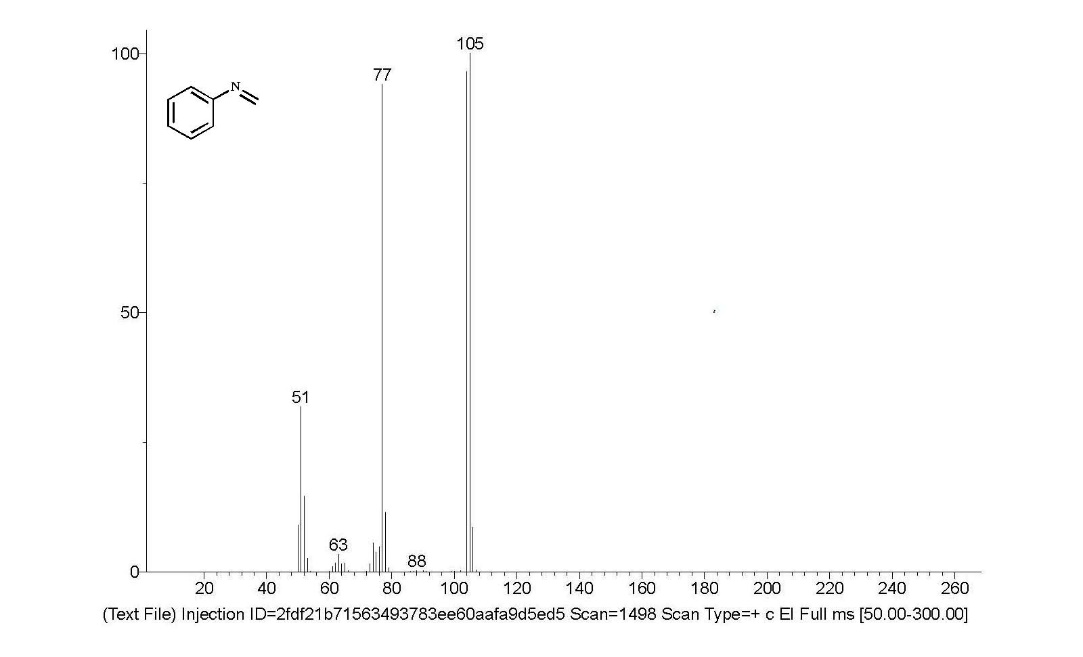


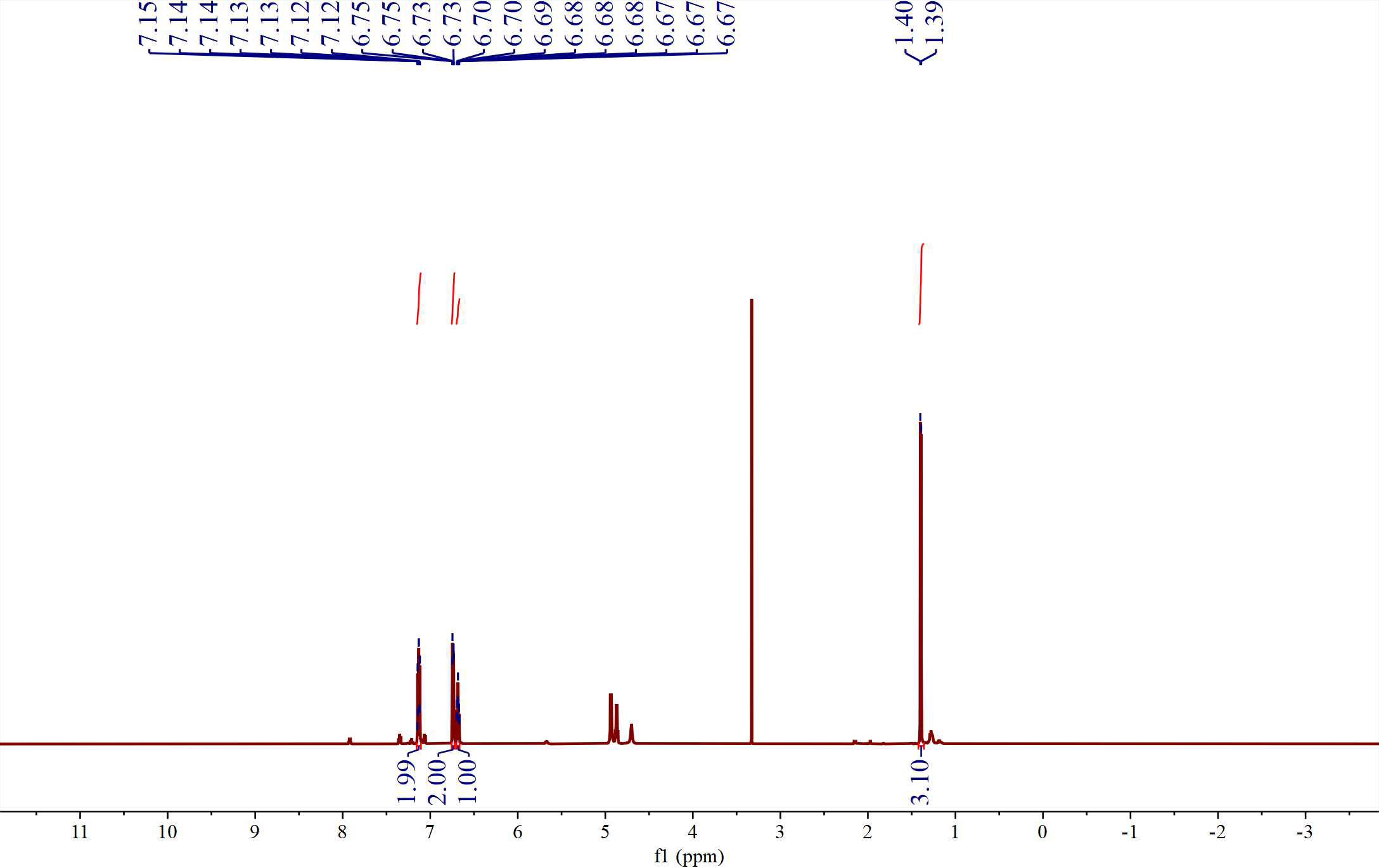


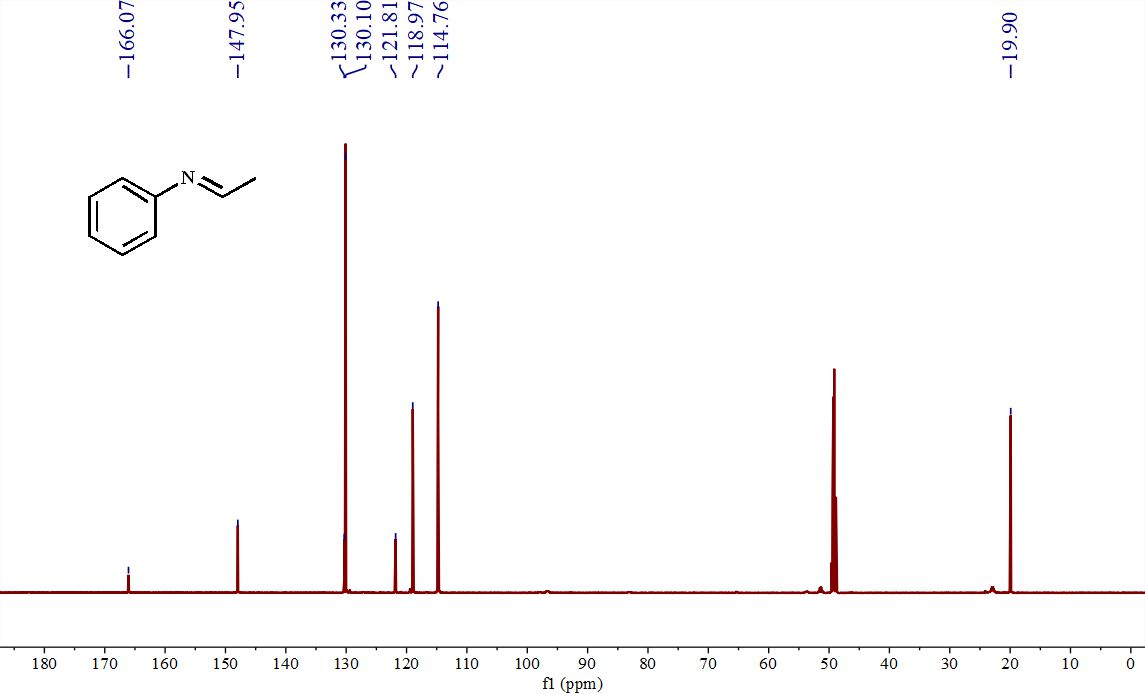


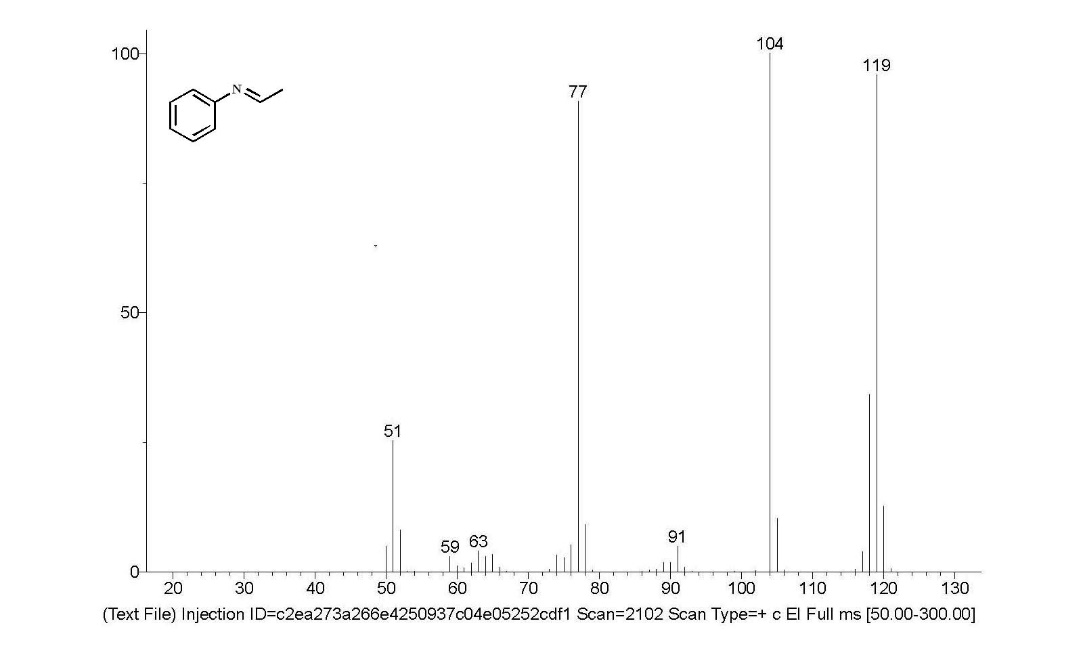


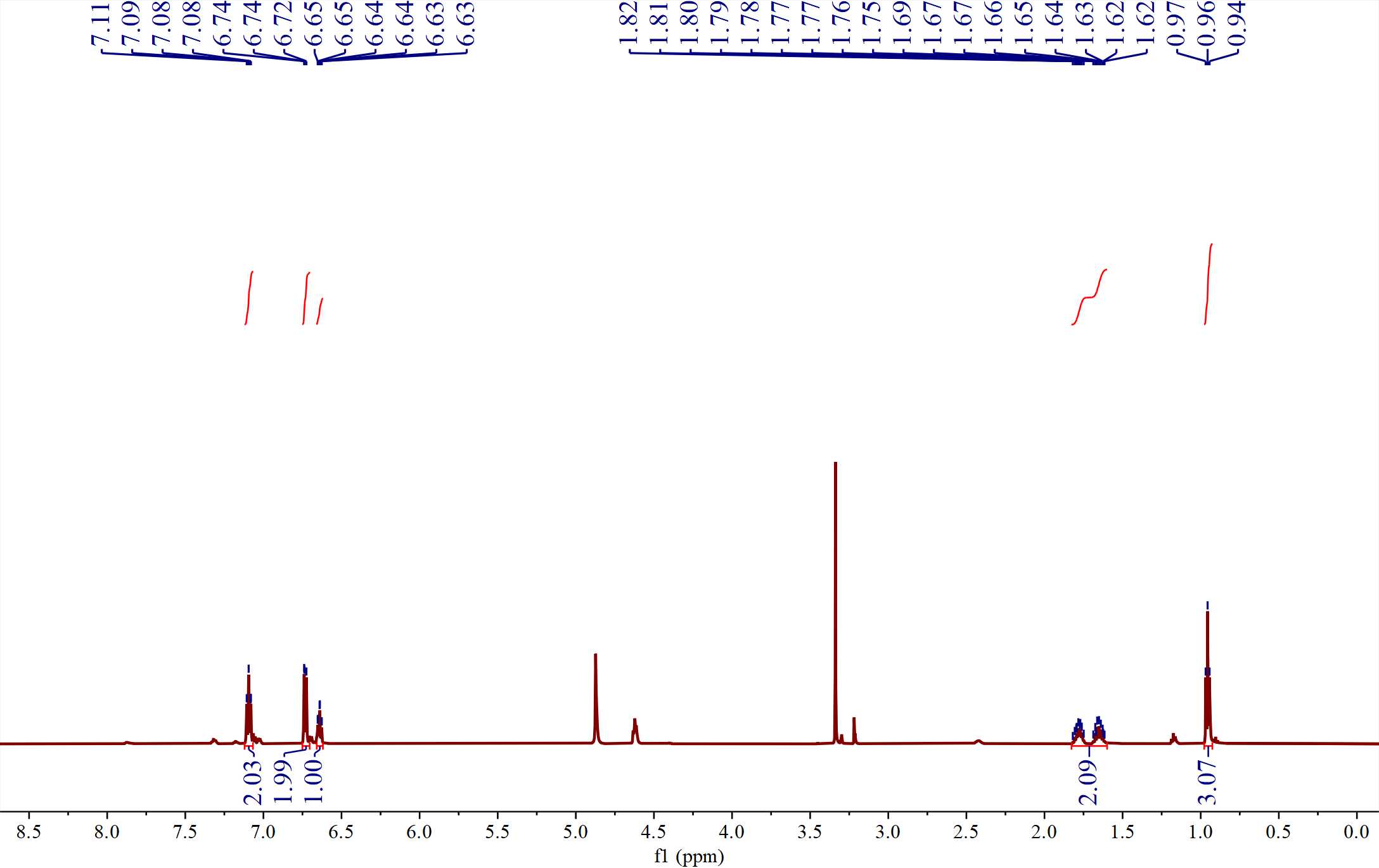


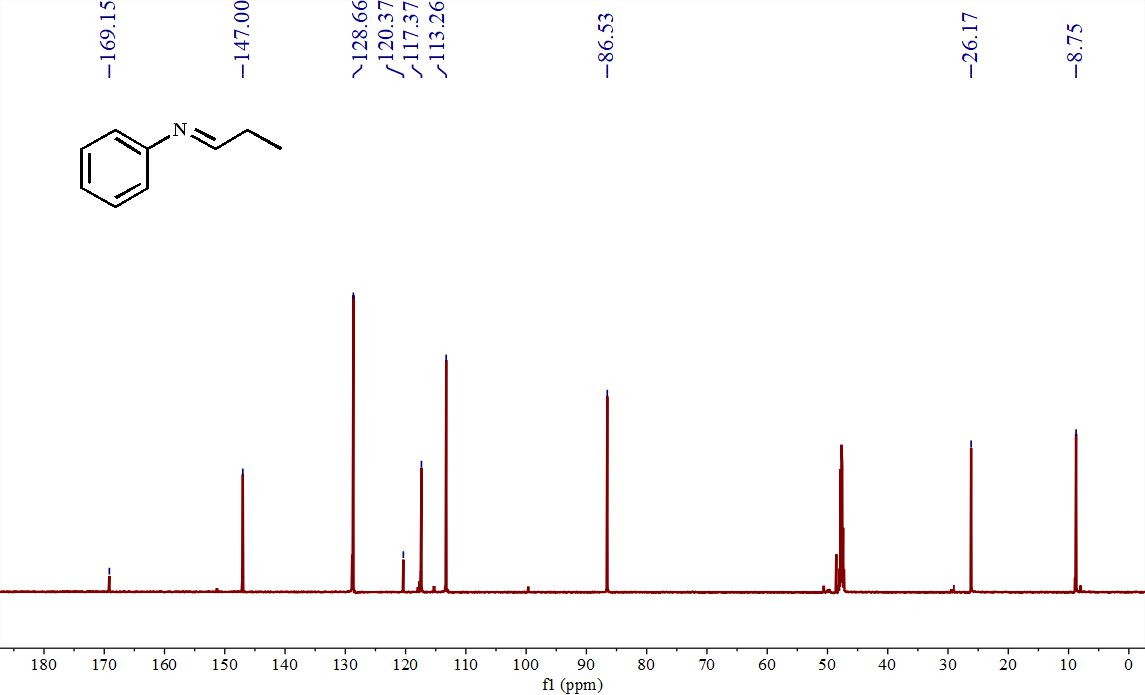


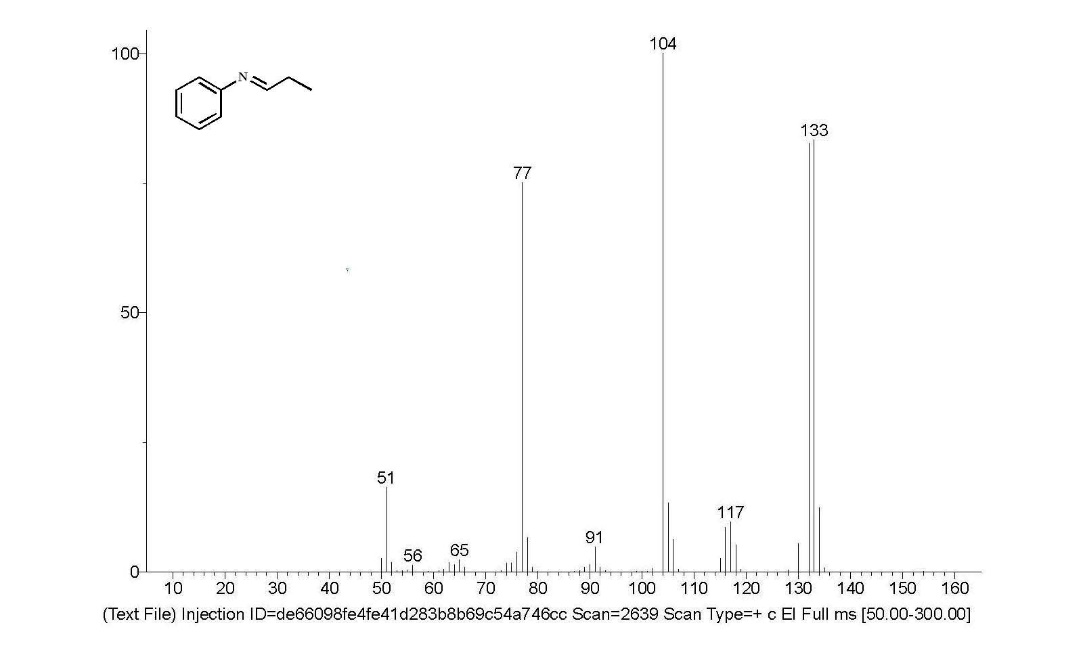


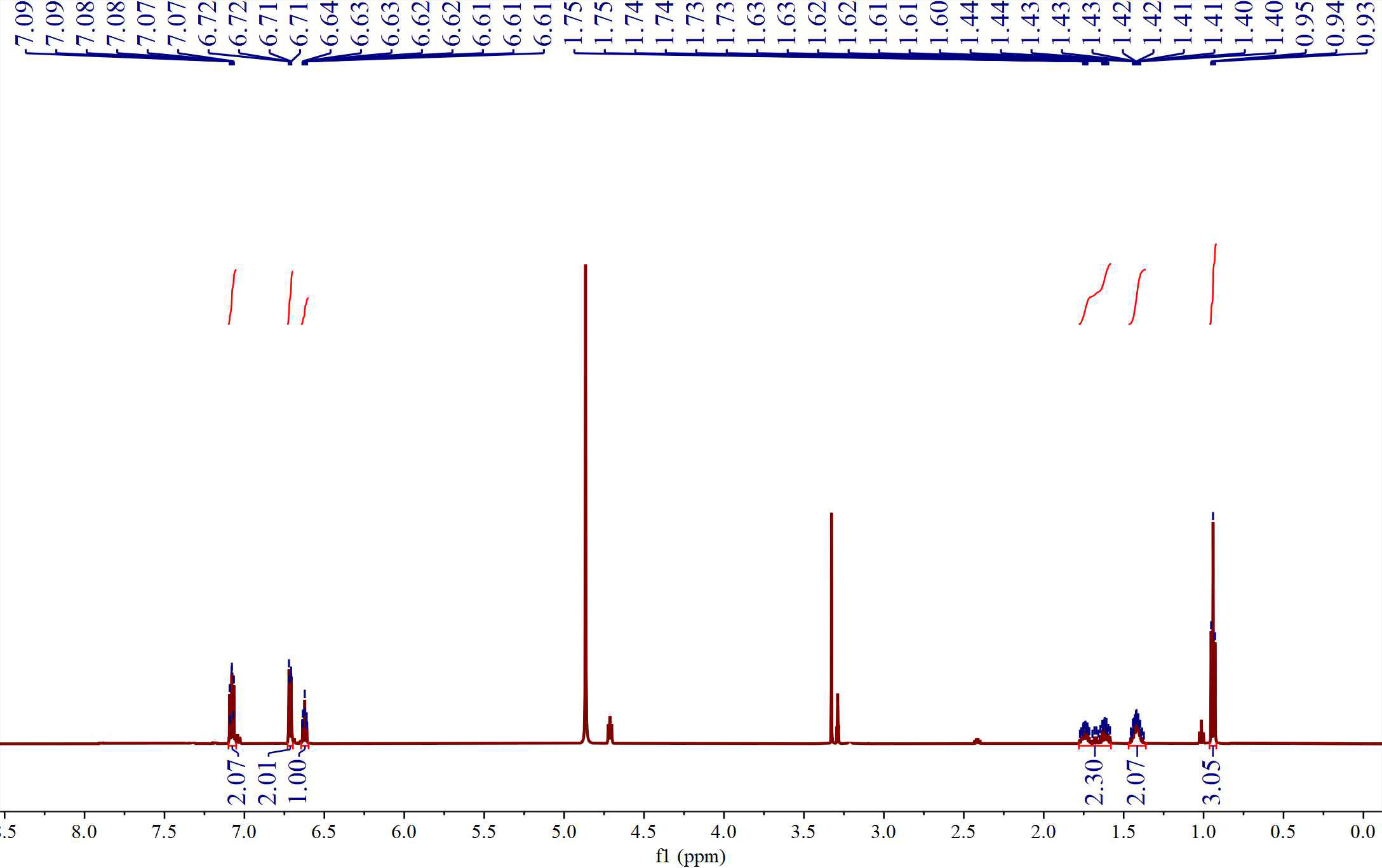


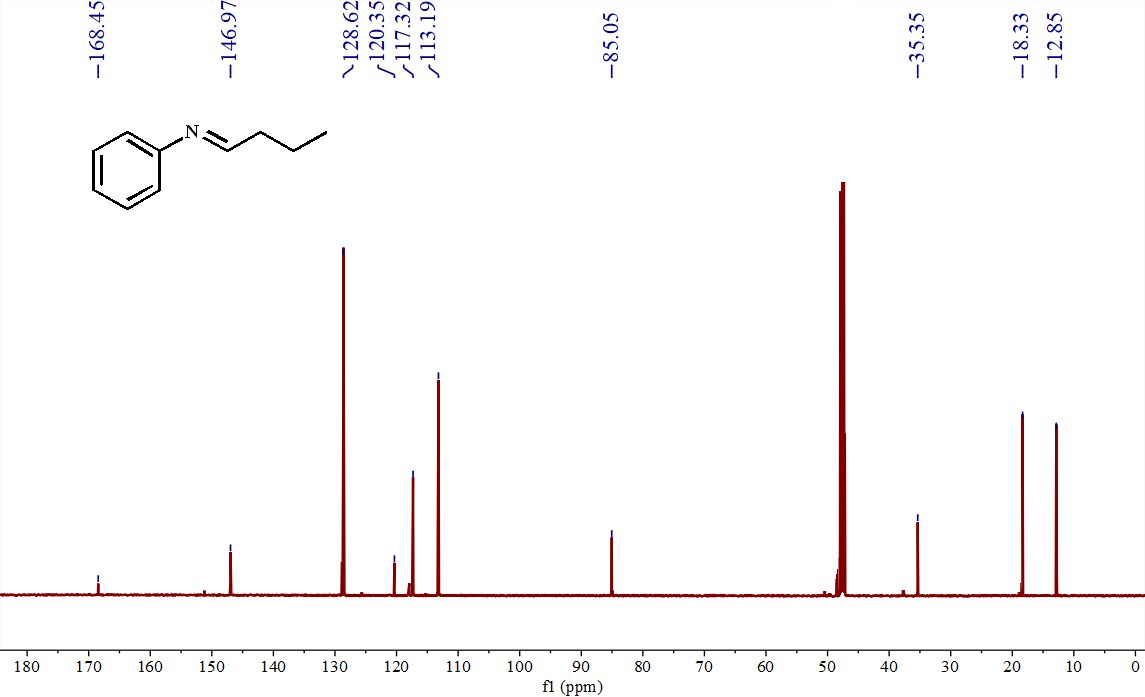


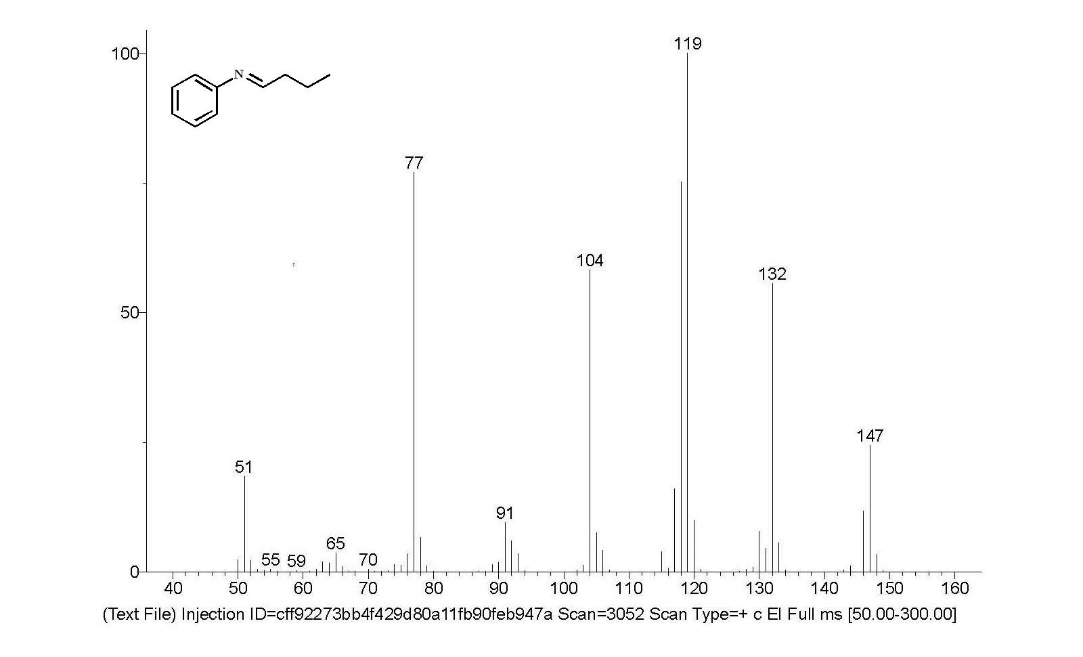


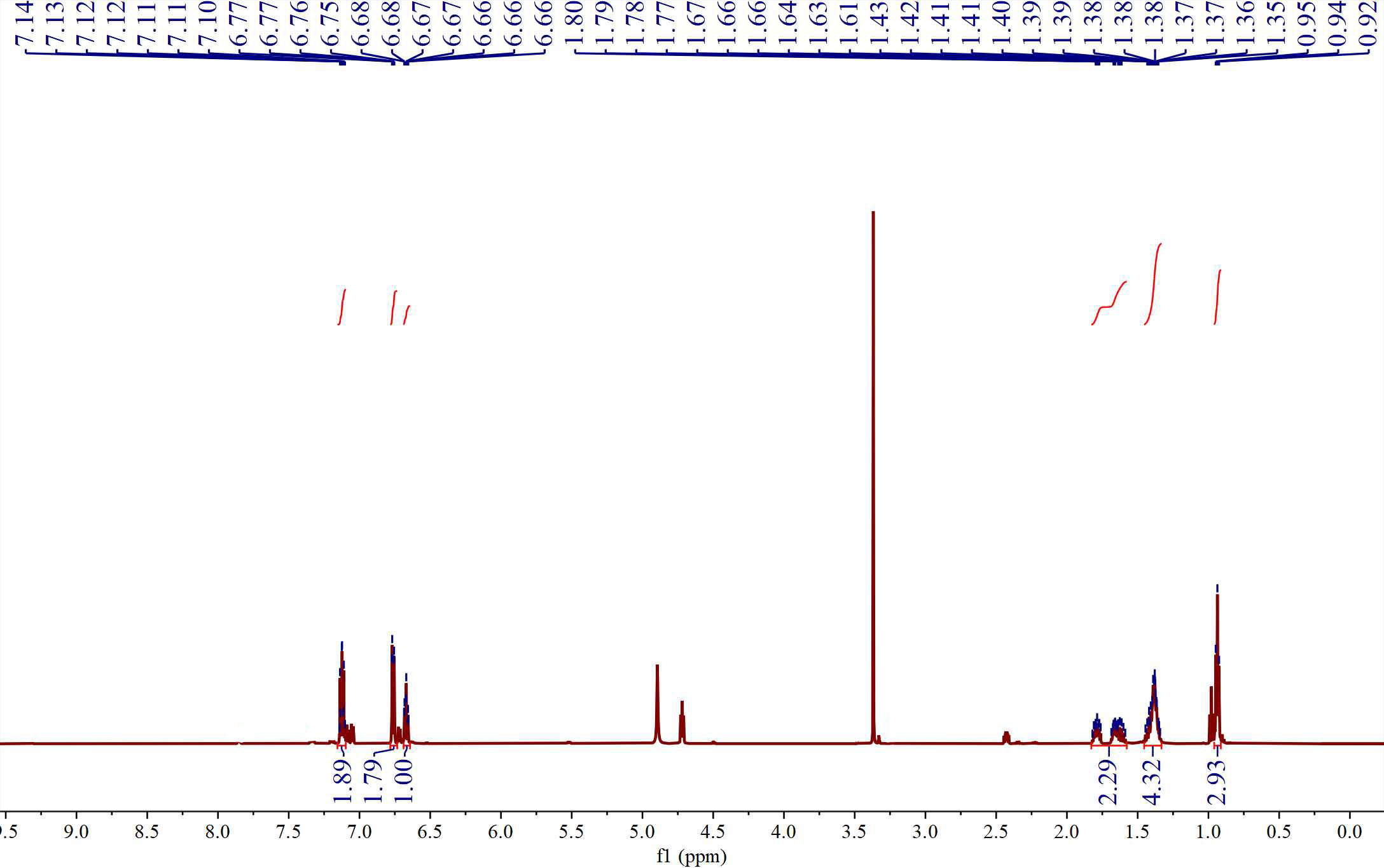


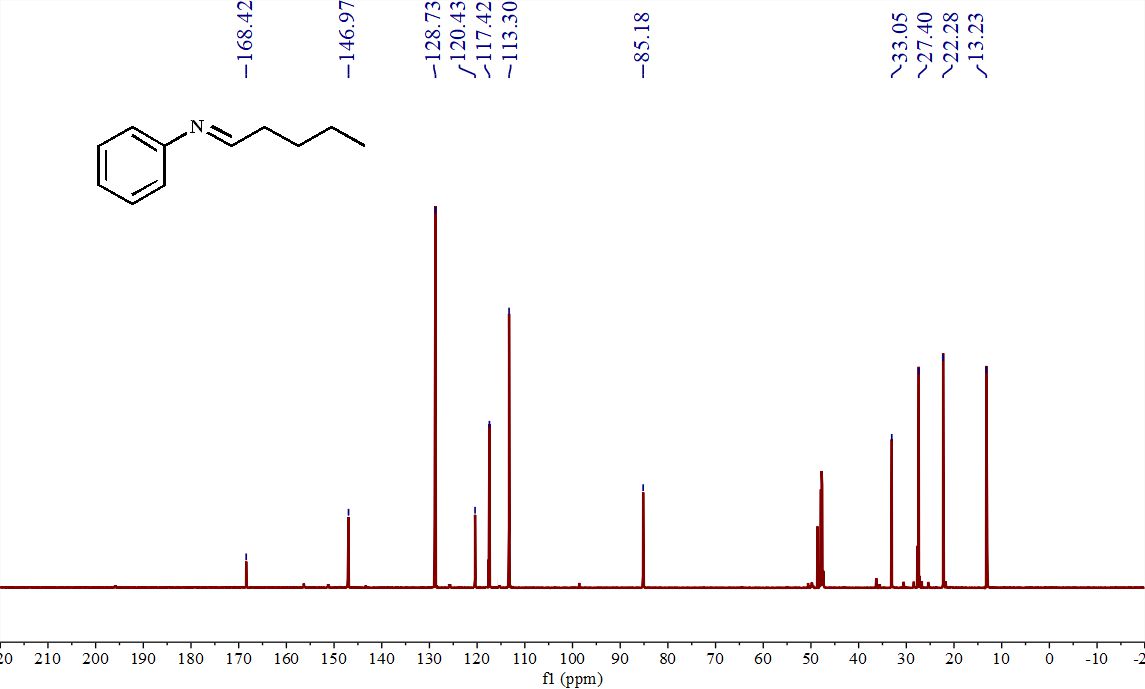


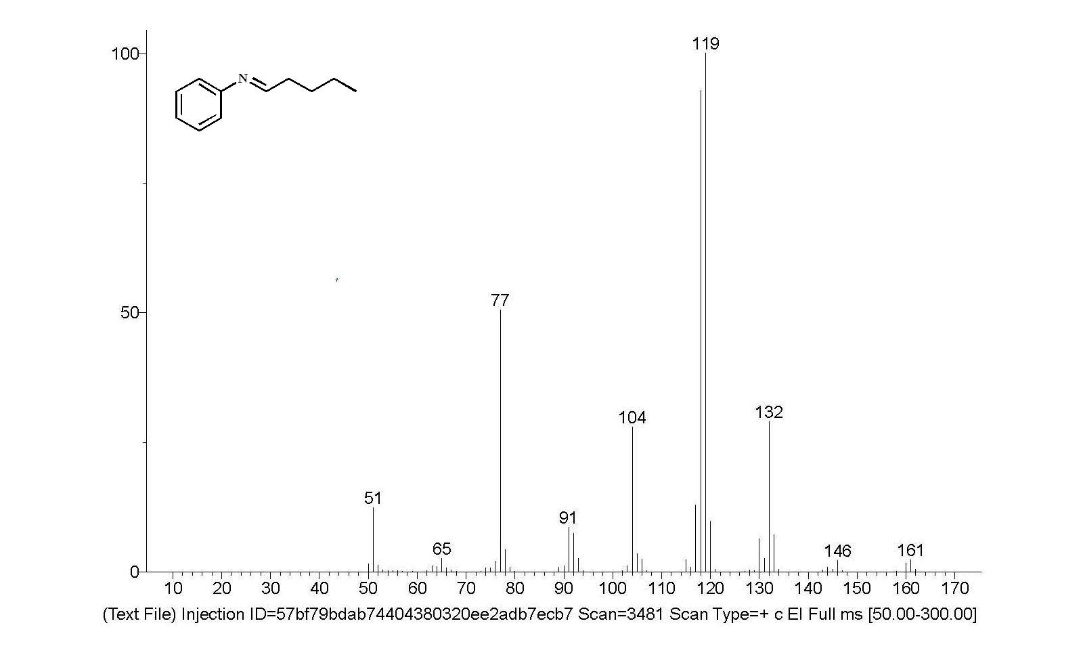


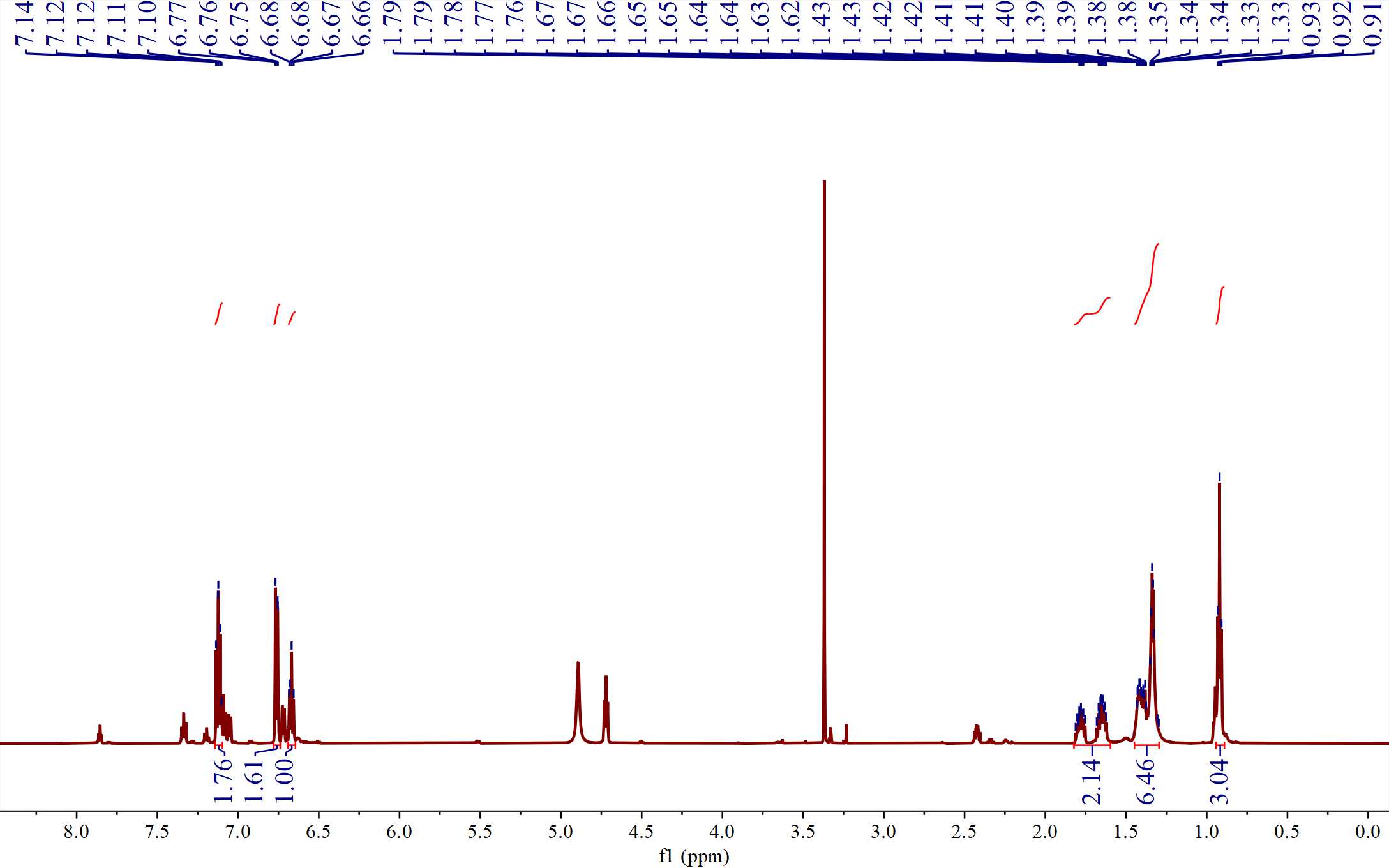


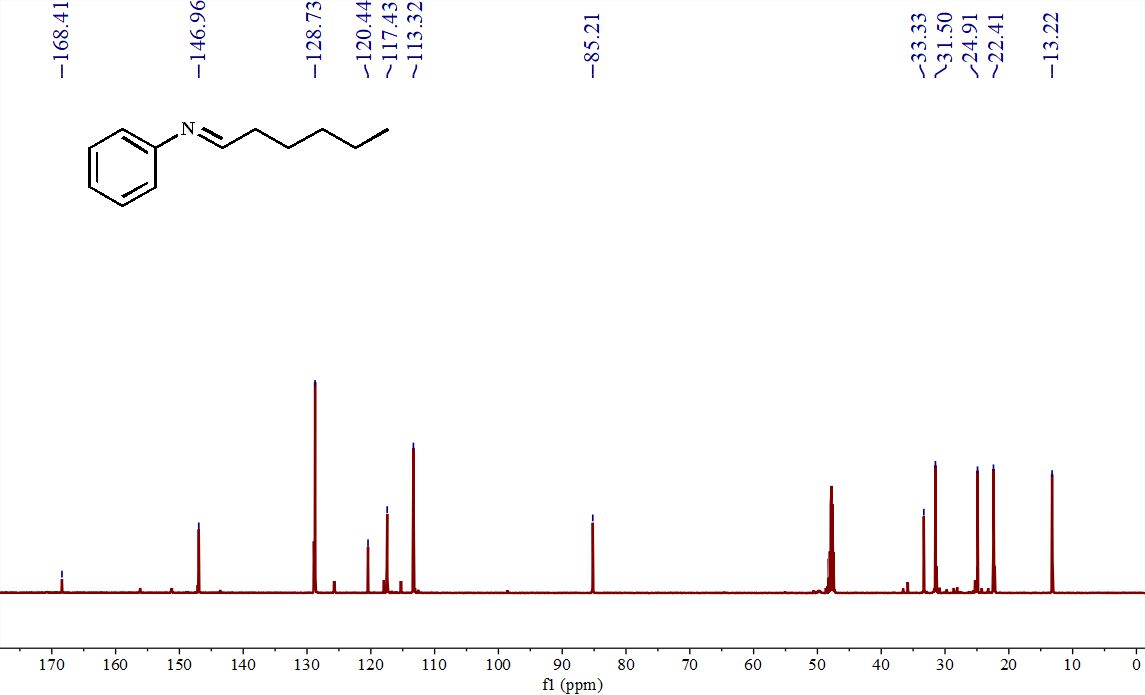


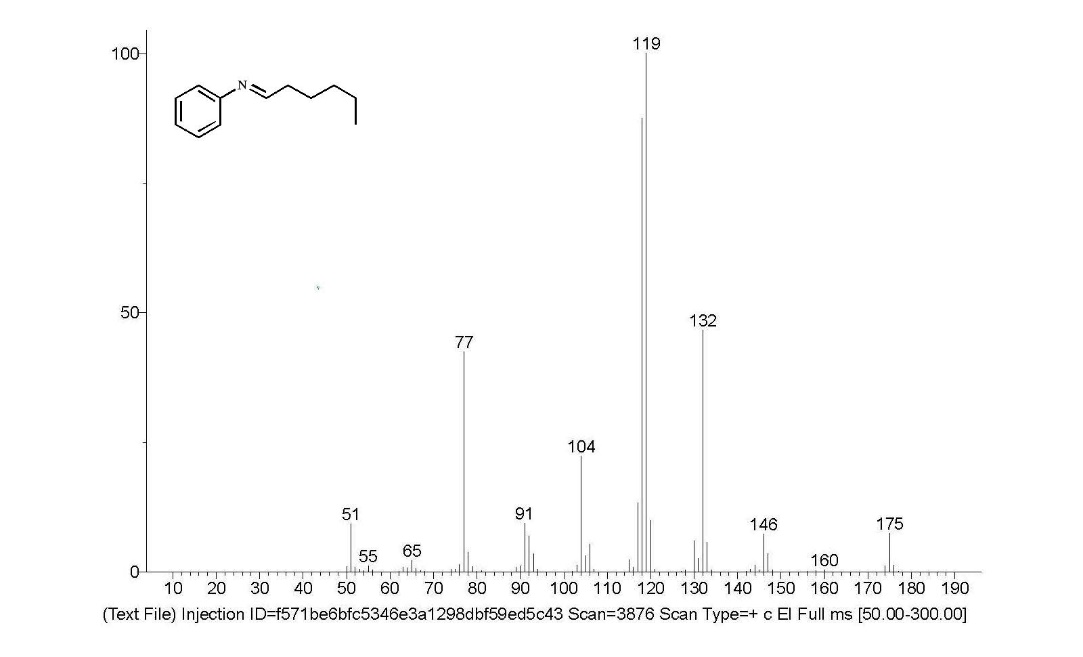


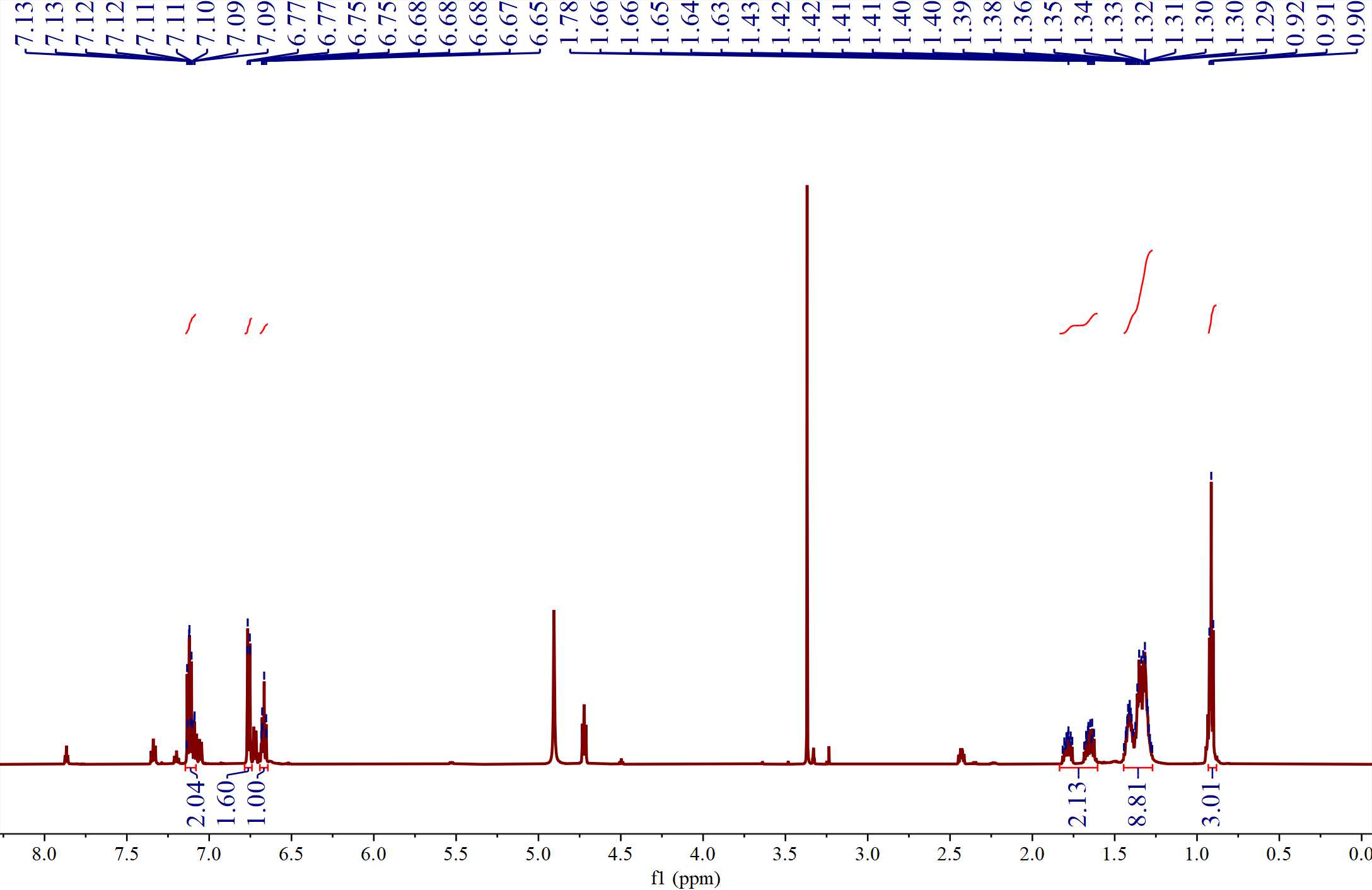


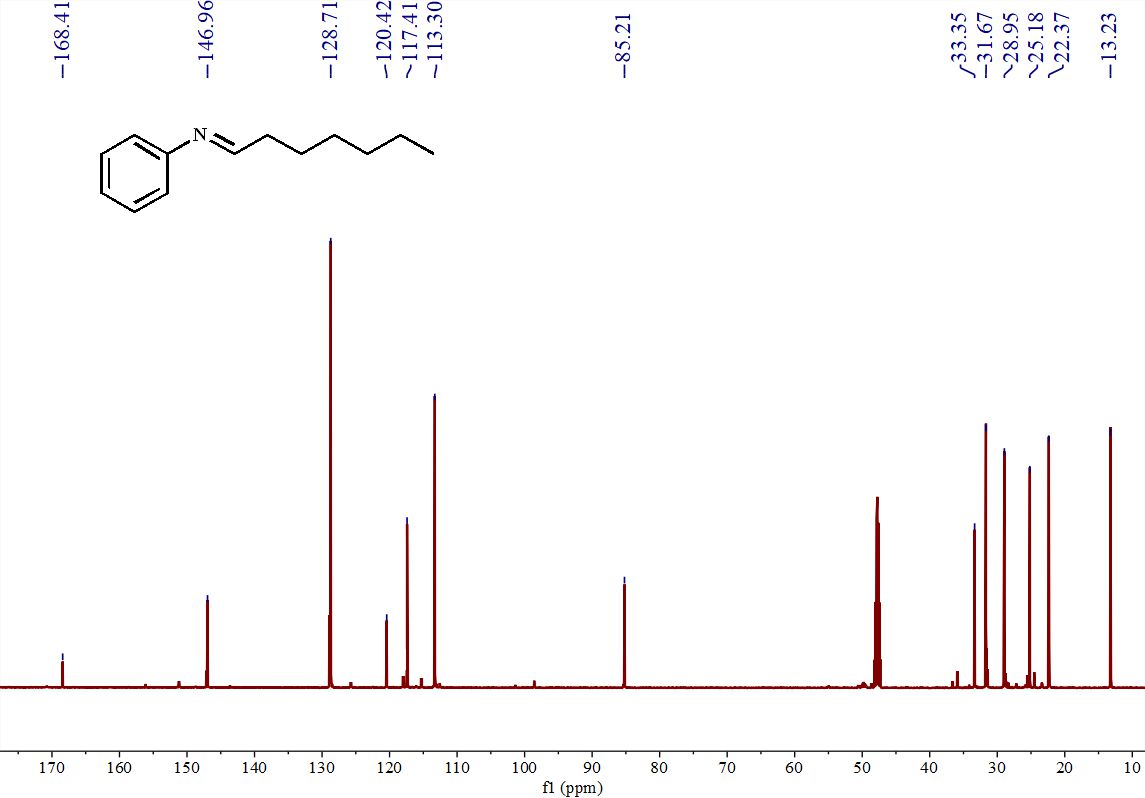


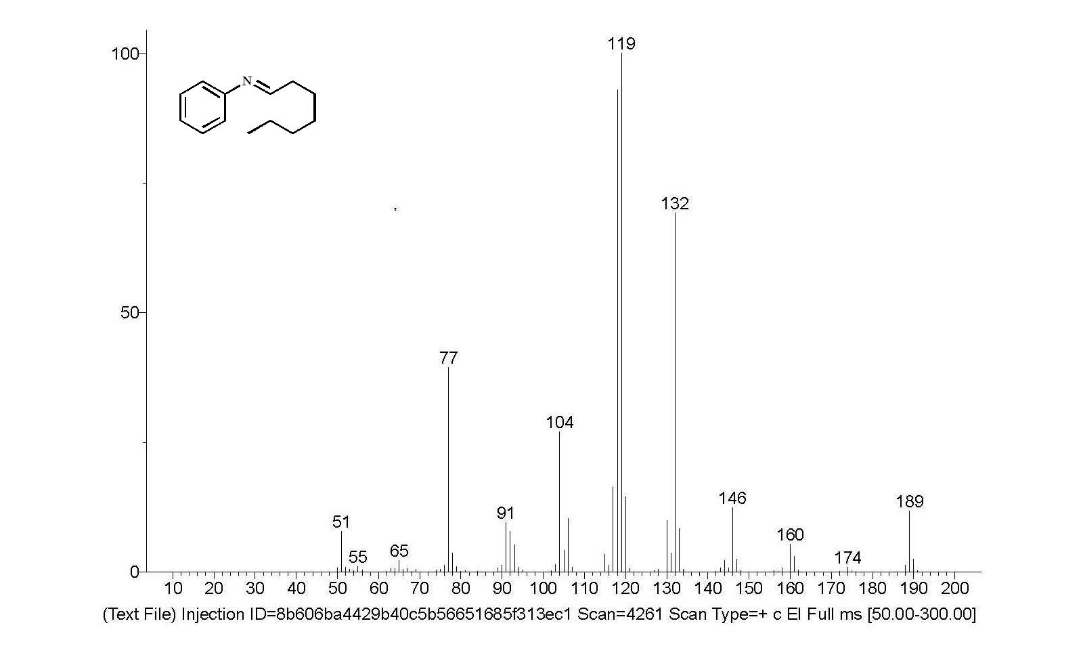


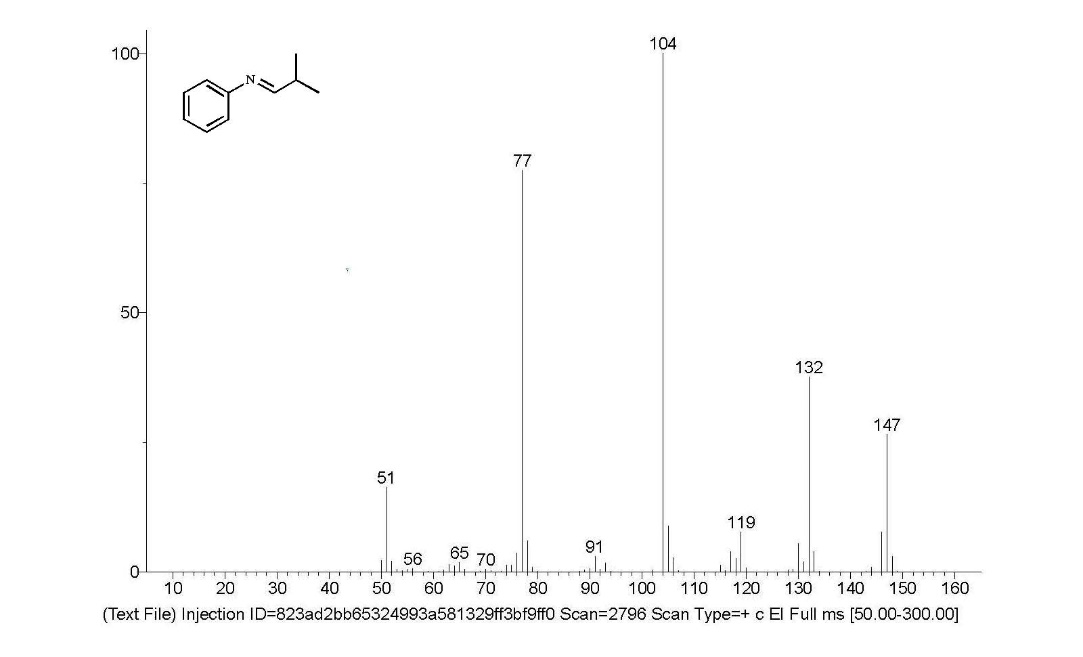


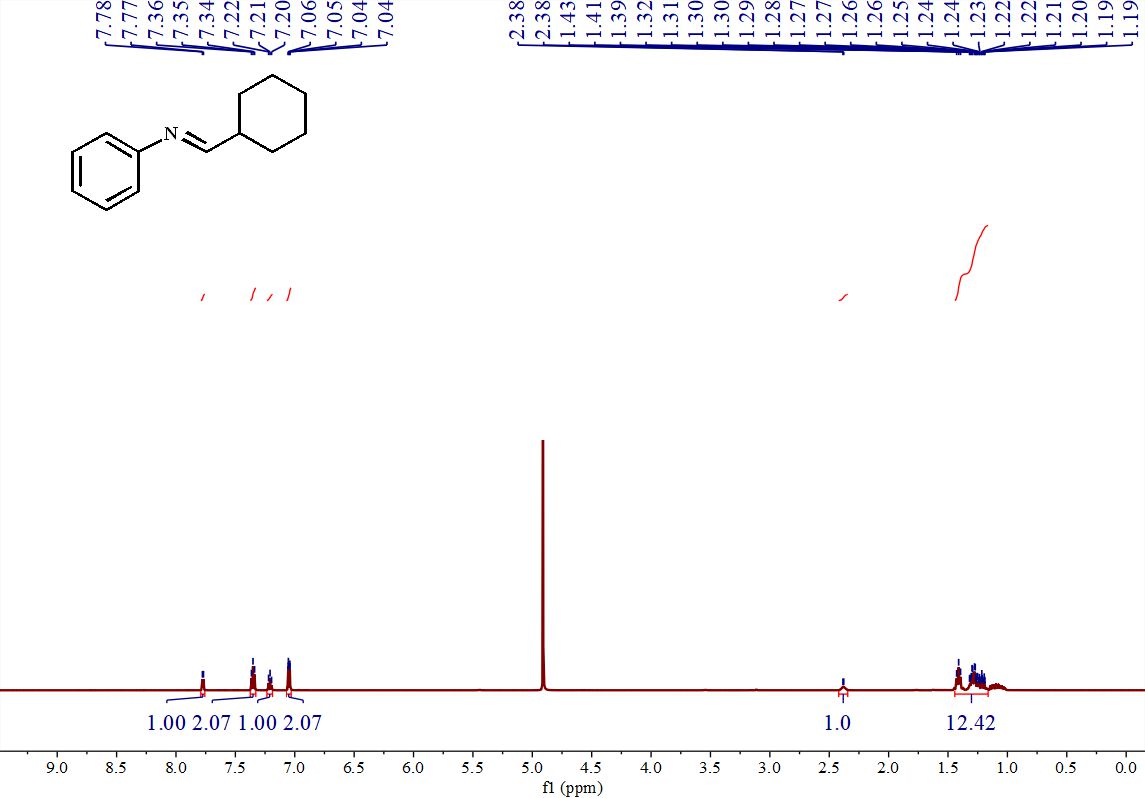


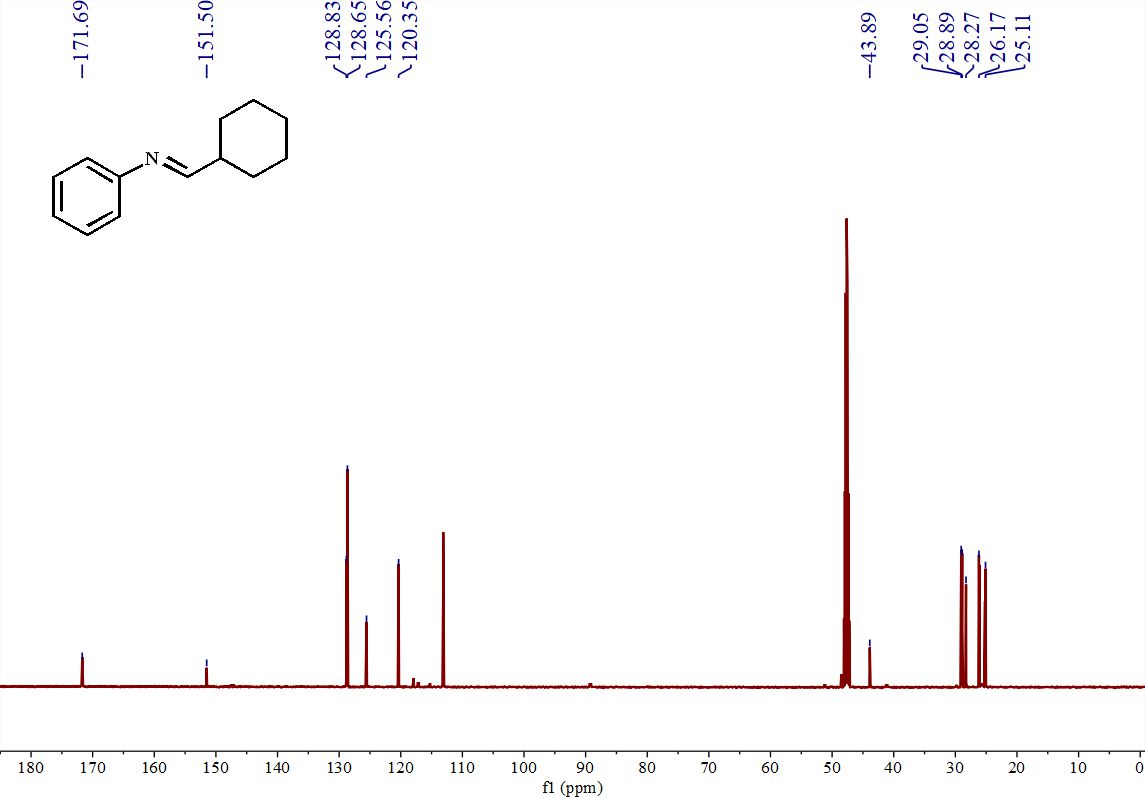


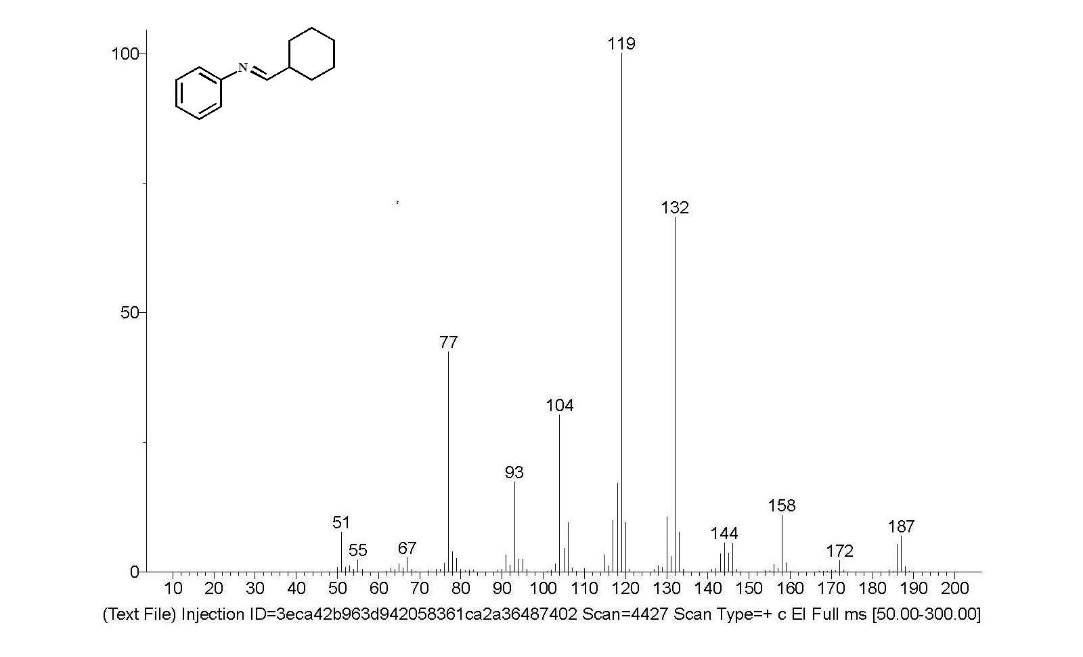


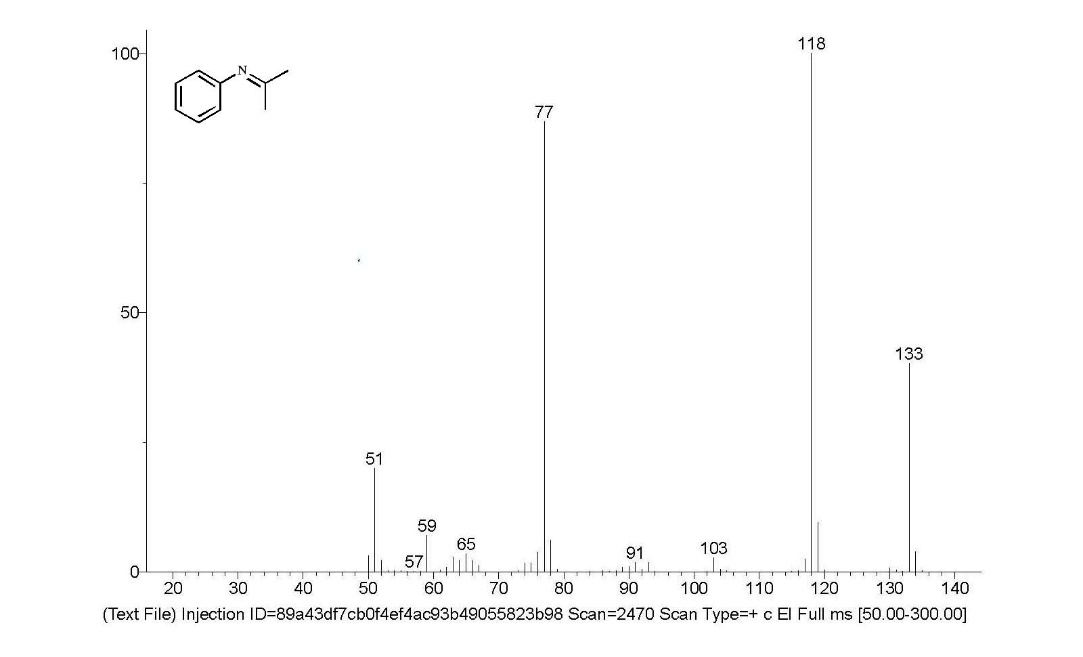


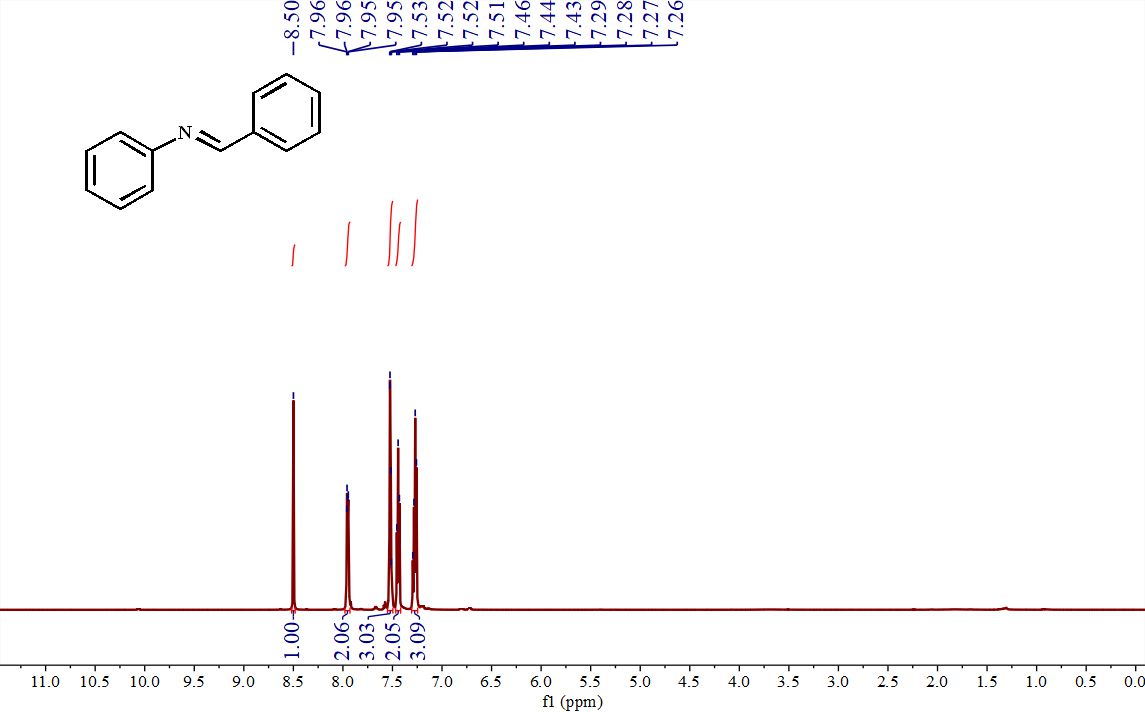


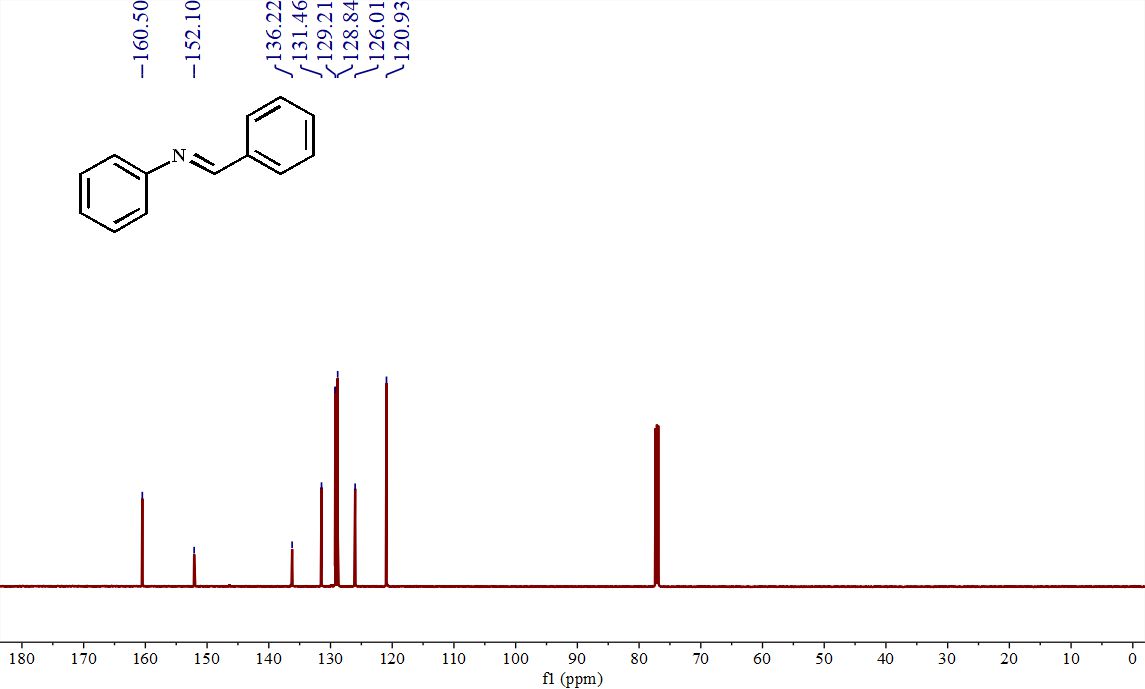


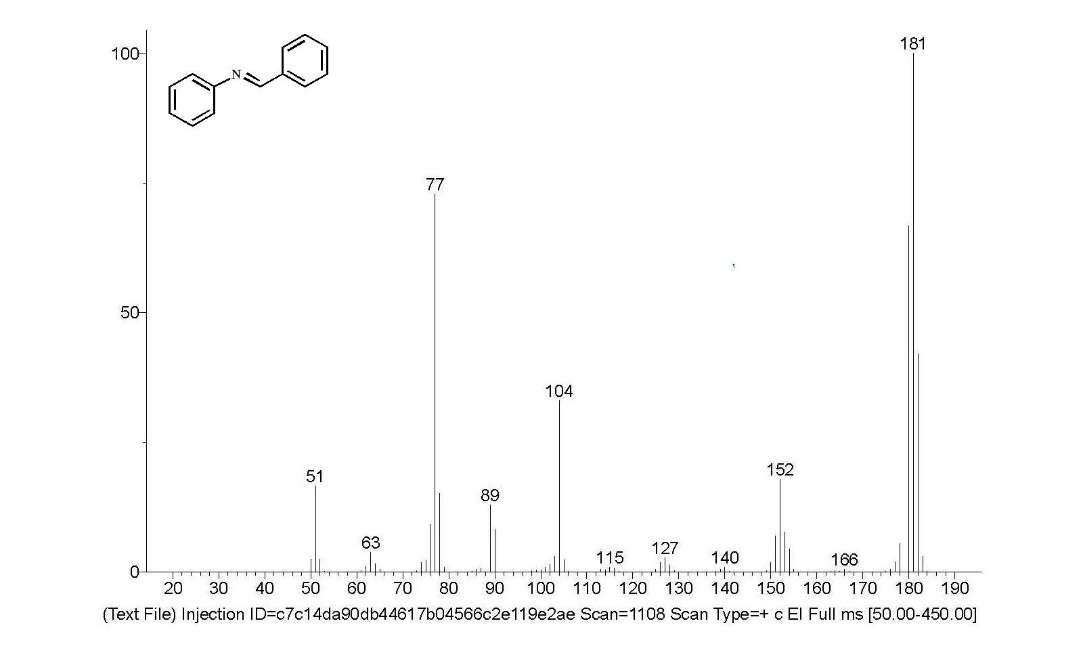


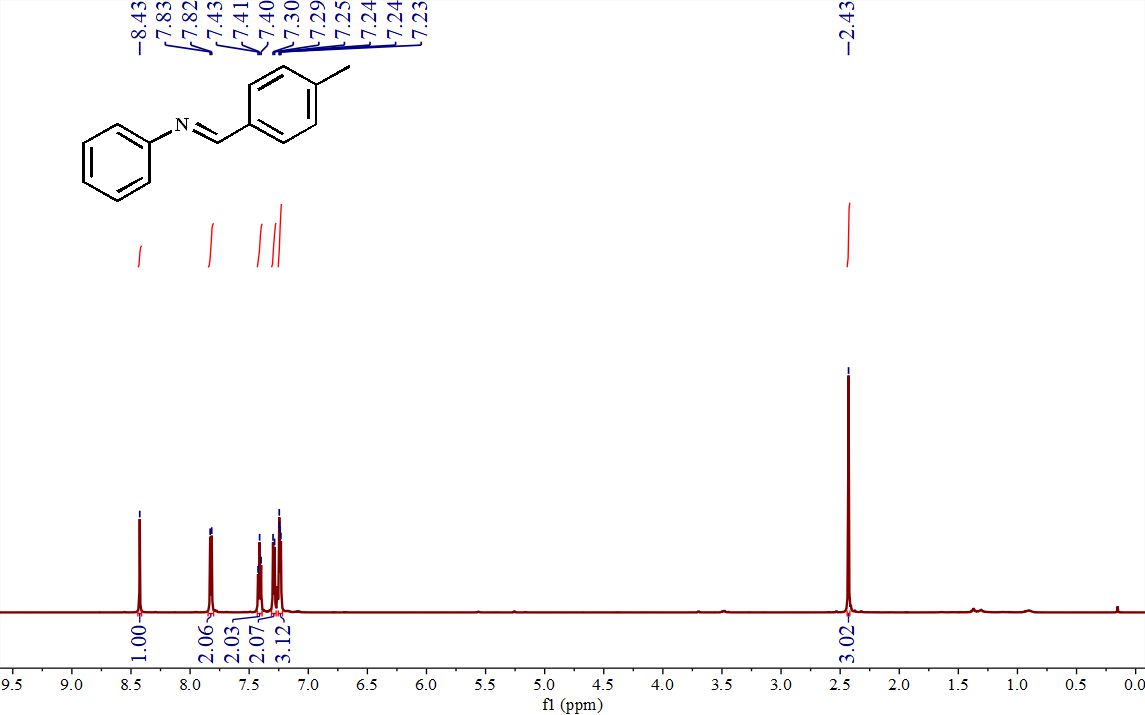


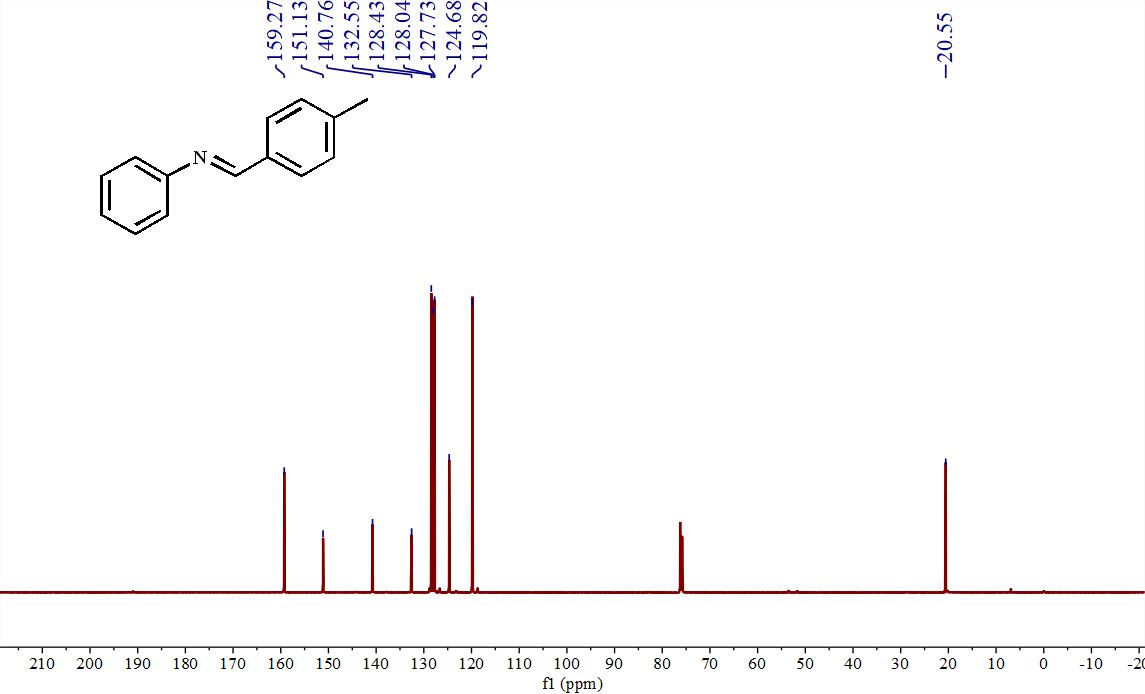


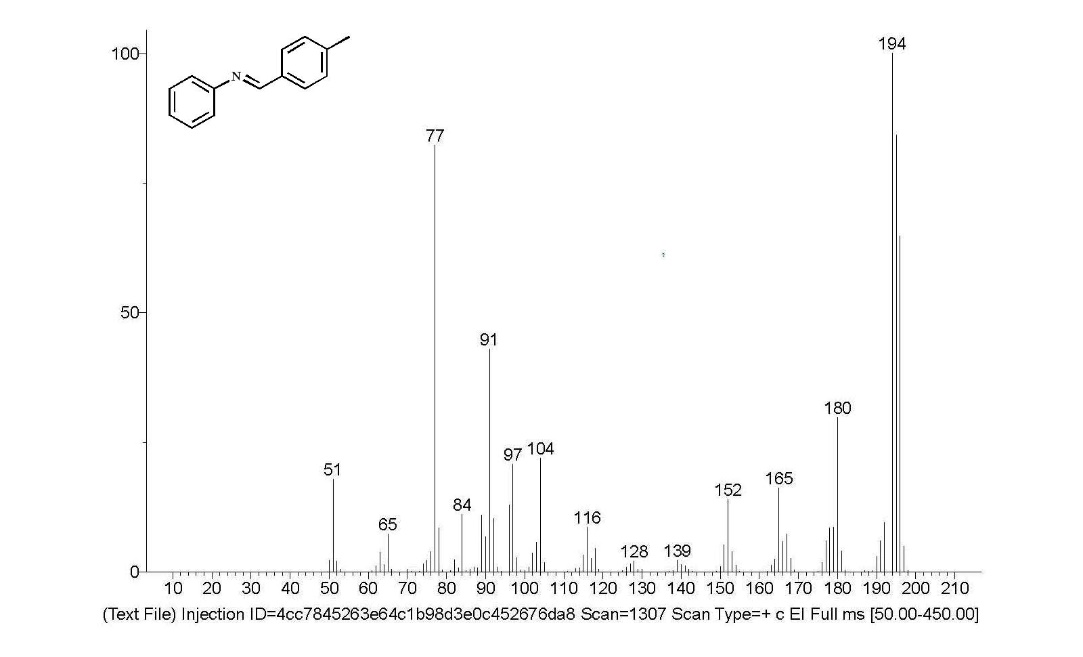


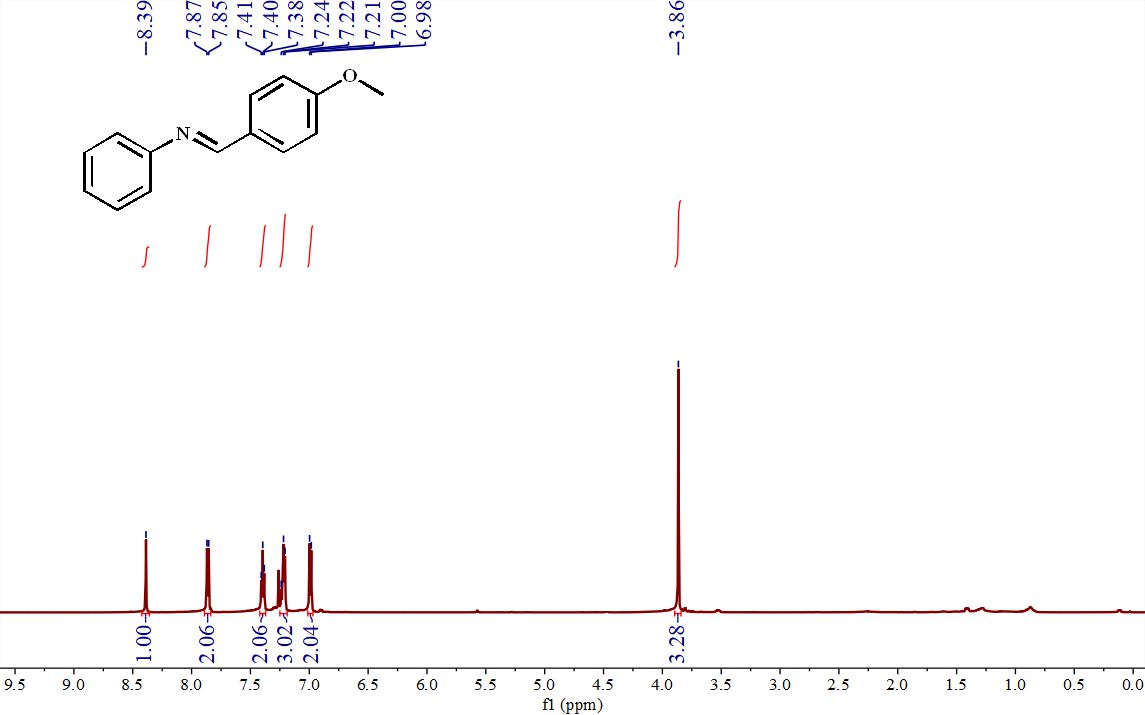


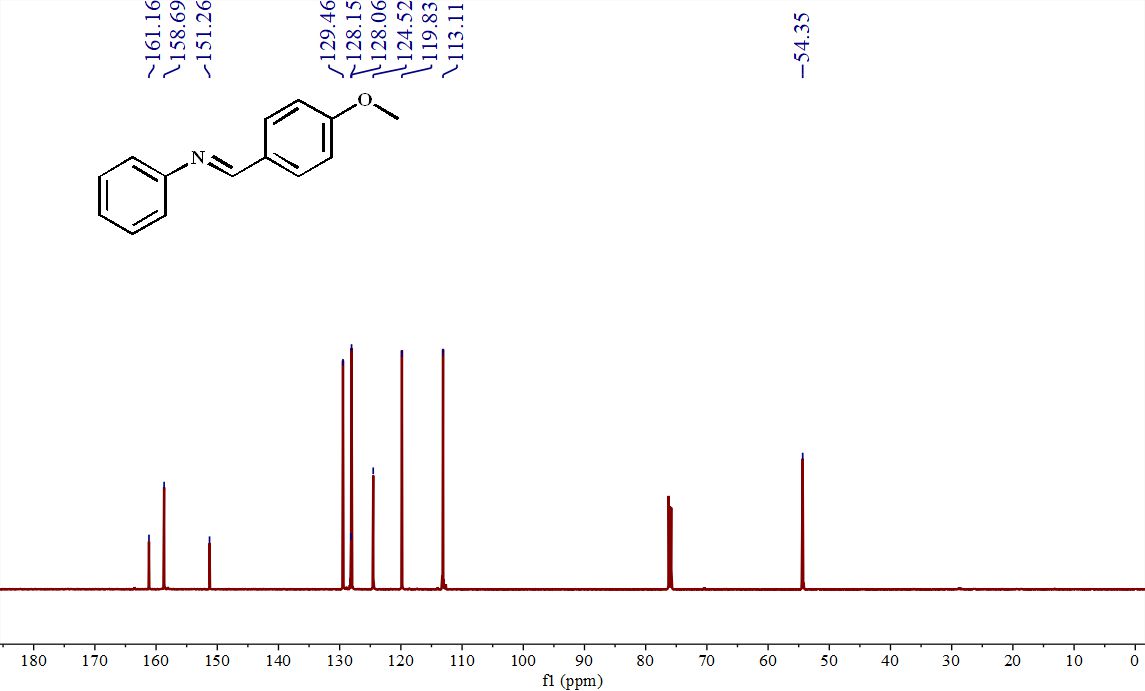


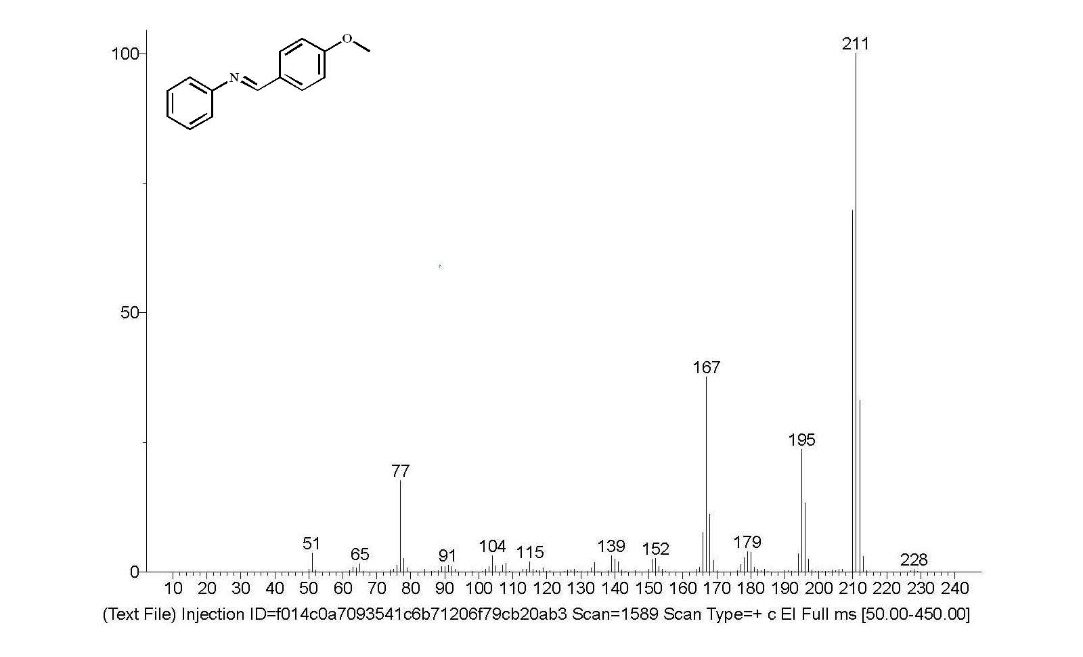


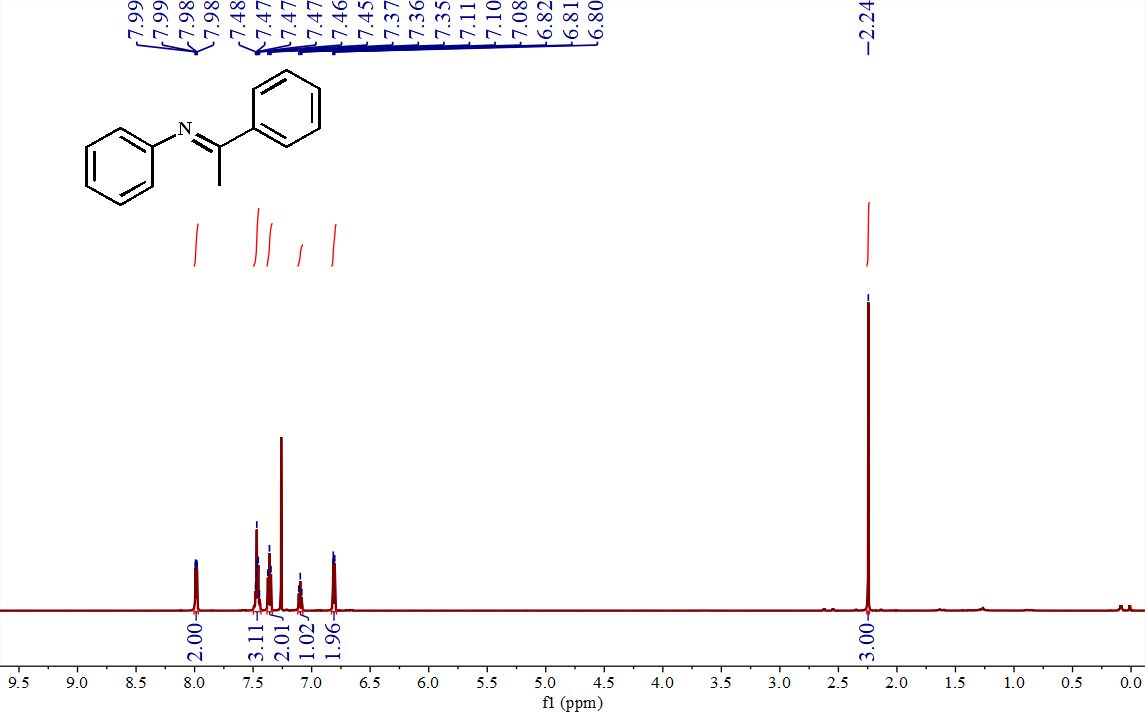

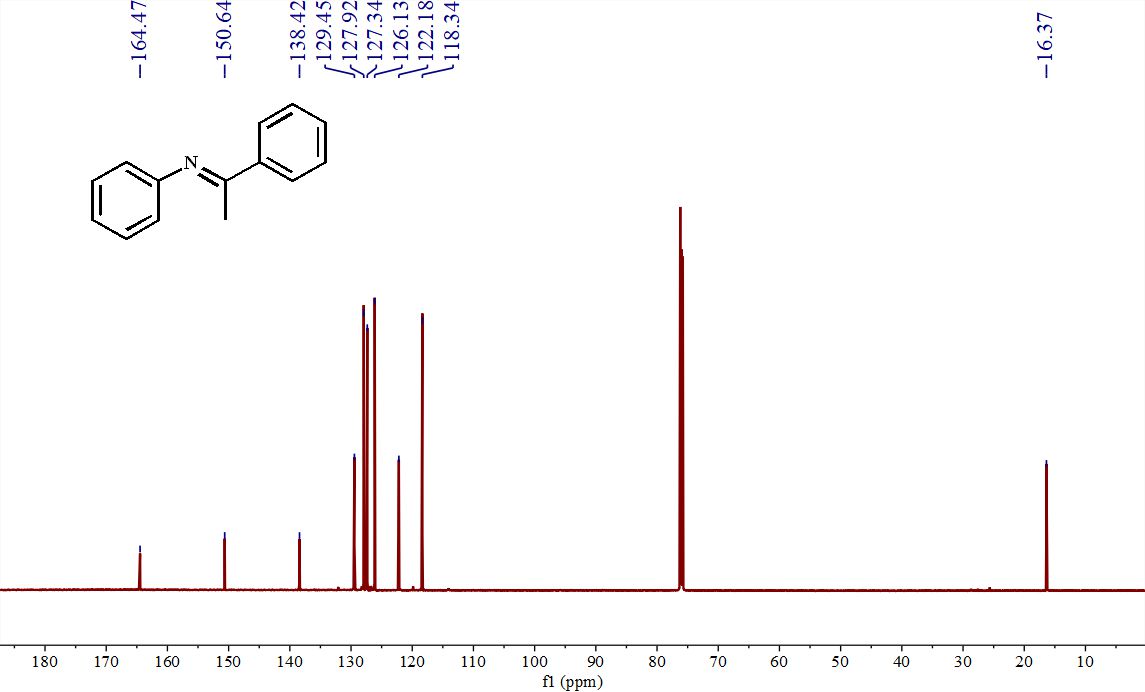

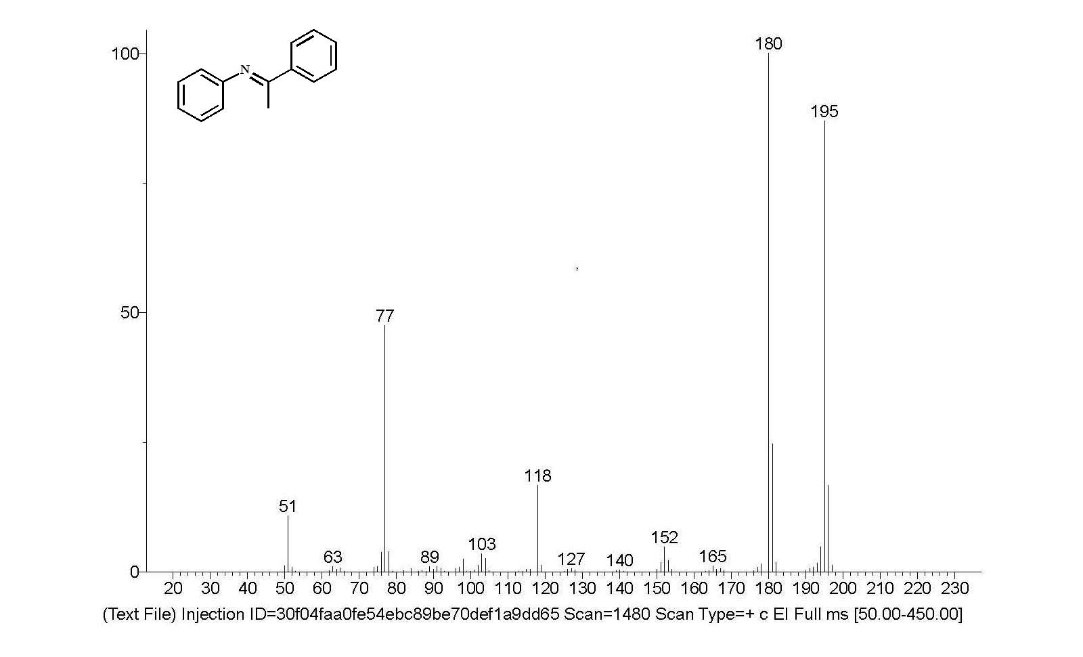


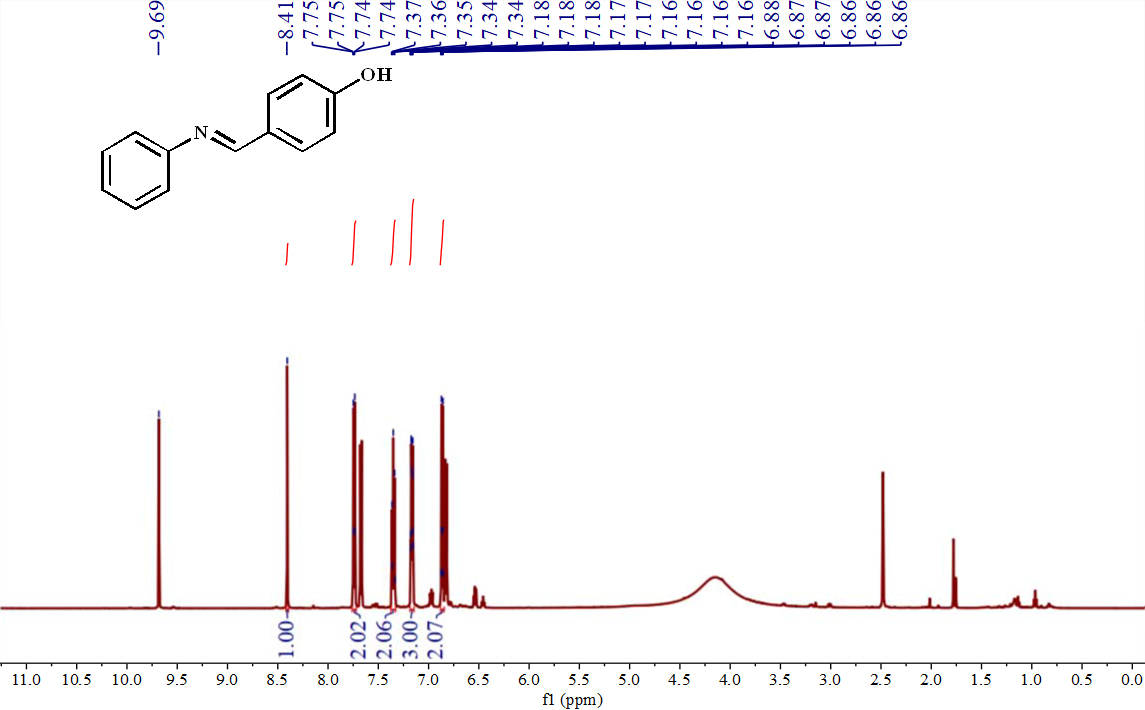


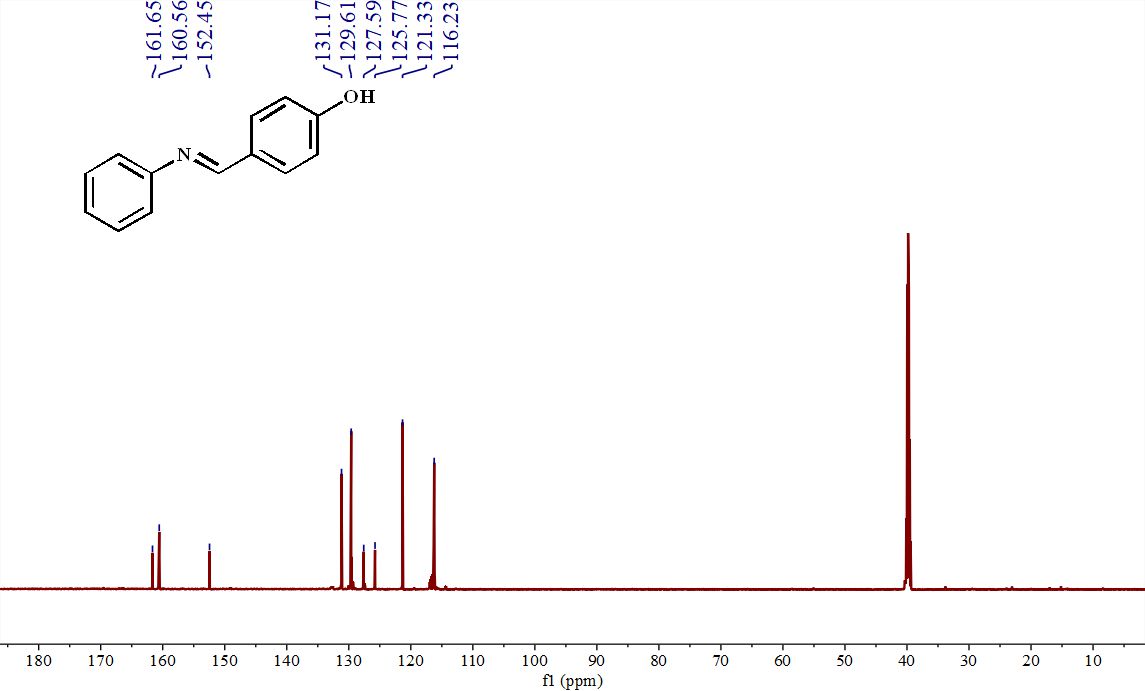


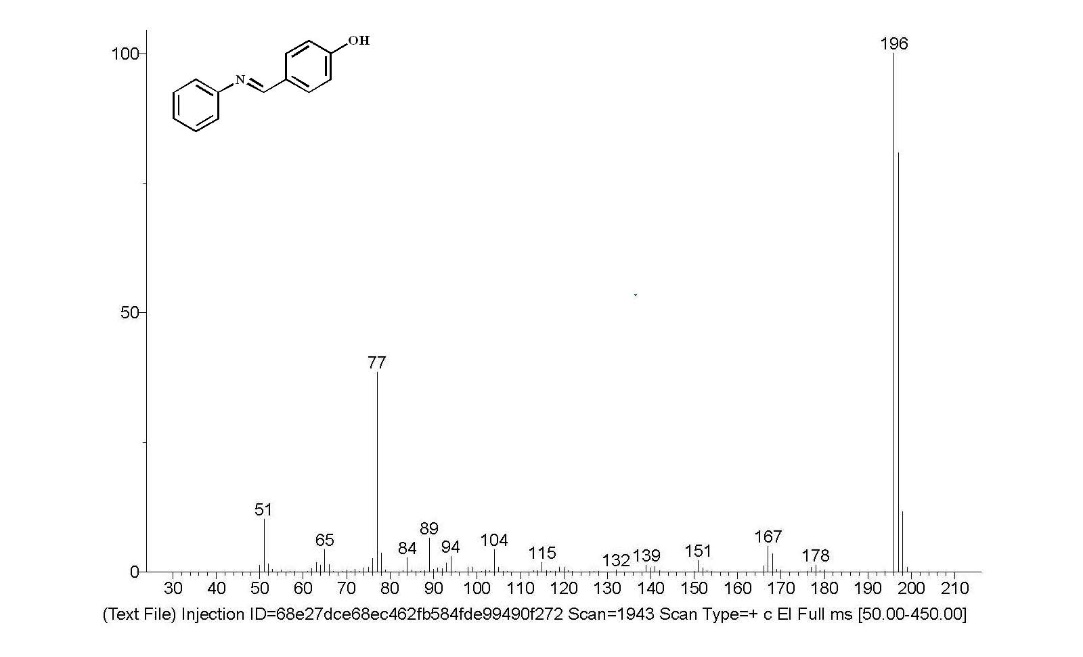


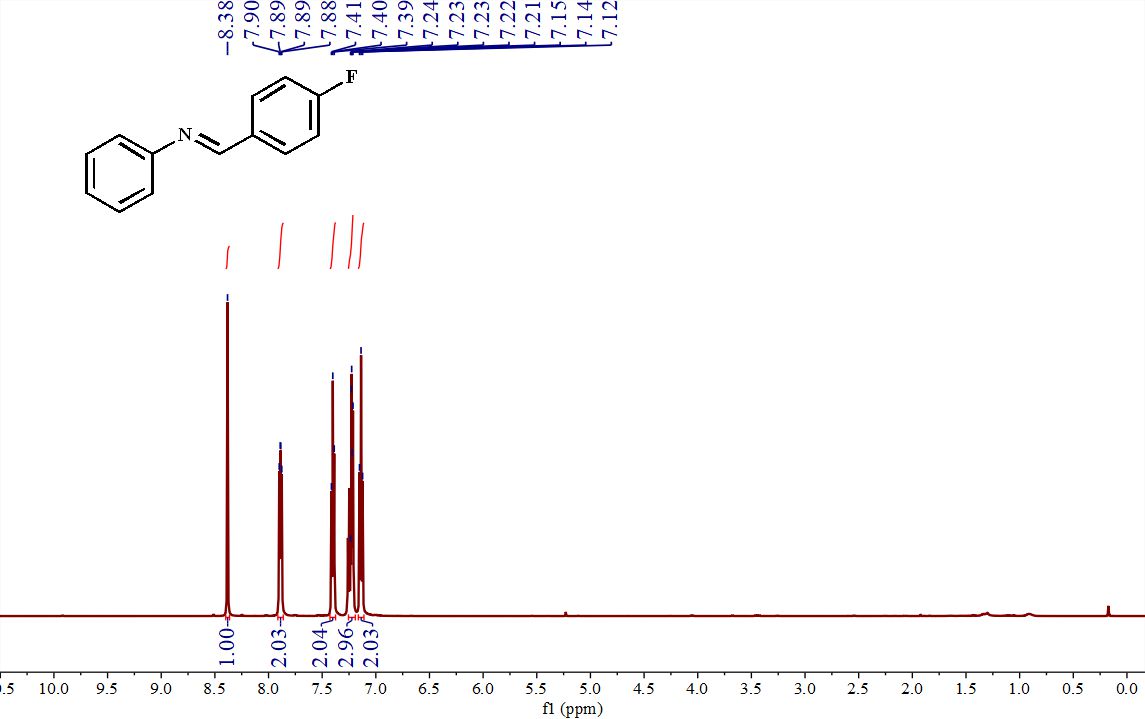


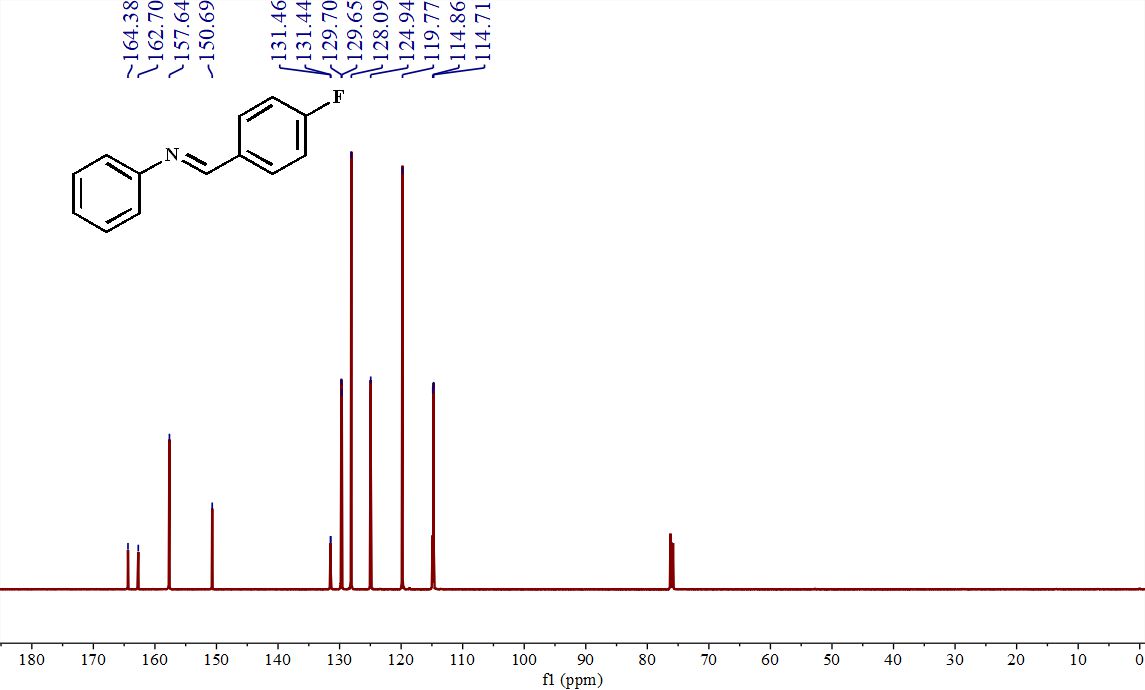


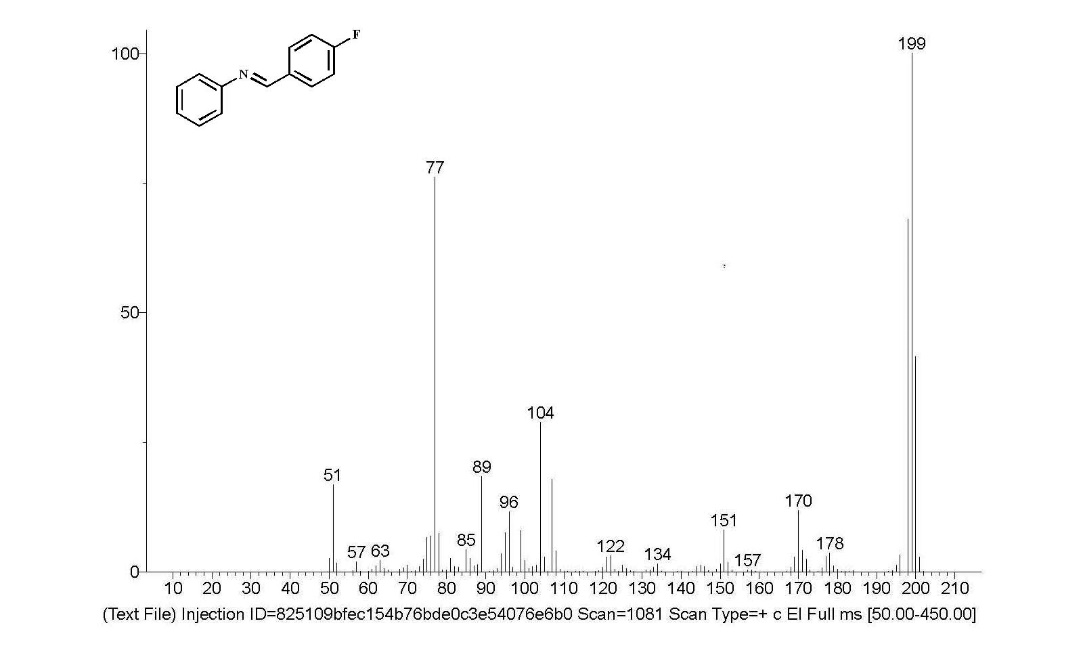


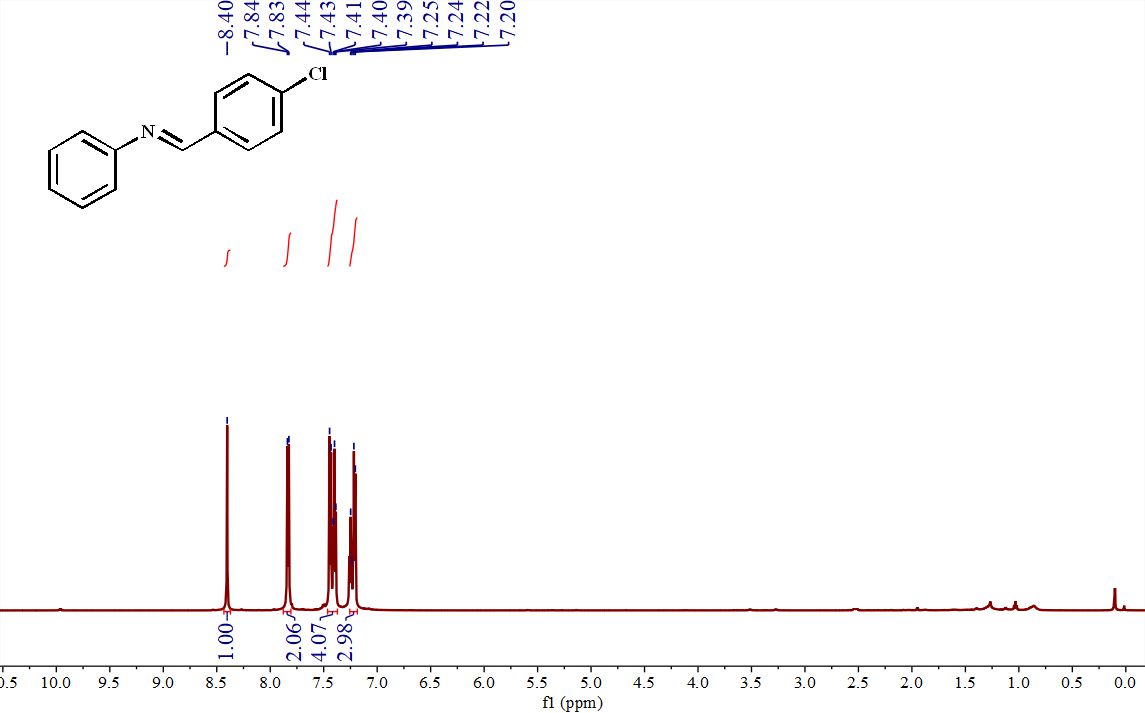


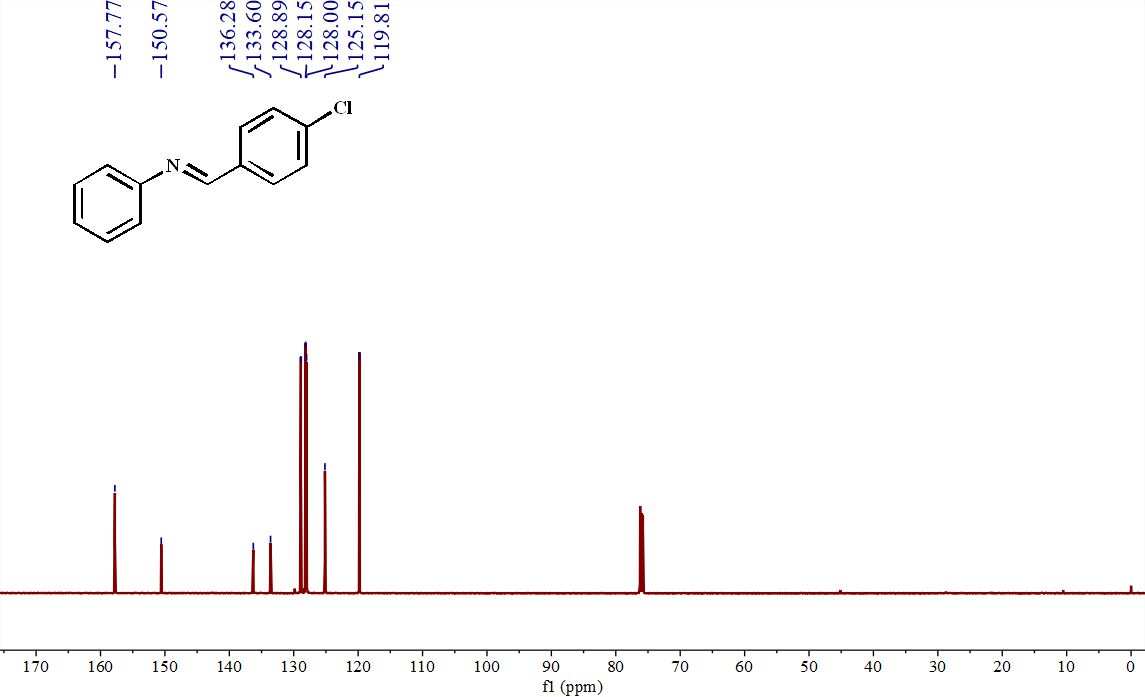


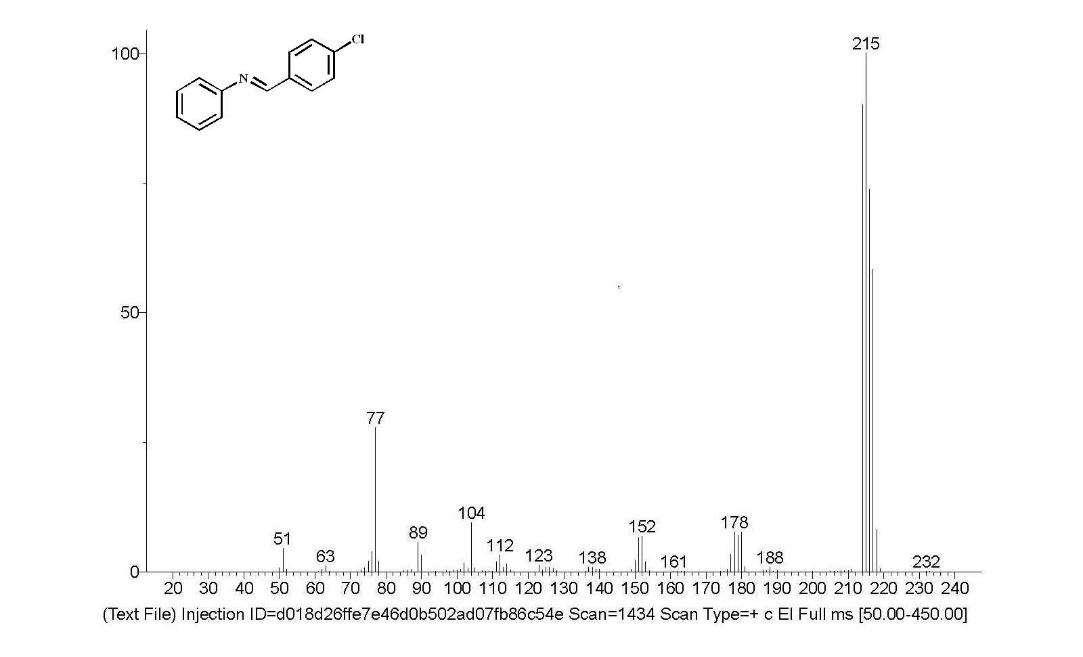


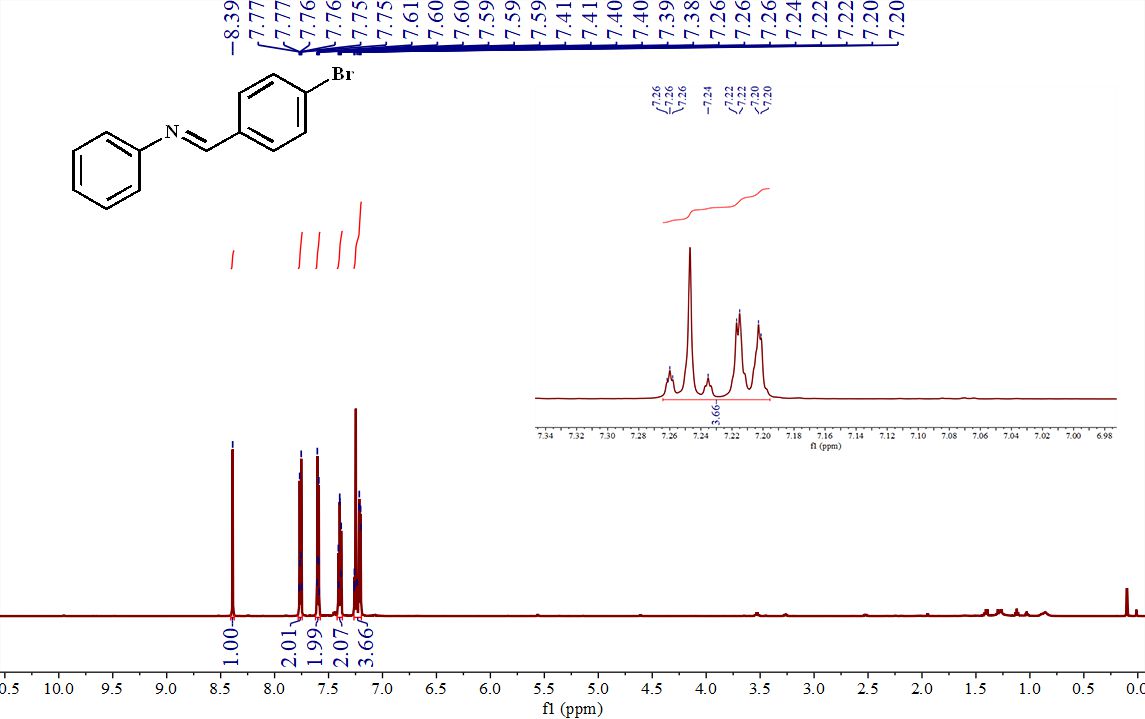


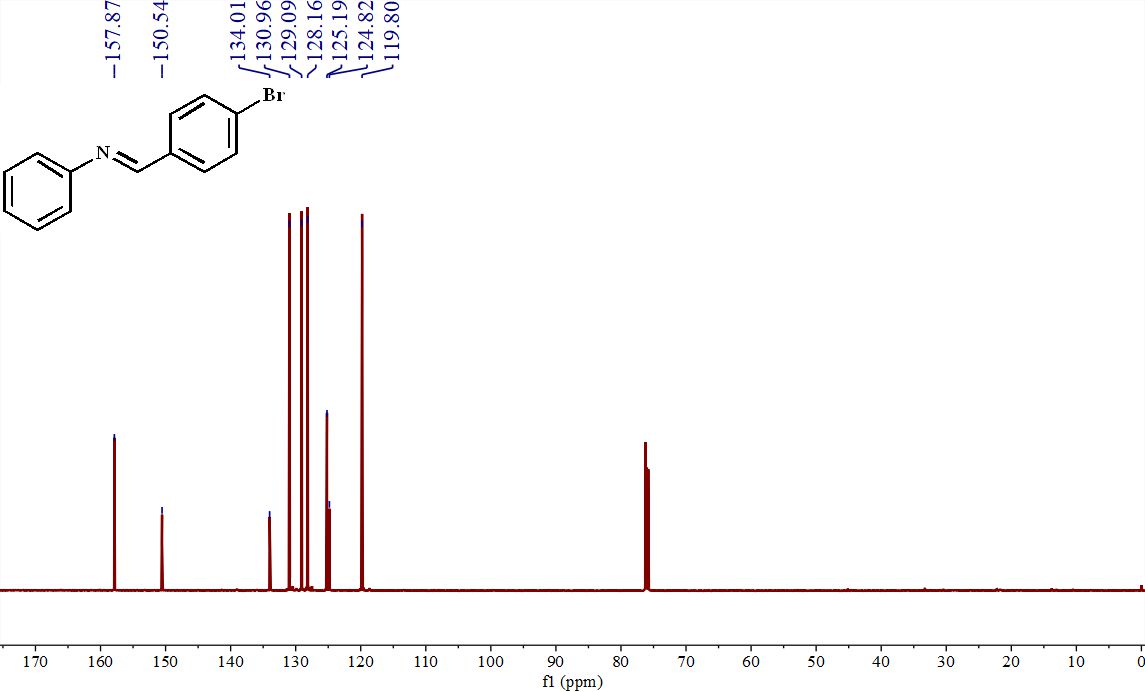


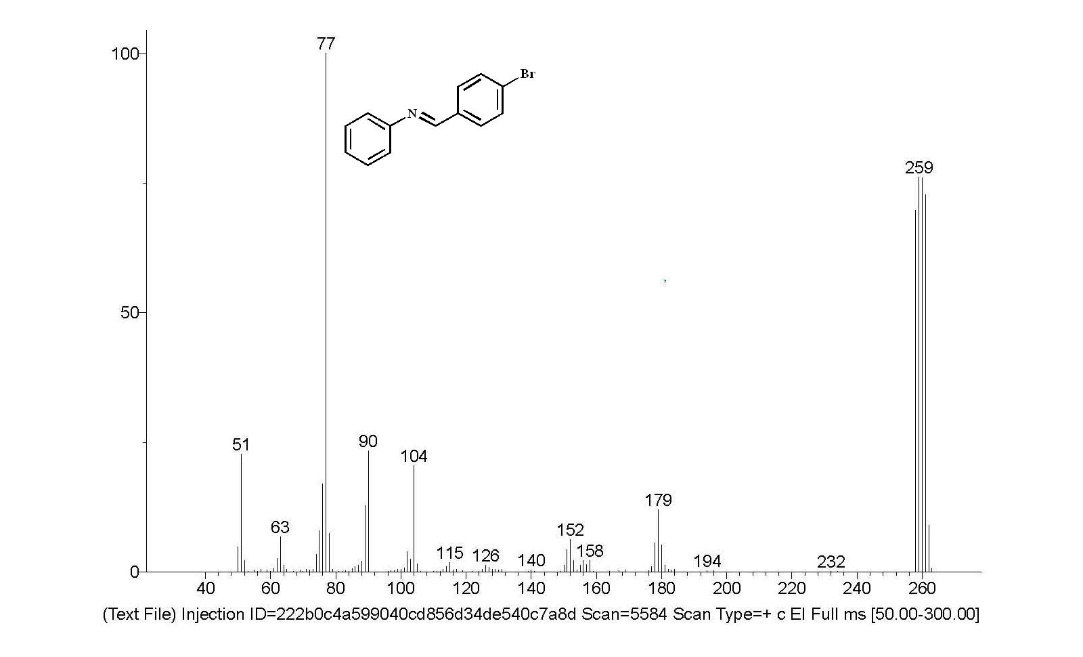


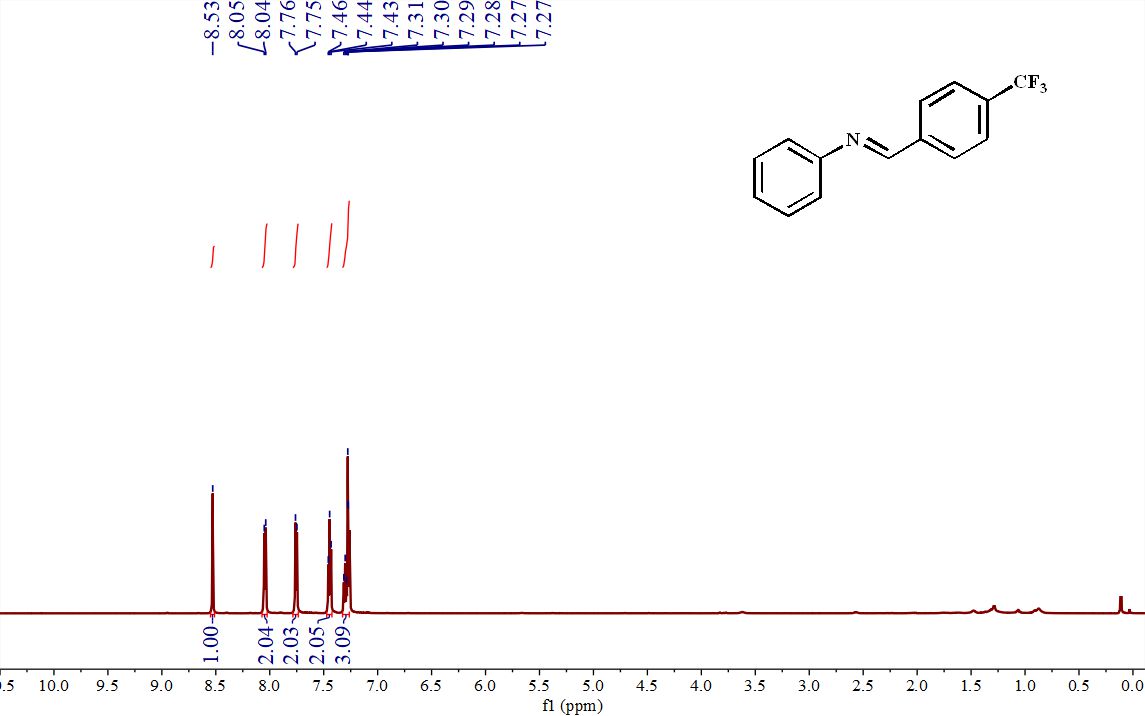


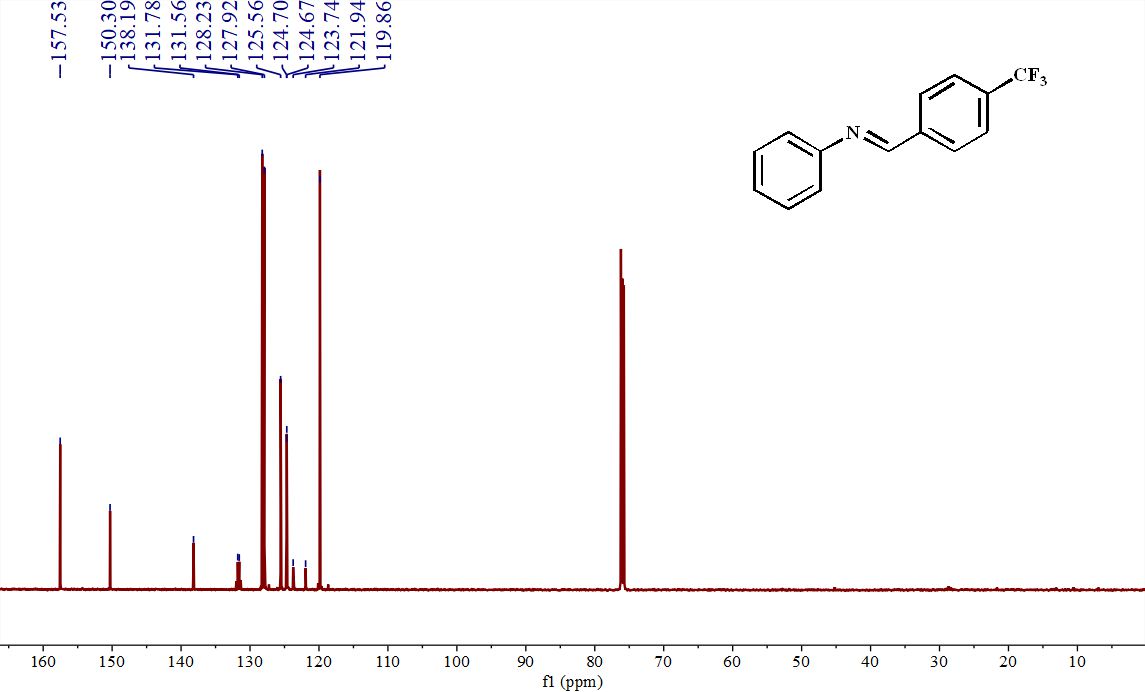


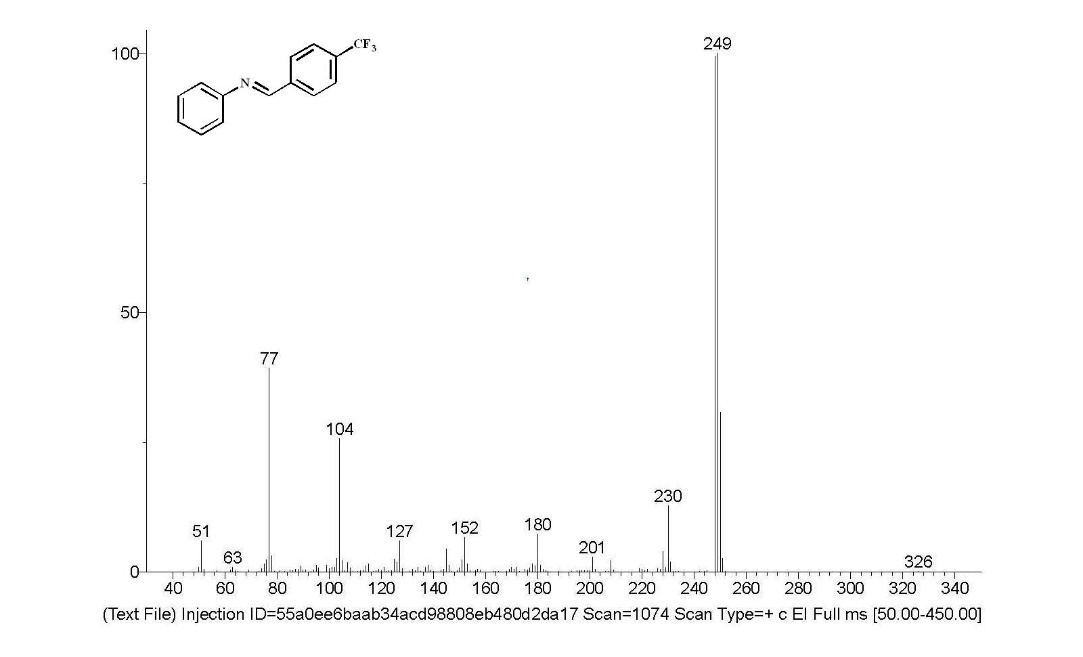


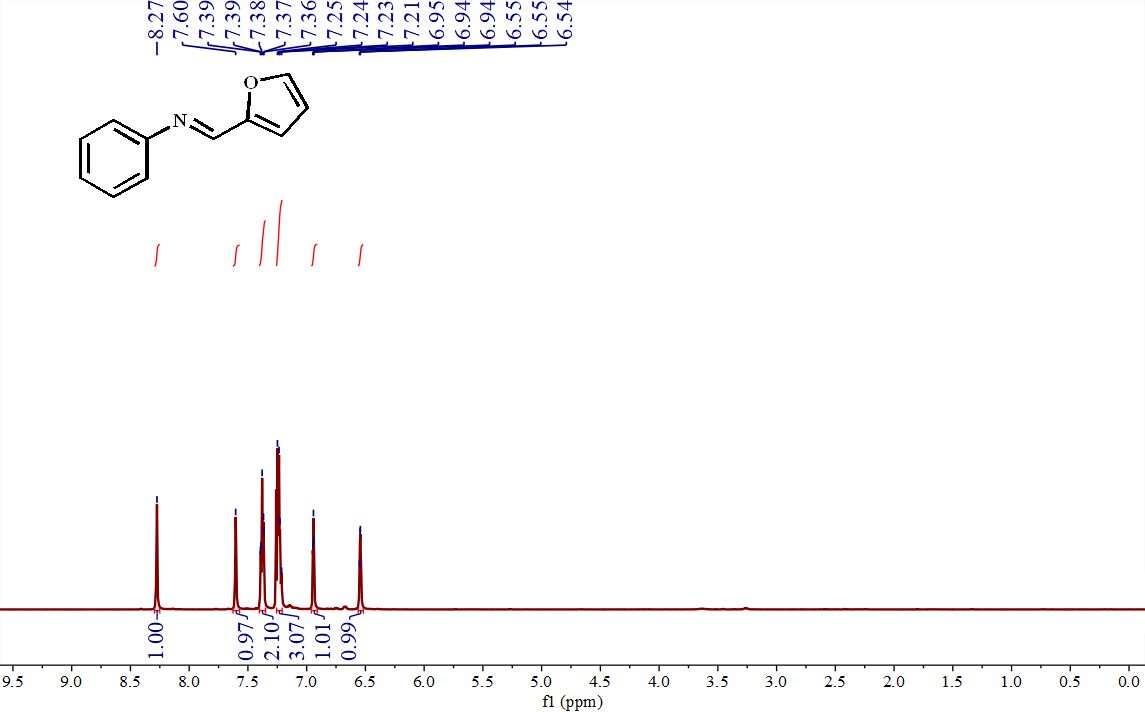


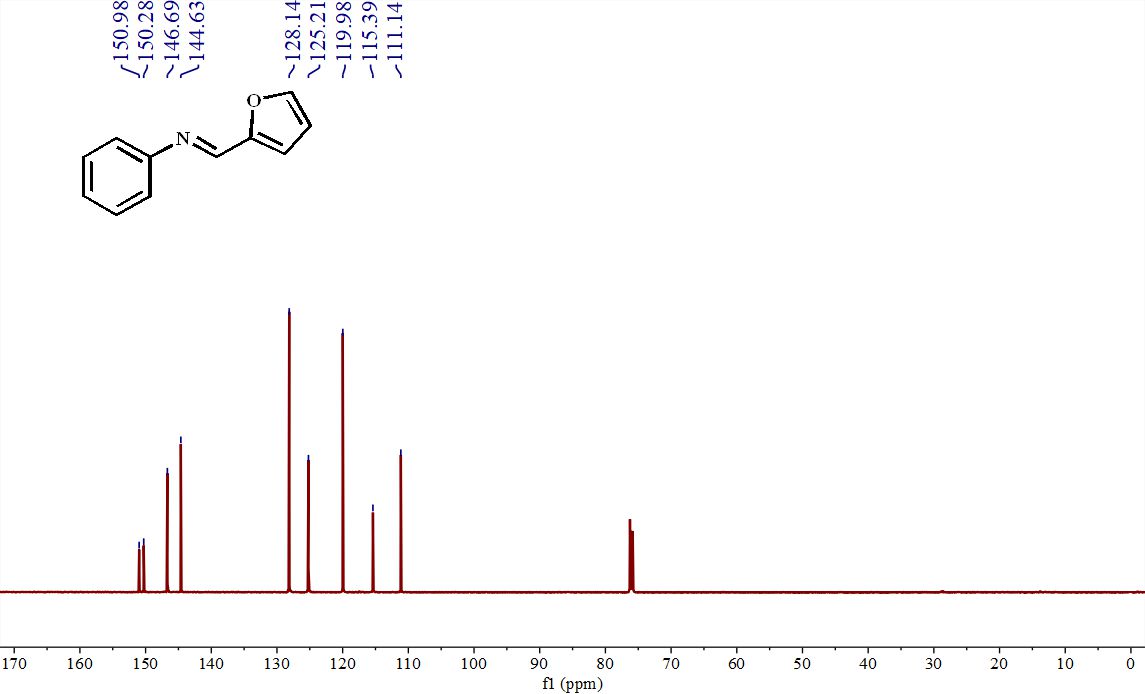


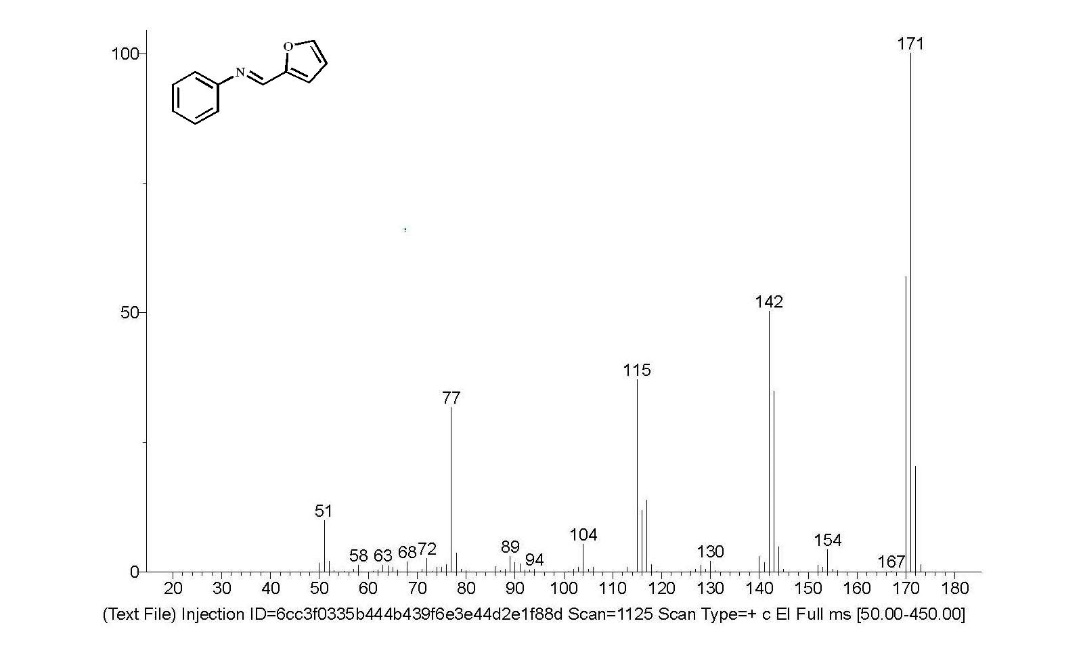


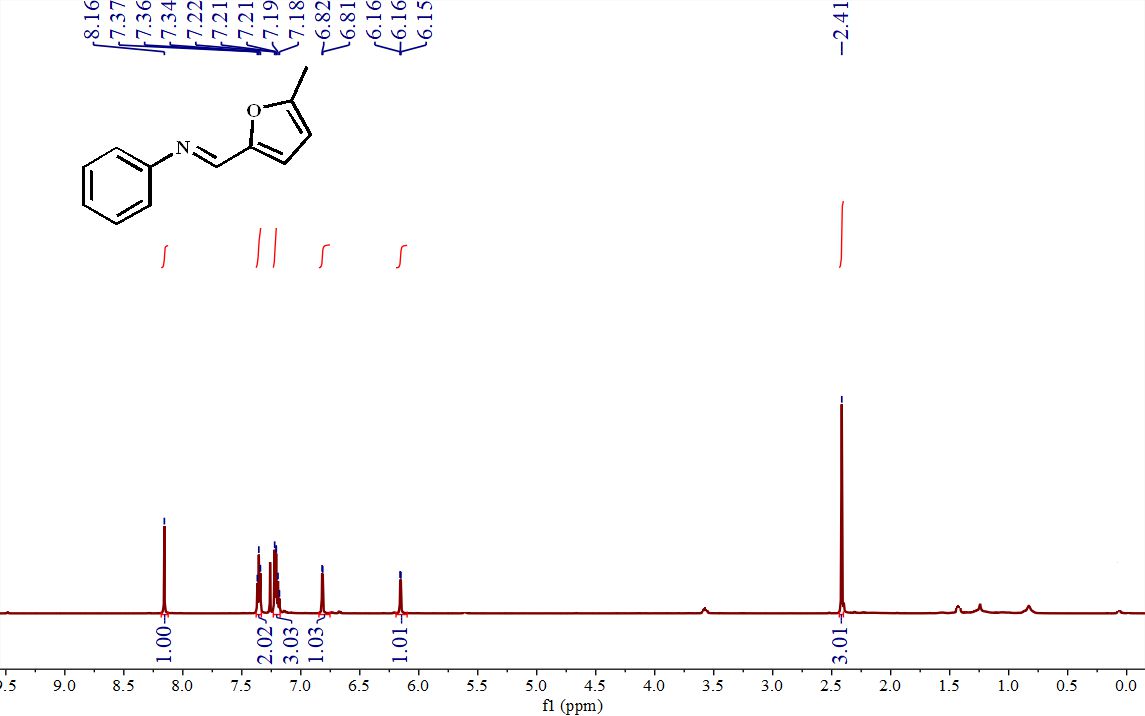


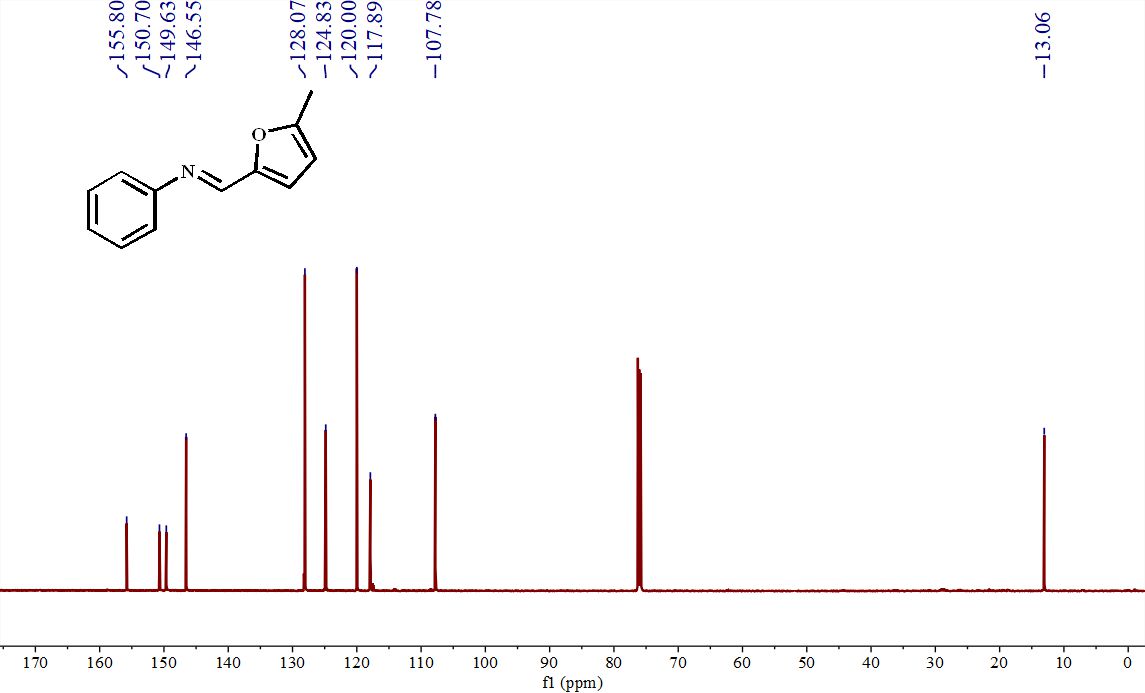


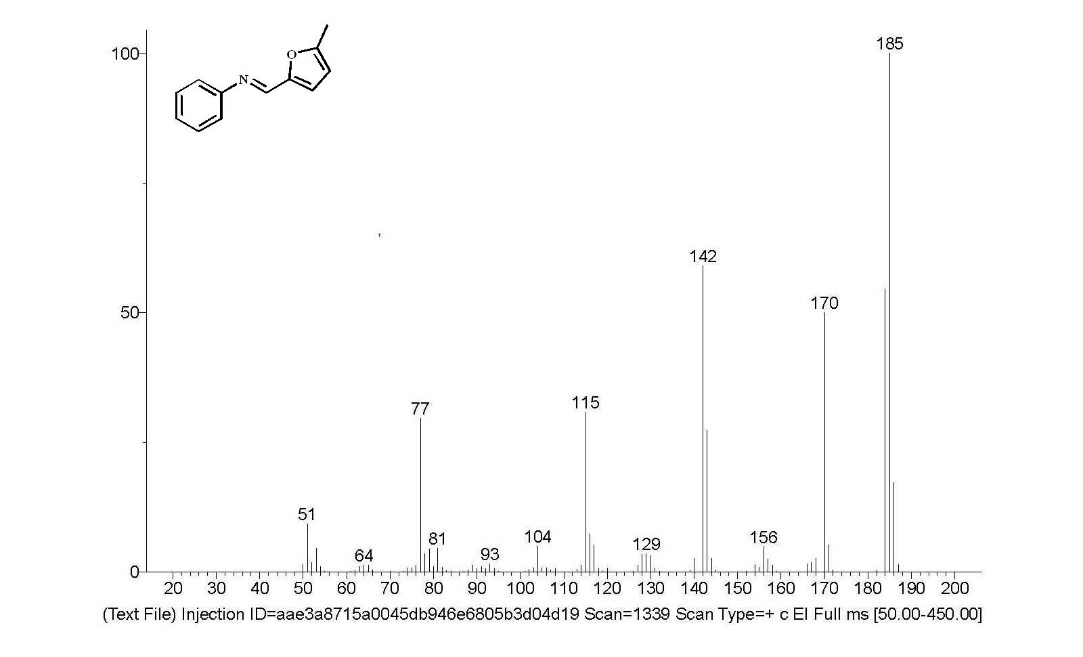


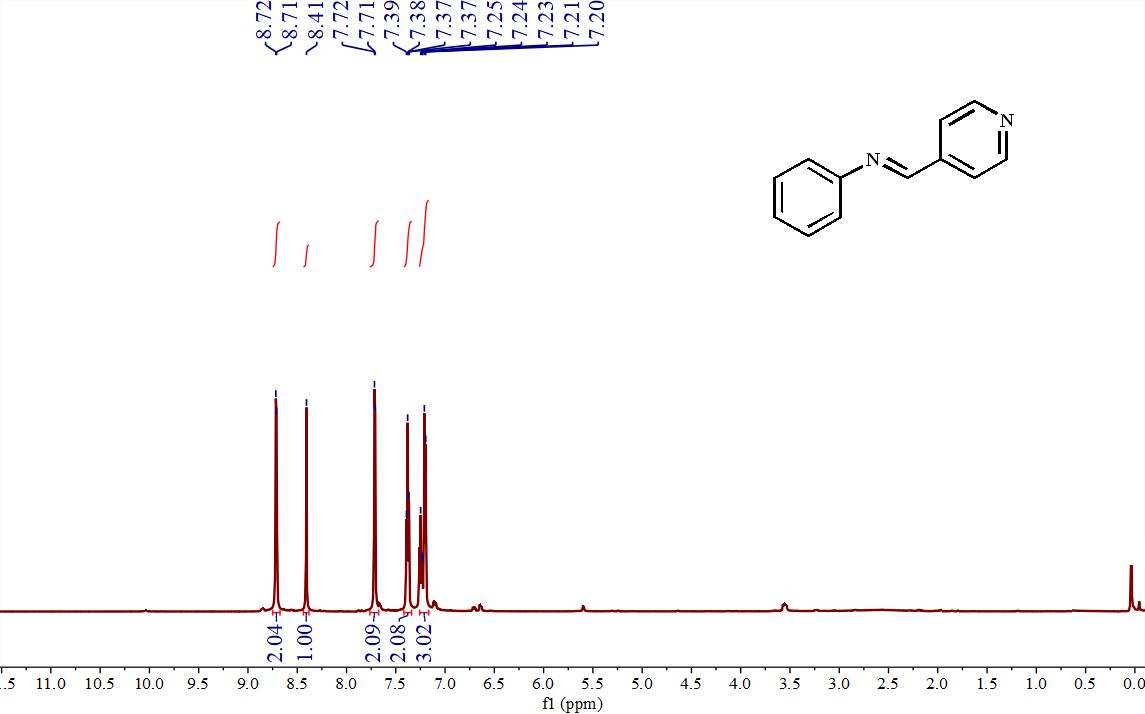


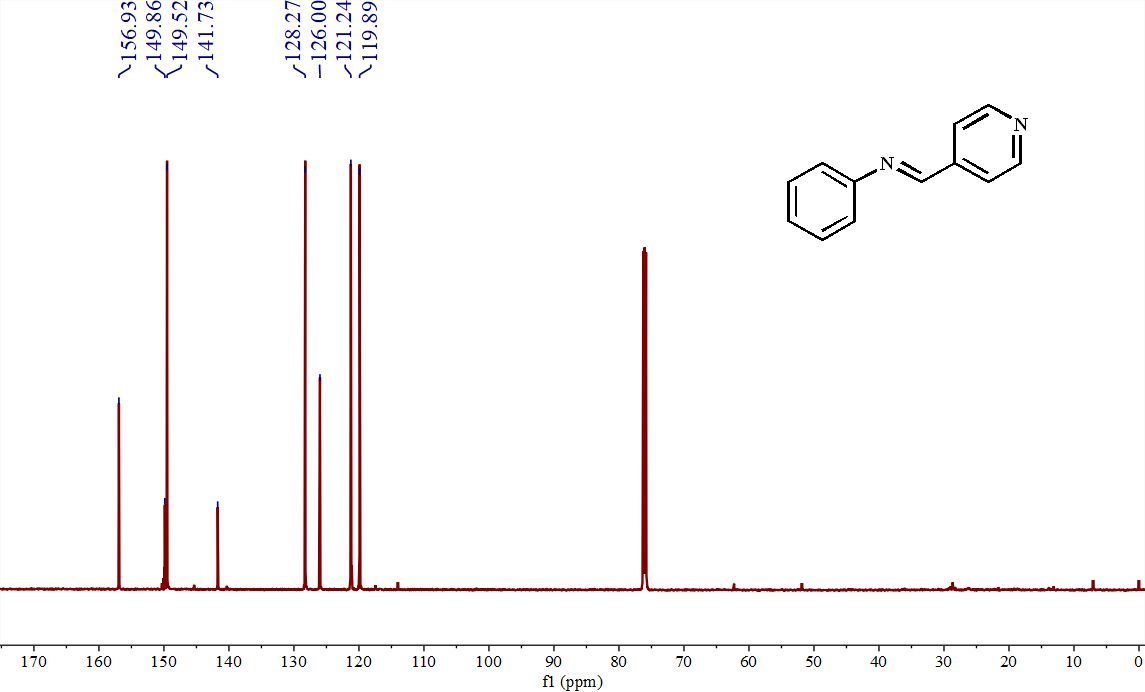


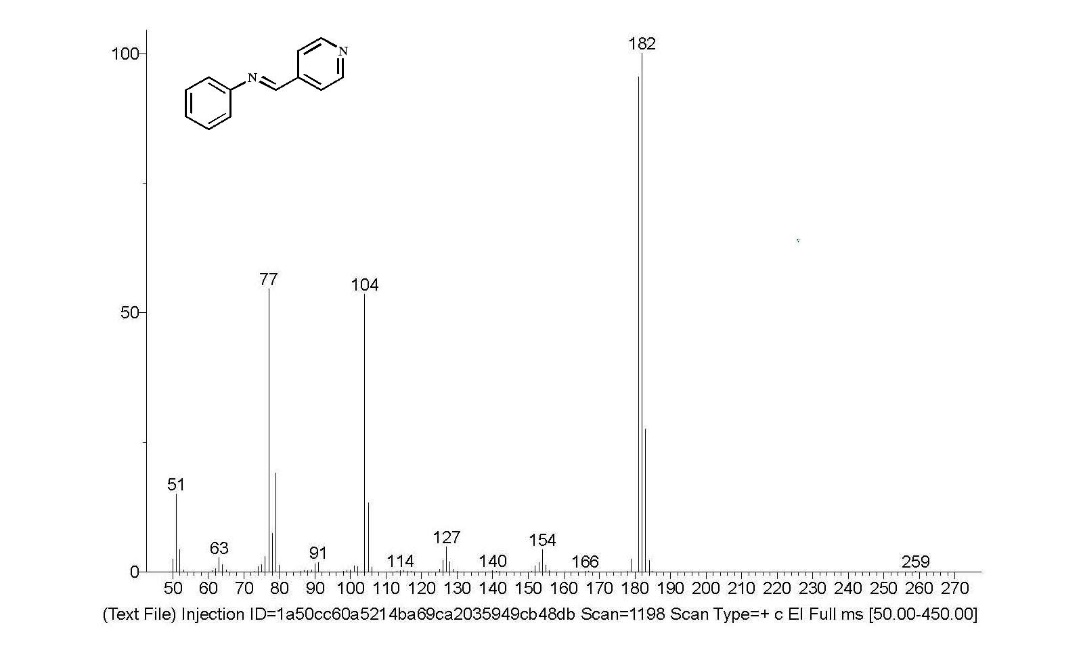


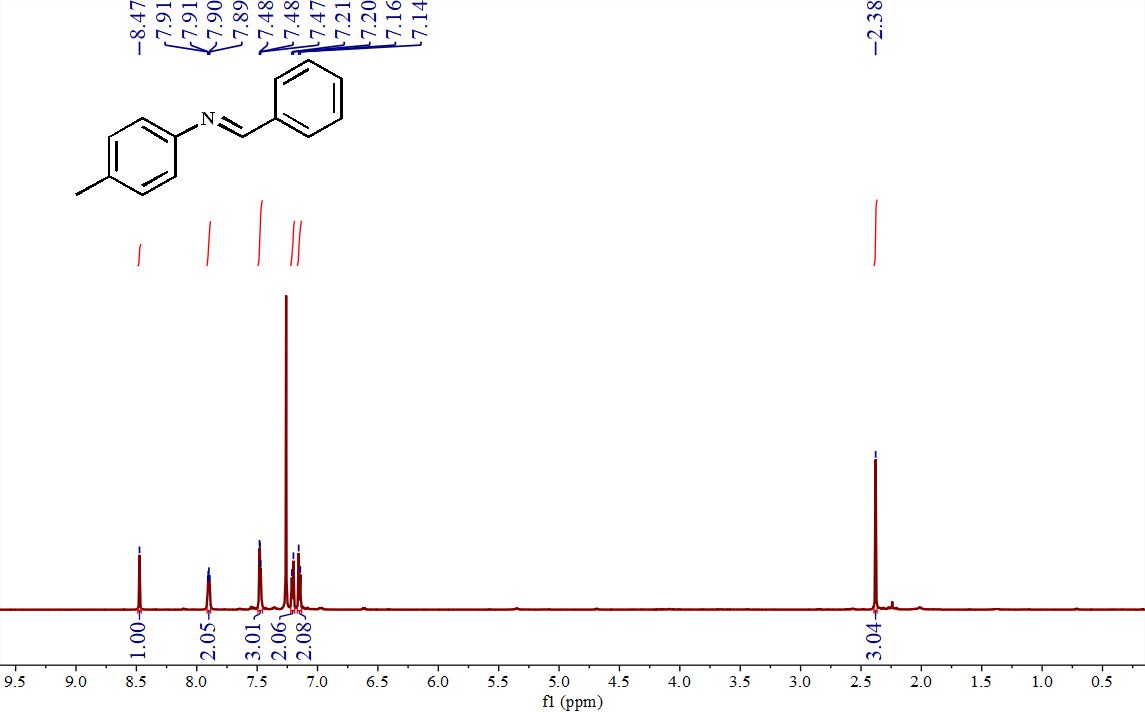


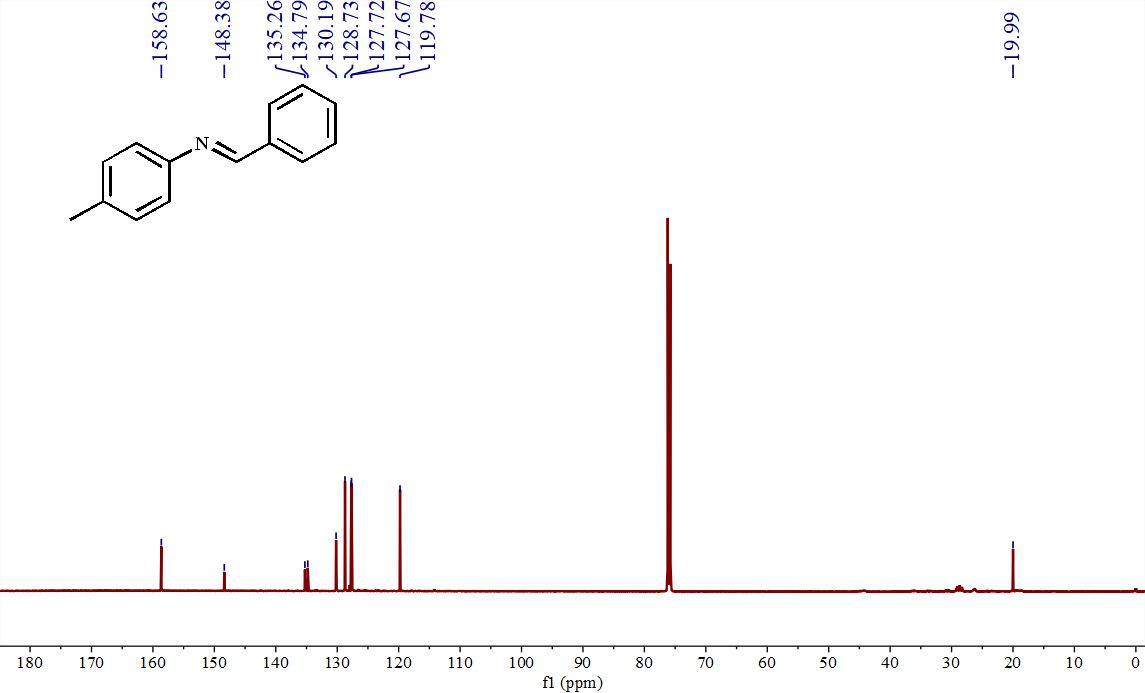


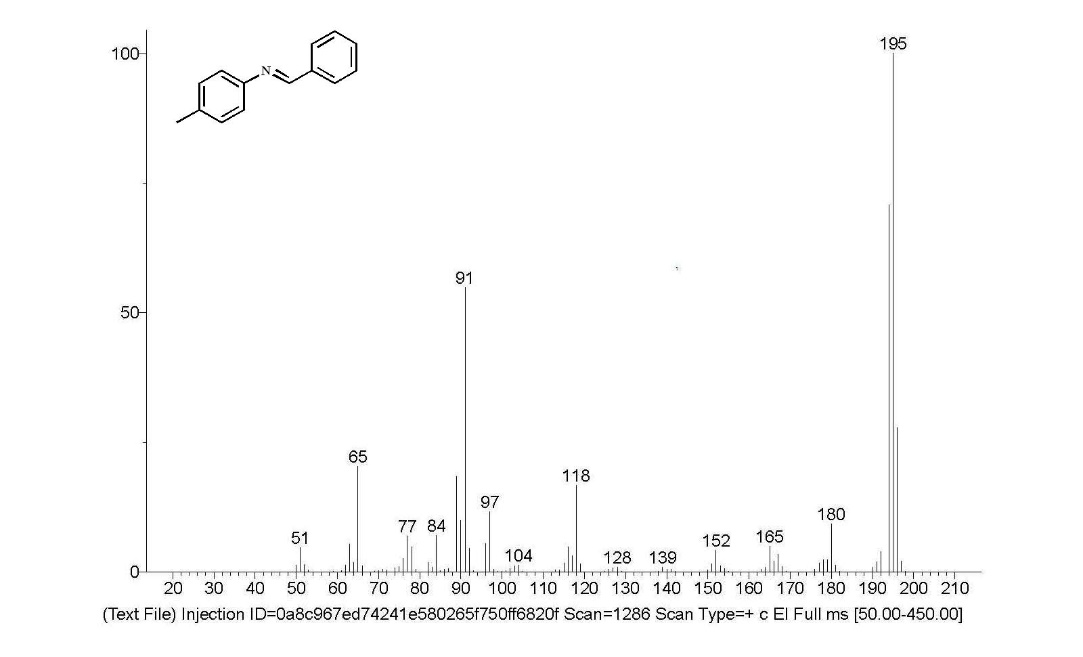


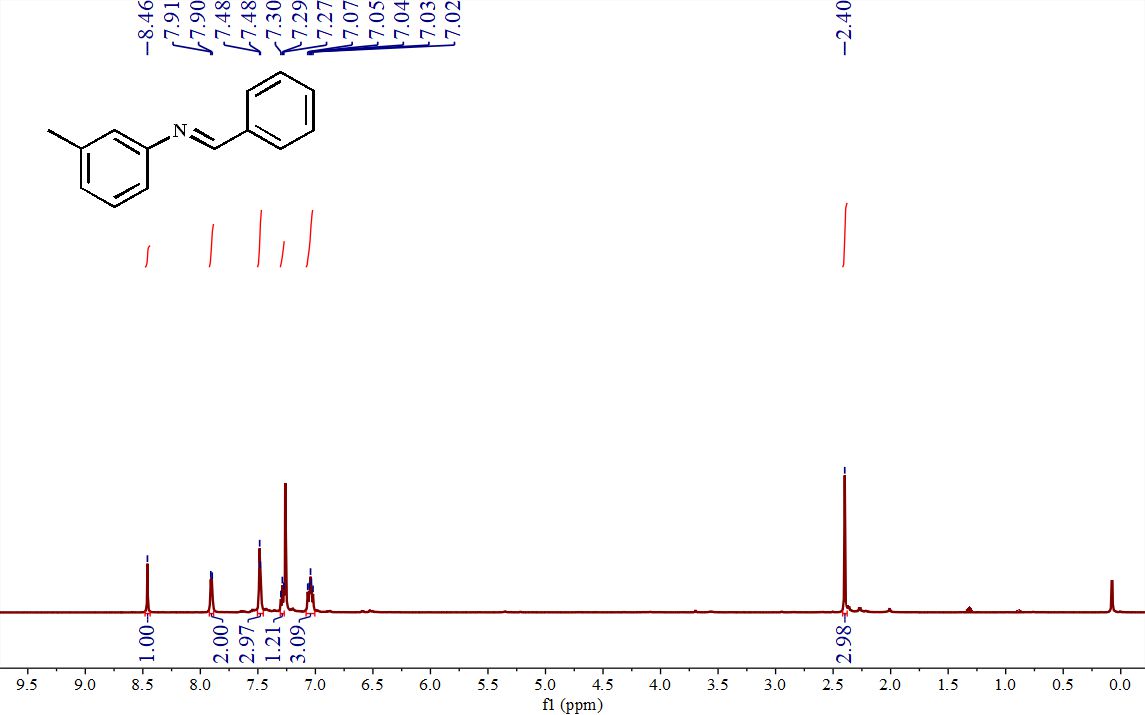


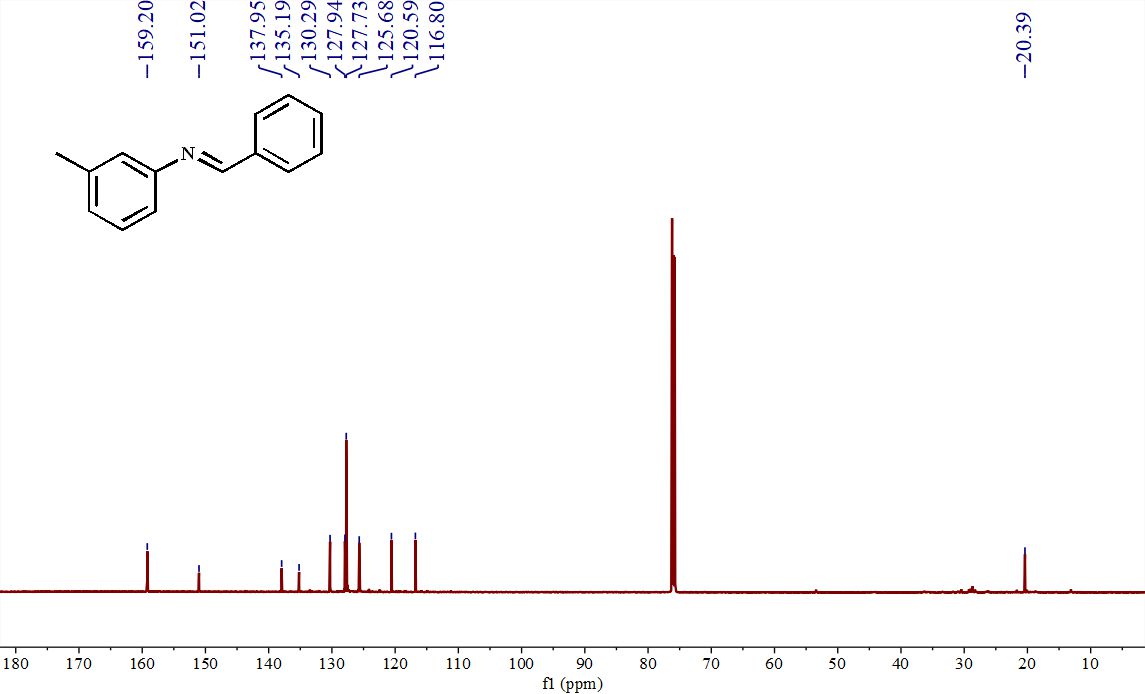


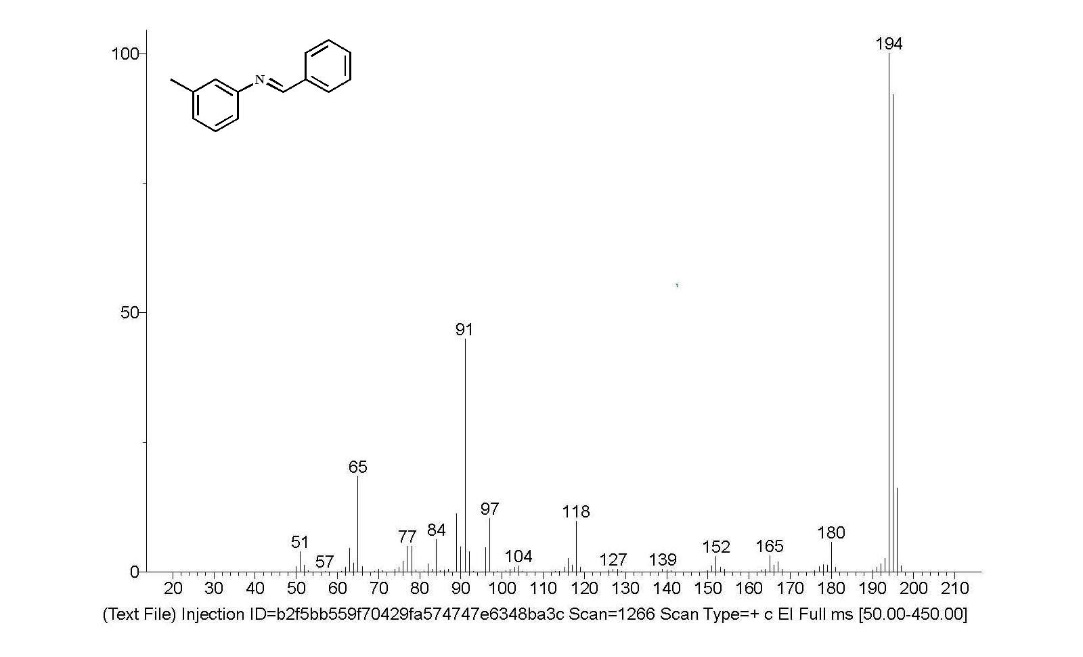


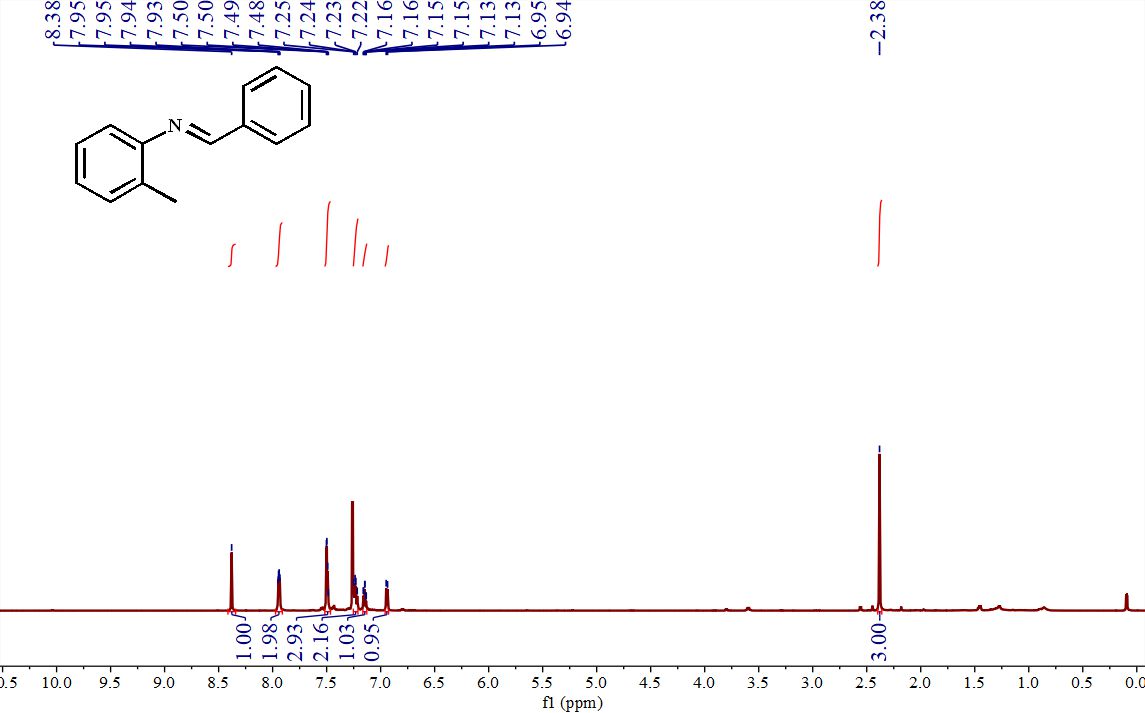


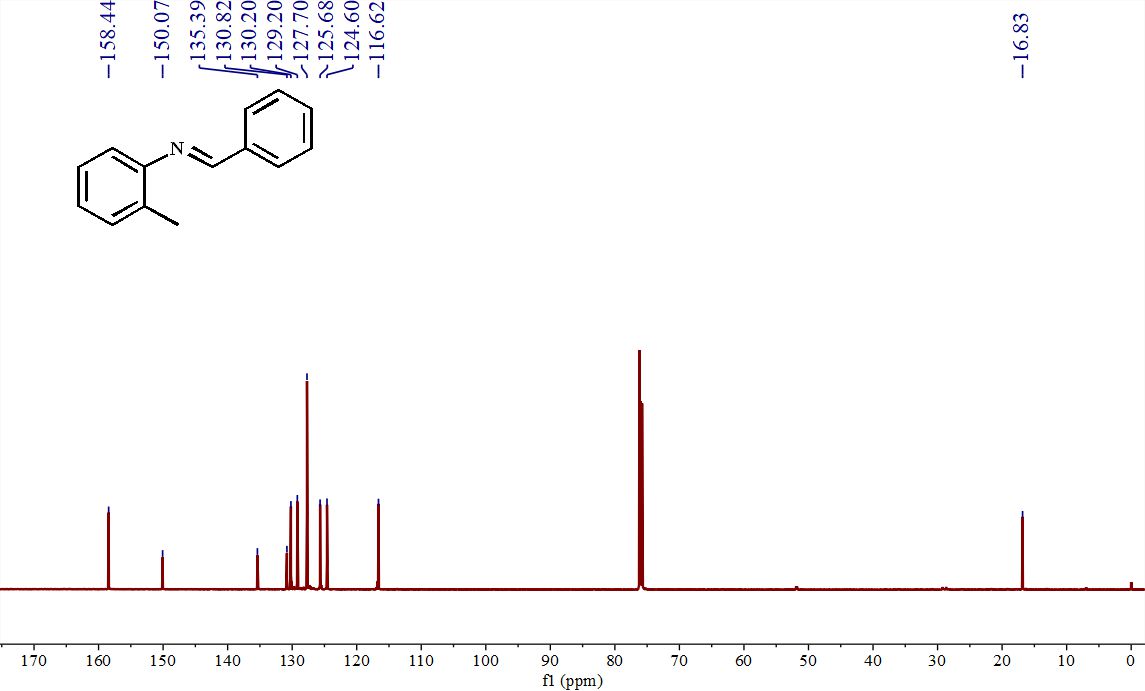


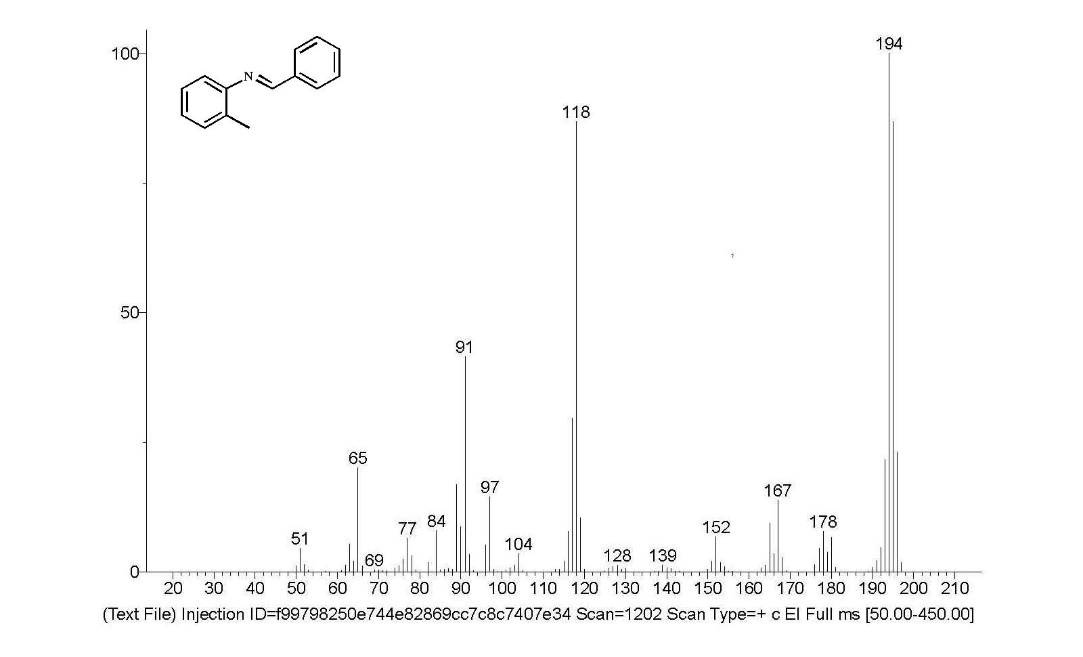


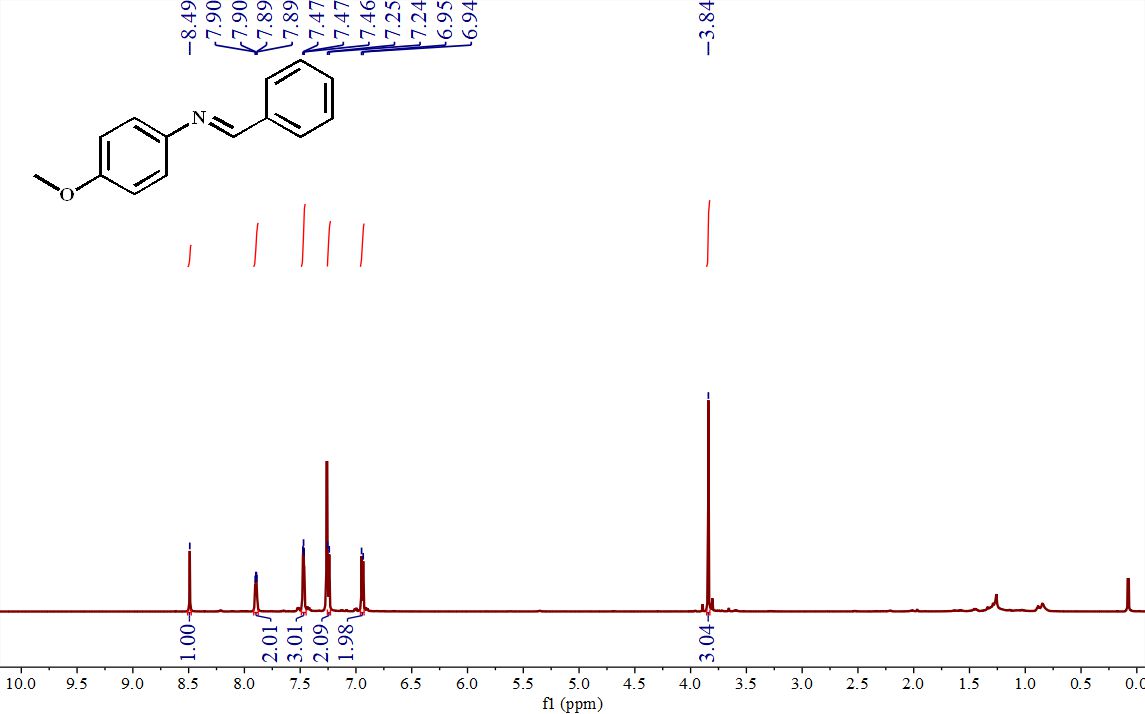


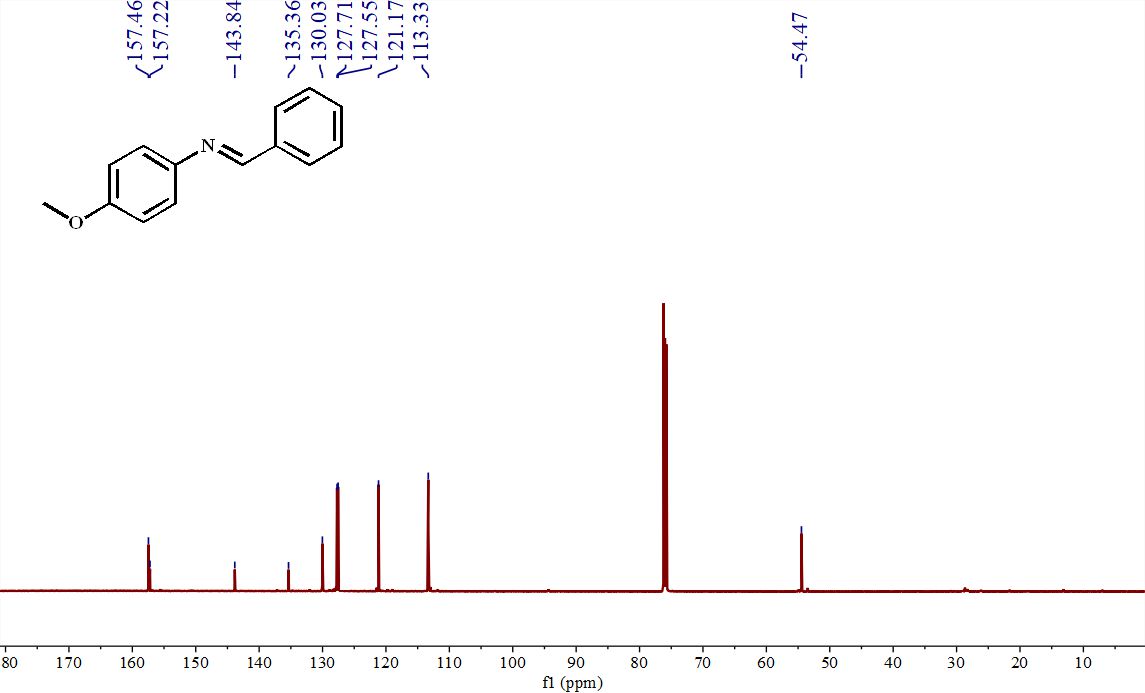


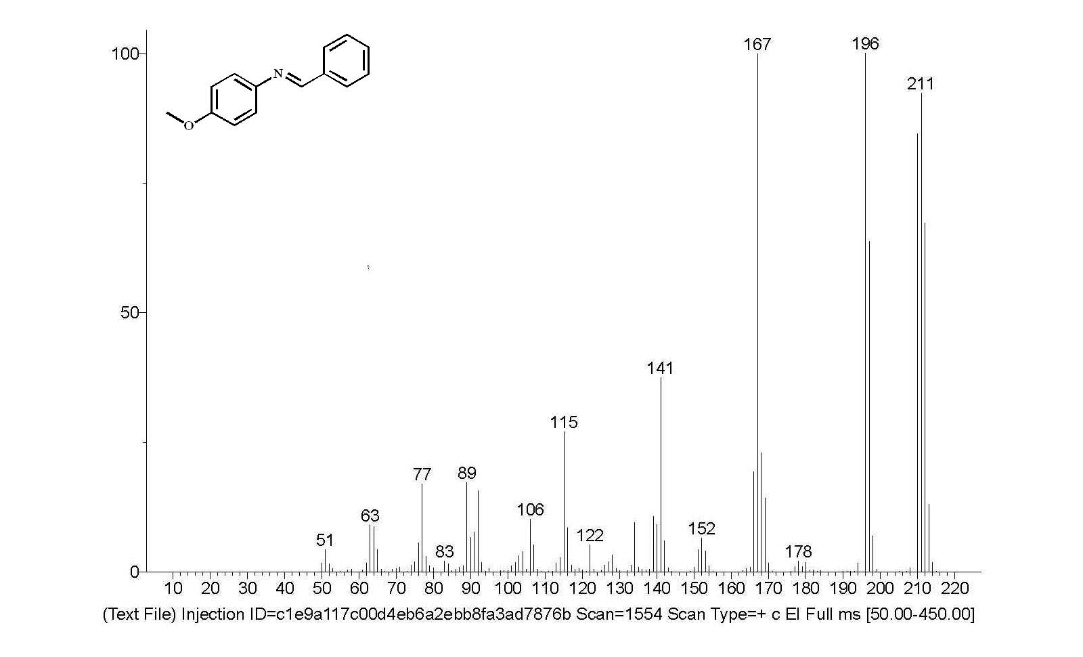


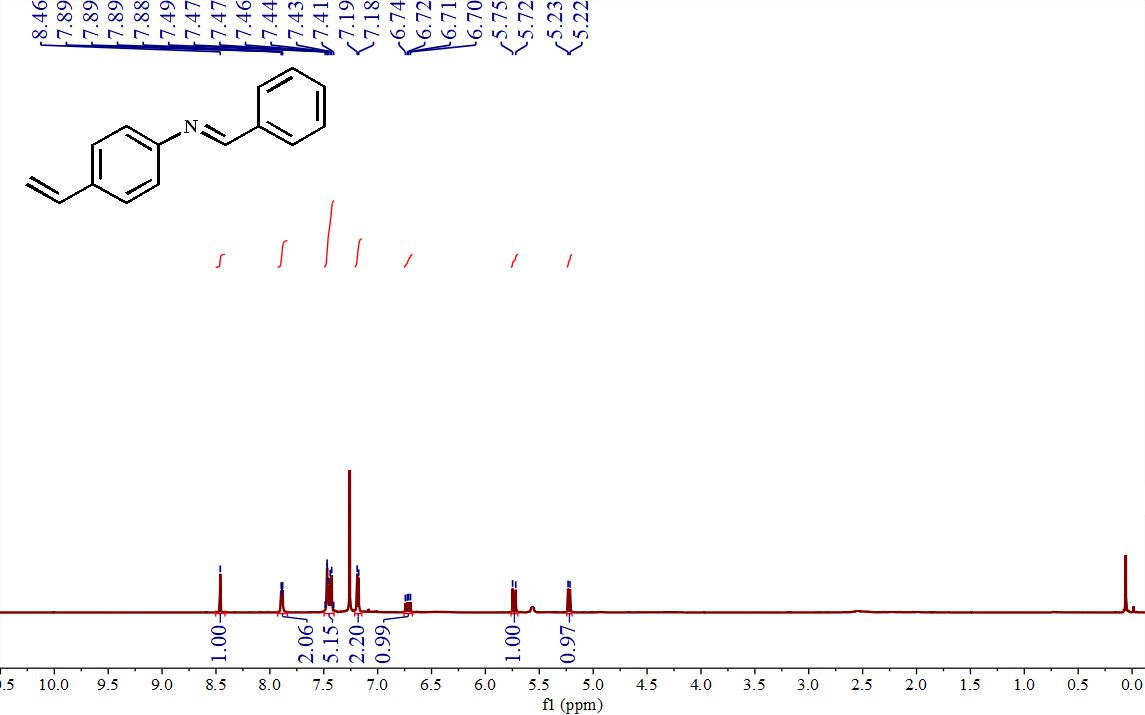


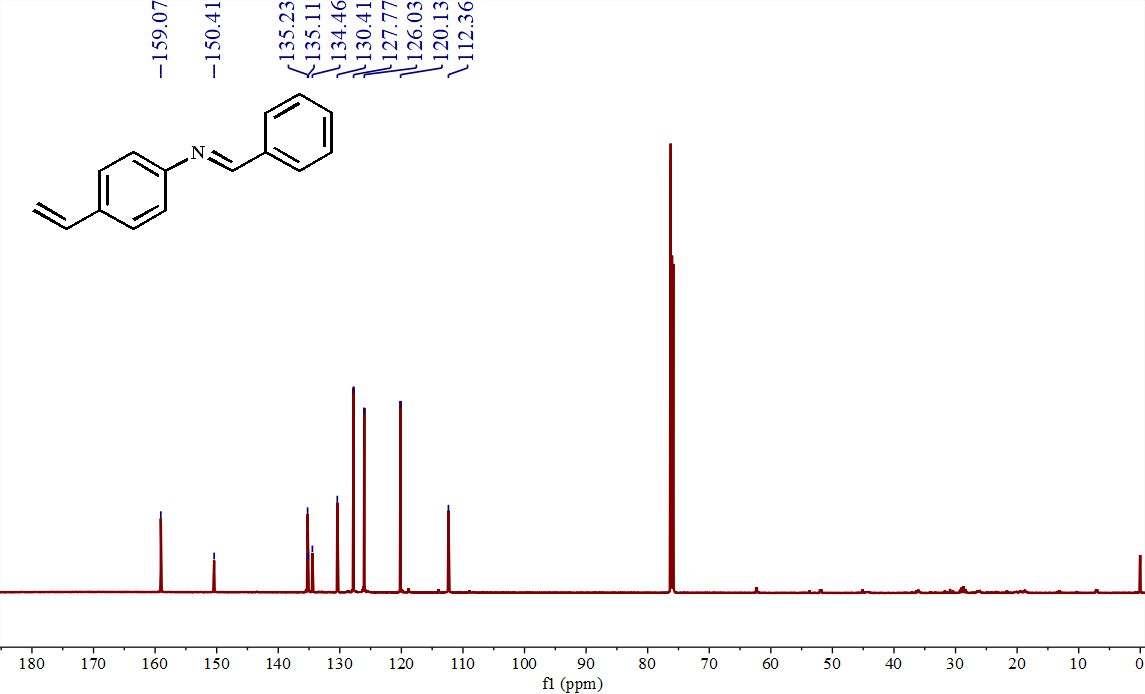


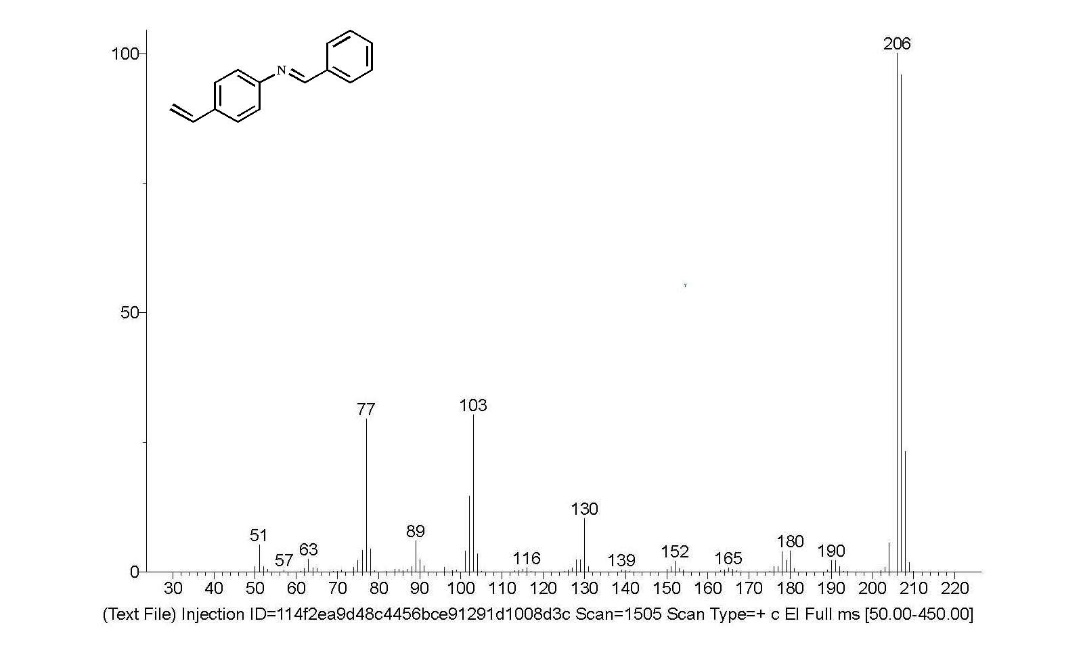


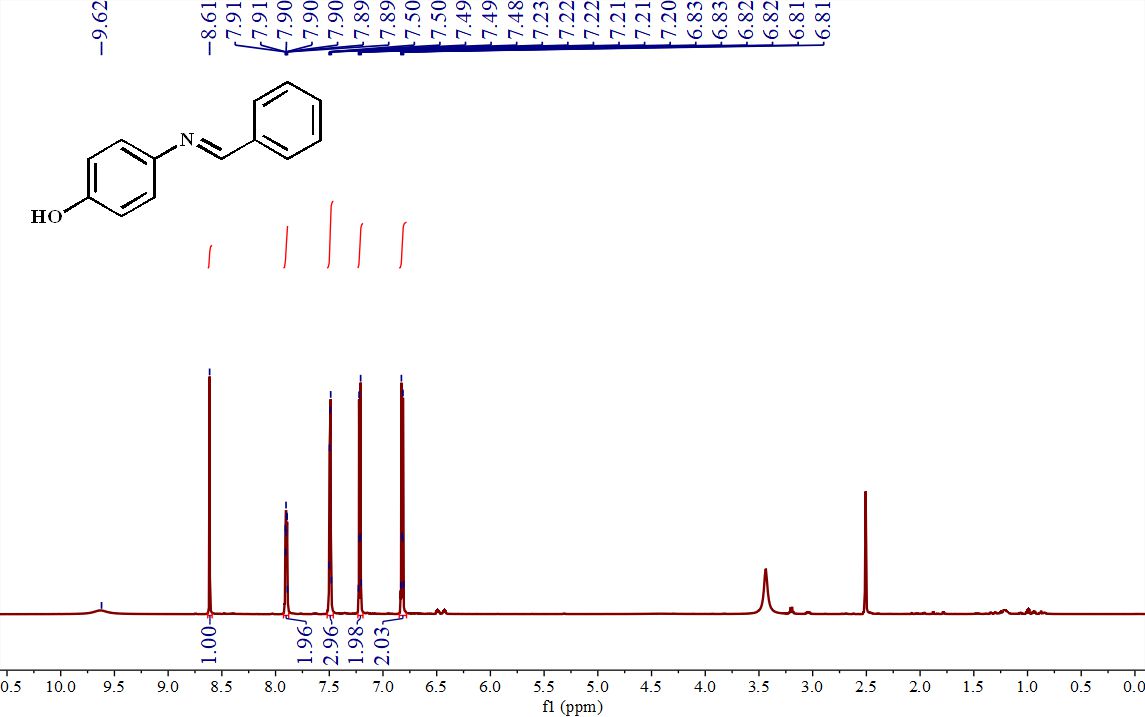


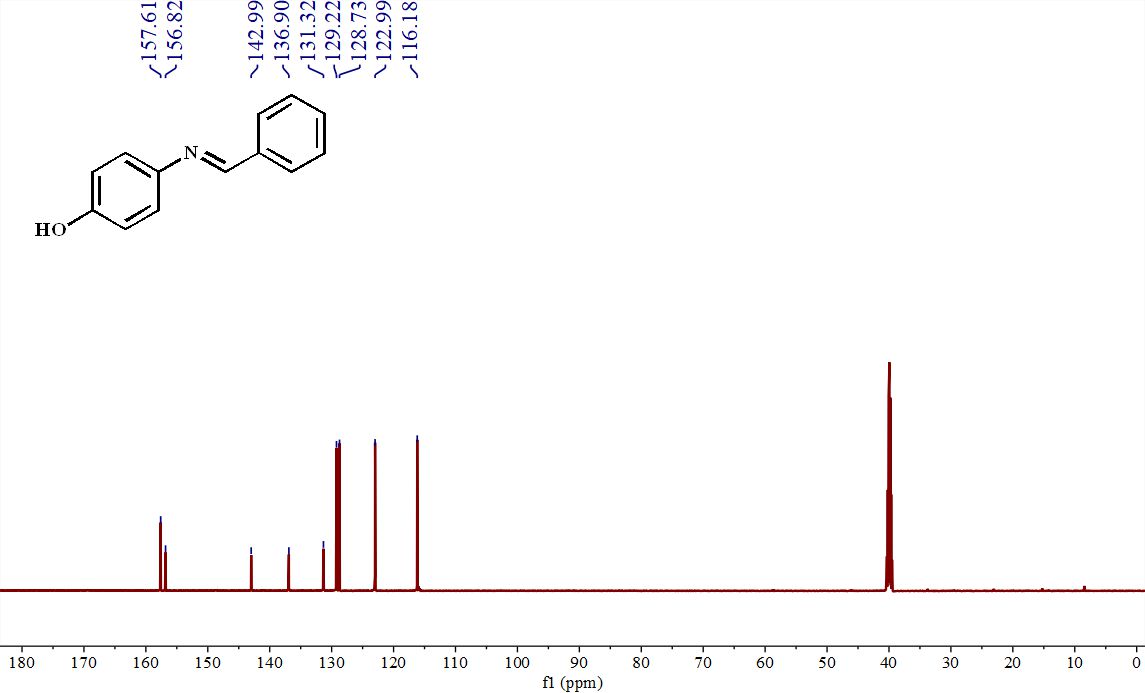


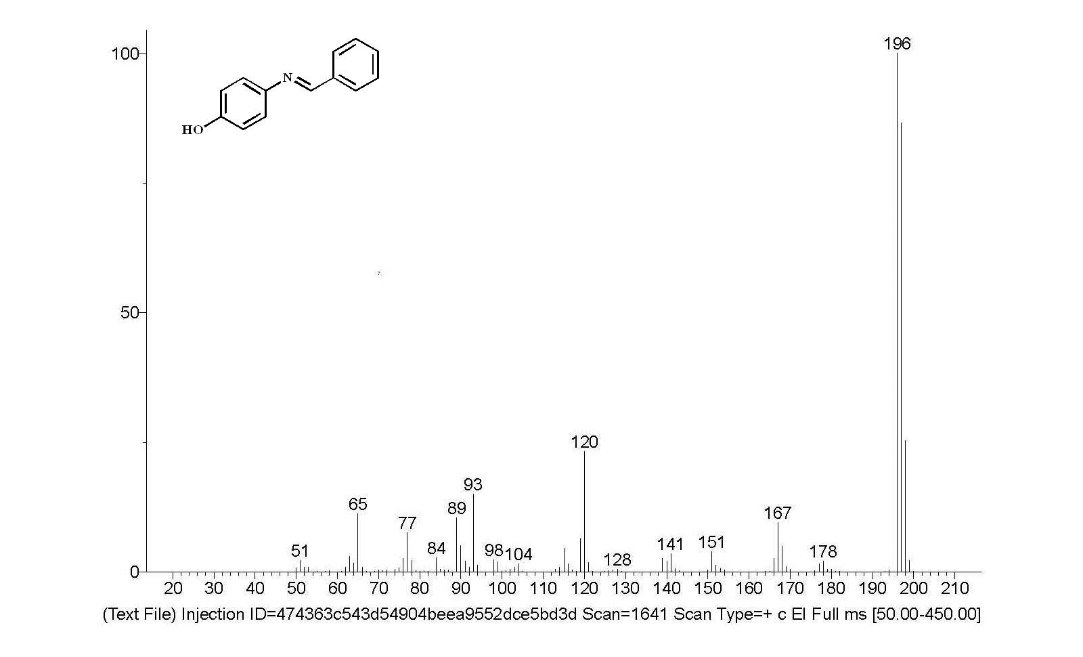


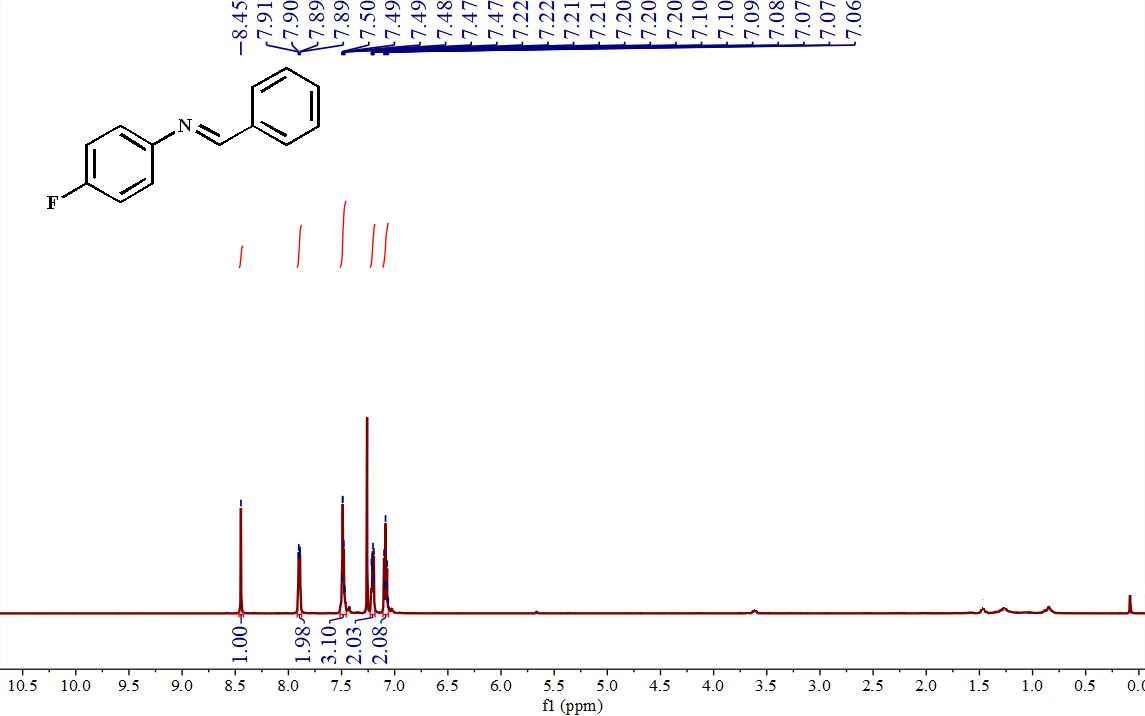


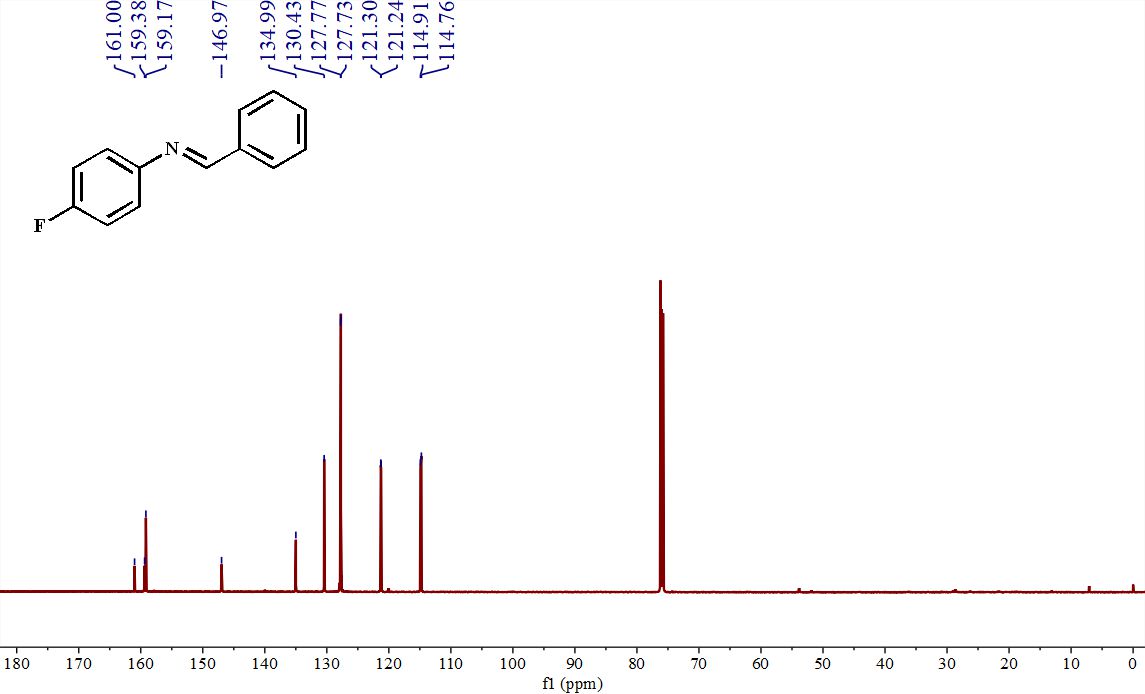


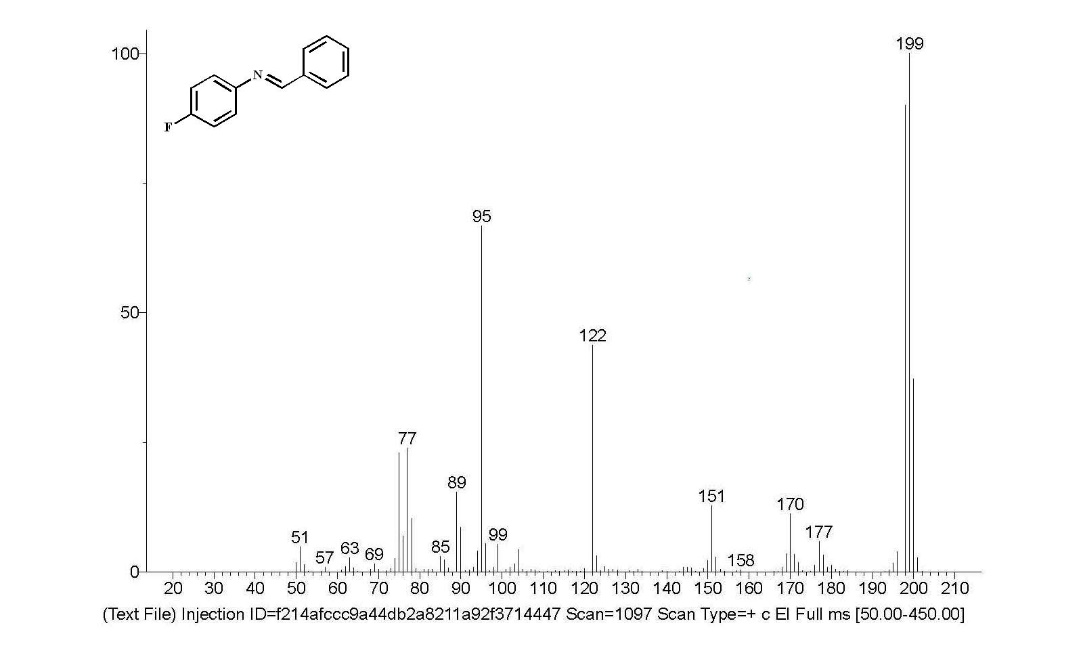


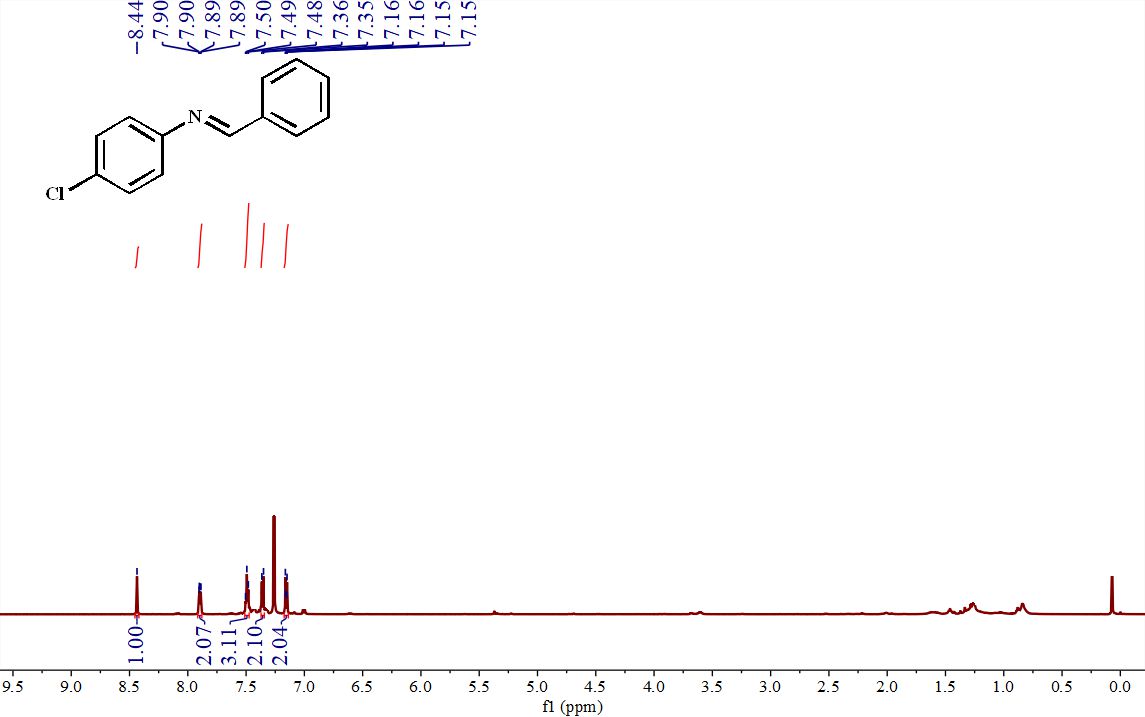


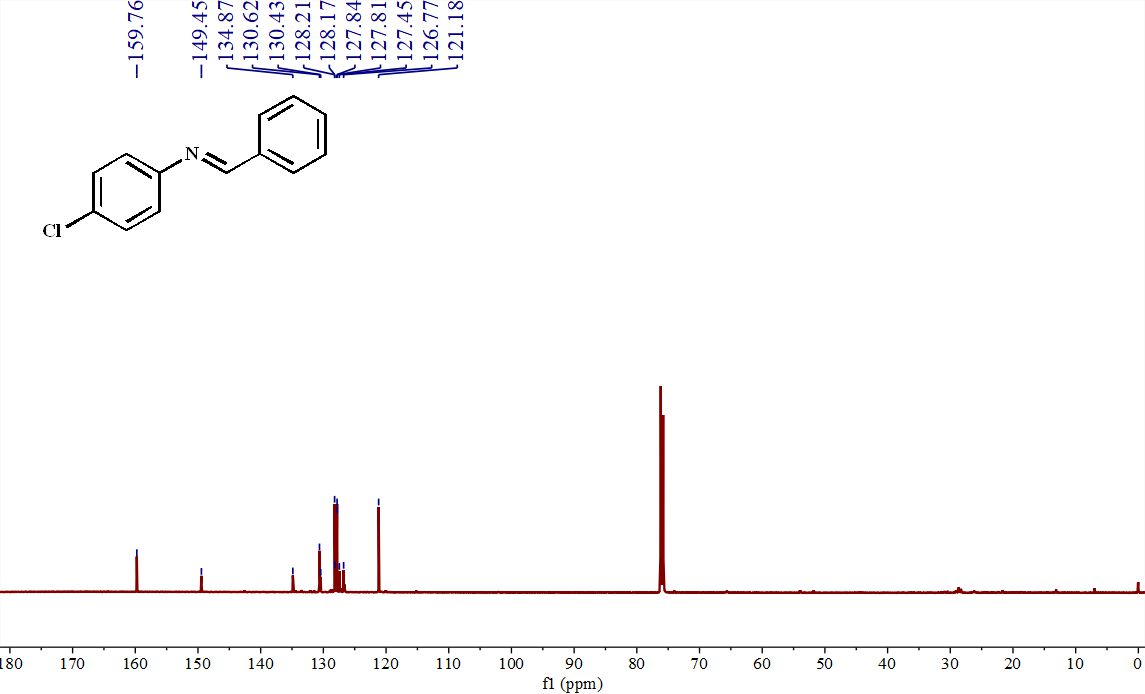


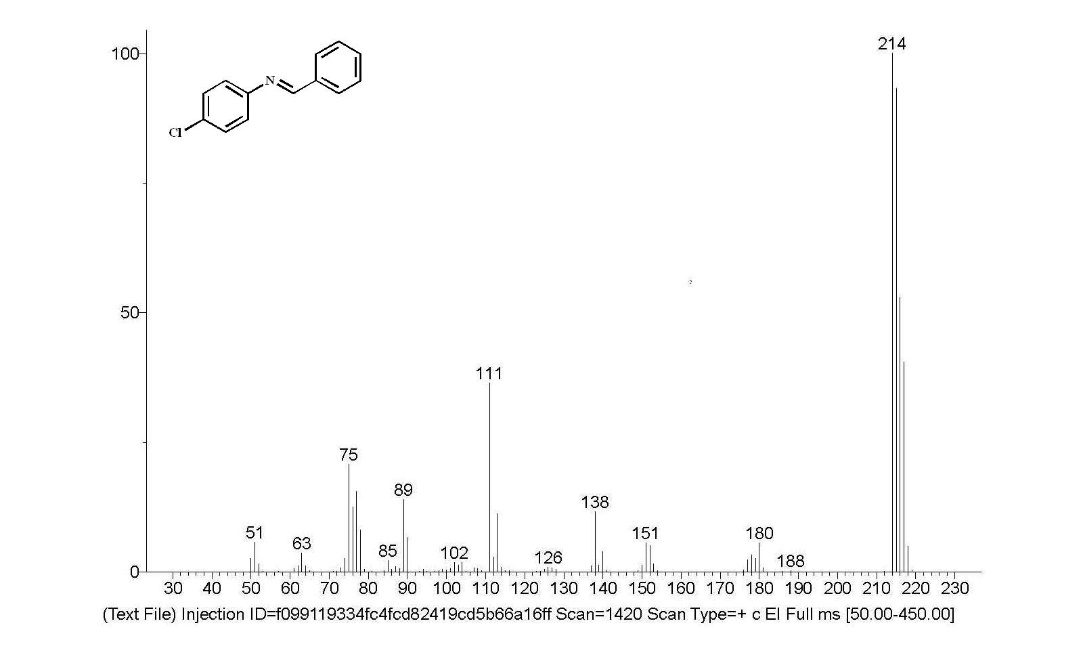


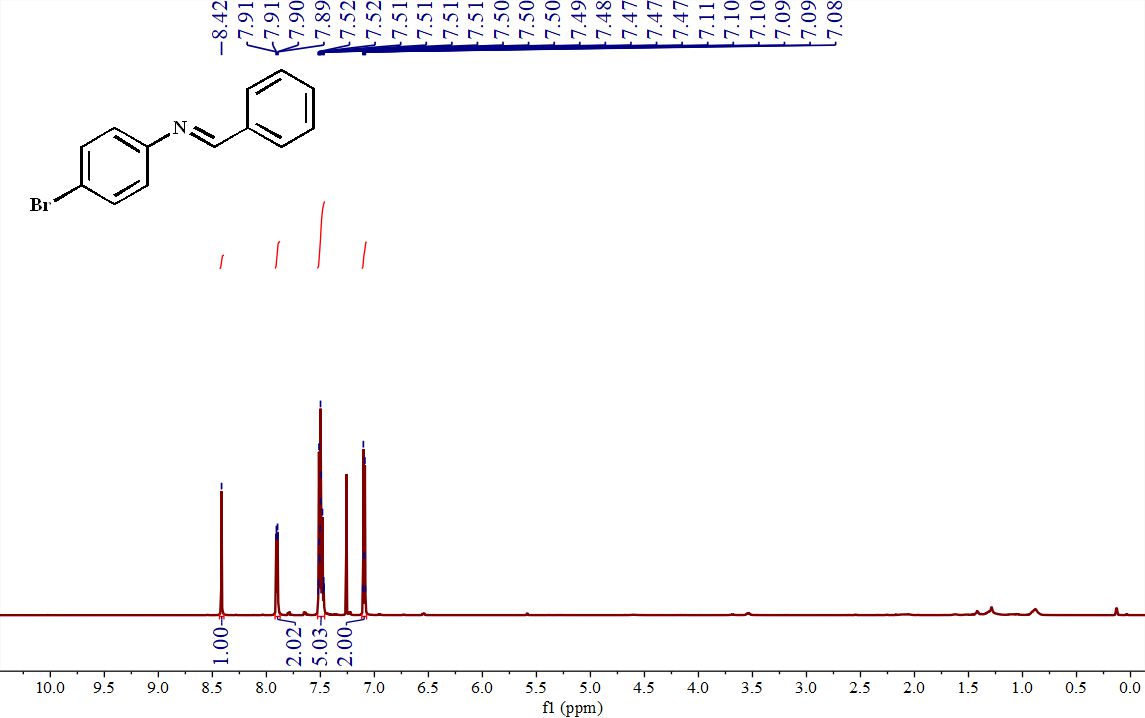


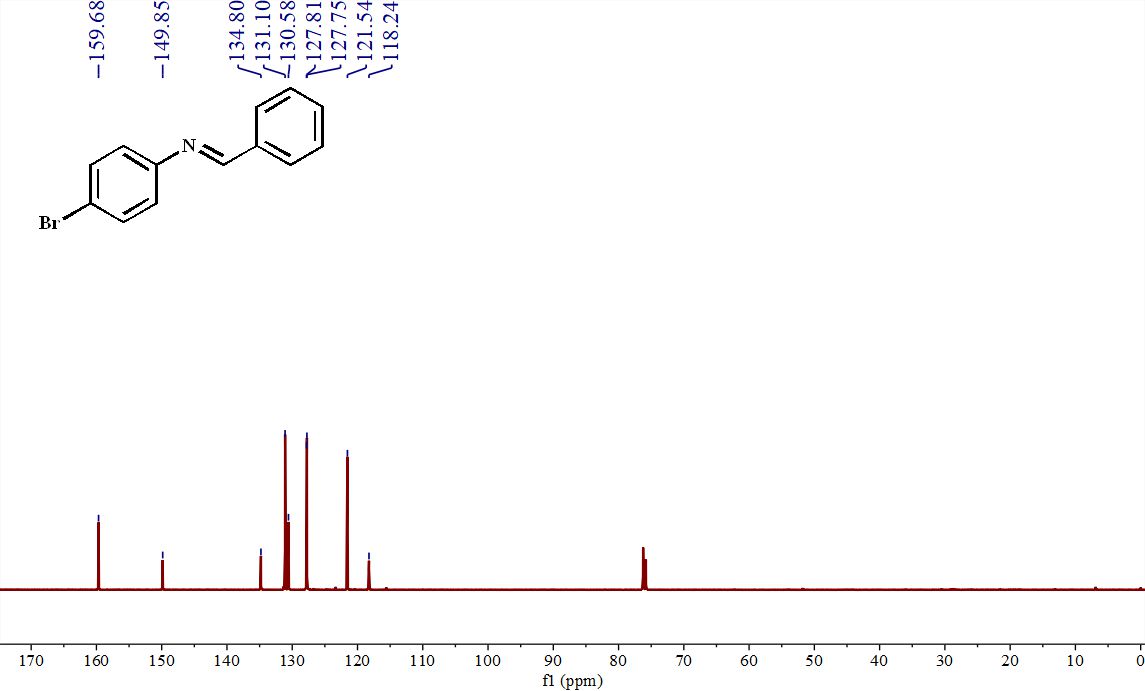


# Supplementary References

1. Wang W, Seiler M, Ivanova II, et al. In situ stopped-flow (SF) MAS NMR spectroscopy: A novel NMR technique applied for the study of aniline methylation on a solid base catalyst. Chem Commun. 2001; 15: 1362-1363.
2. Cossío FP, Alonso C, Lecea B, et al. Mechanism and stereoselectivity of the aza-wittig reaction between phosphazenes and aldehydes. J Org Chem. 2006; 71: 2839-2847.
3. Lee SH, Nikonov GI. Semi-catalytic reduction of secondary amides to imines and aldehydes. Dalton Trans. 2014; 43: 8888-8893.
4. Crampton MR, Lowry PM, Smith IJ. Kinetic and equilibrium studies of anilinoalkane-sulfonate formation. Org Biomol Chem. 2008; 6: 2405-2411.
5. Junor GP, Romero EA, Chen X, et al. Readily available primary aminoboranes as powerful reagents for aldimine synthesis. Angew Chem Int Ed. 2019; 58: 2875-2878.
6. Odom AL. New C-N and C-C bond forming reactions catalyzed by titanium complexes. Dalton Trans. 2005; 2: 225-233.
7. Chen B, Shang SS, Wang LY, et al. Mesoporous carbon derived from vitamin B_12_: A high-performance bifunctional catalyst for imine formation. Chem Commun. 2016; 52: 481-484.
8. Enholm EJ, Forbes DC, Holub DP. A route to vicinal diamines from the samarium (II) iodide-mediated coupling of aldimines. Synthetic Commun. 1990; 20: 981-987.
9. Barluenga J, Jimenez-Aquino A, Aznar F, et al. Modular synthesis of indoles from imines and *o*-dihaloarenes or *o*-chlorosulfonates by a Pd-catalyzed cascade process. J Am Chem Soc. 2009; 131: 4031-4041.
10. Schwob T, Kempe R. Ein wiederverwendbarer cobaltkatalysator für die selektive hydrierung von funktionalisierten nitroarenen und die direkte synthese von iminen und benzimidazolen aus nitroarenen und aldehyden. Angew Chem Int Ed. 2016; 128: 15400-15404.
11. Bäumler C, Kempe R. The direct synthesis of imines, benzimidazoles and quinoxalines from nitroarenes and carbonyl compounds by selective nitroarene hydrogenation employing a reusable iron catalyst. Chem Eur J. 2018; 24: 8989-8993.
12. Cheng LX, Tang JJ, Luo H, et al. Antioxidant and antiproliferative activities of hydroxyl-substituted Schiff bases. Bioorg Med Chem Lett. 2010; 20: 2417-2420.
13. Bennett JS, Charles KL, Miner MR, et al. Ethyl lactate as a tunable solvent for the synthesis of aryl aldimines. Green Chem. 2009; 11: 166-168.
14. Montalvo-Gonza´lez R, Ariza-Castolo A. Molecular structure of di-aryl-aldimines by multinuclear magnetic resonance and X-ray diffraction. J Mol Struct. 2003; 655: 375-389.
15. Troisi L, Ronzini L, Granito C, et al. Stereoselective synthesis and functionalization of 4-heterosub-stituted *β*-lactams. Tetrahedron. 2006; 62: 1564-1574.
16. Liu LH, Zhang SY, Fu XF, et al. Metal-free aerobic oxidative coupling of amines to imines. Chem Commun. 2011; 47: 10148-10150.
17. Esteruelas MA, Honczek N, Oliván M, et al. Direct access to POP-Type Osmium (II) and Osmium (IV) complexes: Osmium a promising alternative to ruthenium for the synthesis of imines from alcohols and amines. Organometallics. 2011; 30: 2468-247.
18. Patil RD, Adimurthy S. Copper‐catalyzed aerobic oxidation of amines to imines under neat conditions with low catalyst loading. Adv Synth Catal. 2011; 353: 1695-1700.
19. da Silva CM, da Silva DL, Martins CV, et al. Synthesis of aryl aldimines and their activity against fungi of clinical interest. Chem Biol Drug Des. 2011; 78: 810-815.
20. Lee CC, Liu ST. Preparation of secondary and tertiary amines from nitroarenes and alcohols. Chem Commun. 2011; 47: 6981-6983.
21. Maginnity PM, Eisenmann JL. Derivatives of o-, m-, and p-aminobenzotrifluoride. II. azomethines containing the trifluoromethyl group. J Am Chem Soc. 1952; 74: 6119-6121.
22. Neuvonen H, Neuvonen K, Fülöp F. Substituent cross-interaction effects on the electronic character of the CN bridging group in substituted benzylidene anilines-models for molecular cores of mesogenic compounds. A ^13^C NMR study and comparison with theoretical results. J Org Chem. 2006; 71: 3141-3148.
23. Montalvo-González R, Ariza-Castolo A. Molecular structure of di-aryl-aldimines by multinuclear magnetic resonance and X-ray diffraction. J Mol Struct. 2003; 655: 375-389.
24. Knöpke LR, Nemati N, Köckritz A, et al. Reaction monitoring of heterogeneously catalyzed hydrogenation of imines by coupled ATR‐FTIR, UV/Vis, and Raman spectroscopy. ChemCatChem. 2010; 2: 273-280.
25. Naeimi H, Salimi F, Rabiei K. Mild and convenient one pot synthesis of Schiff bases in the presence of P_2_O_5_/Al_2_O_3_ as new catalyst under solvent-free conditions. J Mol Catal A: Chem. 2006; 260: 100-104.
26. Zhang EL, Tian HW, Xu SD, et al. Iron-catalyzed direct synthesis of imines from amines or alcohols and amines via aerobic oxidative reactions under air. Org Lett. 2013; 15: 2704-2707.
27. Zhou P, Jiang L, Wang SG, et al. Synthesis of secondary aldimines from the hydrogenative cross-coupling of nitriles and amines over Al_2_O_3_ supported Ni catalysts. ACS Catal. 2019; 9: 8413-8423.
28. Donnelly LJ, Berthet JC, Cantat T. Selective reduction of secondary amides to imines catalysed by Schwartz's reagent. Angew Chem Int Ed. 2022; 61: e202206170.
29. Campbell KN, Sommers AH, Campbell BK. The preparation of unsymmetrical secondary aliphatic amines. J Am Chem Soc. 1944; 66 82-84.
30. Aridoss G, Laali KK. Building heterocyclic hystems with RC(OR)^2+^ carbocations in recyclable brønsted acidic ionic liquids: facile synthesis of 1‐substituted 1H‐1, 2, 3, 4‐tetrazoles, benzazoles and other ring systems with CH(OEt)_3_ and EtC(OEt)_3_ in [EtNH_3_][NO_3_] and [PMIM (SO_3_H)][OTf]. Eur J Org Chem. 2011; 15: 2827-2835.
31. Yang DS, Fu H, Hu LM, et al. Copper-catalyzed synthesis of benzimidazoles *via* cascade reactions of o-haloacetanilide derivatives with amidine hydrochlorides. J Org Chem. 2008; 73: 7841-7844.
32. Zhang ZH, Yin L, Wang YM. An expeditious synthesis of benzimidazole derivatives catalyzed by Lewis acids. Catal Commun. 2007; 8: 1126-1131.
33. Chari MA, Shobha D, Sasaki T. Room temperature synthesis of benzimidazole derivatives using reusable cobalt hydroxide (II) and cobalt oxide (II) as efficient solid catalysts. Tetrahedron Lett. 2011; 52: 5575-5580.
34. Tan KL, Park S, Ellman JA, et al. Intermolecular coupling of alkenes to heterocycles via C-H bond activation. J Org Chem. 2004; 69: 7329-7335.
35. Kim Y, Kumar MR, Park N, et al. Copper-catalyzed, one-pot, three-component synthesis of benzimidazoles by condensation and C-N bond formation. J Org Chem. 2011; 76: 9577-9583.
36. Sharghi H, Beyzavi MH, Doroodmand MM. Reusable porphyrinatoiron (III) complex supported on activated silica as an efficient heterogeneous catalyst for a facile, one‐pot, selective synthesis of 2‐arylbenzimidazole derivatives in the presence of atmospheric air as a “Green” oxidant at ambient temperature. Eur J Org Chem. 2008; 24: 4126-4138.
37. Hazra S, Pilania P, Deb M, et al. Aerobic oxidation of primary amines to imines in water using a cobalt complex as recyclable catalyst under mild conditions. Chem Eur J. 2018; 24: 15766-15771.
38. Daw P, Ben-David Y, Milstein D. Direct synthesis of benzimidazoles by dehydrogenative coupling of aromatic diamines and alcohols catalyzed by cobalt. ACS Catal. 2017; 7: 7456-7460.
39. Shi XK, Guo JM, Liu JP, et al. Unexpectedly simple synthesis of benzazoles by t-BuONa-catalyzed direct aerobic oxidative cyclocondensation of o-thio/hydroxy/ aminoanilines with alcohols under air. Chem Eur J. 2015; 21: 9988-9993.
40. Sharma S, Sharma A, Das P. Supported rhodium (Rh@PS) catalyzed benzimidazoles synthesis using ethanol/methanol as C_2_H_3_/CH source. Adv Synth Catal. 2019; 361: 67-72.
41. Kato JY, Ito Y, Ijuin R, et al. Novel strategy for synthesis of substituted benzimidazo [1, 2-a] quinolines. Org Lett. 2013; 15: 3794-3797.
42. Madabhushi S, Chinthala N, Vangipuram VS, et al. Microwave-assisted efficient one-step synthesis of amides from ketones and benzoxazoles from (2-hydroxyaryl) ketones with acetohydroxamic acid using sulfuric acid as the catalyst. Tetrahedron Lett. 2011; 52: 6103-6107.
43. Marsden SP, McGonagle AE, McKeever-Abbas B. Catalytic aza-wittig cyclizations for heteroaromatic synthesis. Org Lett. 2008; 10: 2589-2591.
44. Ueda S, Nagasawa H. Synthesis of 2‐arylbenzoxazoles by copper-catalyzed intramolecular oxidative C-O coupling of benzanilides. Angew Chem Int Ed. 2008; 47: 6411-6413.
45. Ma DW, Xie SW, Xue P, et al. Efficient and economical access to substituted benzothiazoles: Copper‐catalyzed coupling of 2‐haloanilides with metal sulfides and subsequent condensation. Angew Chem Int Ed. 2009; 48: 4222-4225.
46. Pugmire RJ, Grant DM, Robins MJ, et al. Carbon-13 magnetic resonance. XIV. Aza-analogs of polycyclic aromatic hydrocarbons. J Am Chem Soc. 1969; 91: 6381-6389.
47. Mondal A, Sahoo MK, Subaramanian M, et al. Manganese (I)-catalyzed sustainable synthesis of quinoxaline and quinazoline derivatives with the liberation of dihydrogen. J Org Chem. 2020; 85: 7181-7191.
48. Nguyen TB, Retailleau P. Al-Mourabit A. A simple and straightforward approach to quinoxalines by iron/sulfur-catalyzed redox condensation of *o*-nitroanilines and phenethylamines. Org Lett. 2013; 15: 5238-5241.
49. Aiello S, Wells G, Stone EL, et al. Synthesis and biological properties of benzothiazole, benzoxazole, and chromen-4-one analogues of the potent antitumor agent 2-(3, 4-dimethoxyphenyl)-5-fluorobenzothiazole (PMX 610, NSC 721648). J Med Chem. 2008; 51: 5135-5139.
50. Choi I, Müller V, Lole G, et al. Photoinduced heterogeneous C-H arylation by a reusable hybrid copper catalyst. Chem Eur J. 2020; 26: 3509-3514.
51. Ke F, Zhang P, Lin C, et al. Synthesis of benzimidazoles by CuI-catalyzed three-component reaction of 2-haloaniline, ammonia and aldehyde in water. Org Biomol Chem. 2018; 16: 8090-8094.
52. Das K, Mondal A, Srimani D. Selective synthesis of 2-substituted and 1, 2-disubstituted benzimidazoles directly from aromatic diamines and alcohols catalyzed by molecularly defined nonphosphine manganese (I) complex. J Org Chem. 2018; 83: 9553-9560.
